# Supplementary material for: Temporal trend of population structure, burden of diseases, healthcare resources and expenditure in China, 2000–2019
Source: BMJ Open. 2023 Jan 18;13(1):e062091. doi: 10.1136/bmjopen-2022-062091 (PMC9853154; doi:10.1136/bmjopen-2022-062091)
Supplement: Supplementary data [file bmjopen-2022-062091supp001.pdf]

Supplemental Appendix

Temporal trend of population structure, burden of diseases, healthcare resources and expenditure in China, 2000-2019

Table of Contents

Glossary and Definitions

Section 1: Population

Section 2: Health Care Institutions

Section 3: Medical Personnel

Section 4: Beds in Health Care Institutions

Section 5: Health Expenditures

Supplementary Figures

sFigure 1. Trend in life expectancy and healthy life expectancy at birth and at 60─64 years according to gender between 2000 and 2019.

Supplementary Tables

sTable 1. The average annual percent change in life expectancy at birth and at 60─64 years for 2000─2019

sTable 2. Percentage of healthy life expectancy within total life expectancy at birth for 2000─2019

sTable 3. Percentage of healthy life expectancy within total life expectancy at 60─64 years for 2000─2019

sTable 4. The average annual percent change of all-cause disease burden for 2000─2019

sTable 5. The average annual percent change of causes for 2000─2019

sTable 6. The average annual percent change of risk factors for 2000–2019

Glossary and Definitions

Glossary and definitions of population, healthcare institutions, medical personnel, beds in health care institutions, and health expenditure data were compiled from the 2020 China Statistical Yearbook (CSY) (http://www.stats.gov.cn/tjsj/ndsj/2020/indexeh.htm).

Section 1: Population

Total Population (year-end) (10000 persons): Year-end population refers to the population at midnight on 31 December. Annual statistics regarding the total population do not include data for Hong Kong, the Macau special administrative region and Taiwan province, and overseas numbers.

Urban Population refers to people residing in cities and towns in China.

Rural Population refers to people who reside outside of urban areas.

Birth Rate, Death Rate, and Natural Growth Rate of Population

Birth Rate (or Crude Birth Rate) refers to the ratio of the number of births to the average population (or mid-period population) during a certain period of time (usually a year). Birth rate in the chapter refers to annual birth rate and it is calculated as: number of births / annual average population × 1000. Number of births in the formula refers to live births (i.e., when a baby has breathed or showed any vital phenomena regardless of duration of gestation). Annual average population is the average of the population at the beginning of the year and the population at the end of the year. Sometimes it is substituted by the mid-year population.

Death Rate (or Crude Death Rate) refers to the ratio of the number of deaths to the average population (or mid-period population) during a certain period of time (usually a year). Death rate in the chapter refers to annual death rate and is calculated as: number of deaths / annual average population × 1000. Total Population (year-end) (10000 persons) refers to the population at midnight on 31 December.

Gross Dependency Ratio (or Coefficient) (%) refers to the ratio of non-working-age population to the working-age population. In general, Gross Dependency Ratio describes the number of non-working-age individuals that every 100 people of working age take care of. This reflects the basic relation between population and economic development from a demographic perspective.

Children Dependency Ratio (or Coefficient) (%) refers to the ratio of children to the working-age population. It describes the number of children that every 100 people of working age take care of.

Old Dependency Ratio (or Coefficient) (%) refers to the ratio of the elderly to the working-age population. It describes the number of elderly that every 100 people of working age take care of. Old dependency ratio is one of the indicators that reflect the social implication of population aging from an economic perspective.

Note: For the years, 2000 and 2010, census year estimates were available; the rest of the data were estimated based on annual national sample surveys.

Section 2: Health Care Institutions

Number of Health Care Institutions (unit)

Medical and health care institutions refer to units which have qualified to receive a Certification of Health Care Institution by the administration of public health, or a Certification of Corporate Unit by the civil affairs, administration for industry and commerce, commission office for public sector reform. Health care institutions engage in medical care, disease prevention and control, health supervision and inspection, medicine research, and on-job training. Health care institutions include hospitals, health care institutions at the grass-root level, specialized public health institutions, and other medical and health care institutions.

Number of Hospitals (unit)

Hospitals were considered to include general hospitals, hospitals of traditional Chinese medicine, hospitals that integrate traditional Chinese medicine and Western medicine, national hospitals, specialized hospitals, and nursing hospitals. Specialized disease prevention and cure centers, maternal and child health hospitals, and sanatoriums were excluded.

Health Care Institutions at Grass-root Level (unit)

Health care institutions at the grass-root level included: community health service centers, community service stations, institutes of health on the street, institutes of health in villages and towns, outpatient departments, and clinics (health centers).

Note:
1. Number of village clinics was included in health care institutions.
2. Since 2002, health care institutions no longer include headquarters of higher and secondary medical schools, drug test institutions, frontier health and quarantine institutions, and family.

Section 3: Medical Personnel

Number of Medical Personnel (10000 persons)

Medical personnel refer to all employees engaged at health care institutions, such as hospitals, health care institutions at grass-root level, specialized public health institutions, and other medical and health care institutions. These employees include medical technical personnel, village doctors and assistants, other technical personnel, and managerial and service staff. Medical personnel also refer to workers with payment of wages at the end of the year, including various types of employment personnel (including contract workers) and staff rehired for more than six months. Temporary workers, emeritus and retired, persons leaving the unit while remaining labor relations, and rehired and temporarily employed individuals for less than six months were excluded.

Number of Licensed Doctors (10000 persons)

Licensed doctors refer to medical workers who have obtained licenses of qualified doctors and are employed in medical treatment, disease prevention, or healthcare institutions. Licensed doctors engaged in management positions were excluded. Licensed doctors are divided into four categories: clinician, Chinese medicine physician, dentist, and public health physician.

Number of Registered Nurses (10000 persons)

Registered nurses refer to practical nursing personnel with a certificate of registered nurse. Nurses engaged in management positions were excluded.

Number of Other Technical Personnel (10000 persons)

Other technical personnel represent staff who graduated from high school and other institutions in the majors of chemistry and mathematics, as well as other non-health professionals, who are engaged in health promotion, research, teaching, and other technical work.

Number of Administrative Personnel (10000 persons)

Administrative personnel include persons in charge, personnel mainly engaged in health care, disease control, health surveillance, medical research, and teaching, other business management, and personnel mainly engaged in the party and government, personnel, finance, information, security, and other administrative work.

Note: Data of medical personnel and medical technical personnel include 10000 sanitation supervisors who serve as public servants.

Section 4: Beds in Health Care Institutions

Number of Beds in Health Care Institutions (10000 units)

Number of beds in health care institutions refers to a fixed sum of beds (non-prepared beds) at the end of the year. These include formal beds, simple beds, care beds, beds being disinfected and repaired, and disabled beds due to expansion or overhaul. Obstetric neonatal beds, expectant beds of confinement rooms, inventory beds, observation beds, extra beds, and escort beds for patient's family members were excluded.

Number of Beds in Medical Institutions per 10000 Population

Number of beds in medical institutions refers to a fixed sum of beds (non-prepared beds) at the end of the year. These include formal beds, simple beds, care beds, beds being disinfected and repaired, and disabled beds due to expansion or overhaul. Obstetric neonatal beds, expectant beds of confinement rooms, inventory beds, observation beds, extra beds, and escort beds for patient's family members were excluded.

Number of Beds in Urban Medical Institutions (10000 units)

Number of beds in urban medical institutions refers to a fixed sum of beds (non-prepared beds) at the end of the year. These include formal beds, simple beds, care beds, beds being disinfected and repaired, and disabled beds due to expansion or overhaul. Obstetric neonatal beds, expectant beds of confinement rooms, inventory beds, observation beds, extra beds, and escort beds for patient's family members were excluded.

Number of Beds in Rural Medical Institutions (10000 units)

Number of beds in rural medical institutions refers to a fixed sum of beds (non-prepared beds) at the end of the year. These include formal beds, simple beds, care beds, beds being disinfected and repaired, and disabled beds due to expansion or overhaul. Obstetric neonatal beds, expectant beds of confinement rooms, inventory beds, observation beds, extra beds, and escort beds for patient's family members were excluded.

Beds of Medical Institutions per 10000 Population

Beds of Medical Institutions per 10000 Population = number of beds of medical and health care institutions / population ×10000. The population value used is based on household registration data from the Ministry of Public Security.

Section 5: Health Expenditures

Total Health Expenditure (100 million yuan)

Total health expenditure refers to the total expenditure on medical and health care services (usually in monetary value) by a country or region within a certain period of time (usually a year). Total health expenditure is counted according to the source method. Under certain economic conditions, it reflects the emphasis and burden level of cost on government health care, the society's healthcare, and on individual citizens. Total health expenditure also represents the main features of health financing mode and fairness, as well as the rationality of health financing.

Government Health Expenditure (100 million yuan)

Government expenditure on public health refers to expenditures by the government at all levels of medical and health care services, medical subsidies, health administration and health insurance management, and undertakings of family planning.

Social Health Expenditure (100 million yuan)

Social expenditure on public health refers to all inputs from society, and not from the government in the form of expenditures on social medical security, commercial health insurance, private expenditure on operation of medical and health care, social donations and contributions, and income from administrative fees.

Out-of-pocket Health Expenditure (100 million yuan)

Individual cash expenditure on health refers to the expenditure in cash by rural and urban residents for various health services, including self-payments of residents within the system of multi-medical insurance.

Per Capita Health Expenditure (yuan)

Per capita health expenditure refers to the ratio of total expenditure on health in a year to the average population.

Supplementary Figures

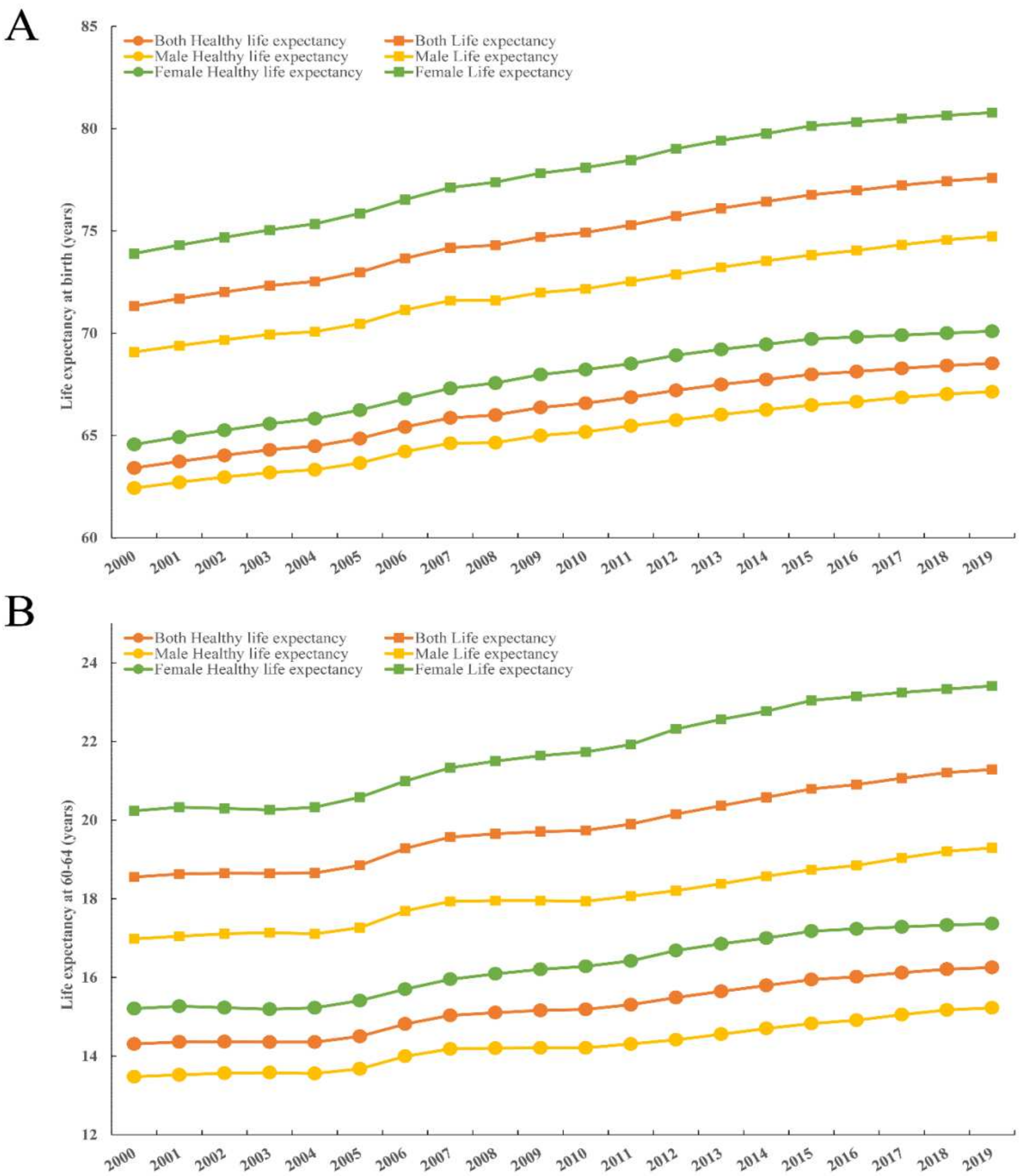

sFigure 1. Trend in life expectancy and healthy life expectancy at birth and at 60–64 years according to gender between 2000 and 2019.

The trend of life expectancy and healthy life expectancy at birth (A) and at 60–64 years (B) according to gender between 2000 and 2019. The distance between life expectancy and healthy life expectancy denotes unhealthy life expectancy (ill-span).

Supplementary Tables

sTable 1. The average annual percent change in life expectancy at birth and at 60–64 years for 2000–2019

	Life expectancy (95% UI)		AAPC (95% CI)		
	2000	2019	2000–2019	2000–2009	2010–2019
**Life expectancy at birth**					
Both					
Life expectancy	71.33 (70.56 to 72.06)	77.59 (76.27 to 79.00)	0.44 (0.43 to 0.46)*	0.50 (0.47 to 0.53)*	0.40 (0.38 to 0.42)*
Healthy life expectancy	63.42 (60.07 to 65.54)	68.53 (60.88 to 70.97)	0.41 (0.38 to 0.43)*	0.50 (0.45 to 0.54)*	0.31 (0.29 to 0.34)*
Male					
Life expectancy	69.08 (68.02 to 70.16)	74.74 (72.78 to 76.70)	0.42 (0.38 to 0.46)*	0.46 (0.37 to 0.54)*	0.39 (0.37 to 0.41)*
Healthy life expectancy	62.43 (60.44 to 64.36)	67.14 (60.64 to 69.82)	0.38 (0.36 to 0.41)*	0.44 (0.39 to 0.49)*	0.32 (0.30 to 0.35)*
Female					
Life expectancy	73.90 (72.91 to 74.81)	80.78 (79.10 to 82.47)	0.47 (0.43 to 0.51)*	0.57 (0.50 to 0.63)*	0.37 (0.32 to 0.41)*
Healthy life expectancy	64.57 (60.86 to 66.96)	70.10 (60.92 to 73.19)	0.43 (0.40 to 0.46)*	0.56 (0.50 to 0.62)*	0.30 (0.27 to 0.33)*
**Life expectancy at 60–64 years**					
Both					
Life expectancy	18.56 (18.06 to 19.02)	21.29 (20.32 to 22.33)	0.76 (0.60 to 0.91)*	0.65 (0.37 to 0.93)*	0.91 (0.81 to 1.01)*
Healthy life expectancy	14.31 (13.16 to 15.43)	16.26 (14.82 to 17.67)	0.69 (0.51 to 0.87)*	0.64 (0.27 to 1.01)*	0.73 (0.66 to 0.80)*
Male					
Life expectancy	16.99 (16.32 to 17.66)	19.29 (17.97 to 20.68)	0.68 (0.61 to 0.74)*	0.58 (0.46 to 0.70)*	0.85 (0.80 to 0.89)*
Healthy life expectancy	13.48 (12.42 to 14.53)	15.23 (13.82 to 16.79)	0.65 (0.55 to 0.76)*	0.56 (0.37 to 0.75)*	0.81 (0.76 to 0.86)*
Female					
Life expectancy	20.24 (19.62 to 20.86)	23.41 (22.10 to 24.77)	0.76 (0.55 to 0.98)*	0.75 (0.41 to 1.09)*	0.76 (0.47 to 1.04)*
Healthy life expectancy	15.21 (13.83 to 16.46)	17.37 (15.61 to 19.16)	0.70 (0.54 to 0.85)*	0.69 (0.40 to 0.98)*	0.68 (0.54 to 0.81)*

Note: AAPC: average annual percent changes; UI, uncertainty interval; CI, confidence interval.
*AAPC significantly differs from zero at the alpha = 0.05 level.

sTable 2. Percentage of healthy life expectancy within total life expectancy at birth for 2000–2019

Year	Healthy life expectancy at birth (years)			Life expectancy at birth (years)			Healthy within total life expectancy (%)		
	Total	Male	Female	Total	Male	Female	Total	Male	Female
2000	63.42	62.43	64.57	71.33	69.08	73.90	88.90	90.38	87.37
2001	63.73	62.72	64.92	71.69	69.40	74.31	88.90	90.37	87.37
2002	64.03	62.96	65.26	72.02	69.67	74.69	88.90	90.37	87.38
2003	64.30	63.20	65.57	72.33	69.94	75.05	88.89	90.36	87.38
2004	64.49	63.33	65.83	72.54	70.08	75.34	88.90	90.37	87.37
2005	64.86	63.66	66.25	72.98	70.47	75.86	88.87	90.34	87.33
2006	65.42	64.22	66.79	73.67	71.14	76.54	88.80	90.27	87.27
2007	65.87	64.62	67.31	74.19	71.60	77.12	88.79	90.25	87.27
2008	66.00	64.65	67.57	74.31	71.61	77.38	88.83	90.28	87.32
2009	66.38	65.00	67.98	74.70	71.99	77.82	88.85	90.30	87.35
2010	66.58	65.18	68.22	74.92	72.17	78.10	88.87	90.31	87.36
2011	66.88	65.48	68.51	75.29	72.53	78.46	88.82	90.27	87.31
2012	67.21	65.75	68.92	75.73	72.88	79.03	88.75	90.22	87.21
2013	67.50	66.03	69.21	76.11	73.23	79.42	88.69	90.17	87.14
2014	67.74	66.26	69.45	76.44	73.53	79.76	88.62	90.11	87.08
2015	67.99	66.49	69.72	76.77	73.83	80.14	88.56	90.06	87.00
2016	68.12	66.65	69.82	76.98	74.05	80.32	88.49	90.01	86.93
2017	68.28	66.86	69.91	77.23	74.33	80.50	88.41	89.95	86.85
2018	68.42	67.03	70.01	77.44	74.57	80.65	88.36	89.89	86.81
2019	68.53	67.14	70.10	77.59	74.74	80.78	88.31	89.84	86.78

sTable 3. Percentage of healthy life expectancy within total life expectancy at 60–64 years for 2000–2019

Year	Healthy life expectancy at 60–64 years			Life expectancy at 60–64 year			Percentage of healthy within total life expectancy		
	Total	Male	Female	Total	Male	Female	Total	Male	Female
2000	14.31	13.48	15.21	18.56	16.99	20.24	77.12%	79.35%	75.15%
2001	14.36	13.52	15.27	18.63	17.05	20.33	77.09%	79.33%	75.11%
2002	14.37	13.57	15.23	18.65	17.11	20.30	77.05%	79.29%	75.06%
2003	14.36	13.58	15.19	18.65	17.14	20.26	76.99%	79.24%	74.98%
2004	14.36	13.56	15.23	18.66	17.11	20.33	76.97%	79.24%	74.93%
2005	14.50	13.68	15.41	18.85	17.27	20.58	76.93%	79.21%	74.87%
2006	14.82	14.00	15.70	19.29	17.69	20.99	76.84%	79.12%	74.80%
2007	15.04	14.18	15.95	19.57	17.93	21.33	76.82%	79.09%	74.79%
2008	15.11	14.20	16.09	19.65	17.95	21.50	76.87%	79.12%	74.84%
2009	15.16	14.21	16.21	19.71	17.95	21.64	76.93%	79.17%	74.90%
2010	15.19	14.21	16.28	19.74	17.94	21.73	76.96%	79.21%	74.93%
2011	15.31	14.31	16.42	19.90	18.07	21.93	76.94%	79.20%	74.89%
2012	15.49	14.42	16.69	20.15	18.21	22.32	76.87%	79.19%	74.78%
2013	15.65	14.56	16.86	20.37	18.39	22.56	76.82%	79.18%	74.71%
2014	15.80	14.71	17.00	20.58	18.58	22.77	76.77%	79.16%	74.65%
2015	15.95	14.83	17.18	20.79	18.74	23.04	76.69%	79.13%	74.55%
2016	16.02	14.91	17.23	20.91	18.85	23.15	76.62%	79.11%	74.45%
2017	16.12	15.05	17.28	21.07	19.04	23.25	76.54%	79.08%	74.35%
2018	16.21	15.17	17.33	21.21	19.21	23.33	76.45%	78.99%	74.28%
2019	16.26	15.23	17.37	21.29	19.29	23.41	76.36%	78.93%	74.18%

sTable 4. The average annual percent change of all-cause disease burden for 2000–2019

Subcategory	All-cause estimates (95% UI)		AAPC (95% CI)		
	2000	2019	2000–2019	2000–2009	2010–2019
**Number**					
Incidence	5,291,848,308 (4,993,009,197 to 5,644,956,487)	5,790,243,111 (5,505,879,019 to 6,107,248,437)	0.48 (0.45 to 0.50)*	0.19 (0.16 to 0.22)*	0.82 (0.78 to 0.85)*
Prevalence	1,241,468,252 (1,234,531,622 to 1,247,534,242)	1,362,817,881 (1,356,594,402 to 1,368,714,232)	0.49 (0.49 to 0.50)*	0.47 (0.47 to 0.48)*	0.52 (0.51 to 0.52)*
Death	8,653,295 (8,170,487 to 9,200,531)	10,653,448 (9,310,978 to 12,069,760)	1.12 (0.93 to 1.31)*	0.89 (0.61 to 1.18)*	1.27 (1.03 to 1.51)*
Years lived with disability	116,137,933 (86,494,934 to 150,490,082)	153,797,874 (115,409,603 to 199,426,123)	1.49 (1.46 to 1.52)*	1.29 (1.24 to 1.34)*	1.74 (1.72 to 1.77)*
Years of life lost	260,536,746 (246,148,674 to 276,354,908)	228,407,694 (197,155,841 to 260,733,993)	-0.70 (-0.95 to -0.46)*	-1.34 (-1.53 to -1.15)*	-0.07 (-0.51 to 0.38)
DALY	376,674,679 (343,098,150 to 416,187,725)	382,205,568 (332,307,099 to 433,824,950)	0.08 (-0.06 to 0.22)	-0.44 (-0.58 to -0.31)*	0.60 (0.39 to 0.82)*
Risk factor (DALY)	176,792,905 (163,998,804 to 190,556,819)	187,705,961 (165,023,927 to 212,244,649)	0.34 (0.20 to 0.48)*	-0.27 (-0.52 to -0.03)*	0.92 (0.79 to 1.06)*
**Age standardized rate**					
Incidence	414,111 (390,563 to 440,957)	418,081 (394,196 to 444,709)	0.04 (0.02 to 0.07)*	-0.21 (-0.24 to -0.17)*	0.34 (0.30 to 0.38)*
Prevalence	94,824 (94,187 to 95,367)	93,439 (92,723 to 94,090)	-0.08 (-0.09 to -0.07)*	-0.17 (-0.19 to -0.15)*	0.00 (-0.01 to 0.00)
Death	956 (910 to 1,010)	634 (561 to 711)	-2.14 (-2.28 to -2.00)*	-2.00 (-2.20 to -1.79)*	-2.38 (-2.56 to -2.21)*
Years lived with disability	9,078 (6,779 to 11,713)	8,924 (6,654 to 11,539)	-0.08 (-0.12 to -0.05)*	-0.34 (-0.38 to -0.30)*	0.22 (0.17 to 0.28)*
Years of life lost	24,833 (23,513 to26,239)	13,347 (11,701 to14,997)	-3.22 (-3.33 to -3.11)*	-3.43 (-3.59 to -3.27)*	-3.09 (-3.23 to -2.96)*
DALY	33,911 (31,131 to 37,035)	22,271 (19,482 to 25,195)	-2.21 (-2.35 to -2.07)*	-2.54 (-2.64 to -2.43)*	-1.85 (-2.12 to -1.58)*
Risk factor (DALY)	16,689 (15,569 to 17,886)	10,310 (9,122 to 11,524)	-2.49 (-2.69 to -2.29)*	-2.93 (-3.27 to -2.58)*	-2.07 (-2.30 to -1.85)*

Note: AAPC: average annual percent changes; UI, uncertainty interval; CI, confidence interval; DALY, disability-adjusted life years. *AAPC significantly differs from zero at the alpha = 0.05 level.

sTable 5. The average annual percent change of causes for 2000–2019

Causes	DALY (95% UI)				AAPC (95% CI)		
	2000	Rank	2019	Rank	2000–2019	2000–2009	2010–2019
**All-age numbers**							
Neglected tropical diseases and malaria	1,704,065 (1,121,580 to 2,525,119)	21	1,137,806 (692, 480 to 1,800,439)	22	-2.07 (-2.35 to -1.79)*	-3.57 (-3.89 to -3.25)*	-0.50 (-0.97 to -0.02)*
Nutritional deficiencies	4,183,588 (3,082,941 to 5,605,252)	19	2,606,926 (1,815,566 to 3,659,294)	18	-2.47 (-2.64 to -2.30)*	-4.30 (-4.49 to -4.11)*	-0.53 (-0.79 to -0.26)*
Neoplasms	54,415,394 (50,247,614 to 58,721,812)	2	67,519,830 (1,418,891 to 3,248,407)	2	1.13 (0.95 to 1.31)*	0.87 (0.57 to 1.17)*	1.42 (1.20 to 1.65)*
Cardiovascular diseases	67,584,492 (62,771,497 to 73,832,854)	1	91,933,122 (1,135,346 to 3,124,922)	1	1.62 (1.45 to 1.79)*	1.70 (1.40 to 2.00)*	1.42 (1.32 to 1.53)*
Chronic respiratory diseases	28,653,966 (23,770,991 to 30,931,146)	3	22,520,549 (1,719,779 to 3,416,880)	4	-1.28 (-1.50 to -1.06)*	-2.02 (-2.40 to -1.65)*	-0.50 (-0.75 to -0.26)*
Digestive diseases	11,699,071 (10,590,221 to 12,920,126)	12	10,017,880 (1,549,912 to 3,496,363)	12	-0.80 (-0.87 to -0.73)*	-1.02 (-1.13 to -0.90)*	-0.51 (-0.60 to -0.43)*
Neurological disorders	12,506,303 (6,994,212 to 20,592,252)	11	17,381,406 (1,666,405 to 3,082,962)	7	1.75 (1.67 to 1.82)*	1.79 (1.70 to 1.89)*	1.72 (1.61 to 1.84)*
Mental disorders	18,110,029 (13,449,072 to 23,810,039)	9	20,293,827 (1,145,615 to 3,345,133)	5	0.59 (0.49 to 0.69)*	0.45 (0.40 to 0.49)*	0.75 (0.54 to 0.95)*
Musculoskeletal disorders	20,089,552 (14,362,360 to 26,771,117)	7	29,933,924 (1,427,199 to 3,823,644)	3	2.10 (2.04 to 2.16)*	2.44 (2.36 to 2.52)*	1.75 (1.67 to 1.83)*
Other non-communicable diseases	24,572,991 (20,333,568 to 29,891,261)	4	19,126,072 (1,446,224 to 3,590,726)	6	-1.31 (-1.48 to -1.13)*	-2.43 (-2.53 to -2.33)*	-0.04 (-0.40 to -032)
Skin and subcutaneous diseases	7,809,025 (5,108,660 to 11,567,830)	16	8,264,702 (1,394,816 to 3,356,265)	13	0.29 (0.27 to 0.32)*	0.33 (0.29 to 0.38)*	0.28 (0.26 to 0.29)*
Sense organ diseases	10,740,782 (7,311,356 to 15,203,366)	14	15,981,667 (1,788,420 to 3,997,480)	9	2.14 (1.98 to 2.30)*	1.98 (1.94 to 2.03)*	2.31 (1.97 to 2.64)*
Transport injuries	16,631,845 (15,221,113 to 18,940,342)	10	13,745,455 (1,719,684 to 3,768,996)	11	-1.02 (-1.35 to -0.69)*	0.57 (0.21 to 0.95)*	-2.66 (-3.22 to -2.10)*
Unintentional injuries	21,114,052 (19,362,287 to 23,062,953)	6	16,003,462 (1,056,699 to 3,613,615)	8	-1.53 (-2.01 to -1.04)*	-1.53 (-2.01 to -1.04)*	-1.53 (-2.01 to -1.04)*
Self-harm and interpersonal violence	11,171,276 (9,775,023 to 12,180,298)	13	5,870,049 (1,030,733 to 3,924,034)	16	-3.35 (-3.70 to -3.01)*	-4.09 (-4.73 to -3.45)*	-2.47 (-2.75 to -2.18)*
HIV/AIDS and sexually transmitted infections	921,444,444 (727,466,466 to 1,222,963)	22	1,752,419 (1,430, 192 to 2,114,213)	19	3.47 (2.72 to 4.22)*	3.07 (2.38 to 3.77)*	4.07 (2.71 to 5.45)*
Respiratory infections and tuberculosis	21,938,222 (20,220,847 to 23,581,022)	5	6,647,872 (1,853,547 to 3,613,257)	15	-6.10 (-6.41 to -5.79)*	-8.88 (-9.36 to -8.40)*	-3.34 (-3.65 to -3.03)*
Enteric infections	3,407,267 (2,990,655 to 3,948,769)	20	1,408,098 (1,047,418 to 3,827,650)	20	-4.41 (-5.28 to -3.54)*	-9.05 (-9.96 to -8.13)*	0.68 (-0.56 to 1.92)
Other infectious diseases	4,581,989 (3,724,438 to 6,219,671)	18	1,244,099 (1,063,407 to 3,462,432)	21	-6.57 (-6.95 to -6.19)*	-9.03 (-9.45 to -8.61)*	-4.04 (-4.71 to -3.36)*
Maternal and neonatal disorders	18,110,589 (16,543,873 to 19,663,056)	8	7,151,571 (1,273,977 to 3,174,922)	14	-4.82 (-5.40 to -4.23)*	-6.06 (-6.60 to -5.51)*	-3.56 (-4.60 to -2.51)*
Substance use disorders	6,291,914 (5,043,188 to 7,751,372)	17	5,755,658 (1,373,805 to 3,343,432)	17	-0.39 (-0.82 to 0.03)	-1.74 (-2.49 to -0.99)*	0.94 (0.53 to 1.36)*
Diabetes and kidney diseases	10,436,824 (9,083,252 to 11,910,785)	15	15,909,173 (1,346,885 to 3,824,247)	10	2.30 (2.09 to 2.51)*	2.87 (2.44 to 3.31)*	1.79 (1.70 to 1.89)*
**Age-standardized rate**							
Neglected tropical diseases and malaria	132 (89 to 194)	21	68 (42 to 107)	22	-3.40 (-3.66 to -3.13)*	-4.75 (-5.06 to -4.45)*	-1.97 (-2.42 to -1.51)*
Nutritional deficiencies	369 (284 to 480)	20	175 (122 to 241)	18	-3.88 (-4.07 to -3.69)*	-5.98 (-6.27 to -5.70)*	-1.84 (-2.11 to -1.58)*
Neoplasms	4,505 (4,163 to 4,847)	2	3,421 (2,933 to 3,946)	2	-1.46 (-1.71 to -1.21)*	-1.62 (-1.88 to -1.36)*	-1.25 (-1.68 to -1.81)*
Cardiovascular diseases	6,441 (5,997 to 7,005)	1	4,938 (4,318 to 5,577)	1	-1.40 (-1.56 to -1.24)*	-1.10 (-1.39 to -0.82)*	-1.84 (-1.94 to -1.75)*
Chronic respiratory diseases	2,947 (2,458 to 3,175)	3	1,270 (1,114 to 1,474)	5	-4.34 (-4.61 to -4.07)*	-4.90 (-5.37 to -4.44)*	-3.74 (-4.04 to -3.45)*
Digestive diseases	974 (889 to 1,070)	12	539 (463 to 616)	14	-3.07 (-3.14 to -3.00)*	-3.29 (-3.41 to -3.16)*	-2.77 (-2.84 to -2.71)*
Neurological disorders	1,103 (650 to 1,773)	11	1,077 (598 to 1,780)	8	-0.15 (-0.24 to -0.06)*	-0.09 (-0.16 to -0.02)*	-0.22 (-0.40 to -0.03)*
Mental disorders	1,317 (978 to 1,727)	9	1,248 (922 to 1,627)	6	-0.27 (-0.34 to -0.20)*	-0.56 (-0.59 to -0.53)*	0.01 (-0.13 to 0.14)

Musculoskeletal disorders	1,536 (1,102 to 2,050)	8	1,585 (1,140 to 2,112)	3	0.16 (0.10 to 0.23)*	0.31 (0.26 to 0.36)*	0.02 (-0.11 to 0.15)
Other non-communicable diseases	2,315 (1,963 to 2,719)	5	1,349 (1,066 to 1,726)	4	-2.81 (-2.96 to -2.67)*	-3.49 (-3.65 to -3.34)*	-2.00 (-2.25 to -1.75)*
Skin and subcutaneous diseases	606 (397 to 898)	16	606 (391 to 909)	13	0.00 (-0.01 to 0.01)	-0.05 (-0.07 to -0.04)*	0.06 (0.03 to 0.08)*
Sense organ diseases	926 (633 to 1,299)	13	863 (591 to 1,226)	9	-0.36 (-0.45 to -0.27)*	-0.46 (-0.49 to -0.43)*	-0.25 (-0.43 to -0.07)*
Transport injuries	1,248 (1,146 to 1,433)	10	860 (740 to 975)	10	-1.98 (-2.42 to -1.54)*	-0.63 (-1.14 to -0.13)*	-3.39 (-4.12 to -2.65)*
Unintentional injuries	1,886 (1,744 to 2,045)	7	1,102 (919 to 1,254)	7	-2.81 (-3.24 to -2.39)*	-2.81 (-3.24 to -2.39)*	-2.81 (-3.24 to -2.39)*
Self-harm and interpersonal violence	807 (708 to 877)	15	361 (310 to 421)	16	-4.19 (-4.62 to -3.76)*	-5.13 (-5.93 to -4.32)*	-3.08 (-3.43 to -2.73)*
HIV/AIDS and sexually transmitted infections	78 (56 to 114)	22	116 (92 to 147)	19	2.02 (1.50 to 2.53)*	2.61 (2.31 to 2.91)*	1.88 (1.05 to 2.72)*
Respiratory infections and tuberculosis	2,396 (2,209 to 2,582)	4	518 (458 to 593)	15	-7.77 (-7.93 to -7.61)*	-9.56 (-9.70 to -9.41)*	-5.88 (-6.18 to -5.58)*
Enteric infections	369 (326 to 422)	19	111 (84 to 144)	20	-6.04 (-6.52 to -5.56)*	-10.47 (-11.00 to -9.94)*	-1.52 (-2.31 to -0.73)*
Other infectious diseases	473 (376 to 659)	17	110 (92 to 131)	21	-7.37 (-7.84 to -6.91)*	-8.51 (-9.21 to -7.80)*	-6.04 (-6.58 to -5.50)*
Maternal and neonatal disorders	2,177 (1,983 to 2,375)	6	792 (699 to 897)	12	-5.26 (-5.36 to -5.16)*	-5.26 (-5.36 to -5.16)*	-5.26 (-5.36 to -5.16)*
Substance use disorders	428 (345 to 526)	18	347 (262 to 443)	17	-1.03 (-1.38 to -0.69)*	-2.60 (-3.02 to -2.18)*	0.85 (0.35 to 1.36)*
Diabetes and kidney diseases	876 (764 to 997)	14	817 (688 to 961)	11	-0.32 (-0.47 to -0.16)*	0.46 (0.17 to 0.75)*	-1.01 (-1.12 to -0.90)*

Note: AAPC: average annual percent changes; UI, uncertainty interval; CI, confidence interval.
*AAPC significantly differs from zero at the alpha = 0.05 level.

sTable 6. The average annual percent change of risk factors for 2000–2019

Risk Factors	DALY (95% UI)				AAPC (95% CI)		
	2000	Rank	2019	Rank	2000–2019	2000–2009	2010–2019
**All-age numbers**							
Unsafe water, sanitation, and handwashing	4,234,714 (3,363,558 to 5,211,019)	15	1,152,504 (805,846 to 1,554,455)	18	-6.60 (-7.28 to -5.91)*	-10.80 (-11.04 to -10.55)*	-2.34 (-3.77 to -0.88)*
Air pollution	49,335,959 (43,328,149 to 55,138,636)	2	42,509,703 (36,337,918 to 49,482,918)	4	-0.78 (-0.98 to -0.58)*	-1.17 (-1.53 to -0.82)*	-0.46 (-0.68 to -0.24)*
Other environmental risks	5,386,702 (3,749,375 to 7,246,194)	14	6,306,070 (4,075,171 to 8,773,453)	13	0.85 (0.72 to 0.98)*	1.14 (0.91 to 1.38)*	0.46 (0.38 to 0.53)*
Child and maternal malnutrition	27,730,286 (24,920,872 to 30,776,034)	5	7,253,783 (6,277,469 to 8,340,678)	12	-6.81 (-7.62 to -5.99)*	-8.92 (-9.38 to -8.47)*	-4.75 (-6.37 to -3.10)*
Tobacco	57,264,585 (52,395,093 to 62,486,440)	1	64,116,000 (54,575,903 to 75,181,983)	1	0.60 (0.42 to 0.78)*	0.30 (0.02 to 0.58)*	0.90 (0.66 to 1.15)*
Alcohol use	14,673,585 (12,536,787 to 17,097,523)	8	17,265,060 (13,765,337 to 21,321,000)	8	0.77 (0.68 to 0.85)*	0.77 (0.68 to 0.85)*	0.77 (0.68 to 0.85)*
Drug use	6,301,278 (5,433,686 to 7,296,963)	13	5,121,794 (4,187,484 to 6,121,198)	14	-1.03 (-1.22 to -0.84)*	-3.28 (-3.50 to -3.07)*	1.54 (1.21 to 1.87)*
High fasting plasma glucose	17,924,145 (14,527,382 to 21,999,850)	7	28,228,439 (22,052,698 to 35,375,022)	5	2.36 (1.82 to 2.90)*	3.64 (2.83 to 4.47)*	1.11 (0.41 to 1.81)*
High systolic blood pressure	35,341,494 (30,227,215 to 40,383,079)	3	54,441,615 (45,474,002 to 63,689,070)	2	2.32 (2.18 to 2.46)*	2.66 (2.40 to 2.91)*	1.84 (1.77 to 1.92)*
High body-mass index	11,704,488 (3,880,854 to 22,527,461)	9	24,830,041 (11,788,976 to 40,545,899)	6	4.02 (3.74 to 4.29)*	4.33 (3.83 to 4.83)*	3.59 (3.48 to 3.70)*
Low bone mineral density	1,769,378 (1,477,619 to 2,061,186)	16	3,320,275 (2,594,480 to 4,004,265)	15	3.39 (3.12 to 3.67)*	4.14 (3.73 to 4.56)*	2.78 (2.42 to 3.15)*
Dietary risks	34,253,322 (27,427,086 to 43,429,953)	4	46,813,126 (35,644,728 to 60,011,858)	3	1.66 (1.53 to 1.78)*	1.97 (1.74 to 2.21)*	1.28 (1.21 to 1.35)*
Low physical activity	1,275,222 (632,556 to 2,484,126)	18	2,507,120 (1,195,139 to 4,842,346)	17	3.54 (2.70 to 4.38)*	3.88 (2.39 to 5.39)*	3.03 (2.65 to 3.41)*
Occupational risks	18,571,331 (16,040,002 to 21,379,719)	6	15,486,849 (12,897,016 to 18,259,216)	9	-0.97 (-1.12 to -0.82)*	-1.51 (-1.78 to -1.24)*	-0.40 (-0.52 to -0.29)*
Intimate partner violence	789,841 (433,187 to 1,234,095)	19	789,926,926 (354,381 to 1,304,433)	19	0.07 (-0.17 to 0.30)	-0.50 (-0.78 to -0.22)*	0.76 (0.39 to 1.14)*
Unsafe sex	1,482,696 (1,293,280 to 1,925,395)	17	2,724,797 (1,976,323 to 3,277,196)	16	3.20 (2.90 to 3.49)*	3.72 (3.50 to 3.94)*	2.95 (2.39 to 3.51)*
Non-optimal temperature	8,427,319 (7,191,510 to 9,779,073)	12	9,325,364 (7,641,727 to 11,224,758)	11	0.44 (-0.88 to 1.77)	-0.61 (-1.39 to 0.18)	1.14 (-1.01 to 3.34)
Kidney dysfunction	8,884,203 (7,881,193 to 10,012,008)	11	13,353,909 (11,150,932 to 15,771,793)	10	2.25 (1.83 to 2.67)*	3.04 (2.37 to 3.71)*	1.47 (0.91 to 2.03)*
High LDL cholesterol	11,217,788 (9,135,992 to 13,780,576)	10	19,813,962 (15,205,317 to 25,139,359)	7	3.11 (2.85 to 3.36)*	3.86 (3.39 to 4.33)*	2.23 (2.08 to 2.37)*
Childhood sexual abuse and bullying	762,744 (311,431 to 1,443,447)	20	646,857,857 (278,772 to 1,183,126)	20	-0.86 (-0.97 to -0.75)*	-1.17 (-1.29 to -1.04)*	-0.40 (-0.58 to -0.22)*
**Age-standardized rate**							
Unsafe water, sanitation, and handwashing	475 (376 to 587)	14	91 (64 to 122)	18	-8.23 (-8.79 to -7.67)*	-11.69 (-12.52 to -10.85)*	-4.58 (-5.22 to -3.93)*
Air pollution	4,860 (4,243 to 5,438)	2	2,265 (1,950 to 2,615)	4	-3.93 (-4.15 to -3.70)*	-4.08 (-4.49 to -3.68)*	-3.86 (-3.97 to -3.74)*
Other environmental risks	488 (338 to 653)	13	331 (213 to 460)	13	-2.00 (-2.13 to -1.87)*	-1.49 (-1.73 to -1.25)*	-2.64 (-2.71 to -2.56)*
Child and maternal malnutrition	3,285 (2,955 to 3,649)	4	786 (690 to 899)	10	-7.24 (-7.44 to -7.04)*	-8.34 (-8.58 to -8.10)*	-6.13 (-6.41 to -5.85)*
Tobacco	5,287 (4,858 to 5,765)	1	3,228 (2,759 to 3,767)	1	-2.57 (-2.83 to -2.32)*	-2.80 (-3.20 to -2.40)*	-2.33 (-2.68 to -1.98)*
Alcohol use	1,137 (972 to 1,328)	8	908 (729 to 1,116)	8	-1.18 (-1.50 to -0.86)*	-1.41 (-1.54 to -1.28)*	-0.92 (-1.56 to -0.27)*
Drug use	449 (390 to 518)	15	295 (238 to 361)	14	-2.11 (-2.74 to -1.48)*	-4.70 (-4.97 to -4.43)*	0.85 (-0.48 to 2.19)
High fasting plasma glucose	1,647 (1,338 to 2,051)	6	1,452 (1,139 to 1,832)	5	-0.72 (-1.23 to -0.20)*	0.72 (-0.06 to 1.51)	-2.11 (-2.76 to -1.44)*
High systolic blood pressure	3,304 (2,820 to 3,777)	3	2,844 (2,392 to 3,321)	2	-0.77 (-0.89 to -0.65)*	-0.29 (-0.51 to -0.07)*	-1.40 (-1.47 to -1.34)*
High body-mass index	962 (315 to 1,891)	10	1,231 (578 to 2,023)	6	1.28 (0.95 to 1.60)*	1.50 (0.91 to 2.10)*	0.94 (0.82 to 1.07)*

Low bone mineral density	164 (137 to 192)	16	177 (138 to 214)	15	0.40 (0.27 to 0.54)*	0.70 (0.55 to 0.86)*	0.20 (-0.02 to 0.42)
Dietary risks	3,022 (2,391 to 3,875)	5	2,394 (1,823 to 3,071)	3	-1.23 (-1.36 to -1.09)*	-0.78 (-1.03 to -0.52)*	-1.75 (-1.83 to -1.68)*
Low physical activity	149 (74 to 287)	17	146 (70 to 280)	16	-0.19 (-0.74 to 0.36)	0.48 (0.17 to 0.79)*	-0.93 (-2.03 to 0.18)
Occupational risks	1,482 (1,277 to 1,700)	7	844 (701 to 997)	9	-2.95 (-3.08 to -2.81)*	-3.67 (-3.88 to -3.45)*	-2.22 (-2.37 to -2.07)*
Intimate partner violence	55 (30 to 86)	19	46 (22 to 76)	20	-0.86 (-1.11 to -0.62)*	-1.68 (-1.94 to -1.41)*	0.09 (-0.32 to 0.50)
Unsafe sex	108 (94 to 141)	18	142 (106 to 170)	17	1.47 (0.88 to 2.06)*	1.83 (1.43 to 2.23)*	1.34 (0.19 to 2.51)*
Non-optimal temperature	936 (813 to 1,075)	11	483 (399 to 582)	12	-3.61 (-4.04 to -3.18)*	-4.39 (-5.06 to -3.71)*	-2.91 (-3.41 to -2.42)*
Kidney dysfunction	781 (690 to 882)	12	709 (593 to 832)	11	-0.45 (-0.97 to 0.08)	054 (-0.31 to 1.39)	-1.41 (-2.10 to -0.72)*
High LDL cholesterol	1,005 (790 to 1,284)	9	1,052 (800 to 1,345)	7	0.28 (0.10 to 0.45)*	1.19 (0.87 to 1.51)*	-0.75 (-0.85 to -0.66)*
Childhood sexual abuse and bullying	54 (22 to 102)	20	50 (20 to 91)	19	-0.41 (-0.51 to -0.31)*	-1.05 (-1.21 to -0.89)*	0.40 (0.31 to 0.48)*

Note: AAPC: average annual percent changes; UI, uncertainty interval; CI, confidence interval; LDL, low-density lipoprotein.
*AAPC significantly differs from zero at the alpha = 0.05 level.
