# Supplementary material for: Temporal trend of population structure, burden of diseases, healthcare resources and expenditure in China, 2000–2019
Source: BMJ Open. 2023 Jan 18;13(1):e062091. doi: 10.1136/bmjopen-2022-062091 (PMC9853154; doi:10.1136/bmjopen-2022-062091)
Supplement: Author's manuscript [file bmjopen-2022-062091.draft_revisions.pdf]

BMJ Open is committed to open peer review. As part of this commitment we make the peer review history of every article we publish publicly available.

When an article is published we post the peer reviewers' comments and the authors' responses online. We also post the versions of the paper that were used during peer review. These are the versions that the peer review comments apply to.

The versions of the paper that follow are the versions that were submitted during the peer review process. They are not the versions of record or the final published versions. They should not be cited or distributed as the published version of this manuscript.

BMJ Open is an open access journal and the full, final, typeset and author-corrected version of record of the manuscript is available on our site with no access controls, subscription charges or pay-per-view fees (http://bmjopen.bmj.com).

If you have any questions on BMJ Open's open peer review process please email info.bmjopen@bmj.com

BMJ Open

Trends in population, health care, and disease burden in China, 2000–2019

Journal:	*BMJ Open*
Manuscript ID	bmjopen-2022-062091
Article Type:	Original research
Date Submitted by the Author:	19-Feb-2022
Complete List of Authors:	Liang, Zhenguo; Third Affiliated Hospital of Sun Yat-Sen University, Department of Rheumatology<br>WU, Dongze; Third Affiliated Hospital of Sun Yat-Sen University, Department of Rheumatology<br>Guo, Cui; The Chinese University of Hong Kong<br>Gu, Jieruo; Third Affiliated Hospital of Sun Yat-Sen University, Department of Rheumatology
Keywords:	Health economics < HEALTH SERVICES ADMINISTRATION & MANAGEMENT, Health policy < HEALTH SERVICES ADMINISTRATION & MANAGEMENT, Health & safety < HEALTH SERVICES ADMINISTRATION & MANAGEMENT

SCHOLARONE™
Manuscripts

I, the Submitting Author has the right to grant and does grant on behalf of all authors of the Work (as defined in the below author licence), an exclusive licence and/or a non-exclusive licence for contributions from authors who are: i) UK Crown employees; ii) where BMJ has agreed a CC-BY licence shall apply, and/or iii) in accordance with the terms applicable for US Federal Government officers or employees acting as part of their official duties; on a worldwide, perpetual, irrevocable, royalty-free basis to BMJ Publishing Group Ltd ("BMJ") its licensees and where the relevant Journal is co-owned by BMJ to the co-owners of the Journal, to publish the Work in this journal and any other BMJ products and to exploit all rights, as set out in our licence.

The Submitting Author accepts and understands that any supply made under these terms is made by BMJ to the Submitting Author unless you are acting as an employee on behalf of your employer or a postgraduate student of an affiliated institution which is paying any applicable article publishing charge ("APC") for Open Access articles. Where the Submitting Author wishes to make the Work available on an Open Access basis (and intends to pay the relevant APC), the terms of reuse of such Open Access shall be governed by a Creative Commons licence – details of these licences and which Creative Commons licence will apply to this Work are set out in our licence referred to above.

Other than as permitted in any relevant BMJ Author's Self Archiving Policies, I confirm this Work has not been accepted for publication elsewhere, is not being considered for publication elsewhere and does not duplicate material already published. I confirm all authors consent to publication of this Work and authorise the granting of this licence.

For peer review only

Trends in population, health care, and disease burden in China, 2000–2019

Authors

Zhenguo Liang[1#], Dongze Wu, PhD[1#], Cui Guo[2#], PhD, Jieruo Gu, PhD[1*]

Institution

1.Department of Rheumatology, The Third Affiliated Hospital of Sun Yat-sen University, Guangzhou, China

2.Jockey Club School of Public Health and Primary Care, The Chinese University of Hong Kong, Hong Kong SAR, China

[#]Contributed equally to the manuscript.

Corresponding authors

[*]Corresponding to Dr. Dongze Wu and Prof. Jieruo Gu.

Dongze Wu, PhD

Address: No. 600 Tianhe Road, Tianhe District, Guangzhou, Guangdong, China, 510000

E-mail: dongze_wu@163.com

Telephone: (+86) 2085253333 / Fax number: (+86) 2085253336

Prof. Jieguo Gu, PhD

Address: No. 600 Tianhe Road, Tianhe District, Guangzhou, Guangdong, China, 510000

E-mail: gujieruo@mail.sysu.edu.cn

Telephone: (+86) 2085253333 / Fax number: (+86) 2085253336

Running Head: Population ageing in China

Keywords: Trend, Population, Heath Care, Disease Burden, China

Word Count: 3233 Reference:38 Table Count: 4 Figure Count:1

For peer review only - http://bmjopen.bmj.com/site/about/guidelines.xhtml

Brief summary

The changes and disease burden of China's population from 2000 to 2019 were analyzed to provide reference value for better facing population aging in China.

Ethical statement

Data released from the Global Health Data Exchange query and the China Statistical Yearbook did not require informed patient consent. This study used an anonymized publicly available data set with no identifiable information of the survey participants. Thus, ethics approval was not required for this study.

Acknowledgments

We thank the staff at the Institute for Health Metrics and Evaluation and its collaborators who prepared publicly available and impactful data. We also thank the editorial board and staff of the National Bureau of Statistics of China who compiled the comprehensive statistical data used.

For peer review only

For peer review only - http://bmjopen.bmj.com/site/about/guidelines.xhtml

Abstract

Objectives

To explore the trend of disease burden evolution from young to old population in China, and to provide guidance and suggestions for areas requiring intervention.

Methods

Temporal trends regarding population and heath care were generated from the China Statistical Yearbook. Temporal trends of disease burden were generated from a Global Burden of Diseases Study that was conducted among individuals younger and older than 70 years between 2000 and 2019. Average annual percent change (AAPC) with 95% confidence interval (CI) was quantified by using the Joinpoint Regression Program.

Results

Regarding the population in China between 2000 and 2019, a decreasing trend was observed among youth aged 0–14 years (AAPC: -1.17), a slow rising trend was observed among individuals aged 15–64 years (AAPC: 1.10), and a rapidly increasing trend was observed among individuals older than 65 years (AAPC: 3.67). Astonishing increasing trends in healthcare institutions (AAPC: 3.97), medical personnel (AAPC: 3.26), and healthcare expenditures (AAPC: 15.28) were also observed. Among individuals younger than 70 years, neoplasms (AAPC: 0.54) and cardiovascular diseases (AAPC: 0.67) remained among the top three causes, while tobacco (AAPC: 0.22) remained a top three risk factor. However, while musculoskeletal disorders (AAPC: 1.88) were not a top three cause in 2000, they are a top three cause in 2019.

Conclusion

Comprehensive regional-specific strategies that target younger populations are urgently

For peer review only - http://bmjopen.bmj.com/site/about/guidelines.xhtml

needed to mitigate the causes and risk factors which represent changes in population dynamics and to reduce disease burden.

Keywords: Trend, Population, Heath Care, Disease Burden, China

KEY QUESTIONS:

What is already known on this topic

The disease risk factors of the elderly population are cancer, cardiovascular diseases, tobacco and diet risks, etc., which have always been the disease burden worthy of attention.

What this study adds

From 2000 to 2019, the birth rate decreased, and the young population decreased, but the aging was aggravated, and the elderly population increased significantly.

How this study might affect research, practice or policy

This study has identified the key areas that need intervention, and established a comprehensive strategy for the young population to reduce the causes and risk factors of population change. By formulating corresponding strategies, the increasing disease burden of people over 70 years old and close to this age group will be reduced.

For peer review only

Introduction

Together with the world's population, people in China struggle against cancer and aging despite efforts to extend lifespan and maximize healthspan[1, 2]. Since 1950, the lifespan at birth has been extended by 26 years in China, while the total fertility rate has decreased from 5.91 to 1.43[3]. Between 2000 and 2019, the life expectancy for males has increased from 69.1 to 74.4 years, while the life expectancy for females has increased from 73.9 to 80.8 years[3]. It has been forecasted that China will become the largest economy by 2035, and that the population in China will decline by 48.0% from 2017 to 2100[4]. Moreover, in the next 20 years, the universal two-to-three-child policy may not reverse a shrinking workforce and rapid population ageing, especially in rural areas[5, 6]. In 2009, China expanded social health insurance coverage to its entire population and reformed its health care delivery system[7, 8].

Health care systems coordinate complex interrelationships among individuals who receive and provide medical financial support[9]. It would have been difficult for China to rigorously control the two pandemics that occurred in 2003 and 2019 without a healthcare system. In particular, an increasing number of modernized medical institutions and well-educated personnel have been established over the past two decades in China[10, 11]. Accelerating rural-to-urban migration and corresponding increases in urban populations have also both led to improved access to health care, although substantial health risks can accompany changes in human activities, diets, and social structures[12]. Therefore, the objective of this study was to quantify trends in population, health care, and disease burden in China between 2000 and 2019. Possible driving forces of disease burden among ageing populations were also examined.

For peer review only - http://bmjopen.bmj.com/site/about/guidelines.xhtml

Methods

China Statistical Yearbook (CSY)

The National Bureau of Statistics (NBS) of China annually publishes the CSY[13]. The Department of Population and Employment Statistics of the NBS conducts statistical population surveys as follows. A national population census is conducted in years ending with 0; a national 1 percent population sample survey is conducted in years ending with 5; and sample surveys on population changes are conducted in the remaining years. The latter covers about 1 per thousand of the total population of China. The sample surveys on population change take the entire nation of China as the population, while each province, autonomous region, or municipality represents a sub-population. A stratified multi-stage systematic probability-proportional-to-size (PPS) cluster sampling scheme is used. Data regarding population, healthcare institutions, personnel, beds, and health expenditures are described in detail in the Supplemental Appendix (including a glossary and definitions).

Global Burden of Disease (GBD)

In 2019, the GBD Study assessed life expectancy, disease burden, risk factor, etiology, and impairment[3, 14, 15]. All-cause incidence, prevalence, death, years lived with disability (YLD), years of life lost (YLL), disability-adjusted life years (DALYs), and attributable risk factors were assessed between 1990 and 2019. The study identified twenty-two level 2 leading causes and twenty level 2 leading risk factors. Both causes and risk factors were assessed according to number, rate, and age-standardized rate of DALYs. The estimation methods and data for burden of disease published in GBD 2019 were used to assess disease burden in China between 2000 and 2019 for the present study.

Statistical analysis

For peer review only - http://bmjopen.bmj.com/site/about/guidelines.xhtml

Temporal trends in population, heath care, and disease burden were assessed by using a Joinpoint regression model. Average annual percent change (AAPC) and associated 95% confidence intervals (CIs) were calculated for the study period. Temporal increasing and decreasing trends were defined according to the statistical significance of the AAPC compared to zero. AAPC values with 95% CIs overlapping with zero were considered stable. All statistical analyses were performed by using the Joinpoint Regression Program (version 4.8.0.1, Statistical Methodology and Applications Branch, Surveillance Research Program, National Cancer Institute, Bethesda, MD, USA). P-values less than 0.05 were considered statistically significant at a two-tailed level.

Patient and public involvement

Patients and the public were not involved in any way in this research.

Results

Population trends between 2000 and 2019

Initially, temporal trends of the population structure in China were examined based on CSY data (gender and age composition, birth rate, and dependency ratio). From 2000 through 2019, an increasing trend in total population (AAPC: 0.52, 95% CI: 0.51 to 0.54), male population (AAPC: 0.47, 95% CI: 0.44 to 0.51), and female population (AAPC: 0.59, 95% CI: 0.56 to 0.62) were observed. As expected, a striking contrary trend in urban population (AAPC: 3.28, 95%CI: 3.18 to 3.38) and rural population (AAPC: -1.98, 95%CI: -2.02 to -1.95) were observed. We further identified that an increasing trend in urban population was attenuated, while a decreasing trend in rural population was aggravated, during 2010–2019 compared to 2000–2009 (Figure 1A, Table 1). A clear decreasing trend in the younger population (ages 0–14 years) was observed in 2000–2019 (AAPC: -1.17, 95%CI: -1.78 to -0.55), especially in 2000–2009. An increasing trend in the

For peer review only - http://bmjopen.bmj.com/site/about/guidelines.xhtml

Table 1. AAPC in population composition, change, and dependency ratio between 2000 and 2019

Subcategory	Number			AAPC (95% CI)		
	2000	2009	2019	2000–2019	2000–2009	2010–2019
**Population composition by gender and area (10000 persons)**						
Total Population	126,743	133,450	140,005	0.52 (0.51 to 0.54)*	0.57 (0.56 to 0.58)*	0.48 (0.45 to 0.51)*
Male Population	65,437	68,647	71,527	0.47 (0.44 to 0.51)*	0.53 (0.50 to 0.57)*	0.43 (0.37 to 0.49)*
Female Population	61,306	64,803	68,478	0.59 (0.56 to 0.62)*	0.62 (0.56 to 0.67)*	0.56 (0.55 to 0.57)*
Urban Population	45,906	64,512	84,843	3.28 (3.18 to 3.38)*	3.86 (3.75 to 3.98)*	2.68 (2.51 to 2.84)*
Rural Population	80,837	68,938	55,162	-1.98 (-2.02 to -1.95)*	-1.79 (-1.86 to -1.73)*	-2.15 (-2.18 to -2.12)*
**Population composition by age group (10000 persons)**						
Population Aged 0–14 y	29,012	24,659	23,492	-1.17 (-1.78 to -0.55)*	-2.24 (-2.76 to -1.71)*	0.31 (-0.23 to 0.85)
Population Aged 15–64 y	88,910	97,484	98,910	0.59 (0.52 to 0.66)*	1.10 (1.03 to 1.18)*	0.02 (-0.10 to 0.14)
Population Aged 65+ y	8,821	11,307	17,603	3.67 (3.51 to 3.82)*	2.81 (2.66 to 2.96)*	4.51 (4.25 to 4.76)*
**Population change (%)**						
Birth Rate	14.03	11.95	10.48	-1.53 (-2.31 to -0.75)*	-1.63 (-2.32 to -0.93)*	-1.57 (-3.03 to -0.09)*
Death Rate	6.45	7.08	7.14	0.54 (0.37 to 0.71)*	1.10 (0.75 to 1.46)*	0.03 (-0.06 to 0.13)
Natural Growth Rate	7.58	4.87	3.34	-4.20 (-5.52 to -2.87)*	-4.64 (-5.69 to -3.57)*	-4.40 (-6.93 to -1.80)*
**Dependency ratio (%)**						
Gross Dependency Ratio	42.6	36.9	41.5	-0.25 (-0.54 to 0.03)	-1.97 (-2.26 to -1.68)*	1.70 (1.21 to 2.19)*
Children Dependency Ratio	32.6	25.3	23.8	-1.76 (-2.54 to -0.96)*	-3.33 (-4.00 to -2.66)*	0.33 (-0.37 to 1.03)
Old Dependency Ratio	9.9	11.6	17.8	3.12 (2.93 to 3.31)*	1.76 (1.45 to 2.08)*	4.49 (4.27 to 4.70)*

AAPC: average annual percent change; CI, confidence interval. *AAPC significantly differs from zero at a two-tailed level of 0.05.

For peer review only - http://bmjopen.bmj.com/site/about/guidelines.xhtml

working population (ages 15–64 years) was observed in 2000–2009 (AAPC: 1.10, 95%CI: 1.03 to 1.18), and then it gradually disappeared over 2010–2019. In contrast, an increasing trend in the post-working population (65+ years) that was observed in 2000–2019 (AAPC: 3.67, 95%CI: 3.51 to 3.82) gradually intensified from 2000–2009 to 2010–2019 (Figure 1B, Table 1).

From 2000 to 2019, the birth rate (AAPC: -1.53, 95%CI: -2.31 to -0.75) and natural growth rate (AAPC: -4.20, 95%CI: -5.52 to -2.87) continued to decrease. Conversely, from 2000 to 2009, the death rate continued to increase (AAPC: 1.10, 95%CI: 0.75 to 1.46), yet from 2010–2019 it remained stable (AAPC: 0.03, 95%CI: -0.06 to 0.13) (Figure 1C, Table 1). The past two decades also exhibited a reverse trend in gross dependency ratio from 2010–2019, a muted decreasing trend in children dependency ratio from 2000–2009 to 2010–2019, and a strengthened increasing trend in old dependency ratio from 2000–2009 to 2010–2019 (Figure 1D, Table 1).

Healthcare trends between 2000 and 2019

Temporal trends in health care, including institutions, personnel, and expenditures, were examined based on CSY data. A consistent increasing trend in number of hospitals (AAPC: 3.97, 95%CI: 3.69 to 4.24), including general hospitals, hospitals that specialize in traditional Chinese medicine, and specialized hospitals, was observed. The number of medical personnel (AAPC: 3.26, 95%CI: 2.76 to 3.77), including medical technical personnel, licensed assistant doctors, licensed doctors, and registered nurses, also continued to increase in 2000–2019. Moreover, the rate of increase of number of medical personnel was higher in 2010–2019 than in 2000–2009. The number of beds in health care institutions (AAPC: 5.54, 95%CI: 5.20 to 5.88), including hospitals, township health service centers, specialized public health institutions, and women and children care agencies, also increased in 2000–2019. Similar to medical personnel, the rate increase for beds in health care institutions was greater in 2010–2019 than in 2000–2009 (Table 2).

For peer review only - http://bmjopen.bmj.com/site/about/guidelines.xhtml

Table 2. AAPC in healthcare institution, personnel, and expenditures between 2000 and 2019

Subcategory	Number			AAPC (95% CI)		
	2000	2009	2019	2000—2019	2000—2009	2010—2019
**Health care institution (unit)**						
Number of Hospitals	16,318	20,291	34,354	3.97 (3.69 to 4.24)*	2.41 (2.03 to 2.80)*	5.72 (5.25 to 6.19)*
Number of General Hospitals	11,872	13,364	19,963	2.84 (2.56 to 3.13)*	1.37 (0.97 to 1.77)*	4.51 (4.03 to 4.99)*
Number of Hospitals Specialized in Traditional Chinese Medicine	2,453	2,728	4,221	2.92 (2.50 to 3.34)*	1.35 (1.20 to 1.50)*	4.69 (3.82 to 5.58)*
Number of Specialized Hospitals	1,543	3,716	8,531	9.65 (9.02 to 10.28)*	10.98 (9.69 to 12.28)*	8.46 (8.25 to 8.68)*
Number of Health Care Institutions at Grass-root Level	1,000,169	882,153	954,390	-0.45 (-0.82 to -0.08)*	-1.88 (-2.60 to -1.15)*	0.85 (0.67 to 1.04)*
Number of Township Health Service Centers	49,229	38,475	36,112	-1.63 (-1.83 to -1.44)*	-2.79 (-3.10 to -2.47)*	-0.47 (-0.66 to -0.28)*
Number of Village Clinics	709,458	632,770	616,094	-1.03 (-1.40 to -0.65)*	-1.88 (-2.53 to -1.23)*	-0.51 (-0.86 to -0.17)*
Number of Outpatient Department	240,934	182,448	266,659	0.42 (-0.18 to 1.03)	-2.73 (-3.45 to -2.01)*	4.05 (3.03 to 5.07)*
Number of Specialized Public Health Institutions	11,386	11,665	15,958	1.63 (-0.23 to 3.54)	0.12 (-0.68 to 0.93)	3.35 (-0.52 to 7.36)
Number of Center for Disease Control and Prevention	3,741	3,536	3,403	-0.40 (-0.50 to -0.29)*	-0.40 (-0.50 to -0.29)*	-0.40 (-0.50 to -0.29)*
Number of Specialized Disease Prevention & Treatment Institution	1,839	1,291	1,128	-2.47 (-2.97 to -1.98)*	-3.61 (-4.46 to -2.76)*	-1.49 (-2.05 to -0.93)*
Number of Women and Children Care Agencies	3,163	3,020	3,071	-0.11 (-0.29 to 0.08)	-0.45 (-0.80 to -0.10)*	0.20 (0.09 to 0.32)*
**Medical Personnel (10000 persons)**						
Number of Medical Personnel	691.04	778.14	1292.83	3.26 (2.76 to 3.77)*	1.15 (0.16 to 2.14)*	5.16 (4.87 to 5.45)*
Number of Medical Technical Personnel	449.08	553.51	1015.40	4.34 (3.99 to 4.68)*	2.30 (1.61 to 3.01)*	6.20 (6.07 to 6.34)*
Number of Licensed (Assistant) Doctors	207.58	232.92	386.69	3.12 (2.64 to 3.59)*	0.92 (0.05 to 1.81)*	5.36 (4.98 to 5.74)*
Number of Licensed Doctors	160.33	190.54	321.05	3.53 (3.12 to 3.95)*	1.50 (0.73 to 2.27)*	5.62 (5.29 to 5.95)*
Number of Registered Nurse	126.68	185.48	444.50	6.79 (6.20 to 7.38)*	4.37 (3.26 to 5.49)*	8.92 (8.49 to 9.34)*
Number of Pharmacist	41.44	34.19	48.34	0.76 (0.23 to 1.29)*	-2.28 (3.31 to -1.23)*	3.57 (3.27 to 3.88)*
Number of Village Doctors and Assistants	131.94	105.10	84.23	-2.49 (-4.28 to -0.66)*	-3.44 (-6.25 to -0.55)*	-2.16 (-4.10 to -0.17)*
Number of Other Technical Personnel	15.75	27.50	50.39	6.30 (6.07 to 6.53)*	6.30 (6.07 to 6.53)*	6.30 (6.07 to 6.53)*
Number of Administrative Personnel	42.68	36.27	54.38	1.25 (0.42 to 2.09)*	-1.74 (-3.27 to -0.18)*	4.01 (3.49 to 4.54)*
Number of Logistics Technical Workers	51.59	55.77	88.43	2.95 (2.13 to 3.78)*	0.84 (-0.26 to 1.94)	4.71 (3.57 to 5.86)*
**Beds in Health Care Institutions**						
Number of Beds in Health Care Institutions (10000 units)	317.70	441.66	880.70	5.54 (5.20 to 5.88)*	3.60 (3.01 to 4.20)*	7.11 (6.82 to 7.41)*
Number of Beds in Hospitals (10000 beds)	216.67	312.08	686.65	6.35 (5.96 to 6.74)*	4.17 (3.55 to 4.81)*	8.16 (7.74 to 8.59)*
Number of Beds in Community Health Service Centers (10000 units)	76.65	109.98	163.11	3.96 (2.90 to 5.04)*	3.73 (1.68 to 5.83)*	3.28 (2.75 to 3.82)*
Number of Beds in Township Health Service Centers (10000 beds)	73.48	93.34	136.99	3.29 (2.18 to 4.41)*	2.38 (0.26 to 4.54)*	3.48 (2.93 to 4.04)*

For peer review only - http://bmjopen.bmj.com/site/about/guidelines.xhtml

For peer review only

Number of Beds in Specialized Public Health Institutions (10000 units)	11.86	15.40	28.50	4.80 (4.31 to 5.28)*	2.97 (2.52 to 3.42)*	6.24 (5.55 to 6.93)*
Number of Beds in Women and Children Care Agencies (10000 units)	7.12	12.61	24.32	6.79 (6.41 to 7.18)*	6.65 (6.15 to 7.15)*	6.74 (6.25 to 7.24)*
Number of Beds in Specialized Disease Prevention & Treatment Institution (10000 beds)	2.84	2.71	4.11	2.29 (0.21 to 4.41)*	0.19 (-3.08 to 3.57)	3.75 (1.61 to 5.95)*
**Health Expenditure (100 million yuan)**						
Total Health Expenditure	4586.63	17541.92	65841.39	15.28 (14.64 to 15.91)*	15.99 (15.09 to 16.90)*	14.04 (13.44 to 14.63)*
Government Health Expenditure	709.52	4816.26	18016.95	18.95 (16.72 to 21.22)*	24.39 (20.91 to 27.97)*	13.52 (11.30 to 15.79)*
Social Health Expenditure	1171.94	6154.49	29150.57	19.03 (17.56 to 20.52)*	21.13 (18.16 to 24.18)*	17.17 (16.18 to 18.16)*
Out-of-pocket Health Expenditure	2705.17	6571.16	18673.87	10.62 (9.24 to 12.02)*	9.82 (9.21 to 10.44)*	10.96 (8.48 to 13.48)*
Per Capita Health Expenditure (yuan)	361.88	1314.26	4702.79	14.67 (14.04 to 15.30)*	15.34 (14.45 to 16.24)*	13.47 (12.89 to 14.06)*

AAPC: average annual percent change; CI, confidence interval. *AAPC significantly differs from zero at a two-tailed level of 0.05.

For peer review only - http://bmjopen.bmj.com/site/about/guidelines.xhtml

Total healthcare expenditure increased from ¥458.66 billion (US$ 55.40 billion) in 2000 to ¥6584.14 billion (US$ 954.68 billion) in 2019, with an AAPC of 15.28 (95%CI: 14.64 to 15.91). The per capita health expenditure increased from ¥361.88 in 2000 to ¥4702.79 in 2019, with an AAPC of 14.67 (95%CI: 14.04 to 15.30). Striking increasing trends in government health expenditure (AAPC: 18.95, 95%CI: 16.72 to 21.22), social health expenditure (AAPC: 19.03, 95%CI: 17.56 to 20.52), and out-of-pocket health expenditure (AAPC: 10.62, 95%CI: 9.24 to 12.02) were also observed in 2000–2019 (Table 2).

Trends in causes and risk factors between 2000 and 2019

According to data from GBD 2019, life expectancy at birth and at 60–64 years were estimated to increase (AAPC: 0.44, 95%CI: 0.43 to 0.46; AAPC: 0.76, 95%CI: 0.60 to 0.91, respectively). In addition, the percentage of healthy life expectancy within total life expectancy at birth and at 60–64 years decreased from 88.90 to 88.32, and from 77.12 to 76.36, respectively (sFigure 1, sTable 1–3). Overall, the number of all-cause DALYs was stable during 2000–2019 (AAPC: 0.08, 95%CI -0.06 to 0.22). However, it decreased between 2000 and 2009 (AAPC: -0.44, 95%CI: -0.58 to -0.31), and it increased between 2010 and 2019 (AAPC: 0.60, 95% CI: 0.39 to 0.82) (sTable 4).

In 2000, the top three causes of DALYs were cardiovascular diseases, neoplasms, and chronic respiratory diseases. In 2019, they were cardiovascular diseases, neoplasms, and musculoskeletal disorders. From 2000–2019, the top three drivers of increasing number of DALYs were HIV/AIDS and sexually transmitted infections (AAPC: 3.47, 95%CI: 2.72 to 4.22), diabetes and kidney diseases (AAPC: 2.30, 95%CI: 2.09 to 2.51), and sense organ diseases (AAPC: 2.14, 95%CI: 1.98 to 2.30). The top two drivers of increasing age-standardized rate of DALYs were HIV/AIDS and sexually transmitted infections (AAPC: 2.02, 95%CI: 1.50 to 2.53) and musculoskeletal disorders

For peer review only - http://bmjopen.bmj.com/site/about/guidelines.xhtml

(AAPC: 0.16, 95%CI: 0.10 to 0.23) (sTable 5).

In 2000, the top three risk factors were tobacco, air pollution, and high systolic blood pressure. In 2019, they were tobacco, high systolic blood pressure, and dietary risks. Between 2000 and 2019, the top three leading risk factors with the largest increasing number of DALYs were high body-mass index (AAPC: 4.02, 95%CI: 3.74 to 4.29), low physical activity (AAPC: 3.54, 95%CI: 2.70 to 4.38), and low bone mineral density (AAPC: 3.39, 95%CI: 3.12 to 3.67). The top three leading factors with the largest increase in age-standardized rate of DALYs were unsafe sex (AAPC: 1.47, 95% CI: 0.88 to 2.06), high body-mass index (AAPC: 1.28, 95%CI: 0.95 to 1.60), and low bone mineral density (AAPC: 0.40, 95%CI: 0.27 to 0.54) (sTable 6).

Trends in disease burden between younger individuals and those older than 70 years

Finally, trends in causes and risk factors for the population with a cut-off point of 70 years were examined for 2000–2019. Among individuals younger than 70 years, musculoskeletal disorders replaced other non-communicable diseases as one of the top three causes from 2000 to 2019. In contrast, the top three causes remained the same among individuals older than 70 years, and included cardiovascular diseases, neoplasms, and chronic respiratory disease (Table 3). Among the population younger than 70 years, the causes with the largest increment in number of DALYs were HIV/AIDS and sexually transmitted infections (AAPC: 3.33, 95%CI: 2.58 to 4.08), musculoskeletal disorders (AAPC: 1.88, 95%CI: 1.84 to 1.92), and sense organ diseases (AAPC: 1.71, 95%CI: 1.43 to 2.00). In the same population, the largest increments in rate of DALYs were HIV/AIDS and sexually transmitted infections (AAPC: 3.42, 95%CI: 2.76 to 4.09), musculoskeletal disorders (AAPC: 2.04, 95%CI: 2.00 to 2.08), and diabetes and kidney diseases (AAPC: 1.88, 95%CI: 1.58 to 2.18). In comparison, the causes among individuals older than 70 years that had the largest increment in both number and rate of DALYs were maternal and neonatal

For peer review only - http://bmjopen.bmj.com/site/about/guidelines.xhtml

Table 3. The AAPC of 22 causes in people younger and older than 70 years of age presented in DALYs (95% UI)

Causes	Age < 70 years			Age ≥ 70 years		
	2000	2019	AAPC (95% CI)	2000	2019	AAPC (95% CI)
**Number**						
Neglected tropical diseases and malaria	1,566,614 (1,022,581 to 2,343,199)	977,312 (586,629 to 1,582,219)	-2.42 (-2.73 to -2.10)*	137,451 (93,607 to 195,272)	160,494 (103,985 to 236,535)	0.83 (0.72 to 0.94)*
Nutritional deficiencies	3,912,001 (2,872,239 to 5,269,234)	2,247,485 (1,515,113 to 3,202,132)	-2.91 (-3.07 to -2.76)*	271,587 (210,115 to 352,577)	359,440 (285,475 to 450,281)	1.51 (1.21 to 1.81)*
Neoplasms	43,633,030 (40,123,940 to 47,258,486)	48,503,754 (40,915,702 to 56,689,779)	0.54 (0.36 to 0.73)*	10,782,364 (10,021,201 to 11,549,415)	19,016,076 (16,369,289 to 21,712,270)	3.00 (2.66 to 3.34)*
Cardiovascular diseases	40,746,813 (37,734,622 to 44,374,198)	45,823,157 (39,092,553 to 53,248,142)	0.67 (0.52 to 0.82)*	26,837,679 (24,915,174 to 29,373,891)	46,109,965 (40,669,589 to 51,698,469)	2.77 (2.26 to 3.29)*
Chronic respiratory diseases	13,237,789 (11,103,026 to 14,475,351)	8,969,697 (7,748,010 to 10,534,163)	-2.04 (-2.22 to -1.86)*	15,416,177 (12,665,862 to 16,567,447)	13,550,852 (11,816,464 to 16,036,186)	-0.70 (-1.15 to -0.24)*
Digestive diseases	9,775,769 (8,808,397 to 10,835,090)	7,710,658 (6,445,828 to 8,964,870)	-1.21 (-1.30 to -1.13)*	1,923,302 (1,762,799 to 2,106,969)	2,307,223 (2,029,697 to 2,600,797)	0.92 (0.72 to 1.13)*
Neurological disorders	9,677,839 (4,755,522 to 16,786,166)	11,207,269 (5,201,198 to 19,881,074)	0.78 (0.70 to 0.86)*	2,828,463 (1,694,636 to 5,326,904)	6,174,136 (3,629,888 to 11,661,806)	4.20 (4.04 to 4.35)*
Mental disorders	17,241,666 (12,746,572 to 22,736,338)	18,539,346 (13,738,299 to 24,152,654)	0.37 (0.29 to 0.46)*	868,363 (645,857 to 1,119,766)	1,754,481 (1,291,689 to 2,254,704)	3.77 (3.70 to 3.83)*
Musculoskeletal disorders	17,725,280 (12,654,675 to 23,783,378)	25,286,535 (18,045,649 to 33,942,087)	1.88 (1.84 to 1.92)*	2,364,272 (1,679,407 to 3,221,901)	4,647,389 (3,292,799 to 6,332,328)	3.60 (3.53 to 3.66)*
Other non-communicable diseases	23,430,651 (19,446,807 to 28,355,357)	16,912,189 (12,820,141 to 22,584,969)	-1.67 (-1.77 to -1.58)*	1,142,340 (831,796 to 1,540,224)	2,213,884 (1,599,002 to 2,992,458)	3.43 (2.88 to 3.98)*
Skin and subcutaneous diseases	7,427,502 (4,833,763 to 10,987,503)	7,564,844 (4,920,805 to 11,324,882)	0.09 (0.07 to 0.12)*	381,523 (263,385 to 542,001)	699,858 (475,558 to 1,030,747)	3.21 (3.14 to 3.28)*
Sense organ diseases	7,881,599 (5,268,499 to 11,424,474)	10,796,982 (7,099,138 to 15,924,830)	1.71 (1.43 to 2.00)*	2,859,183 (2,030,582 to 3,892,635)	5,184,684 (3,650,080 to 7,090,135)	3.18 (3.02 to 3.34)*
Transport injuries	16,117,112 (14,766,378 to 18,356,266)	12,597,412 (10,747,152 to 14,456,584)	-1.31 (-1.64 to -0.98)*	514,733 (454,228 to 601,574)	1,148,043 (946,296 to 1,360,515)	4.24 (3.92 to 4.57)*
Unintentional injuries	19,929,305 (18,307,020 to 21,760,124)	13,173,922 (10,853,663 to 15,202,200)	-2.15 (-2.65 to -1.65)*	1,184,747 (1,024,676 to 1,371,585)	2,829,540 (2,151,923 to 3,469,812)	4.66 (3.90 to 5.43)*
Self-harm and interpersonal violence	10,574,276 (9,275,669 to 11,544,266)	5,232,715 (4,479,867 to 6,200,044)	-3.64 (-3.94 to -3.34)*	597,000 (504,957 to 649,633)	637,335 (544,995 to 738,168)	0.37 (-0.73 to 1.50)
HIV/AIDS and sexually transmitted infections	904,578 (711,182 to 1,201,186)	1,677,944 (1,372,709 to 2,020,540)	3.33 (2.58 to 4.08)*	16,867 (13,900 to 22,250)	74,476 (55,158 to 94,754)	8.17 (6.93 to 9.43)*
Respiratory infections and tuberculosis	19,691,768 (18,048,674 to 21,296,455)	4,601,349 (3,952,381 to 5,376,466)	-7.32 (-7.79 to -6.85)*	2,246,454 (2,045,162 to 2,412,569)	2,046,523 (1,783,057 to 2,379,652)	-0.54 (-0.96 to -0.13)*
Enteric infections	3,259,938 (2,859,853 to 3,751,832)	1,179,711 (874,430 to 1,534,794)	-5.11 (-5.55 to -4.66)*	147,328 (108,525 to 198,192)	228,387 (164,251 to 306,599)	2.47 (2.25 to 2.69)*
Other infectious diseases	4,467,414 (3,610,475 to 6,110,768)	1,143,025 (971,599 to 1,357,011)	-6.85 (-7.29 to -6.42)*	114,576 (103,794 to 126,392)	101,074 (86,237 to 116,157)	-0.59 (-0.98 to -0.21)*
Maternal and neonatal disorders	18,100,148 (16,534,223 to 19,650,436)	7,080,589 (6,220,729 to 8,094,637)	-4.86 (-5.45 to -4.27)*	10,442 (7,450 to 14,363)	70,983 (51,665 to 96,401)	10.64 (10.47 to 10.82)*
Substance use disorders	6,144,467 (4,909,131 to 7,567,428)	5,459,715 (4,162,774 to 6,975,972)	-0.55 (-0.97 to -0.13)*	147,447 (122,722 to 177,177)	295,943 (226,537 to 375,653)	3.80 (3.23 to 4.37)*
Diabetes and kidney diseases	8,018,292 (6,937,163 to 9,207,068)	10,979,432 (9,048,531 to 13,121,983)	1.70 (1.42 to 1.98)*	2,418,531 (2,116,442 to 2,752,813)	4,929,741 (4,274,671 to 5,652,658)	3.78 (3.60 to 3.96)*
**Rate**						
Neglected tropical diseases and malaria	33 (21 to 49)	21 (13 to 34)	-2.26 (-2.56 to -1.96)*	250 (170 to 355)	149 (96 to 219)	-2.70 (-2.80 to -2.59)*
Nutritional deficiencies	82 (60 to 110)	48 (33 to 69)	-2.77 (-2.96 to -2.58)*	494 (383 to 642)	333 (264 to 417)	-2.03 (-2.30 to -1.76)*
Neoplasms	911 (838 to 987)	1,045 (881 to 1,221)	0.68 (0.59 to 0.77)*	19,629 (18,243 to 21,025)	17,613 (15,162 to 20,110)	-0.56 (-0.93 to -0.19)*
Cardiovascular diseases	851 (788 to 927)	987 (842 to 1,147)	0.84 (0.68 to 1.00)*	48,857 (45,357 to 53,474)	42,708 (37,669 to 47,885)	-0.69 (-0.82 to -0.57)*
Chronic respiratory diseases	276 (232 to 302)	193 (167 to 227)	-1.86 (-2.02 to -1.69)*	28,064 (23,058 to 30,160)	12,551 (10,945 to 14,853)	-4.13 (-4.60 to -3.66)*

For peer review only - http://bmjopen.bmj.com/site/about/guidelines.xhtml

Digestive diseases	204 (184 to 226)	166 (139 to 193)	-1.06 (-1.15 to -0.96)*	3,501 (3,209 to 3,836)	2,137 (1,880 to 2,409)	-2.55 (-2.66 to -2.45)*
Neurological disorders	202 (99 to 351)	241 (112 to 428)	0.94 (0.82 to 1.06)*	5,149 (3,085 to 9,697)	5,719 (3,362 to 10,801)	0.59 (0.29 to 0.90)*
Mental disorders	360 (266 to 475)	399 (296 to 520)	0.55 (0.51 to 0.59)*	1,581 (1,176 to 2,038)	1,625 (1,196 to 2,088)	0.15 (0.12 to 0.18)*
Musculoskeletal disorders	370 (264 to 497)	545 (389 to 731)	2.04 (2.00 to 2.08)*	4,304 (3,057 to 5,865)	4,305 (3,050 to 5,865)	-0.04 (-0.15 to 0.08)
Other non-communicable diseases	489 (406 to 592)	364 (276 to 486)	-1.54 (-1.66 to -1.42)*	2,080 (1,514 to 2,804)	2,051 (1,481 to 2,772)	-0.16 (-0.60 to 0.29)
Skin and subcutaneous diseases	155 (101 to 229)	163 (106 to 244)	0.26 (0.23 to 0.29)*	695 (479 to 987)	648 (440 to 955)	-0.38 (-0.39 to -0.36)*
Sense organ diseases	165 (110 to 239)	233 (153 to 343)	1.86 (1.70 to 2.02)*	5,205 (3,697 to 7,086)	4,802 (3,381 to 6,567)	-0.43 (-0.63 to -0.23)*
Transport injuries	337 (308 to 383)	271 (231 to 311)	-1.15 (-1.49 to -0.81)*	937 (827 to 1,095)	1,063 (876 to 1,260)	0.64 (0.33 to 0.96)*
Unintentional injuries	416 (382 to 454)	284 (234 to 327)	-1.93 (-2.44 to -1.42)*	2,157 (1,865 to 2,497)	2,621 (1,993 to 3,214)	1.01 (0.46 to 1.56)*
Self-harm and interpersonal violence	221 (194 to 241)	113 (96 to 134)	-3.49 (-3.80 to -3.18)*	1,087 (919 to 1,183)	590 (505 to 684)	-3.10 (-4.05 to -2.14)*
HIV/AIDS and sexually transmitted infections	19 (15 to 25)	36 (30 to 44)	3.42 (2.76 to 4.09)*	31 (25 to 41)	69 (51 to 88)	4.39 (3.26 to 3.53)*
Respiratory infections and tuberculosis	411 (377 to 445)	99 (85 to 116)	-7.17 (-7.61 to -6.71)*	4,090 (3,723 to 4,392)	1,896 (1,652 to 2,204)	-4.08 (-4.44 to -3.71)*
Enteric infections	68 (60 to 78)	25 (19 to 33)	-4.91 (-5.76 to -4.05)*	268 (198 to 361)	212 (152 to 284)	-1.20 (-1.45 to -0.96)*
Other infectious diseases	93 (75 to 128)	25 (21 to 29)	-6.70 (-7.14 to -6.27)*	209 (189 to 230)	94 (80 to 108)	-4.06 (-4.48 to -3.64)*
Maternal and neonatal disorders	378 (345 to 410)	153 (134 to 174)	-4.71 (-5.29 to -4.14)*	19 (14 to 26)	66 (48 to 89)	6.84 (6.69 to 6.98)*
Substance use disorders	128 (103 to 158)	118 (90 to 150)	-0.38 (-0.85 to 0.09)*	268 (223 to 323)	274 (210 to 348)	0.12 (-0.31 to 0.55)
Diabetes and kidney diseases	167 (145 to 192)	236 (195 to 283)	1.88 (1.58 to 2.18)*	4,403 (3,853 to 5,011)	4,566 (3,959 to 5,236)	0.21 (-0.05 to 0.48)

AAPC: average annual percent change; DALYs, disability adjusted life years; UI, uncertainty interval, CI, confidence interval.
*AAPC significantly differs from zero at a two-tailed level of 0.05.

For peer review only

For peer review only - http://bmjopen.bmj.com/site/about/guidelines.xhtml

disorders (AAPC: 10.64, 95%CI: 10.47 to 10.82; AAPC: 6.84, 95%CI: 6.69 to 6.98, respectively), HIV/AIDS and sexually transmitted infections (AAPC: 8.17, 95%CI: 6.93 to 9.43;AAPC:4.39, 95%CI: 3.26 to 3.53, respectively), and unintentional injuries (AAPC:4.66, 95%CI: 3.90 to 5.43; AAPC: 1.01, 95%CI: 0.46 to 1.56, respectively) (Table 3).

Among the population younger than 70 years, dietary risks (AAPC: 1.02, 95%CI: 0.85 to 1.19) and high systolic blood pressure (AAPC: 1.55, 95%CI: 1.29 to 1.81) replaced air pollution and child and maternal malnutrition to enter the top three risk factors for 2000–2019. In contrast, the top three risk factors remained the same between 2000 and 2019 for the population older than 70 years, and they include: high systolic blood pressure, tobacco, and air pollution (Table 3). Regarding risk factors among the population younger than 70 years, the largest increments in number and rate of DALYs were high body-mass index (AAPC: 3.70, 95%CI: 3.59 to 3.82; AAPC: 3.88, 95%CI: 3.69 to 4.07, respectively), unsafe sex (AAPC: 3.11, 95%CI: 2.80 to 3.42; AAPC: 3.28, 95%CI: 2.96 to 3.60, respectively), and low physical activity (AAPC: 2.75, 95%CI: 2.34 to 3.15; AAPC: 2.91, 95%CI: 2.46 to 3.36, respectively). In contrast, the risk factors with the largest increment in number of DALYs among those older than 70 years were high body-mass index (AAPC: 5.22, 95%CI: 4.79 to 5.65), low bone mineral density (AAPC: 5.22, 95%CI: 4.98 to 5.46), and high LDL cholesterol (AAPC: 5.02, 95%CI: 4.62 to 5.41). Furthermore, the same risk factors with different rankings were observed in terms of rate of DALYs for the same population: low bone mineral density (AAPC: 1.62, 95%CI: 1.38 to 1.86), high body-mass index (AAPC: 1.50, 95%CI: 1.01 to 2.00), and high LDL cholesterol (AAPC: 1.44, 95%CI: 1.04 to 1.84), respectively (Table 4).

For peer review only - http://bmjopen.bmj.com/site/about/guidelines.xhtml

Table 4. The AAPC of twenty risk factors in people younger and older than 70 years of age

Risk factors	Age < 70 years			Age ≥ 70 years		
	2000	2019	AAPC (95% CI)	2000	2019	AAPC (95% CI)
**Number**						
Unsafe water, sanitation, and handwashing	3,996,889 (3,183,257 to 4,887,734)	923,837 (645,583 to 1,254,723)	-7.42 (-8.15 to -6.69)*	237,825 (168,202 to 320,654)	228,667 (159,219 to 307,023)	-0.14 (-0.42 to 0.13)
Air pollution	32,546,257 (28,559,720 to 36,241,262)	24,301,291 (20,582,581 to 28,569,480)	-1.52 (-1.70 to -1.34)*	16,789,702 (14,415,333 to 19,203,189)	18,208,412 (15,565,447 to 21,260,363)	0.27 (0.00 to 0.54)*
Other environmental risks	3,596,355 (2,485,008 to 4,876,076)	3,222,783 (1,977,748 to 4,651,395)	-0.53 (-0.77 to -0.29)*	1,790,347 (1,199,126 to 2,480,222)	3,083,288 (2,034,326 to 4,259,059)	2.86 (2.48 to 3.23)*
Child and maternal malnutrition	27,473,911 (24,705,249 to 30,472,454)	6,937,292 (6,012,640 to 7,958,846)	-6.98 (-7.79 to -6.16)*	256,375 (197,956 to 332,924)	316,491 (248,797 to 398,362)	1.15 (0.66 to 1.63)*
Tobacco	37,227,472 (33,809,017 to 40,999,710)	38,743,936 (32,232,707 to 46,296,710)	0.22 (0.08 to 0.37)*	20,037,114 (18,407,452 to 21,694,478)	25,372,064 (22,095,893 to 28,843,357)	1.24 (0.79 to 1.69)*
Alcohol use	12,593,606 (10,822,456 to 14,698,783)	13,876,946 (11,135,736 to 16,941,170)	0.47 (0.39 to 0.56)*	2,079,979 (1,635,218 to 2,555,460)	3,388,114 (2,514,663 to 4,384,540)	2.51 (2.10 to 2.92)*
Drug use	5,880,735 (5,044,491 to 6,863,455)	4,596,666 (3,725,262 to 5,556,055)	-1.24 (-1.46 to -1.02)*	420,543 (338,823 to 505,234)	525,128 (420,389 to 638,546)	1.10 (0.84 to 1.35)*
High fasting plasma glucose	10,850,938 (8,940,115 to 13,134,425)	16,082,215 (12,638,442 to 19,989,086)	2.09 (1.69 to 2.50)*	7,073,207 (5,239,551 to 9,435,756)	12,146,224 (9,068,765 to 16,392,373)	2.86 (2.43 to 3.29)*
High systolic blood pressure	21,853,891 (18,103,552 to 25,435,298)	28,919,385 (23,316,926 to 34,533,463)	1.55 (1.29 to 1.81)*	13,487,603 (10,597,533 to 16,167,287)	25,522,231 (20,992,213 to 30,524,269)	3.37 (2.90 to 3.84)*
High body-mass index	9,311,731 (3,191,606 to 17,435,563)	18,520,264 (9,033,132 to 29,514,190)	3.70 (3.59 to 3.82)*	2,392,758 (706,129 to 5,015,786)	6,309,777 (2,532,862 to 11,224,097)	5.22 (4.79 to 5.65)*
Low bone mineral density	1,234,815 (1,026,536 to 1,443,017)	1,913,892 (1,512,289 to 2,304,790)	2.37 (2.10 to 2.64)*	534,562 (448,070 to 635,290)	1,406,383 (1,058,504 to 1,740,813)	5.22 (4.98 to 5.46)*
Dietary risks	24,107,231 (19,699,737 to 29,721,184)	29,061,110 (22,436,447 to 36,581,781)	1.02 (0.85 to 1.19)*	10,146,090 (7,449,653 to 13,767,645)	17,752,016 (12,683,501 to 23,791,732)	2.90 (2.59 to 3.21)*
Low physical activity	479,307 (211,814 to 1,031,049)	793,859 (332,155 to 1,622,549)	2.75 (2.34 to 3.15)*	795,916 (391,872 to 1,491,328)	1,713,262 (787,433 to 3,275,090)	3.97 (3.34 to 4.60)*
Occupational risks	14,894,928 (12,651,846 to 17,338,599)	11,828,821 (9,596,301 to 14,324,948)	-1.20 (-1.31 to -1.10)*	3,676,403 (2,901,359 to 4,413,363)	3,658,028 (2,941,788 to 4,424,853)	-0.10 (-0.45 to 0.25)
Intimate partner violence	766,126 (417,803 to 1,191,498)	739,392 (335,422 to 1,209,468)	-0.12 (-0.36 to 0.12)*	23,715 (10,039 to 44,392)	50,534 (16,543 to 99,799)	4.12 (3.96 to 4.29)*
Unsafe sex	1,352,963 (1,174,728 to 1,769,992)	2,445,381 (1,761,748 to 2,945,569)	3.11 (2.80 to 3.42)*	129,732 (113,048 to 182,721)	279,416 (197,910 to 338,222)	4.29 (3.79 to 4.79)*
Non-optimal temperature	3,641,568 (2,714,325 to 4,594,010)	3,468,758 (2,670,613 to 4,405,174)	-0.24 (-1.11 to 0.64)	4,785,750 (4,234,955 to 5,404,845)	5,856,605 (4,952,561 to 6,867,429)	0.76 (0.49 to 1.02)*
Kidney dysfunction	6,244,259 (5,571,754 to 7,041,339)	7,536,042 (6,221,117 to 9,011,471)	1.08 (0.82 to 1.35)*	2,639,944 (2,227,097 to 3,100,279)	5,817,866 (4,717,831 to 7,022,861)	4.30 (3.63 to 4.97)*
High LDL cholesterol	8,217,261 (6,919,451 to 9,686,198)	12,184,167 (9,541,238 to 14,971,019)	2.16 (1.91 to 2.42)*	3,000,527 (1,868,209 to 4,589,459)	7,629,795 (4,751,976 to 11,248,818)	5.02 (4.62 to 5.41)*
Childhood sexual abuse and bullying	755,840 (307,189 to 1,434,919)	629,932 (269,309 to 1,156,818)	-0.95 (-1.06 to -0.84)*	6,905 (2,954 to 12,651)	16,924 (7,261 to 32,044)	4.86 (4.72 to 5.00)*
**Rate**						
Unsafe water, sanitation, and handwashing	83 (66 to 102)	20 (14 to 27)	-7.24 (-7.97 to -6.51)*	433 (306 to 584)	212 (147 to 284)	-3.66 (-3.92 to -3.40)*
Air pollution	680 (596 to 757)	523 (443 to 615)	-1.36 (-1.53 to -1.19)*	30,565 (26,242 to 34,958)	16,865 (14,417 to 19,692)	-3.04 (-3.35 to -2.74)*
Other environmental risks	75 (52 to 102)	69 (43 to 100)	-0.37 (-0.59 to -0.14)*	3,259 (2,183 to 4,515)	2,856 (1,884 to 3,945)	-0.64 (-0.95 to -0.32)*
Child and maternal malnutrition	574 (516 to 636)	149 (130 to 171)	-6.83 (-7.48 to -6.18)*	467 (360 to 606)	293 (230 to 369)	-2.43 (-2.80 to -2.06)*
Tobacco	777 (706 to 856)	835 (694 to 997)	0.38 (0.23 to 0.53)*	36,476 (33,510 to 39,494)	23,500 (20,466 to 26,716)	-2.29 (-2.71 to -1.87)*
Alcohol use	263 (226 to 307)	299 (240 to 365)	0.61 (0.33 to 0.90)*	3,786 (2,977 to 4,652)	3,138 (2,329 to 4,061)	-1.09 (-1.57 to -0.61)*
Drug use	123 (105 to 143)	99 (80 to 120)	-1.02 (-1.22 to -0.82)*	766 (617 to 920)	486 (389 to 591)	-2.34 (-2.54 to -2.15)*

For peer review only - http://bmjopen.bmj.com/site/about/guidelines.xhtml

High fasting plasma glucose	227 (187 to 274)	346 (272 to 431)	2.25 (1.82 to 2.69)*	12,876 (9,538 to 17,177)	11,250 (8,400 to 15,183)	-0.79 (-1.48 to -0.10)*
High systolic blood pressure	456 (378 to 531)	623 (502 to 744)	1.72 (1.47 to 1.98)*	24,553 (19,292 to 29,432)	23,639 (19,444 to 28,272)	-0.15 (-0.42 to 0.13)
High body-mass index	194 (67 to 364)	399 (195 to 636)	3.88 (3.69 to 4.07)*	4,356 (1,285 to 9,131)	5,844 (2,346 to 10,396)	1.50 (1.01 to 2.00)*
Low bone mineral density	26 (21 to 30)	41 (33 to 50)	2.54 (2.20 to 2.89)*	973 (816 to 1,157)	1,303 (980 to 1,612)	1.62 (1.38 to 1.86)*
Dietary risks	503 (411 to 621)	626 (483 to 788)	1.19 (1.02 to 1.36)*	18,470 (13,562 to 25,063)	16,442 (11,748 to 22,037)	-0.58 (-0.74 to -0.43)*
Low physical activity	10 (4 to 22)	17 (7 to 35)	2.91 (2.46 to 3.36)*	1,449 (713 to 2,715)	1,587 (729 to 3,033)	0.40 (-0.21 to 1.02)*
Occupational risks	311 (264 to 362)	255 (207 to 309)	-1.05 (-1.10 to -0.99)*	6,693 (5,282 to 8,034)	3,388 (2,725 to 4,098)	-3.51 (-3.97 to -3.05)*
Intimate partner violence	16 (9 to 25)	16 (7 to 26)	0.02 (-0.23 to 0.27)*	43 (18 to 81)	47 (15 to 92)	0.49 (0.31 to 0.66)*
Unsafe sex	28 (25 to 37)	53 (38 to 63)	3.28 (2.96 to 3.60)*	236 (206 to 333)	259 (183 to 313)	0.69 (0.16 to 1.22)*
Non-optimal temperature	76 (57 to 96)	75 (58 to 95)	-0.05 (-0.89 to 0.81)*	8,712 (7,710 to 9,839)	5,425 (4,587 to 6,361)	-2.59 (-3.60 to -1.56)*
Kidney dysfunction	130 (116 to 147)	162 (134 to 194)	1.25 (1.00 to 1.49)*	4,806 (4,054 to 5,644)	5,389 (4,370 to 6,505)	0.64 (-0.02 to 1.31)
High LDL cholesterol	172 (144 to 202)	262 (206 to 322)	2.33 (2.07 to 2.59)*	5,462 (3,401 to 8,355)	7,067 (4,401 to 10,419)	1.44 (1.04 to 1.84)*
Childhood sexual abuse and bullying	16 (6 to 30)	14 (6 to 25)	-0.80 (-0.89 to -0.72)*	13 (5 to 23)	16 (7 to 30)	1.21 (1.06 to 1.37)*

AAPC: average annual percent change; DALYs, disability adjusted life years; UI, uncertainty interval, CI, confidence interval.
*AAPC significantly differs from zero at a two-tailed level of 0.05.

For peer review only

For peer review only - http://bmjopen.bmj.com/site/about/guidelines.xhtml

Discussion

The trends we observed in the population structure of China highlights the potential for an irreversible population decline to occur before the household wealth of G7 nations is achieved in China. Despite easing of birth limits in China, couples have been put off by high costs of living, expensive childcare, career choices, and maternity leave. In addition, the post-working population has been increasing at an accelerating rate, suggesting a clear deterioration in population structure and ageing[14]. Thus, the ability of China to ease strains on its ageing population in the next 30 years is a pressing concern[16].

On the one hand, continuous effort is needed to address the top three causes and risk factors which have been identified among the population in China between 2000 and 2019. Our findings show that neoplasms and cardiovascular diseases remain among the top three causes, and tobacco remains among the top three risk factors for individuals younger than 70 years. Comprehensive regional-specific strategies, including effective tobacco-control policy, recommendations for healthier lifestyles, and control of chronic infections, are needed as the cancer spectrum in China is changing from that of a developing country to that of a developed country[17, 18]. Regulatory reforms also need to further reduce the lag between approval of new cancer drugs by the US FDA and their subsequent approval in China, and they need to facilitate the development and registration of drugs that may have clinical superiority over existing drugs[19]. Turning the inverted pyramid of the health care system is essential for the battle against cardiovascular disease due to the increasing number of older patients, the high proportion of out-of-hospital deaths, and the gaps in lifestyle indicators between what is preferred and what is currently observed[20, 21]. Life course management of disease across the entire spectrum of health care services has led to a shift from a patient-centered and treatment-dominated model to a people-centered and health-centered model. It is hypothesized that the latter may help to integrate preventive and treatment measures for cardiovascular disease[22]. Tobacco sales have also increased despite a tight anti-smoking policy in China. Potential interference from the tobacco industry and the social currency of tobacco are suspected to be factors[23-25]. Therefore, great efforts should be taken not only to minimize these practices, but also to slow tobacco production[26].

On the other hand, it is crucial to focus on incremental driving causes and risk factors in the early stages to

For peer review only - http://bmjopen.bmj.com/site/about/guidelines.xhtml

mitigate future ageing-driven disease burden. Our data show that musculoskeletal disorders, dietary risks, and high systolic blood pressure are among the top three causes and risk factors in people younger than 70 years. Split analyses were performed to examine variations according to age, and a cut-off point of 70 years was used because ischemic heart disease and stroke were the top-ranked causes of DALYs in the population aged 70 years and older[14]. A previous study quantified the temporal trends in disease burden of musculoskeletal disorders which could be attributed to changes in risk factors [27]. The results obtained support population-wide initiatives which target high body-mass index to help mitigate the burden of musculoskeletal disorders[27]. Changes in diets to correct for urbanization-driven dietary shifts to higher consumption of refined sugars, refined fats, oils, and meats may also help prevent diet-related and chronic non-communicable diseases[28]. With resources available to control high systolic blood pressure and reduce the burden of hypertension, efforts are needed to improve access to high value antihypertensive medications and to implement artificial intelligence-based digital health[29, 30].

We identified key domains that require intervention in order to reduce an increasing disease burden among individuals older than 70 years. Most importantly, the top three causes and risk factors for this age group remained unchanged. Thus, our findings indicate that greater effort is needed to tackle the rapidly increasing age-standardized rate of DALYs due to traditional major risk factors, including high body-mass index, low bone mineral density, and high LDL cholesterol. Moreover, the increasing burden of maternal and neonatal disorders, HIV/AIDS and sexually transmitted infections, and unintentional injuries needs to be highlighted as well.

Health care for older individuals is undergoing unprecedented challenges due to underprepared geriatric hospitals and nursing homes, an unbalanced health care insurance system between urban and rural areas, and an absence of education on national aging and post-working health[16]. Consequently, there is an urgent need to identify traits in older people who experience short ill-spans. By identifying healthy behaviors and casual genetic and metabolic factors of the latter, it may be possible to decrease the burden of late-life diseases and extend healthspan[31]. The development of long-term health systems to meet the needs of older individuals in an age-friendly environment is fundamental to fostering healthy ageing and maximizing the functional abilities of older individuals[32].

For peer review only - http://bmjopen.bmj.com/site/about/guidelines.xhtml

Health care has experienced an upsurge in regard to medical institutions, personnel, beds, and expenditures. Our findings clearly demonstrate that China has increased its health spending much faster than its economic growth. In 2018, China provided $644.7 million for the development of health assistance[33]. This trend in increasing health care expenditures will be further aggravated considering the high costs related to chronic illnesses, population growth, and ageing in the United States[9, 34]. While China has reformed its primary health care system to insure its population[7, 35], improving the quality of primary health care and increasing reimbursement for chronic non-communicable diseases are key steps to alleviating national disease burden[36, 37]. In addition, the cost-effectiveness of heath expenditures on disease burden and targeting the poor should be primary considerations in public health policy given the limited financial resources for heath[38].

It must also be noted that there were several limitations associated with the present study. First, temporal trends in health care utilization, risk factors, and disease burden were not quantified due to changes in the age structure of the population and temporal trends in disease burden attributable to changes in risk factors. Second, population and health care data from CSY 2020 were combined with modeled disease burden data from GBD 2019 based on an assumption that these two sources of data are consistent. Third, the most recent trends could not be captured due to time lags in data release by the NBS of China and GBD studies. Fourth, all of the limitations of the GBD methodology described elsewhere [3, 14, 15] could have potentially affected the present study. Fifth, owing to the nature of the data and methods from CSY, we are currently unable to parse population estimates into more narrow age groups and provide deflator-adjusted health expenditure data. National-level data were collected and analyzed, and therefore, within-country variations were not considered.

Conclusion

Comprehensive regional-specific strategies that target the younger population in China are needed to mitigate the causes and risk factors which have paralleled dynamic population changes that have occurred between 2000 and 2019 in China. We have identified key domains that require intervention in order to reduce an increasing disease burden among individuals currently older than 70 years, as well as those approaching this age group.

For peer review only - http://bmjopen.bmj.com/site/about/guidelines.xhtml

Contributors

Dr. Dongze Wu and Prof. Jieruo Gu had full access to all of the data in the study and takes responsibility for the integrity of the data and the accuracy of the data analysis. Dr. Dongze Wu, Dr.Zhenguo Liang, Dr. Cui Guo, and Prof. Jieruo Gu conceived and designed the study, performed the analysis, wrote the paper. All authors reviewed and revised the paper.

Conflict of Interest

The authors confirm that there are no conflicts of interest.

Funding Sources

Project funded by China Postdoctoral Science Foundation (2020TQ0380) and Guangdong Clinical Research Center of Immune disease (2020B1111170008). The Global Burden of Disease Study is funded by the Bill and Melinda Gates Foundation. The funder was not involved in the preparation of this manuscript.

Data sharing statement

Data used for the analyses are publicly available from the Institute of Health Metrics and Evaluation (www.ihme.org) and the National Bureau of Statistics of China (https://data.stats.gov.cn/english/index.htm; http://www.stats.gov.cn/tjsj/ndsj/2020/indexeh.htm).

For peer review only

For peer review only - http://bmjopen.bmj.com/site/about/guidelines.xhtml

References

1. Lopez-Otin C, Blasco MA, Partridge L, Serrano M, Kroemer G. The hallmarks of aging. Cell. 2013;153(6):1194-217.
2. Lopez-Otin C, Kroemer G. Hallmarks of Health. Cell. 2021;184(1):33-63.
3. Collaborators GBDD. Global age-sex-specific fertility, mortality, healthy life expectancy (HALE), and population estimates in 204 countries and territories, 1950-2019: a comprehensive demographic analysis for the Global Burden of Disease Study 2019. Lancet. 2020;396(10258):1160-203.
4. Vollset SE, Goren E, Yuan CW, Cao J, Smith AE, Hsiao T, et al. Fertility, mortality, migration, and population scenarios for 195 countries and territories from 2017 to 2100: a forecasting analysis for the Global Burden of Disease Study. Lancet. 2020;396(10258):1285-306.
5. Zeng Y, Hesketh T. The effects of China's universal two-child policy. Lancet. 2016;388(10054):1930-8.
6. Tatum M. China's three-child policy. Lancet. 2021;397(10291):2238.
7. Li X, Lu J, Hu S, Cheng KK, De Maeseneer J, Meng Q, et al. The primary health-care system in China. Lancet. 2017;390(10112):2584-94.
8. Yip W, Fu H, Chen AT, Zhai T, Jian W, Xu R, et al. 10 years of health-care reform in China: progress and gaps in Universal Health Coverage. Lancet. 2019;394(10204):1192-204.
9. Moses H, 3rd, Matheson DH, Dorsey ER, George BP, Sadoff D, Yoshimura S. The anatomy of health care in the United States. JAMA. 2013;310(18):1947-63.
10. Zhong NS, Zheng BJ, Li YM, Poon, Xie ZH, Chan KH, et al. Epidemiology and cause of severe acute respiratory syndrome (SARS) in Guangdong, People's Republic of China, in February, 2003. Lancet. 2003;362(9393):1353-8.
11. Zhu N, Zhang D, Wang W, Li X, Yang B, Song J, et al. A Novel Coronavirus from Patients with Pneumonia in China, 2019. N Engl J Med. 2020;382(8):727-33.
12. Gong P, Liang S, Carlton EJ, Jiang Q, Wu J, Wang L, et al. Urbanisation and health in China. Lancet. 2012;379(9818):843-52.
13. Ning JJ, Xian ZD, Li XC, Mao YF, Sheng LY, Zeng YP, et al. China Statistical Yearbook 2020 [Available from: http://www.stats.gov.cn/tjsj/ndsj/2020/indexeh.htm.
14. Diseases GBD, Injuries C. Global burden of 369 diseases and injuries in 204 countries and territories, 1990-2019: a systematic analysis for the Global Burden of Disease Study 2019. Lancet. 2020;396(10258):1204-22.
15. Collaborators GBDRF. Global burden of 87 risk factors in 204 countries and territories, 1990-2019: a systematic analysis for the Global Burden of Disease Study 2019. Lancet. 2020;396(10258):1223-49.
16. Wang XQ, Chen PJ. Population ageing challenges health care in China. Lancet. 2014;383(9920):870.
17. Feng RM, Zong YN, Cao SM, Xu RH. Current cancer situation in China: good or bad news from the 2018 Global Cancer Statistics? Cancer Commun (Lond). 2019;39(1):22.
18. Chen W, Xia C, Zheng R, Zhou M, Lin C, Zeng H, et al. Disparities by province, age, and sex in site-specific cancer burden attributable to 23 potentially modifiable risk factors in China: a comparative risk assessment. Lancet Glob Health. 2019;7(2):e257-e69.
19. Li G, Liu Y, Xie C, Zhou Q, Chen X. Characteristics of expedited programmes for cancer drug approval in China. Nat Rev Drug Discov. 2021;20(6):416.
20. Zhao D, Liu J, Wang M, Zhang X, Zhou M. Epidemiology of cardiovascular disease in China: current features and implications. Nat Rev Cardiol. 2019;16(4):203-12.
21. Du X, Patel A, Anderson CS, Dong J, Ma C. Epidemiology of Cardiovascular Disease in China and Opportunities for Improvement: JACC International. J Am Coll Cardiol. 2019;73(24):3135-47.
22. Liu S, Li Y, Zhou M. Addressing the Cardiovascular Disease Burden in China-Is It Possible Without Surgery?-Reply. JAMA Cardiol. 2019;4(9):953.

For peer review only - http://bmjopen.bmj.com/site/about/guidelines.xhtml

23. Luo H, Zhao Y. Increasing tobacco sales under the anti-smoking policy in China. Lancet. 2020;395(10240):1834.
24. Yang G, Wang Y, Wu Y, Yang J, Wan X. The road to effective tobacco control in China. Lancet. 2015;385(9972):1019-28.
25. Rich ZC, Xiao S. Tobacco as a social currency: cigarette gifting and sharing in China. Nicotine Tob Res. 2012;14(3):258-63.
26. Dan X, Yuankai S, Chen W. Tobacco in China. Lancet. 2014;383(9934):2045-6.
27. Wu D, Wong P, Guo C, Tam LS, Gu J. Pattern and trend of five major musculoskeletal disorders in China from 1990 to 2017: findings from the Global Burden of Disease Study 2017. BMC Med. 2021;19(1):34.
28. Tilman D, Clark M. Global diets link environmental sustainability and human health. Nature. 2014;515(7528):518-22.
29. Su M, Zhang Q, Bai X, Wu C, Li Y, Mossialos E, et al. Availability, cost, and prescription patterns of antihypertensive medications in primary health care in China: a nationwide cross-sectional survey. Lancet. 2017;390(10112):2559-68.
30. Brouwers S, Sudano I, Kokubo Y, Sulaica EM. Arterial hypertension. Lancet. 2021.
31. Partridge L, Deelen J, Slagboom PE. Facing up to the global challenges of ageing. Nature. 2018;561(7721):45-56.
32. Beard JR, Officer A, de Carvalho IA, Sadana R, Pot AM, Michel JP, et al. The World report on ageing and health: a policy framework for healthy ageing. Lancet. 2016;387(10033):2145-54.
33. Global Burden of Disease Health Financing Collaborator N. Past, present, and future of global health financing: a review of development assistance, government, out-of-pocket, and other private spending on health for 195 countries, 1995-2050. Lancet. 2019;393(10187):2233-60.
34. Dieleman JL, Squires E, Bui AL, Campbell M, Chapin A, Hamavid H, et al. Factors Associated With Increases in US Health Care Spending, 1996-2013. JAMA. 2017;318(17):1668-78.
35. The L. China's health-care reform: an independent evaluation. Lancet. 2019;394(10204):1113.
36. Jan S, Laba TL, Essue BM, Gheorghe A, Muhunthan J, Engelgau M, et al. Action to address the household economic burden of non-communicable diseases. Lancet. 2018;391(10134):2047-58.
37. Li X, Krumholz HM, Yip W, Cheng KK, De Maeseneer J, Meng Q, et al. Quality of primary health care in China: challenges and recommendations. Lancet. 2020;395(10239):1802-12.
38. Li H, Jiang L. Catastrophic medical insurance in China. Lancet. 2017;390(10104):1724-5.

For peer review only

For peer review only - http://bmjopen.bmj.com/site/about/guidelines.xhtml

Figure legends

Figure 1. Temporal population trends in China between 2000 and 2019.

Temporal population composition is presented according to gender and area (A), age group (B), birth rate, death rate, and natural growth rate (C), and gross ratio, children ratio, and old dependency ratio (D).

For peer review only

For peer review only - http://bmjopen.bmj.com/site/about/guidelines.xhtml

Supplemental appendix

sFigure 1. Trend in life expectancy and healthy life expectancy at birth and at 60–64 years according to gender between 2000 and 2019.

sTable 1. AAPC in life expectancy at birth and at 60–64 years for 2000–2019

sTable 2. Percentage of healthy life expectancy within total life expectancy at birth for 2000–2019

sTable 3. Percentage of healthy life expectancy within total life expectancy at 60–64 years for 2000–2019

sTable 4. AAPC of all-cause disease burden for 2000–2019

sTable 5. AAPC of causes for 2000–2019

sTable 6. AAPC of risk factors for 2000–2019

For peer review only

For peer review only - http://bmjopen.bmj.com/site/about/guidelines.xhtml

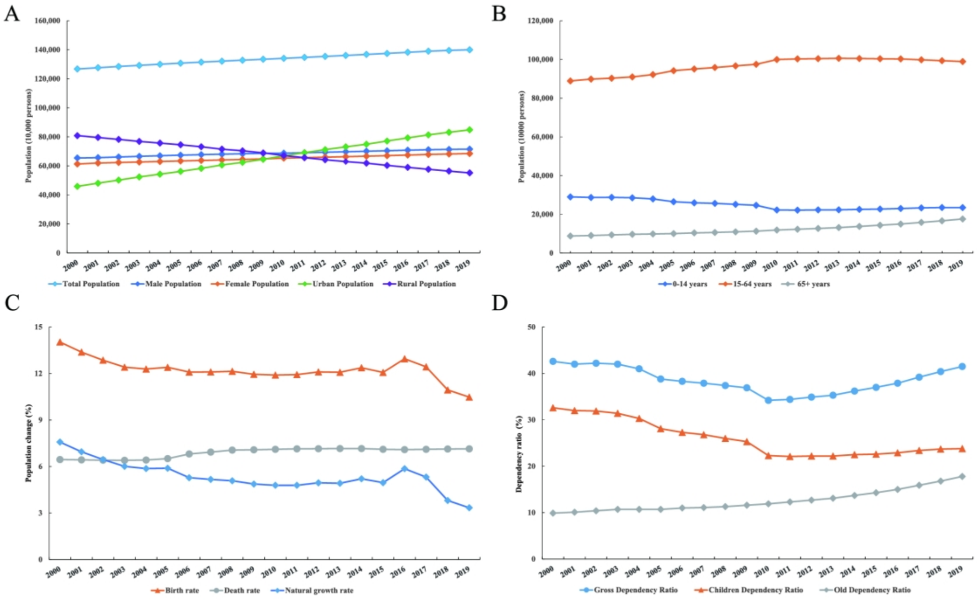

Temporal population trends in China between 2000 and 2019.

200x123mm (300 x 300 DPI)

Supplemental Appendix

Trends in population, health care, and disease burden in China, 2000–2019

Table of Contents

Glossary and Definitions

Section 1: Population

Section 2: Health Care Institutions

Section 3: Medical Personnel

Section 4: Beds in Health Care Institutions

Section 5: Health Expenditures

Supplementary Figures

sFigure 1. Trend in life expectancy and healthy life expectancy at birth and at 60–64 years according to gender between 2000 and 2019.

Supplementary Tables

sTable 1. AAPC in life expectancy at birth and at 60–64 years for 2000–2019

sTable 2. Percentage of healthy life expectancy within total life expectancy at birth for 2000–2019

sTable 3. Percentage of healthy life expectancy within total life expectancy at 60–64 years for 2000–2019

sTable 4. AAPC of all-cause disease burden for 2000–2019

sTable 5. AAPC of causes for 2000–2019

sTable 6. AAPC of risk factors for 2000–2019

For peer review only

Glossary and Definitions

Glossary and definitions of population, healthcare institutions, medical personnel, beds in health care institutions, and health expenditure data were compiled from the 2020 China Statistical Yearbook (CSY) (http://www.stats.gov.cn/tjsj/ndsj/2020/indexeh.htm).

Section 1: Population

Total Population (year-end) (10000 persons): Year-end population refers to the population at midnight on 31 December. Annual statistics regarding the total population do not include data for Hong Kong, the Macau special administrative region and Taiwan province, and overseas numbers.

Urban Population refers to people residing in cities and towns in China.

Rural Population refers to people who reside outside of urban areas.

Birth Rate, Death Rate, and Natural Growth Rate of Population

Birth Rate (or Crude Birth Rate) refers to the ratio of the number of births to the average population (or mid-period population) during a certain period of time (usually a year). Birth rate in the chapter refers to annual birth rate and it is calculated as: number of births / annual average population × 1000. Number of births in the formula refers to live births (i.e., when a baby has breathed or showed any vital phenomena regardless of duration of gestation). Annual average population is the average of the population at the beginning of the year and the population at the end of the year. Sometimes it is substituted by the mid-year population.

Death Rate (or Crude Death Rate) refers to the ratio of the number of deaths to the average population (or mid-period population) during a certain period of time (usually a year). Death rate in the chapter refers to annual death rate and is calculated as: number of deaths / annual average population × 1000. Total Population (year-end) (10000 persons) refers to the population at midnight on 31 December.

Gross Dependency Ratio (or Coefficient) (%) refers to the ratio of non-working-age population to the working-age population. In general, Gross Dependency Ratio describes the number of non-working-age individuals that every 100 people of working age take care of. This reflects the basic relation between population and economic development from a demographic perspective.

Children Dependency Ratio (or Coefficient) (%) refers to the ratio of children to the working-age population. It describes the number of children that every 100 people of working age take care of.

Old Dependency Ratio (or Coefficient) (%) refers to the ratio of the elderly to the working-age population. It describes the number of elderly that every 100 people of working age take care of. Old dependency ratio is one of the indicators that reflect the social implication of population aging from an economic perspective.

Note: For the years, 2000 and 2010, census year estimates were available; the rest of the data were estimated based on annual national sample surveys.

Section 2: Health Care Institutions

Number of Health Care Institutions (unit)

Medical and health care institutions refer to units which have qualified to receive a Certification of Health Care Institution by the administration of public health, or a Certification of Corporate Unit by the civil affairs, administration for industry and commerce, commission office for public sector reform. Health care institutions engage in medical care, disease prevention and control, health supervision and inspection, medicine research, and on-job training. Health care institutions include hospitals, health care institutions at the grass-root level, specialized public health institutions, and other medical and health care institutions.

Number of Hospitals (unit)

Hospitals were considered to include general hospitals, hospitals of traditional Chinese medicine, hospitals that integrate traditional Chinese medicine and Western medicine, national hospitals, specialized hospitals, and nursing hospitals. Specialized disease prevention and cure centers, maternal and child health hospitals, and sanatoriums were excluded.

For peer review only

Health Care Institutions at Grass-root Level (unit)

Health care institutions at the grass-root level included: community health service centers, community service stations, institutes of health on the street, institutes of health in villages and towns, outpatient departments, and clinics (health centers).

Note:
1. Number of village clinics was included in health care institutions.
2. Since 2002, health care institutions no longer include headquarters of higher and secondary medical schools, drug test institutions, frontier health and quarantine institutions, and family.

Section 3: Medical Personnel

Number of Medical Personnel (10000 persons)

Medical personnel refer to all employees engaged at health care institutions, such as hospitals, health care institutions at grass-root level, specialized public health institutions, and other medical and health care institutions. These employees include medical technical personnel, village doctors and assistants, other technical personnel, and managerial and service staff. Medical personnel also refer to workers with payment of wages at the end of the year, including various types of employment personnel (including contract workers) and staff rehired for more than six months. Temporary workers, emeritus and retired, persons leaving the unit while remaining labor relations, and rehired and temporarily employed individuals for less than six months were excluded.

Number of Licensed Doctors (10000 persons)

Licensed doctors refer to medical workers who have obtained licenses of qualified doctors and are employed in medical treatment, disease prevention, or healthcare institutions. Licensed doctors engaged in management positions were excluded. Licensed doctors are divided into four categories: clinician, Chinese medicine physician, dentist, and public health physician.

Number of Registered Nurses (10000 persons)

Registered nurses refer to practical nursing personnel with a certificate of registered nurse. Nurses engaged in management positions were excluded.

Number of Other Technical Personnel (10000 persons)

Other technical personnel represent staff who graduated from high school and other institutions in the majors of chemistry and mathematics, as well as other non-health professionals, who are engaged in health promotion, research, teaching, and other technical work.

Number of Administrative Personnel (10000 persons)

Administrative personnel include persons in charge, personnel mainly engaged in health care, disease control, health surveillance, medical research, and teaching, other business management, and personnel mainly engaged in the party and government, personnel, finance, information, security, and other administrative work.

Note: Data of medical personnel and medical technical personnel include 10000 sanitation supervisors who serve as public servants.

Section 4: Beds in Health Care Institutions

Number of Beds in Health Care Institutions (10000 units)

Number of beds in health care institutions refers to a fixed sum of beds (non-prepared beds) at the end of the year. These include formal beds, simple beds, care beds, beds being disinfected and repaired, and disabled beds due to expansion or overhaul. Obstetric neonatal beds, expectant beds of confinement rooms, inventory beds, observation beds, extra beds, and escort beds for patient's family members were excluded.

Number of Beds in Medical Institutions per 10000 Population

Number of beds in medical institutions refers to a fixed sum of beds (non-prepared beds) at the end of the year. These include formal beds, simple beds, care beds, beds being disinfected and repaired, and disabled beds due to expansion or overhaul. Obstetric neonatal beds, expectant beds of confinement rooms, inventory beds, observation beds, extra beds, and escort beds for patient's family members were excluded.

For peer review only

Number of Beds in Urban Medical Institutions (10000 units)

Number of beds in urban medical institutions refers to a fixed sum of beds (non-prepared beds) at the end of the year. These include formal beds, simple beds, care beds, beds being disinfected and repaired, and disabled beds due to expansion or overhaul. Obstetric neonatal beds, expectant beds of confinement rooms, inventory beds, observation beds, extra beds, and escort beds for patient's family members were excluded.

Number of Beds in Rural Medical Institutions (10000 units)

Number of beds in rural medical institutions refers to a fixed sum of beds (non-prepared beds) at the end of the year. These include formal beds, simple beds, care beds, beds being disinfected and repaired, and disabled beds due to expansion or overhaul. Obstetric neonatal beds, expectant beds of confinement rooms, inventory beds, observation beds, extra beds, and escort beds for patient's family members were excluded.

Beds of Medical Institutions per 10000 Population

Beds of Medical Institutions per 10000 Population = number of beds of medical and health care institutions / population ×10000. The population value used is based on household registration data from the Ministry of Public Security.

Section 5: Health Expenditures

Total Health Expenditure (100 million yuan)

Total health expenditure refers to the total expenditure on medical and health care services (usually in monetary value) by a country or region within a certain period of time (usually a year). Total health expenditure is counted according to the source method. Under certain economic conditions, it reflects the emphasis and burden level of cost on government health care, the society's healthcare, and on individual citizens. Total health expenditure also represents the main features of health financing mode and fairness, as well as the rationality of health financing.

Government Health Expenditure (100 million yuan)

Government expenditure on public health refers to expenditures by the government at all levels of medical and health care services, medical subsidies, health administration and health insurance management, and undertakings of family planning.

Social Health Expenditure (100 million yuan)

Social expenditure on public health refers to all inputs from society, and not from the government in the form of expenditures on social medical security, commercial health insurance, private expenditure on operation of medical and health care, social donations and contributions, and income from administrative fees.

Out-of-pocket Health Expenditure (100 million yuan)

Individual cash expenditure on health refers to the expenditure in cash by rural and urban residents for various health services, including self-payments of residents within the system of multi-medical insurance.

Per Capita Health Expenditure (yuan)

Per capita health expenditure refers to the ratio of total expenditure on health in a year to the average population.

For peer review only

Supplementary Figures

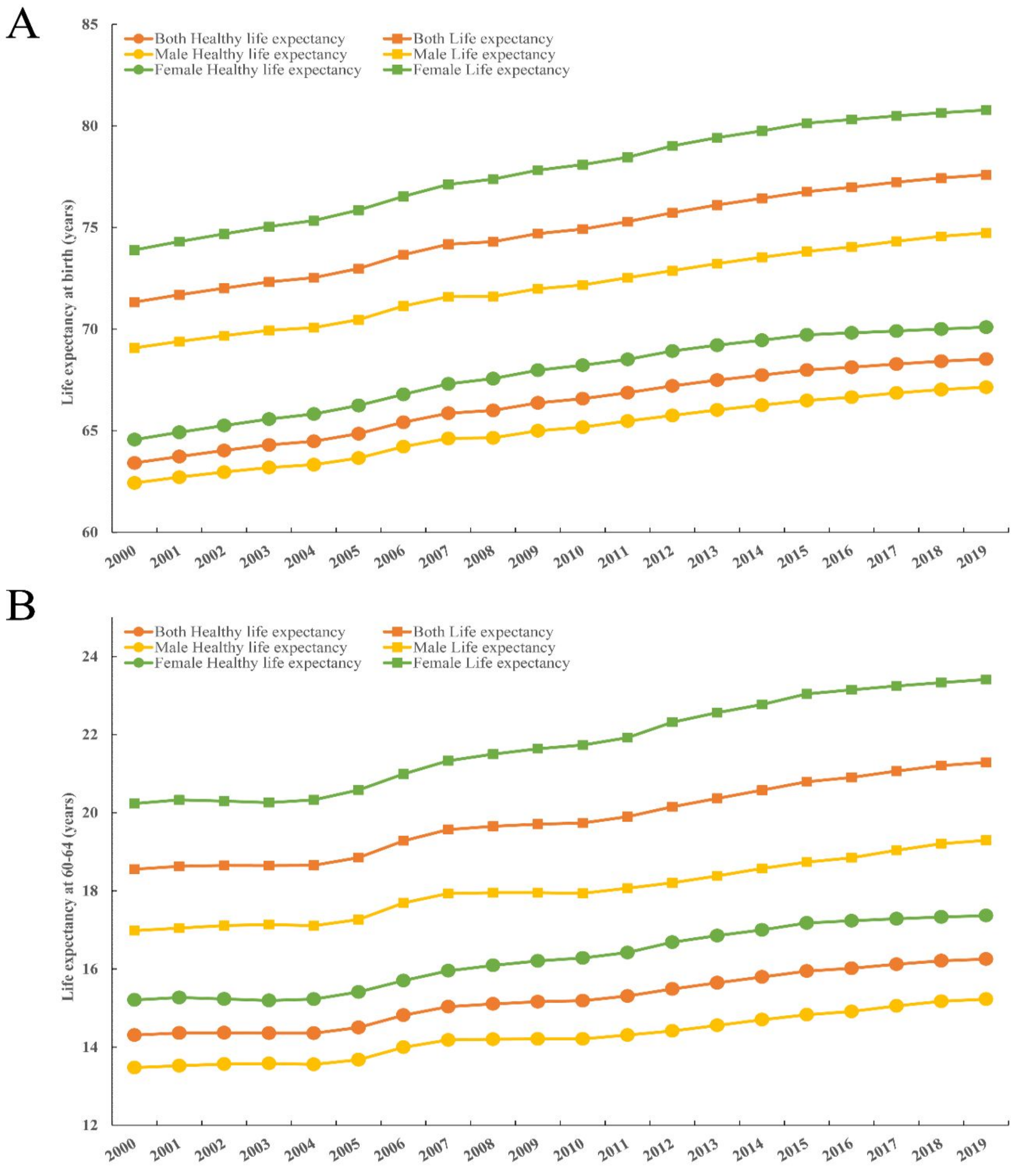

sFigure 1. Trend in life expectancy and healthy life expectancy at birth and at 60–64 years according to gender between 2000 and 2019.

The trend of life expectancy and healthy life expectancy at birth (A) and at 60–64 years (B) according to gender between 2000 and 2019. The distance between life expectancy and healthy life expectancy denotes unhealthy life expectancy (ill-span).

Supplementary Tables

sTable 1. AAPC in life expectancy at birth and at 60–64 years for 2000–2019

	Life expectancy (95% UI)		AAPC (95% CI)		
	2000	2019	2000–2019	2000–2009	2010–2019
		**Life expectancy at birth**			
Both					
Life expectancy	71.33 (70.56 to 72.06)	77.59 (76.27 to 79.00)	0.44 (0.43 to 0.46)*	0.50 (0.47 to 0.53)*	0.40 (0.38 to 0.42)*
Healthy life expectancy	63.42 (60.07 to 65.54)	68.53 (60.88 to 70.97)	0.41 (0.38 to 0.43)*	0.50 (0.45 to 0.54)*	0.31 (0.29 to 0.34)*
Male					
Life expectancy	69.08 (68.02 to 70.16)	74.74 (72.78 to 76.70)	0.42 (0.38 to 0.46)*	0.46 (0.37 to 0.54)*	0.39 (0.37 to 0.41)*
Healthy life expectancy	62.43 (60.44 to 64.36)	67.14 (60.64 to 69.82)	0.38 (0.36 to 0.41)*	0.44 (0.39 to 0.49)*	0.32 (0.30 to 0.35)*
Female					
Life expectancy	73.90 (72.91 to 74.81)	80.78 (79.10 to 82.47)	0.47 (0.43 to 0.51)*	0.57 (0.50 to 0.63)*	0.37 (0.32 to 0.41)*
Healthy life expectancy	64.57 (60.86 to 66.96)	70.10 (60.92 to 73.19)	0.43 (0.40 to 0.46)*	0.56 (0.50 to 0.62)*	0.30 (0.27 to 0.33)*
		**Life expectancy at 60–64 years**			
Both					
Life expectancy	18.56 (18.06 to 19.02)	21.29 (20.32 to 22.33)	0.76 (0.60 to 0.91)*	0.65 (0.37 to 0.93)*	0.91 (0.81 to 1.01)*
Healthy life expectancy	14.31 (13.16 to 15.43)	16.26 (14.82 to 17.67)	0.69 (0.51 to 0.87)*	0.64 (0.27 to 1.01)*	0.73 (0.66 to 0.80)*
Male					
Life expectancy	16.99 (16.32 to 17.66)	19.29 (17.97 to 20.68)	0.68 (0.61 to 0.74)*	0.58 (0.46 to 0.70)*	0.85 (0.80 to 0.89)*
Healthy life expectancy	13.48 (12.42 to 14.53)	15.23 (13.82 to 16.79)	0.65 (0.55 to 0.76)*	0.56 (0.37 to 0.75)*	0.81 (0.76 to 0.86)*
Female					
Life expectancy	20.24 (19.62 to 20.86)	23.41 (22.10 to 24.77)	0.76 (0.55 to 0.98)*	0.75 (0.41 to 1.09)*	0.76 (0.47 to 1.04)*
Healthy life expectancy	15.21 (13.83 to 16.46)	17.37 (15.61 to 19.16)	0.70 (0.54 to 0.85)*	0.69 (0.40 to 0.98)*	0.68 (0.54 to 0.81)*

Note: AAPC: average annual percent changes; UI, uncertainty interval; CI, confidence interval.
*AAPC significantly differs from zero at the alpha = 0.05 level.

For peer review only

sTable 2. Percentage of healthy life expectancy within total life expectancy at birth for 2000–2019

Year	Healthy life expectancy at birth (years)			Life expectancy at birth (years)			Healthy within total life expectancy (%)		
	Total	Male	Female	Total	Male	Female	Total	Male	Female
2000	63.42	62.43	64.57	71.33	69.08	73.90	88.90	90.38	87.37
2001	63.73	62.72	64.92	71.69	69.40	74.31	88.90	90.37	87.37
2002	64.03	62.96	65.26	72.02	69.67	74.69	88.90	90.37	87.38
2003	64.30	63.20	65.57	72.33	69.94	75.05	88.89	90.36	87.38
2004	64.49	63.33	65.83	72.54	70.08	75.34	88.90	90.37	87.37
2005	64.86	63.66	66.25	72.98	70.47	75.86	88.87	90.34	87.33
2006	65.42	64.22	66.79	73.67	71.14	76.54	88.80	90.27	87.27
2007	65.87	64.62	67.31	74.19	71.60	77.12	88.79	90.25	87.27
2008	66.00	64.65	67.57	74.31	71.61	77.38	88.83	90.28	87.32
2009	66.38	65.00	67.98	74.70	71.99	77.82	88.85	90.30	87.35
2010	66.58	65.18	68.22	74.92	72.17	78.10	88.87	90.31	87.36
2011	66.88	65.48	68.51	75.29	72.53	78.46	88.82	90.27	87.31
2012	67.21	65.75	68.92	75.73	72.88	79.03	88.75	90.22	87.21
2013	67.50	66.03	69.21	76.11	73.23	79.42	88.69	90.17	87.14
2014	67.74	66.26	69.45	76.44	73.53	79.76	88.62	90.11	87.08
2015	67.99	66.49	69.72	76.77	73.83	80.14	88.56	90.06	87.00
2016	68.12	66.65	69.82	76.98	74.05	80.32	88.49	90.01	86.93
2017	68.28	66.86	69.91	77.23	74.33	80.50	88.41	89.95	86.85
2018	68.42	67.03	70.01	77.44	74.57	80.65	88.36	89.89	86.81
2019	68.53	67.14	70.10	77.59	74.74	80.78	88.31	89.84	86.78

For peer review only

sTable 3. Percentage of healthy life expectancy within total life expectancy at 60–64 years for 2000–2019

Year	Healthy life expectancy at 60–64 years			Life expectancy at 60–64 year			Percentage of healthy within total life expectancy		
	Total	Male	Female	Total	Male	Female	Total	Male	Female
2000	14.31	13.48	15.21	18.56	16.99	20.24	77.12%	79.35%	75.15%
2001	14.36	13.52	15.27	18.63	17.05	20.33	77.09%	79.33%	75.11%
2002	14.37	13.57	15.23	18.65	17.11	20.30	77.05%	79.29%	75.06%
2003	14.36	13.58	15.19	18.65	17.14	20.26	76.99%	79.24%	74.98%
2004	14.36	13.56	15.23	18.66	17.11	20.33	76.97%	79.24%	74.93%
2005	14.50	13.68	15.41	18.85	17.27	20.58	76.93%	79.21%	74.87%
2006	14.82	14.00	15.70	19.29	17.69	20.99	76.84%	79.12%	74.80%
2007	15.04	14.18	15.95	19.57	17.93	21.33	76.82%	79.09%	74.79%
2008	15.11	14.20	16.09	19.65	17.95	21.50	76.87%	79.12%	74.84%
2009	15.16	14.21	16.21	19.71	17.95	21.64	76.93%	79.17%	74.90%
2010	15.19	14.21	16.28	19.74	17.94	21.73	76.96%	79.21%	74.93%
2011	15.31	14.31	16.42	19.90	18.07	21.93	76.94%	79.20%	74.89%
2012	15.49	14.42	16.69	20.15	18.21	22.32	76.87%	79.19%	74.78%
2013	15.65	14.56	16.86	20.37	18.39	22.56	76.82%	79.18%	74.71%
2014	15.80	14.71	17.00	20.58	18.58	22.77	76.77%	79.16%	74.65%
2015	15.95	14.83	17.18	20.79	18.74	23.04	76.69%	79.13%	74.55%
2016	16.02	14.91	17.23	20.91	18.85	23.15	76.62%	79.11%	74.45%
2017	16.12	15.05	17.28	21.07	19.04	23.25	76.54%	79.08%	74.35%
2018	16.21	15.17	17.33	21.21	19.21	23.33	76.45%	78.99%	74.28%
2019	16.26	15.23	17.37	21.29	19.29	23.41	76.36%	78.93%	74.18%

For peer review only

sTable 4. AAPC of all-cause disease burden for 2000–2019

Subcategory	All-cause estimates (95% UI)		AAPC (95% CI)		
	2000	2019	2000–2019	2000–2009	2010–2019
**Number**					
Incidence	5,291,848,308 (4,993,009,197 to 5,644,956,487)	5,790,243,111 (5,505,879,019 to 6,107,248,437)	0.48 (0.45 to 0.50)*	0.19 (0.16 to 0.22)*	0.82 (0.78 to 0.85)*
Prevalence	1,241,468,252 (1,234,531,622 to 1,247,534,242)	1,362,817,881 (1,356,594,402 to 1,368,714,232)	0.49 (0.49 to 0.50)*	0.47 (0.47 to 0.48)*	0.52 (0.51 to 0.52)*
Death	8,653,295 (8,170,487 to 9,200,531)	10,653,448 (9,310,978 to 12,069,760)	1.12 (0.93 to 1.31)*	0.89 (0.61 to 1.18)*	1.27 (1.03 to 1.51)*
Years lived with disability	116,137,933 (86,494,934 to 150,490,082)	153,797,874 (115,409,603 to 199,426,123)	1.49 (1.46 to 1.52)*	1.29 (1.24 to 1.34)*	1.74 (1.72 to 1.77)*
Years of life lost	260,536,746 (246,148,674 to 276,354,908)	228,407,694 (197,155,841 to 260,733,993)	-0.70 (-0.95 to -0.46)*	-1.34 (-1.53 to -1.15)*	-0.07 (-0.51 to 0.38)
DALY	376,674,679 (343,098,150 to 416,187,725)	382,205,568 (332,307,099 to 433,824,950)	0.08 (-0.06 to 0.22)	-0.44 (-0.58 to -0.31)*	0.60 (0.39 to 0.82)*
Risk factor (DALY)	176,792,905 (163,998,804 to 190,556,819)	187,705,961 (165,023,927 to 212,244,649)	0.34 (0.20 to 0.48)*	-0.27 (-0.52 to -0.03)*	0.92 (0.79 to 1.06)*
**Age standardized rate**					
Incidence	414,111 (390,563 to 440,957)	418,081 (394,196 to 444,709)	0.04 (0.02 to 0.07)*	-0.21 (-0.24 to -0.17)*	0.34 (0.30 to 0.38)*
Prevalence	94,824 (94,187 to 95,367)	93,439 (92,723 to 94,090)	-0.08 (-0.09 to -0.07)*	-0.17 (-0.19 to -0.15)*	0.00 (-0.01 to 0.00)
Death	956 (910 to 1,010)	634 (561 to 711)	-2.14 (-2.28 to -2.00)*	-2.00 (-2.20 to -1.79)*	-2.38 (-2.56 to -2.21)*
Years lived with disability	9,078 (6,779 to 11,713)	8,924 (6,654 to 11,539)	-0.08 (-0.12 to -0.05)*	-0.34 (-0.38 to -0.30)*	0.22 (0.17 to 0.28)*
Years of life lost	24,833 (23,513 to26,239)	13,347 (11,701 to14,997)	-3.22 (-3.33 to -3.11)*	-3.43 (-3.59 to -3.27)*	-3.09 (-3.23 to -2.96)*
DALY	33,911 (31,131 to 37,035)	22,271 (19,482 to 25,195)	-2.21 (-2.35 to -2.07)*	-2.54 (-2.64 to -2.43)*	-1.85 (-2.12 to -1.58)*
Risk factor (DALY)	16,689 (15,569 to 17,886)	10,310 (9,122 to 11,524)	-2.49 (-2.69 to -2.29)*	-2.93 (-3.27 to -2.58)*	-2.07 (-2.30 to -1.85)*

Note: AAPC: average annual percent changes; UI, uncertainty interval; CI, confidence interval; DALY, disability-adjusted life years. *AAPC significantly differs from zero at the alpha = 0.05 level.

For peer review only

sTable 5. AAPC of causes for 2000–2019

Causes	DALY (95% UI)				AAPC (95% CI)		
	2000	Rank	2019	Rank	2000–2019	2000–2009	2010–2019
**All-age numbers**							
Neglected tropical diseases and malaria	1,704,065 (1,121,580 to 2,525,119)	21	1,137,806 (692, 480 to 1,800,439)	22	-2.07 (-2.35 to -1.79)*	-3.57 (-3.89 to -3.25)*	-0.50 (-0.97 to -0.02)*
Nutritional deficiencies	4,183,588 (3,082,941 to 5,605,252)	19	2,606,926 (1,815,566 to 3,659,294)	18	-2.47 (-2.64 to -2.30)*	-4.30 (-4.49 to -4.11)*	-0.53 (-0.79 to -0.26)*
Neoplasms	54,415,394 (50,247,614 to 58,721,812)	2	67,519,830 (1,418,891 to 3,248,407)	2	1.13 (0.95 to 1.31)*	0.87 (0.57 to 1.17)*	1.42 (1.20 to 1.65)*
Cardiovascular diseases	67,584,492 (62,771,497 to 73,832,854)	1	91,933,122 (1,135,346 to 3,124,922)	1	1.62 (1.45 to 1.79)*	1.70 (1.40 to 2.00)*	1.42 (1.32 to 1.53)*
Chronic respiratory diseases	28,653,966 (23,770,991 to 30,931,146)	3	22,520,549 (1,719,779 to 3,416,880)	4	-1.28 (-1.50 to -1.06)*	-2.02 (-2.40 to -1.65)*	-0.50 (-0.75 to -0.26)*
Digestive diseases	11,699,071 (10,590,221 to 12,920,126)	12	10,017,880 (1,549,912 to 3,496,363)	12	-0.80 (-0.87 to -0.73)*	-1.02 (-1.13 to -0.90)*	-0.51 (-0.60 to -0.43)*
Neurological disorders	12,506,303 (6,994,212 to 20,592,252)	11	17,381,406 (1,666,405 to 3,082,962)	7	1.75 (1.67 to 1.82)*	1.79 (1.70 to 1.89)*	1.72 (1.61 to 1.84)*
Mental disorders	18,110,029 (13,449,072 to 23,810,039)	9	20,293,827 (1,145,615 to 3,345,133)	5	0.59 (0.49 to 0.69)*	0.45 (0.40 to 0.49)*	0.75 (0.54 to 0.95)*
Musculoskeletal disorders	20,089,552 (14,362,360 to 26,771,117)	7	29,933,924 (1,427,199 to 3,823,644)	3	2.10 (2.04 to 2.16)*	2.44 (2.36 to 2.52)*	1.75 (1.67 to 1.83)*
Other non-communicable diseases	24,572,991 (20,333,568 to 29,891,261)	4	19,126,072 (1,446,224 to 3,590,726)	6	-1.31 (-1.48 to -1.13)*	-2.43 (-2.53 to -2.33)*	-0.04 (-0.40 to -032)
Skin and subcutaneous diseases	7,809,025 (5,108,660 to 11,567,830)	16	8,264,702 (1,394,816 to 3,356,265)	13	0.29 (0.27 to 0.32)*	0.33 (0.29 to 0.38)*	0.28 (0.26 to 0.29)*
Sense organ diseases	10,740,782 (7,311,356 to 15,203,366)	14	15,981,667 (1,788,420 to 3,997,480)	9	2.14 (1.98 to 2.30)*	1.98 (1.94 to 2.03)*	2.31 (1.97 to 2.64)*
Transport injuries	16,631,845 (15,221,113 to 18,940,342)	10	13,745,455 (1,719,684 to 3,768,996)	11	-1.02 (-1.35 to -0.69)*	0.57 (0.21 to 0.95)*	-2.66 (-3.22 to -2.10)*
Unintentional injuries	21,114,052 (19,362,287 to 23,062,953)	6	16,003,462 (1,056,699 to 3,613,615)	8	-1.53 (-2.01 to -1.04)*	-1.53 (-2.01 to -1.04)*	-1.53 (-2.01 to -1.04)*
Self-harm and interpersonal violence	11,171,276 (9,775,023 to 12,180,298)	13	5,870,049 (1,030,733 to 3,924,034)	16	-3.35 (-3.70 to -3.01)*	-4.09 (-4.73 to -3.45)*	-2.47 (-2.75 to -2.18)*
HIV/AIDS and sexually transmitted infections	921,444,444 (727,466,466 to 1,222,963)	22	1,752,419 (1,430, 192 to 2,114,213)	19	3.47 (2.72 to 4.22)*	3.07 (2.38 to 3.77)*	4.07 (2.71 to 5.45)*
Respiratory infections and tuberculosis	21,938,222 (20,220,847 to 23,581,022)	5	6,647,872 (1,853,547 to 3,613,257)	15	-6.10 (-6.41 to -5.79)*	-8.88 (-9.36 to -8.40)*	-3.34 (-3.65 to -3.03)*
Enteric infections	3,407,267 (2,990,655 to 3,948,769)	20	1,408,098 (1,047,418 to 3,827,650)	20	-4.41 (-5.28 to -3.54)*	-9.05 (-9.96 to -8.13)*	0.68 (-0.56 to 1.92)
Other infectious diseases	4,581,989 (3,724,438 to 6,219,671)	18	1,244,099 (1,063,407 to 3,462,432)	21	-6.57 (-6.95 to -6.19)*	-9.03 (-9.45 to -8.61)*	-4.04 (-4.71 to -3.36)*
Maternal and neonatal disorders	18,110,589 (16,543,873 to 19,663,056)	8	7,151,571 (1,273,977 to 3,174,922)	14	-4.82 (-5.40 to -4.23)*	-6.06 (-6.60 to -5.51)*	-3.56 (-4.60 to -2.51)*
Substance use disorders	6,291,914 (5,043,188 to 7,751,372)	17	5,755,658 (1,373,805 to 3,343,432)	17	-0.39 (-0.82 to 0.03)	-1.74 (-2.49 to -0.99)*	0.94 (0.53 to 1.36)*
Diabetes and kidney diseases	10,436,824 (9,083,252 to 11,910,785)	15	15,909,173 (1,346,885 to 3,824,247)	10	2.30 (2.09 to 2.51)*	2.87 (2.44 to 3.31)*	1.79 (1.70 to 1.89)*
**Age-standardized rate**							
Neglected tropical diseases and malaria	132 (89 to 194)	21	68 (42 to 107)	22	-3.40 (-3.66 to -3.13)*	-4.75 (-5.06 to -4.45)*	-1.97 (-2.42 to -1.51)*
Nutritional deficiencies	369 (284 to 480)	20	175 (122 to 241)	18	-3.88 (-4.07 to -3.69)*	-5.98 (-6.27 to -5.70)*	-1.84 (-2.11 to -1.58)*
Neoplasms	4,505 (4,163 to 4,847)	2	3,421 (2,933 to 3,946)	2	-1.46 (-1.71 to -1.21)*	-1.62 (-1.88 to -1.36)*	-1.25 (-1.68 to -1.81)*
Cardiovascular diseases	6,441 (5,997 to 7,005)	1	4,938 (4,318 to 5,577)	1	-1.40 (-1.56 to -1.24)*	-1.10 (-1.39 to -0.82)*	-1.84 (-1.94 to -1.75)*
Chronic respiratory diseases	2,947 (2,458 to 3,175)	3	1,270 (1,114 to 1,474)	5	-4.34 (-4.61 to -4.07)*	-4.90 (-5.37 to -4.44)*	-3.74 (-4.04 to -3.45)*
Digestive diseases	974 (889 to 1,070)	12	539 (463 to 616)	14	-3.07 (-3.14 to -3.00)*	-3.29 (-3.41 to -3.16)*	-2.77 (-2.84 to -2.71)*
Neurological disorders	1,103 (650 to 1,773)	11	1,077 (598 to 1,780)	8	-0.15 (-0.24 to -0.06)*	-0.09 (-0.16 to -0.02)*	-0.22 (-0.40 to -0.03)*
Mental disorders	1,317 (978 to 1,727)	9	1,248 (922 to 1,627)	6	-0.27 (-0.34 to -0.20)*	-0.56 (-0.59 to -0.53)*	0.01 (-0.13 to 0.14)

For peer review only

For peer review only - http://bmjopen.bmj.com/site/about/guidelines.xhtml

Musculoskeletal disorders	1,536 (1,102 to 2,050)	8	1,585 (1,140 to 2,112)	3	0.16 (0.10 to 0.23)*	0.31 (0.26 to 0.36)*	0.02 (-0.11 to 0.15)
Other non-communicable diseases	2,315 (1,963 to 2,719)	5	1,349 (1,066 to 1,726)	4	-2.81 (-2.96 to -2.67)*	-3.49 (-3.65 to -3.34)*	-2.00 (-2.25 to -1.75)*
Skin and subcutaneous diseases	606 (397 to 898)	16	606 (391 to 909)	13	0.00 (-0.01 to 0.01)	-0.05 (-0.07 to -0.04)*	0.06 (0.03 to 0.08)*
Sense organ diseases	926 (633 to 1,299)	13	863 (591 to 1,226)	9	-0.36 (-0.45 to -0.27)*	-0.46 (-0.49 to -0.43)*	-0.25 (-0.43 to -0.07)*
Transport injuries	1,248 (1,146 to 1,433)	10	860 (740 to 975)	10	-1.98 (-2.42 to -1.54)*	-0.63 (-1.14 to -0.13)*	-3.39 (-4.12 to -2.65)*
Unintentional injuries	1,886 (1,744 to 2,045)	7	1,102 (919 to 1,254)	7	-2.81 (-3.24 to -2.39)*	-2.81 (-3.24 to -2.39)*	-2.81 (-3.24 to -2.39)*
Self-harm and interpersonal violence	807 (708 to 877)	15	361 (310 to 421)	16	-4.19 (-4.62 to -3.76)*	-5.13 (-5.93 to -4.32)*	-3.08 (-3.43 to -2.73)*
HIV/AIDS and sexually transmitted infections	78 (56 to 114)	22	116 (92 to 147)	19	2.02 (1.50 to 2.53)*	2.61 (2.31 to 2.91)*	1.88 (1.05 to 2.72)*
Respiratory infections and tuberculosis	2,396 (2,209 to 2,582)	4	518 (458 to 593)	15	-7.77 (-7.93 to -7.61)*	-9.56 (-9.70 to -9.41)*	-5.88 (-6.18 to -5.58)*
Enteric infections	369 (326 to 422)	19	111 (84 to 144)	20	-6.04 (-6.52 to -5.56)*	-10.47 (-11.00 to -9.94)*	-1.52 (-2.31 to -0.73)*
Other infectious diseases	473 (376 to 659)	17	110 (92 to 131)	21	-7.37 (-7.84 to -6.91)*	-8.51 (-9.21 to -7.80)*	-6.04 (-6.58 to -5.50)*
Maternal and neonatal disorders	2,177 (1,983 to 2,375)	6	792 (699 to 897)	12	-5.26 (-5.36 to -5.16)*	-5.26 (-5.36 to -5.16)*	-5.26 (-5.36 to -5.16)*
Substance use disorders	428 (345 to 526)	18	347 (262 to 443)	17	-1.03 (-1.38 to -0.69)*	-2.60 (-3.02 to -2.18)*	0.85 (0.35 to 1.36)*
Diabetes and kidney diseases	876 (764 to 997)	14	817 (688 to 961)	11	-0.32 (-0.47 to -0.16)*	0.46 (0.17 to 0.75)*	-1.01 (-1.12 to -0.90)*

Note: AAPC: average annual percent changes; UI, uncertainty interval; CI, confidence interval.
*AAPC significantly differs from zero at the alpha = 0.05 level.

For peer review only

sTable 6. AAPC of risk factors for 2000–2019

Risk Factors	DALY (95% UI)				AAPC (95% CI)		
	2000	Rank	2019	Rank	2000–2019	2000–2009	2010–2019
**All-age numbers**							
Unsafe water, sanitation, and handwashing	4,234,714 (3,363,558 to 5,211,019)	15	1,152,504 (805,846 to 1,554,455)	18	-6.60 (-7.28 to -5.91)*	-10.80 (-11.04 to -10.55)*	-2.34 (-3.77 to -0.88)*
Air pollution	49,335,959 (43,328,149 to 55,138,636)	2	42,509,703 (36,337,918 to 49,482,918)	4	-0.78 (-0.98 to -0.58)*	-1.17 (-1.53 to -0.82)*	-0.46 (-0.68 to -0.24)*
Other environmental risks	5,386,702 (3,749,375 to 7,246,194)	14	6,306,070 (4,075,171 to 8,773,453)	13	0.85 (0.72 to 0.98)*	1.14 (0.91 to 1.38)*	0.46 (0.38 to 0.53)*
Child and maternal malnutrition	27,730,286 (24,920,872 to 30,776,034)	5	7,253,783 (6,277,469 to 8,340,678)	12	-6.81 (-7.62 to -5.99)*	-8.92 (-9.38 to -8.47)*	-4.75 (-6.37 to -3.10)*
Tobacco	57,264,585 (52,395,093 to 62,486,440)	1	64,116,000 (54,575,903 to 75,181,983)	1	0.60 (0.42 to 0.78)*	0.30 (0.02 to 0.58)*	0.90 (0.66 to 1.15)*
Alcohol use	14,673,585 (12,536,787 to 17,097,523)	8	17,265,060 (13,765,337 to 21,321,000)	8	0.77 (0.68 to 0.85)*	0.77 (0.68 to 0.85)*	0.77 (0.68 to 0.85)*
Drug use	6,301,278 (5,433,686 to 7,296,963)	13	5,121,794 (4,187,484 to 6,121,198)	14	-1.03 (-1.22 to -0.84)*	-3.28 (-3.50 to -3.07)*	1.54 (1.21 to 1.87)*
High fasting plasma glucose	17,924,145 (14,527,382 to 21,999,850)	7	28,228,439 (22,052,698 to 35,375,022)	5	2.36 (1.82 to 2.90)*	3.64 (2.83 to 4.47)*	1.11 (0.41 to 1.81)*
High systolic blood pressure	35,341,494 (30,227,215 to 40,383,079)	3	54,441,615 (45,474,002 to 63,689,070)	2	2.32 (2.18 to 2.46)*	2.66 (2.40 to 2.91)*	1.84 (1.77 to 1.92)*
High body-mass index	11,704,488 (3,880,854 to 22,527,461)	9	24,830,041 (11,788,976 to 40,545,899)	6	4.02 (3.74 to 4.29)*	4.33 (3.83 to 4.83)*	3.59 (3.48 to 3.70)*
Low bone mineral density	1,769,378 (1,477,619 to 2,061,186)	16	3,320,275 (2,594,480 to 4,004,265)	15	3.39 (3.12 to 3.67)*	4.14 (3.73 to 4.56)*	2.78 (2.42 to 3.15)*
Dietary risks	34,253,322 (27,427,086 to 43,429,953)	4	46,813,126 (35,644,728 to 60,011,858)	3	1.66 (1.53 to 1.78)*	1.97 (1.74 to 2.21)*	1.28 (1.21 to 1.35)*
Low physical activity	1,275,222 (632,556 to 2,484,126)	18	2,507,120 (1,195,139 to 4,842,346)	17	3.54 (2.70 to 4.38)*	3.88 (2.39 to 5.39)*	3.03 (2.65 to 3.41)*
Occupational risks	18,571,331 (16,040,002 to 21,379,719)	6	15,486,849 (12,897,016 to 18,259,216)	9	-0.97 (-1.12 to -0.82)*	-1.51 (-1.78 to -1.24)*	-0.40 (-0.52 to -0.29)*
Intimate partner violence	789,841 (433,187 to 1,234,095)	19	789,926,926 (354,381 to 1,304,433)	19	0.07 (-0.17 to 0.30)	-0.50 (-0.78 to -0.22)*	0.76 (0.39 to 1.14)*
Unsafe sex	1,482,696 (1,293,280 to 1,925,395)	17	2,724,797 (1,976,323 to 3,277,196)	16	3.20 (2.90 to 3.49)*	3.72 (3.50 to 3.94)*	2.95 (2.39 to 3.51)*
Non-optimal temperature	8,427,319 (7,191,510 to 9,779,073)	12	9,325,364 (7,641,727 to 11,224,758)	11	0.44 (-0.88 to 1.77)	-0.61 (-1.39 to 0.18)	1.14 (-1.01 to 3.34)
Kidney dysfunction	8,884,203 (7,881,193 to 10,012,008)	11	13,353,909 (11,150,932 to 15,771,793)	10	2.25 (1.83 to 2.67)*	3.04 (2.37 to 3.71)*	1.47 (0.91 to 2.03)*
High LDL cholesterol	11,217,788 (9,135,992 to 13,780,576)	10	19,813,962 (15,205,317 to 25,139,359)	7	3.11 (2.85 to 3.36)*	3.86 (3.39 to 4.33)*	2.23 (2.08 to 2.37)*
Childhood sexual abuse and bullying	762,744 (311,431 to 1,443,447)	20	646,857,857 (278,772 to 1,183,126)	20	-0.86 (-0.97 to -0.75)*	-1.17 (-1.29 to -1.04)*	-0.40 (-0.58 to -0.22)*
**Age-standardized rate**							
Unsafe water, sanitation, and handwashing	475 (376 to 587)	14	91 (64 to 122)	18	-8.23 (-8.79 to -7.67)*	-11.69 (-12.52 to -10.85)*	-4.58 (-5.22 to -3.93)*
Air pollution	4,860 (4,243 to 5,438)	2	2,265 (1,950 to 2,615)	4	-3.93 (-4.15 to -3.70)*	-4.08 (-4.49 to -3.68)*	-3.86 (-3.97 to -3.74)*
Other environmental risks	488 (338 to 653)	13	331 (213 to 460)	13	-2.00 (-2.13 to -1.87)*	-1.49 (-1.73 to -1.25)*	-2.64 (-2.71 to -2.56)*
Child and maternal malnutrition	3,285 (2,955 to 3,649)	4	786 (690 to 899)	10	-7.24 (-7.44 to -7.04)*	-8.34 (-8.58 to -8.10)*	-6.13 (-6.41 to -5.85)*
Tobacco	5,287 (4,858 to 5,765)	1	3,228 (2,759 to 3,767)	1	-2.57 (-2.83 to -2.32)*	-2.80 (-3.20 to -2.40)*	-2.33 (-2.68 to -1.98)*
Alcohol use	1,137 (972 to 1,328)	8	908 (729 to 1,116)	8	-1.18 (-1.50 to -0.86)*	-1.41 (-1.54 to -1.28)*	-0.92 (-1.56 to -0.27)*
Drug use	449 (390 to 518)	15	295 (238 to 361)	14	-2.11 (-2.74 to -1.48)*	-4.70 (-4.97 to -4.43)*	0.85 (-0.48 to 2.19)
High fasting plasma glucose	1,647 (1,338 to 2,051)	6	1,452 (1,139 to 1,832)	5	-0.72 (-1.23 to -0.20)*	0.72 (-0.06 to 1.51)	-2.11 (-2.76 to -1.44)*
High systolic blood pressure	3,304 (2,820 to 3,777)	3	2,844 (2,392 to 3,321)	2	-0.77 (-0.89 to -0.65)*	-0.29 (-0.51 to -0.07)*	-1.40 (-1.47 to -1.34)*
High body-mass index	962 (315 to 1,891)	10	1,231 (578 to 2,023)	6	1.28 (0.95 to 1.60)*	1.50 (0.91 to 2.10)*	0.94 (0.82 to 1.07)*

For peer review only - http://bmjopen.bmj.com/site/about/guidelines.xhtml

Low bone mineral density	164 (137 to 192)	16	177 (138 to 214)	15	0.40 (0.27 to 0.54)*	0.70 (0.55 to 0.86)*	0.20 (-0.02 to 0.42)
Dietary risks	3,022 (2,391 to 3,875)	5	2,394 (1,823 to 3,071)	3	-1.23 (-1.36 to -1.09)*	-0.78 (-1.03 to -0.52)*	-1.75 (-1.83 to -1.68)*
Low physical activity	149 (74 to 287)	17	146 (70 to 280)	16	-0.19 (-0.74 to 0.36)	0.48 (0.17 to 0.79)*	-0.93 (-2.03 to 0.18)
Occupational risks	1,482 (1,277 to 1,700)	7	844 (701 to 997)	9	-2.95 (-3.08 to -2.81)*	-3.67 (-3.88 to -3.45)*	-2.22 (-2.37 to -2.07)*
Intimate partner violence	55 (30 to 86)	19	46 (22 to 76)	20	-0.86 (-1.11 to -0.62)*	-1.68 (-1.94 to -1.41)*	0.09 (-0.32 to 0.50)
Unsafe sex	108 (94 to 141)	18	142 (106 to 170)	17	1.47 (0.88 to 2.06)*	1.83 (1.43 to 2.23)*	1.34 (0.19 to 2.51)*
Non-optimal temperature	936 (813 to 1,075)	11	483 (399 to 582)	12	-3.61 (-4.04 to -3.18)*	-4.39 (-5.06 to -3.71)*	-2.91 (-3.41 to -2.42)*
Kidney dysfunction	781 (690 to 882)	12	709 (593 to 832)	11	-0.45 (-0.97 to 0.08)	054 (-0.31 to 1.39)	-1.41 (-2.10 to -0.72)*
High LDL cholesterol	1,005 (790 to 1,284)	9	1,052 (800 to 1,345)	7	0.28 (0.10 to 0.45)*	1.19 (0.87 to 1.51)*	-0.75 (-0.85 to -0.66)*
Childhood sexual abuse and bullying	54 (22 to 102)	20	50 (20 to 91)	19	-0.41 (-0.51 to -0.31)*	-1.05 (-1.21 to -0.89)*	0.40 (0.31 to 0.48)*

Note: AAPC: average annual percent changes; UI, uncertainty interval; CI, confidence interval; LDL, low-density lipoprotein.
*AAPC significantly differs from zero at the alpha = 0.05 level.

For peer review only

BMJ Open

Temporal trend of population structure, burden of diseases, healthcare resources and expenditure in China, 2000-2019

Journal:	*BMJ Open*
Manuscript ID	bmjopen-2022-062091.R1
Article Type:	Original research
Date Submitted by the Author:	15-Jun-2022

Complete List of Authors:	Liang, Zhenguo; Third Affiliated Hospital of Sun Yat-Sen University, Department of Rheumatology<br>WU, Dongze; Third Affiliated Hospital of Sun Yat-Sen University, Department of Rheumatology<br>Guo, Cui; The Chinese University of Hong Kong<br>Gu, Jieruo; Third Affiliated Hospital of Sun Yat-Sen University, Department of Rheumatology

**Primary Subject Heading**:	Health policy
Secondary Subject Heading:	Global health, Health policy
Keywords:	Health economics < HEALTH SERVICES ADMINISTRATION & MANAGEMENT, Health policy < HEALTH SERVICES ADMINISTRATION & MANAGEMENT, Health & safety < HEALTH SERVICES ADMINISTRATION & MANAGEMENT

SCHOLARONE™
Manuscripts

I, the Submitting Author has the right to grant and does grant on behalf of all authors of the Work (as defined in the below author licence), an exclusive licence and/or a non-exclusive licence for contributions from authors who are: i) UK Crown employees; ii) where BMJ has agreed a CC-BY licence shall apply, and/or iii) in accordance with the terms applicable for US Federal Government officers or employees acting as part of their official duties; on a worldwide, perpetual, irrevocable, royalty-free basis to BMJ Publishing Group Ltd ("BMJ") its licensees and where the relevant Journal is co-owned by BMJ to the co-owners of the Journal, to publish the Work in this journal and any other BMJ products and to exploit all rights, as set out in our licence.

The Submitting Author accepts and understands that any supply made under these terms is made by BMJ to the Submitting Author unless you are acting as an employee on behalf of your employer or a postgraduate student of an affiliated institution which is paying any applicable article publishing charge ("APC") for Open Access articles. Where the Submitting Author wishes to make the Work available on an Open Access basis (and intends to pay the relevant APC), the terms of reuse of such Open Access shall be governed by a Creative Commons licence – details of these licences and which Creative Commons licence will apply to this Work are set out in our licence referred to above.

Other than as permitted in any relevant BMJ Author's Self Archiving Policies, I confirm this Work has not been accepted for publication elsewhere, is not being considered for publication elsewhere and does not duplicate material already published. I confirm all authors consent to publication of this Work and authorise the granting of this licence.

For peer review only

Temporal trend of population structure, burden of diseases, healthcare resources and expenditure in China, 2000-2019

Authors

Zhenguo Liang[1#], Dongze Wu, PhD[1#], Cui Guo[2#], PhD, Jieruo Gu, PhD[1*]

Institution

1.Department of Rheumatology, The Third Affiliated Hospital of Sun Yat-sen University, Guangzhou, China

2.Jockey Club School of Public Health and Primary Care, The Chinese University of Hong Kong, Hong Kong SAR, China

[#]Contributed equally to the manuscript.

Corresponding authors

[*]Corresponding to Prof. Jieruo Gu.

Prof. Jieguo Gu, PhD

Address: No. 600 Tianhe Road, Tianhe District, Guangzhou, Guangdong, China, 510000

E-mail: gujieruo@mail.sysu.edu.cn

Telephone: (+86) 2085253333 / Fax number: (+86) 2085253336

Running Head: Population ageing in China

Keywords: Trend, Population, Heath Care, Disease Burden, China

Word Count: 3581 **Reference:40** **Table Count: 4** **Figure Count:1**

For peer review only - http://bmjopen.bmj.com/site/about/guidelines.xhtml

Abstract

Objectives

To explore the trend of disease burden evolution from young to old population in China, and to provide guidance and suggestions for areas requiring intervention.

Design

A cross-sectional and longitudinal analysis

Setting

Population and healthcare data from China Statistical Yearbook (CSY), disease burden and risk factor data from the Global Burden of Diseases (GBD) between 2000 and 2019.

Measures and methods

Primary outcomes are trends in population composition, population change, dependency ratio, healthcare institution, personnel, expenditure, disease burden and risk factor from 2000 to 2019. Secondary outcomes are i) the main drivers of disease burden and risk factor in individuals younger and older than 70, ii) temporal trend in population, healthcare, and disease burden from 2000 to 2009, and from 2010 to 2019. Average annual percent change (AAPC) with 95% confidence interval (CI) was quantified by using the Joinpoint Regression Program.

Results

Regarding the population in China between 2000 and 2019, a decreasing trend was observed among youth aged 0–14 years (AAPC: -1.17), a slow rising trend was observed among individuals aged 15–64 years (AAPC: 1.10), and a rapidly increasing trend was observed among individuals older than 65 years (AAPC: 3.67). Astonishing increasing trends in healthcare institutions (AAPC: 3.97), medical personnel (AAPC: 3.26), and healthcare expenditures (AAPC: 15.28) were also observed. Among individuals younger than 70 years, neoplasms (AAPC: 0.54) and cardiovascular

For peer review only - http://bmjopen.bmj.com/site/about/guidelines.xhtml

diseases (AAPC: 0.67) remained among the top three causes, while tobacco (AAPC: 0.22) remained a top three risk factor. However, while musculoskeletal disorders (AAPC: 1.88) were not a top three cause in 2000, they are a top three cause in 2019.

Conclusion

Comprehensive age-regional-specific strategies that target younger populations are urgently needed to reduce disease burden. The disease burden from metabolic risk factors, neoplasms and musculoskeletal disorders were identified as key domains.

Keywords: Trend, Population, Heath Care, Disease Burden, China

Strengths and limitations of this study

- This study characterized the temporal trend of population structure and composition, health resources and expenditures, disease burden and risk factor in China from 2000 to 2019.
- The analysis integrated Global Burden of Disease study and China Statistical Yearbook to assess the disease burden, population structure, health resources and expenditures, respectively.
- Our data identified disease burden from metabolic risk factors, neoplasms and musculoskeletal disorders as key domains that require intervention to reduce an increasing disease burden among individuals currently older than 70 years, as well as those approaching this age group.
- The principal limitation of this study is unavailability of provincial level data in disease burden, population, and healthcare and inability to provide granular policy recommendation.

For peer review only - http://bmjopen.bmj.com/site/about/guidelines.xhtml

Introduction

Together with the world's population, people in China struggle against cancer and aging despite efforts to extend lifespan and maximize healthspan[1, 2]. Since 1950, the lifespan at birth has been extended by 26 years in China, while the total fertility rate has decreased from 5.91 to 1.43[3]. Between 2000 and 2019, the life expectancy for males has increased from 69.1 to 74.4 years, while the life expectancy for females has increased from 73.9 to 80.8 years[3]. It has been forecasted that China will become the largest economy by 2035, and that the population in China will decline by 48.0% from 2017 to 2100[4]. Moreover, in the next 20 years, the universal two-to-three-child policy may not reverse a shrinking workforce and rapid population ageing, especially in rural areas[5, 6]. In 2009, China expanded social health insurance coverage to its entire population and reformed its health care delivery system[7, 8].

Health care systems coordinate complex interrelationships among individuals who receive and provide medical financial support[9]. It would have been difficult for China to rigorously control the two pandemics that occurred in 2003 and 2019 without a healthcare system. In particular, an increasing number of modernized medical institutions and well-educated personnel have been established over the past two decades in China[10, 11]. Accelerating rural-to-urban migration and corresponding increases in urban populations have also both led to improved access to health care, although substantial health risks can accompany changes in human activities, diets, and social structures[12]. Although two GBD studies have assessed the disease burden and risk factor at provincial level in China[13, 14], the study further to evaluate healthcare professional and expenditure and to identify key domain to mitigate the aging driven disease burden. The study also tries to dissect the implication of a central tension among 3 mutually conflicting forces: intractable disease burden, population ageing, and upsurging healthcare investment. Therefore, the objective

For peer review only - http://bmjopen.bmj.com/site/about/guidelines.xhtml

of this study was to quantify trends in population, health care, and disease burden in China between 2000 and 2019. Possible driving forces of disease burden among ageing populations were also examined.

Methods

Data sources

The National Bureau of Statistics (NBS) of China annually publishes the China Statistical Yearbook (CSY)[15]. The Department of Population and Employment Statistics of the NBS conducts statistical population surveys as follows. A national population census is conducted in years ending with 0; a national 1 percent population sample survey is conducted in years ending with 5; and sample surveys on population changes are conducted in the remaining years. The latter covers about 1 per thousand of the total population of China. The sample surveys on population change take the entire nation of China as the population, while each province, autonomous region, or municipality represents a sub-population. A stratified multi-stage systematic probability-proportional-to-size (PPS) cluster sampling scheme is used. Data regarding population, healthcare institutions, personnel, beds, and health expenditures are described in detail in the Supplemental Appendix (including a glossary and definitions).

The Global Burden of Disease（GBD）2019 study assessed life expectancy, disease burden, risk factor, etiology, and impairment[3, 16, 17]. All-cause incidence, prevalence, death, years lived with disability (YLD), years of life lost (YLL), disability-adjusted life years (DALYs), and attributable risk factors were assessed between 1990 and 2019. The study identified twenty-two level 2 leading causes and twenty level 2 leading risk factors. Both causes and risk factors were assessed according to number, rate, and age-standardized rate of DALYs. The estimation methods and data for burden of disease published in GBD 2019 were used to assess disease burden in China

For peer review only - http://bmjopen.bmj.com/site/about/guidelines.xhtml

between 2000 and 2019 for the present study.

Outcomes of measurements

The primary outcomes are temporal trend in population, healthcare, and disease burden from 2000 to 2019, including population composition, population change, dependency ratio, healthcare institution, personnel, expenditure, disease burden and risk factor. The secondary outcomes are i) the main drivers of disease burden and risk factor in individuals younger and older than 70, ii) temporal trend in population, healthcare, and disease burden from 2000 to 2009, and from 2010 to 2019.

Statistical analysis

The population composition, change, dependency ratio, healthcare institution, personnel, and expenditures in terms of number, disease burden and risk factors in terms of number and rate, were assessed by using a Joinpoint regression model. The disease burden and risk factor in individuals younger and older than 70 years in term of number and rate were also assessed to identify the driving cause and risk factors. Average annual percent change (AAPC) and associated 95% confidence intervals (CIs) were calculated for the study period. Temporal increasing and decreasing trends were defined according to the statistical significance of the AAPC compared to zero. AAPC values with 95% CIs overlapping with zero were considered stable. Two segmented line regression with joinpoint of 2000, 2009, and 2019 were calculated to show the temporal trend from 2000 to 2009, from 2010 to 2019. All statistical analyses were performed by using the Joinpoint Regression Program (version 4.8.0.1, Statistical Methodology and Applications Branch, Surveillance Research Program, National Cancer Institute, Bethesda, MD, USA). P-values less than 0.05 were considered statistically significant at a two-tailed level.

For peer review only - http://bmjopen.bmj.com/site/about/guidelines.xhtml

Patient and public involvement statement

Patients and the public were not involved in any way in this research.

Results

Population trends between 2000 and 2019

Initially, temporal trends of the population structure in China were examined based on CSY data (gender and age composition, birth rate, and dependency ratio). From 2000 through 2019, an increasing trend in total population (AAPC: 0.52, 95% CI: 0.51 to 0.54), male population (AAPC: 0.47, 95% CI: 0.44 to 0.51), and female population (AAPC: 0.59, 95% CI: 0.56 to 0.62) were observed. As expected, a striking contrary trend in urban population (AAPC: 3.28, 95%CI: 3.18 to 3.38) and rural population (AAPC: -1.98, 95%CI: -2.02 to -1.95) were observed. We further identified that an increasing trend in urban population was attenuated, while a decreasing trend in rural population was aggravated, during 2010–2019 compared to 2000–2009 (Figure 1A, Table 1).

A clear decreasing trend in the younger population (ages 0–14 years) was observed in 2000–2019 (AAPC: -1.17, 95%CI: -1.78 to -0.55), especially in 2000–2009. An increasing trend in the working population (ages 15–64 years) was observed in 2000–2009 (AAPC: 1.10, 95%CI: 1.03 to 1.18), and then it gradually disappeared over 2010–2019. In contrast, an increasing trend in the post-working population (65+ years) that was observed in 2000–2019 (AAPC: 3.67, 95%CI: 3.51 to 3.82) gradually intensified from 2000–2009 to 2010–2019 (Figure 1B, Table 1).

From 2000 to 2019, the birth rate (AAPC: -1.53, 95%CI: -2.31 to -0.75) and natural growth rate (AAPC: -4.20, 95%CI: -5.52 to -2.87) continued to decrease. Conversely, from 2000 to 2009, the death rate continued to increase (AAPC: 1.10, 95%CI: 0.75 to 1.46), yet from 2010–2019 it remained stable (AAPC: 0.03, 95%CI: -0.06 to 0.13) (Figure 1C, Table 1). The past two decades also exhibited a reverse trend in gross dependency ratio from 2010–2019, a muted decreasing trend

For peer review only - http://bmjopen.bmj.com/site/about/guidelines.xhtml

157 in children dependency ratio from 2000–2009 to 2010–2019, and a strengthened increasing trend
158 in old dependency ratio from 2000–2009 to 2010–2019 (Figure 1D, Table 1).

For peer review only

For peer review only - http://bmjopen.bmj.com/site/about/guidelines.xhtml

Table 1. AAPC in population composition, change, and dependency ratio between 2000 and 2019

Subcategory	Number			AAPC (95% CI)		
	2000	2009	2019	2000–2019	2000–2009	2010–2019
**Population composition by gender and area (10000 persons)**						
Total Population	126,743	133,450	140,005	0.52 (0.51 to 0.54)*	0.57 (0.56 to 0.58)*	0.48 (0.45 to 0.51)*
Male Population	65,437	68,647	71,527	0.47 (0.44 to 0.51)*	0.53 (0.50 to 0.57)*	0.43 (0.37 to 0.49)*
Female Population	61,306	64,803	68,478	0.59 (0.56 to 0.62)*	0.62 (0.56 to 0.67)*	0.56 (0.55 to 0.57)*
Urban Population	45,906	64,512	84,843	3.28 (3.18 to 3.38)*	3.86 (3.75 to 3.98)*	2.68 (2.51 to 2.84)*
Rural Population	80,837	68,938	55,162	-1.98 (-2.02 to -1.95)*	-1.79 (-1.86 to -1.73)*	-2.15 (-2.18 to -2.12)*
**Population composition by age group (10000 persons)**						
Population Aged 0–14 y	29,012	24,659	23,492	-1.17 (-1.78 to -0.55)*	-2.24 (-2.76 to -1.71)*	0.31 (-0.23 to 0.85)
Population Aged 15–64 y	88,910	97,484	98,910	0.59 (0.52 to 0.66)*	1.10 (1.03 to 1.18)*	0.02 (-0.10 to 0.14)
Population Aged 65+ y	8,821	11,307	17,603	3.67 (3.51 to 3.82)*	2.81 (2.66 to 2.96)*	4.51 (4.25 to 4.76)*
**Population change (%)**						
Birth Rate	14.03	11.95	10.48	-1.53 (-2.31 to -0.75)*	-1.63 (-2.32 to -0.93)*	-1.57 (-3.03 to -0.09)*
Death Rate	6.45	7.08	7.14	0.54 (0.37 to 0.71)*	1.10 (0.75 to 1.46)*	0.03 (-0.06 to 0.13)
Natural Growth Rate	7.58	4.87	3.34	-4.20 (-5.52 to -2.87)*	-4.64 (-5.69 to -3.57)*	-4.40 (-6.93 to -1.80)*
**Dependency ratio (%)**						
Gross Dependency Ratio	42.6	36.9	41.5	-0.25 (-0.54 to 0.03)	-1.97 (-2.26 to -1.68)*	1.70 (1.21 to 2.19)*
Children Dependency Ratio	32.6	25.3	23.8	-1.76 (-2.54 to -0.96)*	-3.33 (-4.00 to -2.66)*	0.33 (-0.37 to 1.03)
Old Dependency Ratio	9.9	11.6	17.8	3.12 (2.93 to 3.31)*	1.76 (1.45 to 2.08)*	4.49 (4.27 to 4.70)*

AAPC: average annual percent change; CI, confidence interval. *AAPC significantly differs from zero at a two-tailed level of 0.05.

For peer review only

For peer review only - http://bmjopen.bmj.com/site/about/guidelines.xhtml

161 **Health resources and expenditure between 2000 and 2019**

162 Temporal trends in health sources, including institutions, personnel, and expenditures,
163 were examined based on CSY data. A consistent increasing trend in number of hospitals
164 (AAPC: 3.97, 95%CI: 3.69 to 4.24), including general hospitals, hospitals that specialize
165 in traditional Chinese medicine, and specialized hospitals, was observed. The number of
166 medical personnel (AAPC: 3.26, 95%CI: 2.76 to 3.77), including medical technical
167 personnel, licensed assistant doctors, licensed doctors, and registered nurses, also
168 continued to increase in 2000–2019. Moreover, the rate of increase of number of medical
169 personnel was higher in 2010–2019 than in 2000–2009. The number of beds in health care
170 institutions (AAPC: 5.54, 95%CI: 5.20 to 5.88), including hospitals, township health
171 service centers, specialized public health institutions, and women and children care
172 agencies, also increased in 2000–2019. Like medical personnel, the rate increase for beds
173 in health care institutions was greater in 2010–2019 than in 2000–2009 (Table 2).

174 Total healthcare expenditure increased from ¥458.66 billion (US$ 55.40 billion) in 2000
175 to ¥6584.14 billion (US$ 954.68 billion) in 2019, with an AAPC of 15.28 (95%CI: 14.64
176 to 15.91). The per capita health expenditure increased from ¥361.88 in 2000 to ¥4702.79
177 in 2019, with an AAPC of 14.67 (95%CI: 14.04 to 15.30). Striking increasing trends in
178 government health expenditure (AAPC: 18.95, 95%CI: 16.72 to 21.22), social health
179 expenditure (AAPC: 19.03, 95%CI: 17.56 to 20.52), and out-of-pocket health expenditure
180 (AAPC: 10.62, 95%CI: 9.24 to 12.02) were also observed in 2000–2019 (Table 2).

For peer review only - http://bmjopen.bmj.com/site/about/guidelines.xhtml

Table 2. AAPC in healthcare institution, personnel, and expenditures between 2000 and 2019

Subcategory	Number			AAPC (95% CI)		
	2000	2009	2019	2000—2019	2000—2009	2010—2019
**Health care institution (unit)**						
Number of Hospitals	16,318	20,291	34,354	3.97 (3.69 to 4.24)*	2.41 (2.03 to 2.80)*	5.72 (5.25 to 6.19)*
Number of General Hospitals	11,872	13,364	19,963	2.84 (2.56 to 3.13)*	1.37 (0.97 to 1.77)*	4.51 (4.03 to 4.99)*
Number of Hospitals Specialized in Traditional Chinese Medicine	2,453	2,728	4,221	2.92 (2.50 to 3.34)*	1.35 (1.20 to 1.50)*	4.69 (3.82 to 5.58)*
Number of Specialized Hospitals	1,543	3,716	8,531	9.65 (9.02 to 10.28)*	10.98 (9.69 to 12.28)*	8.46 (8.25 to 8.68)*
Number of Health Care Institutions at Grass-root Level	1,000,169	882,153	954,390	-0.45 (-0.82 to -0.08)*	-1.88 (-2.60 to -1.15)*	0.85 (0.67 to 1.04)*
Number of Township Health Service Centers	49,229	38,475	36,112	-1.63 (-1.83 to -1.44)*	-2.79 (-3.10 to -2.47)*	-0.47 (-0.66 to -0.28)*
Number of Village Clinics	709,458	632,770	616,094	-1.03 (-1.40 to -0.65)*	-1.88 (-2.53 to -1.23)*	-0.51 (-0.86 to -0.17)*
Number of Outpatient Department	240,934	182,448	266,659	0.42 (-0.18 to 1.03)	-2.73 (-3.45 to -2.01)*	4.05 (3.03 to 5.07)*
Number of Specialized Public Health Institutions	11,386	11,665	15,958	1.63 (-0.23 to 3.54)	0.12 (-0.68 to 0.93)	3.35 (-0.52 to 7.36)
Number of Center for Disease Control and Prevention	3,741	3,536	3,403	-0.40 (-0.50 to -0.29)*	-0.40 (-0.50 to -0.29)*	-0.40 (-0.50 to -0.29)*
Number of Specialized Disease Prevention & Treatment Institution	1,839	1,291	1,128	-2.47 (-2.97 to -1.98)*	-3.61 (-4.46 to -2.76)*	-1.49 (-2.05 to -0.93)*
Number of Women and Children Care Agencies	3,163	3,020	3,071	-0.11 (-0.29 to 0.08)	-0.45 (-0.80 to -0.10)*	0.20 (0.09 to 0.32)*
**Medical Personnel (10000 persons)**						
Number of Medical Personnel	691.04	778.14	1292.83	3.26 (2.76 to 3.77)*	1.15 (0.16 to 2.14)*	5.16 (4.87 to 5.45)*
Number of Medical Technical Personnel	449.08	553.51	1015.40	4.34 (3.99 to 4.68)*	2.30 (1.61 to 3.01)*	6.20 (6.07 to 6.34)*
Number of Licensed (Assistant) Doctors	207.58	232.92	386.69	3.12 (2.64 to 3.59)*	0.92 (0.05 to 1.81)*	5.36 (4.98 to 5.74)*
Number of Licensed Doctors	160.33	190.54	321.05	3.53 (3.12 to 3.95)*	1.50 (0.73 to 2.27)*	5.62 (5.29 to 5.95)*
Number of Registered Nurse	126.68	185.48	444.50	6.79 (6.20 to 7.38)*	4.37 (3.26 to 5.49)*	8.92 (8.49 to 9.34)*
Number of Pharmacist	41.44	34.19	48.34	0.76 (0.23 to 1.29)*	-2.28 (3.31 to -1.23)*	3.57 (3.27 to 3.88)*
Number of Village Doctors and Assistants	131.94	105.10	84.23	-2.49 (-4.28 to -0.66)*	-3.44 (-6.25 to -0.55)*	-2.16 (-4.10 to -0.17)*
Number of Other Technical Personnel	15.75	27.50	50.39	6.30 (6.07 to 6.53)*	6.30 (6.07 to 6.53)*	6.30 (6.07 to 6.53)*
Number of Administrative Personnel	42.68	36.27	54.38	1.25 (0.42 to 2.09)*	-1.74 (-3.27 to -0.18)*	4.01 (3.49 to 4.54)*
Number of Logistics Technical Workers	51.59	55.77	88.43	2.95 (2.13 to 3.78)*	0.84 (-0.26 to 1.94)	4.71 (3.57 to 5.86)*
**Beds in Health Care Institutions**						
Number of Beds in Health Care Institutions (10000 units)	317.70	441.66	880.70	5.54 (5.20 to 5.88)*	3.60 (3.01 to 4.20)*	7.11 (6.82 to 7.41)*
Number of Beds in Hospitals (10000 beds)	216.67	312.08	686.65	6.35 (5.96 to 6.74)*	4.17 (3.55 to 4.81)*	8.16 (7.74 to 8.59)*
Number of Beds in Community Health Service Centers (10000 units)	76.65	109.98	163.11	3.96 (2.90 to 5.04)*	3.73 (1.68 to 5.83)*	3.28 (2.75 to 3.82)*
Number of Beds in Township Health Service Centers (10000 beds)	73.48	93.34	136.99	3.29 (2.18 to 4.41)*	2.38 (0.26 to 4.54)*	3.48 (2.93 to 4.04)*

For peer review only - http://bmjopen.bmj.com/site/about/guidelines.xhtml

Number of Beds in Specialized Public Health Institutions (10000 units)	11.86	15.40	28.50	4.80 (4.31 to 5.28)*	2.97 (2.52 to 3.42)*	6.24 (5.55 to 6.93)*
Number of Beds in Women and Children Care Agencies (10000 units)	7.12	12.61	24.32	6.79 (6.41 to 7.18)*	6.65 (6.15 to 7.15)*	6.74 (6.25 to 7.24)*
Number of Beds in Specialized Disease Prevention & Treatment Institution (10000 beds)	2.84	2.71	4.11	2.29 (0.21 to 4.41)*	0.19 (-3.08 to 3.57)	3.75 (1.61 to 5.95)*
**Health Expenditure (100 million yuan)**						
Total Health Expenditure	4586.63	17541.92	65841.39	15.28 (14.64 to 15.91)*	15.99 (15.09 to 16.90)*	14.04 (13.44 to 14.63)*
Government Health Expenditure	709.52	4816.26	18016.95	18.95 (16.72 to 21.22)*	24.39 (20.91 to 27.97)*	13.52 (11.30 to 15.79)*
Social Health Expenditure	1171.94	6154.49	29150.57	19.03 (17.56 to 20.52)*	21.13 (18.16 to 24.18)*	17.17 (16.18 to 18.16)*
Out-of-pocket Health Expenditure	2705.17	6571.16	18673.87	10.62 (9.24 to 12.02)*	9.82 (9.21 to 10.44)*	10.96 (8.48 to 13.48)*
Per Capita Health Expenditure (yuan)	361.88	1314.26	4702.79	14.67 (14.04 to 15.30)*	15.34 (14.45 to 16.24)*	13.47 (12.89 to 14.06)*

AAPC: average annual percent change; CI, confidence interval. *AAPC significantly differs from zero at a two-tailed level of 0.05.

For peer review only

For peer review only - http://bmjopen.bmj.com/site/about/guidelines.xhtml

183 **Population health, disease burden and risk factors between 2000 and 2019**

184 According to data from GBD 2019, life expectancy at birth and at 60–64 years were
185 estimated to increase (AAPC: 0.44, 95%CI: 0.43 to 0.46; AAPC: 0.76, 95%CI: 0.60 to 0.91,
186 respectively). In addition, the percentage of healthy life expectancy within total life
187 expectancy at birth and at 60–64 years decreased from 88.90 to 88.32, and from 77.12 to
188 76.36, respectively (sFigure 1, sTable 1–3). Overall, the number of all-cause DALYs was
189 stable during 2000–2019 (AAPC: 0.08, 95%CI -0.06 to 0.22). However, it decreased
190 between 2000 and 2009 (AAPC: -0.44, 95%CI: -0.58 to -0.31), and it increased between
191 2010 and 2019 (AAPC: 0.60, 95% CI: 0.39 to 0.82) (sTable 4).

192 In 2000, the top three causes of DALYs were cardiovascular diseases, neoplasms, and
193 chronic respiratory diseases. In 2019, they were cardiovascular diseases, neoplasms, and
194 musculoskeletal disorders. From 2000–2019, the top three drivers of increasing number of
195 DALYs were HIV/AIDS and sexually transmitted infections (AAPC: 3.47, 95%CI: 2.72
196 to 4.22), diabetes and kidney diseases (AAPC: 2.30, 95%CI: 2.09 to 2.51), and sense organ
197 diseases (AAPC: 2.14, 95%CI: 1.98 to 2.30). The top two drivers of increasing age-
198 standardized rate of DALYs were HIV/AIDS and sexually transmitted infections (AAPC:
199 2.02, 95%CI: 1.50 to 2.53) and musculoskeletal disorders (AAPC: 0.16, 95%CI: 0.10 to
200 0.23) (sTable 5).

201 In 2000, the top three risk factors were tobacco, air pollution, and high systolic blood
202 pressure. In 2019, they were tobacco, high systolic blood pressure, and dietary risks.
203 Between 2000 and 2019, the top three leading risk factors with the largest increasing
204 number of DALYs were high body-mass index (AAPC: 4.02, 95%CI: 3.74 to 4.29), low
205 physical activity (AAPC: 3.54, 95%CI: 2.70 to 4.38), and low bone mineral density (AAPC:

For peer review only - http://bmjopen.bmj.com/site/about/guidelines.xhtml

3.39, 95%CI: 3.12 to 3.67). The top three leading factors with the largest increase in age-standardized rate of DALYs were unsafe sex (AAPC: 1.47, 95% CI: 0.88 to 2.06), high body-mass index (AAPC: 1.28, 95%CI: 0.95 to 1.60), and low bone mineral density (AAPC: 0.40, 95%CI: 0.27 to 0.54) (sTable 6).

Trends in disease burden between younger individuals and those older than 70 years

Finally, trends in causes and risk factors for the population with a cut-off point of 70 years were examined for 2000–2019. Among individuals younger than 70 years, musculoskeletal disorders replaced other non-communicable diseases as one of the top three causes from 2000 to 2019. In contrast, the top three causes remained the same among individuals older than 70 years, and included cardiovascular diseases, neoplasms, and chronic respiratory disease (Table 3). Among the population younger than 70 years, the causes with the largest increment in number of DALYs were HIV/AIDS and sexually transmitted infections (AAPC: 3.33, 95%CI: 2.58 to 4.08), musculoskeletal disorders (AAPC: 1.88, 95%CI: 1.84 to 1.92), and sense organ diseases (AAPC: 1.71, 95%CI: 1.43 to 2.00). In the same population, the largest increments in rate of DALYs were HIV/AIDS and sexually transmitted infections (AAPC: 3.42, 95%CI: 2.76 to 4.09), musculoskeletal disorders (AAPC: 2.04, 95%CI: 2.00 to 2.08), and diabetes and kidney diseases (AAPC: 1.88, 95%CI: 1.58 to 2.18). In comparison, the causes among individuals older than 70 years that had the largest increment in both number and rate of DALYs were maternal and neonatal disorders (AAPC: 10.64, 95%CI: 10.47 to 10.82; AAPC: 6.84, 95%CI: 6.69 to 6.98, respectively), HIV/AIDS and sexually transmitted infections (AAPC: 8.17, 95%CI: 6.93 to 9.43;AAPC:4.39, 95%CI: 3.26 to 3.53, respectively), and unintentional injuries (AAPC:4.66, 95%CI: 3.90 to 5.43; AAPC: 1.01, 95%CI: 0.46 to 1.56, respectively) (Table

For peer review only - http://bmjopen.bmj.com/site/about/guidelines.xhtml

3).

Among the population younger than 70 years, dietary risks (AAPC: 1.02, 95%CI: 0.85 to 1.19) and high systolic blood pressure (AAPC: 1.55, 95%CI: 1.29 to 1.81) replaced air pollution and child and maternal malnutrition to enter the top three risk factors for 2000–2019. In contrast, the top three risk factors remained the same between 2000 and 2019 for the population older than 70 years, and they include: high systolic blood pressure, tobacco, and air pollution (Table 3). Regarding risk factors among the population younger than 70 years, the largest increments in number and rate of DALYs were high body-mass index (AAPC: 3.70, 95%CI: 3.59 to 3.82; AAPC: 3.88, 95%CI: 3.69 to 4.07, respectively), unsafe sex (AAPC: 3.11, 95%CI: 2.80 to 3.42; AAPC: 3.28, 95%CI: 2.96 to 3.60, respectively), and low physical activity (AAPC: 2.75, 95%CI: 2.34 to 3.15; AAPC: 2.91, 95%CI: 2.46 to 3.36, respectively). In contrast, the risk factors with the largest increment in number of DALYs among those older than 70 years were high body-mass index (AAPC: 5.22, 95%CI: 4.79 to 5.65), low bone mineral density (AAPC: 5.22, 95%CI: 4.98 to 5.46), and high LDL cholesterol (AAPC: 5.02, 95%CI: 4.62 to 5.41). Furthermore, the same risk factors with different rankings were observed in terms of rate of DALYs for the same population: low bone mineral density (AAPC: 1.62, 95%CI: 1.38 to 1.86), high body-mass index (AAPC: 1.50, 95%CI: 1.01 to 2.00), and high LDL cholesterol (AAPC: 1.44, 95%CI: 1.04 to 1.84), respectively (Table 4).

For peer review only - http://bmjopen.bmj.com/site/about/guidelines.xhtml

Table 3. The AAPC of 22 causes in people younger and older than 70 years of age presented in DALYs (95% UI)

Causes	Age < 70 years			Age ≥ 70 years		
	2000	2019	AAPC (95% CI)	2000	2019	AAPC (95% CI)
Number						
Neglected tropical diseases and malaria	1,566,614 (1,022,581 to 2,343,199)	977,312 (586,629 to 1,582,219)	-2.42 (-2.73 to -2.10)*	137,451 (93,607 to 195,272)	160,494 (103,985 to 236,535)	0.83 (0.72 to 0.94)*
Nutritional deficiencies	3,912,001 (2,872,239 to 5,269,234)	2,247,485 (1,515,113 to 3,202,132)	-2.91 (-3.07 to -2.76)*	271,587 (210,115 to 352,577)	359,440 (285,475 to 450,281)	1.51 (1.21 to 1.81)*
Neoplasms	43,633,030 (40,123,940 to 47,258,486)	48,503,754 (40,915,702 to 56,689,779)	0.54 (0.36 to 0.73)*	10,782,364 (10,021,201 to 11,549,415)	19,016,076 (16,369,289 to 21,712,270)	3.00 (2.66 to 3.34)*
Cardiovascular diseases	40,746,813 (37,734,622 to 44,374,198)	45,823,157 (39,092,553 to 53,248,142)	0.67 (0.52 to 0.82)*	26,837,679 (24,915,174 to 29,373,891)	46,109,965 (40,669,589 to 51,698,469)	2.77 (2.26 to 3.29)*
Chronic respiratory diseases	13,237,789 (11,103,026 to 14,475,351)	8,969,697 (7,748,010 to 10,534,163)	-2.04 (-2.22 to -1.86)*	15,416,177 (12,665,862 to 16,567,447)	13,550,852 (11,816,464 to 16,036,186)	-0.70 (-1.15 to -0.24)*
Digestive diseases	9,775,769 (8,808,397 to 10,835,090)	7,710,658 (6,445,828 to 8,964,870)	-1.21 (-1.30 to -1.13)*	1,923,302 (1,762,799 to 2,106,969)	2,307,223 (2,029,697 to 2,600,797)	0.92 (0.72 to 1.13)*
Neurological disorders	9,677,839 (4,755,522 to 16,786,166)	11,207,269 (5,201,198 to 19,881,074)	0.78 (0.70 to 0.86)*	2,828,463 (1,694,636 to 5,326,904)	6,174,136 (3,629,888 to 11,661,806)	4.20 (4.04 to 4.35)*
Mental disorders	17,241,666 (12,746,572 to 22,736,338)	18,539,346 (13,738,299 to 24,152,654)	0.37 (0.29 to 0.46)*	868,363 (645,857 to 1,119,766)	1,754,481 (1,291,689 to 2,254,704)	3.77 (3.70 to 3.83)*
Musculoskeletal disorders	17,725,280 (12,654,675 to 23,783,378)	25,286,535 (18,045,649 to 33,942,087)	1.88 (1.84 to 1.92)*	2,364,272 (1,679,407 to 3,221,901)	4,647,389 (3,292,799 to 6,332,328)	3.60 (3.53 to 3.66)*
Other non-communicable diseases	23,430,651 (19,446,807 to 28,355,357)	16,912,189 (12,820,141 to 22,584,969)	-1.67 (-1.77 to -1.58)*	1,142,340 (831,796 to 1,540,224)	2,213,884 (1,599,002 to 2,992,458)	3.43 (2.88 to 3.98)*
Skin and subcutaneous diseases	7,427,502 (4,833,763 to 10,987,503)	7,564,844 (4,920,805 to 11,324,882)	0.09 (0.07 to 0.12)*	381,523 (263,385 to 542,001)	699,858 (475,558 to 1,030,747)	3.21 (3.14 to 3.28)*
Sense organ diseases	7,881,599 (5,268,499 to 11,424,474)	10,796,982 (7,099,138 to 15,924,830)	1.71 (1.43 to 2.00)*	2,859,183 (2,030,582 to 3,892,635)	5,184,684 (3,650,080 to 7,090,135)	3.18 (3.02 to 3.34)*
Transport injuries	16,117,112 (14,766,378 to 18,356,266)	12,597,412 (10,747,152 to 14,456,584)	-1.31 (-1.64 to -0.98)*	514,733 (454,228 to 601,574)	1,148,043 (946,296 to 1,360,515)	4.24 (3.92 to 4.57)*
Unintentional injuries	19,929,305 (18,307,020 to 21,760,124)	13,173,922 (10,853,663 to 15,202,200)	-2.15 (-2.65 to -1.65)*	1,184,747 (1,024,676 to 1,371,585)	2,829,540 (2,151,923 to 3,469,812)	4.66 (3.90 to 5.43)*
Self-harm and interpersonal violence	10,574,276 (9,275,669 to 11,544,266)	5,232,715 (4,479,867 to 6,200,044)	-3.64 (-3.94 to -3.34)*	597,000 (504,957 to 649,633)	637,335 (544,995 to 738,168)	0.37 (-0.73 to 1.50)
HIV/AIDS and sexually transmitted infections	904,578 (711,182 to 1,201,186)	1,677,944 (1,372,709 to 2,020,540)	3.33 (2.58 to 4.08)*	16,867 (13,900 to 22,250)	74,476 (55,158 to 94,754)	8.17 (6.93 to 9.43)*
Respiratory infections and tuberculosis	19,691,768 (18,048,674 to 21,296,455)	4,601,349 (3,952,381 to 5,376,466)	-7.32 (-7.79 to -6.85)*	2,246,454 (2,045,162 to 2,412,569)	2,046,523 (1,783,057 to 2,379,652)	-0.54 (-0.96 to -0.13)*
Enteric infections	3,259,938 (2,859,853 to 3,751,832)	1,179,711 (874,430 to 1,534,794)	-5.11 (-5.55 to -4.66)*	147,328 (108,525 to 198,192)	228,387 (164,251 to 306,599)	2.47 (2.25 to 2.69)*
Other infectious diseases	4,467,414 (3,610,475 to 6,110,768)	1,143,025 (971,599 to 1,357,011)	-6.85 (-7.29 to -6.42)*	114,576 (103,794 to 126,392)	101,074 (86,237 to 116,157)	-0.59 (-0.98 to -0.21)*
Maternal and neonatal disorders	18,100,148 (16,534,223 to 19,650,436)	7,080,589 (6,220,729 to 8,094,637)	-4.86 (-5.45 to -4.27)*	10,442 (7,450 to 14,363)	70,983 (51,665 to 96,401)	10.64 (10.47 to 10.82)*
Substance use disorders	6,144,467 (4,909,131 to 7,567,428)	5,459,715 (4,162,774 to 6,975,972)	-0.55 (-0.97 to -0.13)*	147,447 (122,722 to 177,177)	295,943 (226,537 to 375,653)	3.80 (3.23 to 4.37)*
Diabetes and kidney diseases	8,018,292 (6,937,163 to 9,207,068)	10,979,432 (9,048,531 to 13,121,983)	1.70 (1.42 to 1.98)*	2,418,531 (2,116,442 to 2,752,813)	4,929,741 (4,274,671 to 5,652,658)	3.78 (3.60 to 3.96)*
Rate						
Neglected tropical diseases and malaria	33 (21 to 49)	21 (13 to 34)	-2.26 (-2.56 to -1.96)*	250 (170 to 355)	149 (96 to 219)	-2.70 (-2.80 to -2.59)*
Nutritional deficiencies	82 (60 to 110)	48 (33 to 69)	-2.77 (-2.96 to -2.58)*	494 (383 to 642)	333 (264 to 417)	-2.03 (-2.30 to -1.76)*
Neoplasms	911 (838 to 987)	1,045 (881 to 1,221)	0.68 (0.59 to 0.77)*	19,629 (18,243 to 21,025)	17,613 (15,162 to 20,110)	-0.56 (-0.93 to -0.19)*
Cardiovascular diseases	851 (788 to 927)	987 (842 to 1,147)	0.84 (0.68 to 1.00)*	48,857 (45,357 to 53,474)	42,708 (37,669 to 47,885)	-0.69 (-0.82 to -0.57)*
Chronic respiratory diseases	276 (232 to 302)	193 (167 to 227)	-1.86 (-2.02 to -1.69)*	28,064 (23,058 to 30,160)	12,551 (10,945 to 14,853)	-4.13 (-4.60 to -3.66)*

For peer review only - http://bmjopen.bmj.com/site/about/guidelines.xhtml

Digestive diseases	204 (184 to 226)	166 (139 to 193)	-1.06 (-1.15 to -0.96)*	3,501 (3,209 to 3,836)	2,137 (1,880 to 2,409)	-2.55 (-2.66 to -2.45)*
Neurological disorders	202 (99 to 351)	241 (112 to 428)	0.94 (0.82 to 1.06)*	5,149 (3,085 to 9,697)	5,719 (3,362 to 10,801)	0.59 (0.29 to 0.90)*
Mental disorders	360 (266 to 475)	399 (296 to 520)	0.55 (0.51 to 0.59)*	1,581 (1,176 to 2,038)	1,625 (1,196 to 2,088)	0.15 (0.12 to 0.18)*
Musculoskeletal disorders	370 (264 to 497)	545 (389 to 731)	2.04 (2.00 to 2.08)*	4,304 (3,057 to 5,865)	4,305 (3,050 to 5,865)	-0.04 (-0.15 to 0.08)
Other non-communicable diseases	489 (406 to 592)	364 (276 to 486)	-1.54 (-1.66 to -1.42)*	2,080 (1,514 to 2,804)	2,051 (1,481 to 2,772)	-0.16 (-0.60 to 0.29)
Skin and subcutaneous diseases	155 (101 to 229)	163 (106 to 244)	0.26 (0.23 to 0.29)*	695 (479 to 987)	648 (440 to 955)	-0.38 (-0.39 to -0.36)*
Sense organ diseases	165 (110 to 239)	233 (153 to 343)	1.86 (1.70 to 2.02)*	5,205 (3,697 to 7,086)	4,802 (3,381 to 6,567)	-0.43 (-0.63 to -0.23)*
Transport injuries	337 (308 to 383)	271 (231 to 311)	-1.15 (-1.49 to -0.81)*	937 (827 to 1,095)	1,063 (876 to 1,260)	0.64 (0.33 to 0.96)*
Unintentional injuries	416 (382 to 454)	284 (234 to 327)	-1.93 (-2.44 to -1.42)*	2,157 (1,865 to 2,497)	2,621 (1,993 to 3,214)	1.01 (0.46 to 1.56)*
Self-harm and interpersonal violence	221 (194 to 241)	113 (96 to 134)	-3.49 (-3.80 to -3.18)*	1,087 (919 to 1,183)	590 (505 to 684)	-3.10 (-4.05 to -2.14)*
HIV/AIDS and sexually transmitted infections	19 (15 to 25)	36 (30 to 44)	3.42 (2.76 to 4.09)*	31 (25 to 41)	69 (51 to 88)	4.39 (3.26 to 3.53)*
Respiratory infections and tuberculosis	411 (377 to 445)	99 (85 to 116)	-7.17 (-7.61 to -6.71)*	4,090 (3,723 to 4,392)	1,896 (1,652 to 2,204)	-4.08 (-4.44 to -3.71)*
Enteric infections	68 (60 to 78)	25 (19 to 33)	-4.91 (-5.76 to -4.05)*	268 (198 to 361)	212 (152 to 284)	-1.20 (-1.45 to -0.96)*
Other infectious diseases	93 (75 to 128)	25 (21 to 29)	-6.70 (-7.14 to -6.27)*	209 (189 to 230)	94 (80 to 108)	-4.06 (-4.48 to -3.64)*
Maternal and neonatal disorders	378 (345 to 410)	153 (134 to 174)	-4.71 (-5.29 to -4.14)*	19 (14 to 26)	66 (48 to 89)	6.84 (6.69 to 6.98)*
Substance use disorders	128 (103 to 158)	118 (90 to 150)	-0.38 (-0.85 to 0.09)*	268 (223 to 323)	274 (210 to 348)	0.12 (-0.31 to 0.55)
Diabetes and kidney diseases	167 (145 to 192)	236 (195 to 283)	1.88 (1.58 to 2.18)*	4,403 (3,853 to 5,011)	4,566 (3,959 to 5,236)	0.21 (-0.05 to 0.48)

AAPC: average annual percent change; DALYs, disability adjusted life years; UI, uncertainty interval, CI, confidence interval.
*AAPC significantly differs from zero at a two-tailed level of 0.05.

For peer review only

For peer review only - http://bmjopen.bmj.com/site/about/guidelines.xhtml

Table 4. The AAPC of twenty risk factors in people younger and older than 70 years of age

Risk factors	Age < 70 years			Age ≥ 70 years		
	2000	2019	AAPC (95% CI)	2000	2019	AAPC (95% CI)
**Number**						
Unsafe water, sanitation, and handwashing	3,996,889 (3,183,257 to 4,887,734)	923,837 (645,583 to 1,254,723)	-7.42 (-8.15 to -6.69)*	237,825 (168,202 to 320,654)	228,667 (159,219 to 307,023)	-0.14 (-0.42 to 0.13)
Air pollution	32,546,257 (28,559,720 to 36,241,262)	24,301,291 (20,582,581 to 28,569,480)	-1.52 (-1.70 to -1.34)*	16,789,702 (14,415,333 to 19,203,189)	18,208,412 (15,565,447 to 21,260,363)	0.27 (0.00 to 0.54)*
Other environmental risks	3,596,355 (2,485,008 to 4,876,076)	3,222,783 (1,977,748 to 4,651,395)	-0.53 (-0.77 to -0.29)*	1,790,347 (1,199,126 to 2,480,222)	3,083,288 (2,034,326 to 4,259,059)	2.86 (2.48 to 3.23)*
Child and maternal malnutrition	27,473,911 (24,705,249 to 30,472,454)	6,937,292 (6,012,640 to 7,958,846)	-6.98 (-7.79 to -6.16)*	256,375 (197,956 to 332,924)	316,491 (248,797 to 398,362)	1.15 (0.66 to 1.63)*
Tobacco	37,227,472 (33,809,017 to 40,999,710)	38,743,936 (32,232,707 to 46,296,710)	0.22 (0.08 to 0.37)*	20,037,114 (18,407,452 to 21,694,478)	25,372,064 (22,095,893 to 28,843,357)	1.24 (0.79 to 1.69)*
Alcohol use	12,593,606 (10,822,456 to 14,698,783)	13,876,946 (11,135,736 to 16,941,170)	0.47 (0.39 to 0.56)*	2,079,979 (1,635,218 to 2,555,460)	3,388,114 (2,514,663 to 4,384,540)	2.51 (2.10 to 2.92)*
Drug use	5,880,735 (5,044,491 to 6,863,455)	4,596,666 (3,725,262 to 5,556,055)	-1.24 (-1.46 to -1.02)*	420,543 (338,823 to 505,234)	525,128 (420,389 to 638,546)	1.10 (0.84 to 1.35)*
High fasting plasma glucose	10,850,938 (8,940,115 to 13,134,425)	16,082,215 (12,638,442 to 19,989,086)	2.09 (1.69 to 2.50)*	7,073,207 (5,239,551 to 9,435,756)	12,146,224 (9,068,765 to 16,392,373)	2.86 (2.43 to 3.29)*
High systolic blood pressure	21,853,891 (18,103,552 to 25,435,298)	28,919,385 (23,316,926 to 34,533,463)	1.55 (1.29 to 1.81)*	13,487,603 (10,597,533 to 16,167,287)	25,522,231 (20,992,213 to 30,524,269)	3.37 (2.90 to 3.84)*
High body-mass index	9,311,731 (3,191,606 to 17,435,563)	18,520,264 (9,033,132 to 29,514,190)	3.70 (3.59 to 3.82)*	2,392,758 (706,129 to 5,015,786)	6,309,777 (2,532,862 to 11,224,097)	5.22 (4.79 to 5.65)*
Low bone mineral density	1,234,815 (1,026,536 to 1,443,017)	1,913,892 (1,512,289 to 2,304,790)	2.37 (2.10 to 2.64)*	534,562 (448,070 to 635,290)	1,406,383 (1,058,504 to 1,740,813)	5.22 (4.98 to 5.46)*
Dietary risks	24,107,231 (19,699,737 to 29,721,184)	29,061,110 (22,436,447 to 36,581,781)	1.02 (0.85 to 1.19)*	10,146,090 (7,449,653 to 13,767,645)	17,752,016 (12,683,501 to 23,791,732)	2.90 (2.59 to 3.21)*
Low physical activity	479,307 (211,814 to 1,031,049)	793,859 (332,155 to 1,622,549)	2.75 (2.34 to 3.15)*	795,916 (391,872 to 1,491,328)	1,713,262 (787,433 to 3,275,090)	3.97 (3.34 to 4.60)*
Occupational risks	14,894,928 (12,651,846 to 17,338,599)	11,828,821 (9,596,301 to 14,324,948)	-1.20 (-1.31 to -1.10)*	3,676,403 (2,901,359 to 4,413,363)	3,658,028 (2,941,788 to 4,424,853)	-0.10 (-0.45 to 0.25)
Intimate partner violence	766,126 (417,803 to 1,191,498)	739,392 (335,422 to 1,209,468)	-0.12 (-0.36 to 0.12)*	23,715 (10,039 to 44,392)	50,534 (16,543 to 99,799)	4.12 (3.96 to 4.29)*
Unsafe sex	1,352,963 (1,174,728 to 1,769,992)	2,445,381 (1,761,748 to 2,945,569)	3.11 (2.80 to 3.42)*	129,732 (113,048 to 182,721)	279,416 (197,910 to 338,222)	4.29 (3.79 to 4.79)*
Non-optimal temperature	3,641,568 (2,714,325 to 4,594,010)	3,468,758 (2,670,613 to 4,405,174)	-0.24 (-1.11 to 0.64)	4,785,750 (4,234,955 to 5,404,845)	5,856,605 (4,952,561 to 6,867,429)	0.76 (0.49 to 1.02)*
Kidney dysfunction	6,244,259 (5,571,754 to 7,041,339)	7,536,042 (6,221,117 to 9,011,471)	1.08 (0.82 to 1.35)*	2,639,944 (2,227,097 to 3,100,279)	5,817,866 (4,717,831 to 7,022,861)	4.30 (3.63 to 4.97)*
High LDL cholesterol	8,217,261 (6,919,451 to 9,686,198)	12,184,167 (9,541,238 to 14,971,019)	2.16 (1.91 to 2.42)*	3,000,527 (1,868,209 to 4,589,459)	7,629,795 (4,751,976 to 11,248,818)	5.02 (4.62 to 5.41)*
Childhood sexual abuse and bullying	755,840 (307,189 to 1,434,919)	629,932 (269,309 to 1,156,818)	-0.95 (-1.06 to -0.84)*	6,905 (2,954 to 12,651)	16,924 (7,261 to 32,044)	4.86 (4.72 to 5.00)*
**Rate**						
Unsafe water, sanitation, and handwashing	83 (66 to 102)	20 (14 to 27)	-7.24 (-7.97 to -6.51)*	433 (306 to 584)	212 (147 to 284)	-3.66 (-3.92 to -3.40)*
Air pollution	680 (596 to 757)	523 (443 to 615)	-1.36 (-1.53 to -1.19)*	30,565 (26,242 to 34,958)	16,865 (14,417 to 19,692)	-3.04 (-3.35 to -2.74)*
Other environmental risks	75 (52 to 102)	69 (43 to 100)	-0.37 (-0.59 to -0.14)*	3,259 (2,183 to 4,515)	2,856 (1,884 to 3,945)	-0.64 (-0.95 to -0.32)*
Child and maternal malnutrition	574 (516 to 636)	149 (130 to 171)	-6.83 (-7.48 to -6.18)*	467 (360 to 606)	293 (230 to 369)	-2.43 (-2.80 to -2.06)*
Tobacco	777 (706 to 856)	835 (694 to 997)	0.38 (0.23 to 0.53)*	36,476 (33,510 to 39,494)	23,500 (20,466 to 26,716)	-2.29 (-2.71 to -1.87)*
Alcohol use	263 (226 to 307)	299 (240 to 365)	0.61 (0.33 to 0.90)*	3,786 (2,977 to 4,652)	3,138 (2,329 to 4,061)	-1.09 (-1.57 to -0.61)*
Drug use	123 (105 to 143)	99 (80 to 120)	-1.02 (-1.22 to -0.82)*	766 (617 to 920)	486 (389 to 591)	-2.34 (-2.54 to -2.15)*

For peer review only

For peer review only - http://bmjopen.bmj.com/site/about/guidelines.xhtml

High fasting plasma glucose	227 (187 to 274)	346 (272 to 431)	2.25 (1.82 to 2.69)*	12,876 (9,538 to 17,177)	11,250 (8,400 to 15,183)	-0.79 (-1.48 to -0.10)*
High systolic blood pressure	456 (378 to 531)	623 (502 to 744)	1.72 (1.47 to 1.98)*	24,553 (19,292 to 29,432)	23,639 (19,444 to 28,272)	-0.15 (-0.42 to 0.13)
High body-mass index	194 (67 to 364)	399 (195 to 636)	3.88 (3.69 to 4.07)*	4,356 (1,285 to 9,131)	5,844 (2,346 to 10,396)	1.50 (1.01 to 2.00)*
Low bone mineral density	26 (21 to 30)	41 (33 to 50)	2.54 (2.20 to 2.89)*	973 (816 to 1,157)	1,303 (980 to 1,612)	1.62 (1.38 to 1.86)*
Dietary risks	503 (411 to 621)	626 (483 to 788)	1.19 (1.02 to 1.36)*	18,470 (13,562 to 25,063)	16,442 (11,748 to 22,037)	-0.58 (-0.74 to -0.43)*
Low physical activity	10 (4 to 22)	17 (7 to 35)	2.91 (2.46 to 3.36)*	1,449 (713 to 2,715)	1,587 (729 to 3,033)	0.40 (-0.21 to 1.02)*
Occupational risks	311 (264 to 362)	255 (207 to 309)	-1.05 (-1.10 to -0.99)*	6,693 (5,282 to 8,034)	3,388 (2,725 to 4,098)	-3.51 (-3.97 to -3.05)*
Intimate partner violence	16 (9 to 25)	16 (7 to 26)	0.02 (-0.23 to 0.27)*	43 (18 to 81)	47 (15 to 92)	0.49 (0.31 to 0.66)*
Unsafe sex	28 (25 to 37)	53 (38 to 63)	3.28 (2.96 to 3.60)*	236 (206 to 333)	259 (183 to 313)	0.69 (0.16 to 1.22)*
Non-optimal temperature	76 (57 to 96)	75 (58 to 95)	-0.05 (-0.89 to 0.81)*	8,712 (7,710 to 9,839)	5,425 (4,587 to 6,361)	-2.59 (-3.60 to -1.56)*
Kidney dysfunction	130 (116 to 147)	162 (134 to 194)	1.25 (1.00 to 1.49)*	4,806 (4,054 to 5,644)	5,389 (4,370 to 6,505)	0.64 (-0.02 to 1.31)
High LDL cholesterol	172 (144 to 202)	262 (206 to 322)	2.33 (2.07 to 2.59)*	5,462 (3,401 to 8,355)	7,067 (4,401 to 10,419)	1.44 (1.04 to 1.84)*
Childhood sexual abuse and bullying	16 (6 to 30)	14 (6 to 25)	-0.80 (-0.89 to -0.72)*	13 (5 to 23)	16 (7 to 30)	1.21 (1.06 to 1.37)*

AAPC: average annual percent change; DALYs, disability adjusted life years; UI, uncertainty interval, CI, confidence interval.
*AAPC significantly differs from zero at a two-tailed level of 0.05.

For peer review only

For peer review only - http://bmjopen.bmj.com/site/about/guidelines.xhtml

Discussion

In the past two decades, although China invested huge heath institution, personnel, and expenditures to extend life expectancy and improve healthy life expectancy, population structure deteriorated, the total disease burden remained largely unchanged, ageing driven disease burden continuously intensified. The trends we observed in the population structure of China highlights the potential for an irreversible population decline to occur before the household wealth of G7 nations is achieved in China. Despite easing of birth limits in China, couples have been put off by high costs of living, expensive childcare, career choices, and maternity leave. In addition, the post-working population has been increasing at an accelerating rate, suggesting a clear deterioration in population structure and ageing[16]. Thus, the ability of China to ease strains on its ageing population in the next 30 years is a pressing concern[18].

On the one hand, continuous effort is needed to address the top three causes and risk factors which have been identified among the population in China between 2000 and 2019. Our findings show that neoplasms and cardiovascular diseases remain among the top three causes, and tobacco remains among the top three risk factors for individuals younger than 70 years. Comprehensive regional-specific strategies, including effective tobacco-control policy, recommendations for healthier lifestyles, and control of chronic infections, are needed as the cancer spectrum in China is changing from that of a developing country to that of a developed country[19, 20]. Regulatory reforms also need to further reduce the lag between approval of new cancer drugs by the US FDA and their subsequent approval in China, and they need to facilitate the development and registration of drugs that may have clinical superiority over existing drugs[21]. Turning the inverted pyramid of the health care

For peer review only - http://bmjopen.bmj.com/site/about/guidelines.xhtml

system is essential for the battle against cardiovascular disease due to the increasing number of older patients, the high proportion of out-of-hospital deaths, and the gaps in lifestyle indicators between what is preferred and what is currently observed[22, 23]. All provinces in Chinese mainland have made great progress in increasing life expectancy at birth. Life course management of disease across the entire spectrum of health care services has led to a shift from a patient-centered and treatment-dominated model to a people-centered and health-centered model. It is hypothesized that the latter may help to integrate preventive and treatment measures for cardiovascular disease[24]. Tobacco sales have also increased despite a tight anti-smoking policy in China. Potential interference from the tobacco industry and the social currency of tobacco are suspected to be factors[25-27]. Therefore, great efforts should be taken not only to minimize these practices, but also to slow tobacco production[28].

On the other hand, it is crucial to focus on incremental driving causes and risk factors in the early stages to mitigate future ageing-driven disease burden. Our data show that musculoskeletal disorders, dietary risks, and high systolic blood pressure are among the top three causes and risk factors in people younger than 70 years. Split analyses were performed to examine variations according to age, and a cut-off point of 70 years was used because ischemic heart disease and stroke were the top-ranked causes of DALYs in the population aged 70 years and older[16]. A previous study quantified the temporal trends in disease burden of musculoskeletal disorders which could be attributed to changes in risk factors [27]. The results obtained support population-wide initiatives which target high body-mass index to help mitigate the burden of musculoskeletal disorders[29]. Changes in diets to correct for urbanization-driven dietary shifts to higher consumption of refined

For peer review only - http://bmjopen.bmj.com/site/about/guidelines.xhtml

sugars, refined fats, oils, and meats may also help prevent diet-related and chronic non-communicable diseases[30]. With resources available to control high systolic blood pressure and reduce the burden of hypertension, efforts are needed to improve access to high value antihypertensive medications and to implement artificial intelligence-based digital health[31, 32].

We identified key domains that require intervention in order to reduce an increasing disease burden among individuals older than 70 years. Most importantly, the top three causes and risk factors for this age group remained unchanged. Thus, our findings indicate that greater effort is needed to tackle the rapidly increasing age-standardized rate of DALYs due to traditional major risk factors, including high body-mass index, low bone mineral density, and high LDL cholesterol. Moreover, the increasing burden of maternal and neonatal disorders, HIV/AIDS and sexually transmitted infections, and unintentional injuries needs to be highlighted as well.

Health care for older individuals is undergoing unprecedented challenges due to underprepared geriatric hospitals and nursing homes, an unbalanced health care insurance system between urban and rural areas, and an absence of education on national aging and post-working health[18]. Consequently, there is an urgent need to identify traits in older people who experience short ill-spans. By identifying healthy behaviors and casual genetic and metabolic factors of the latter, it may be possible to decrease the burden of late-life diseases and extend healthspan[33]. The development of long-term health systems to meet the needs of older individuals in an age-friendly environment is fundamental to fostering healthy ageing and maximizing the functional abilities of older individuals[34].

Health care has experienced an upsurge in regard to medical institutions, personnel,

For peer review only

For peer review only - http://bmjopen.bmj.com/site/about/guidelines.xhtml

beds, and expenditures. Our findings clearly demonstrate that China has increased its health spending much faster than its economic growth. In 2018, China provided $644.7 million for the development of health assistance[35]. This trend in increasing health care expenditures will be further aggravated considering the high costs related to chronic illnesses, population growth, and ageing in the United States[9, 36]. While China has reformed its primary health care system to insure its population[7, 37], improving the quality of primary health care and increasing reimbursement for chronic non-communicable diseases are key steps to alleviating national disease burden[38, 39]. In addition, the cost-effectiveness of heath expenditures on disease burden and targeting the poor should be primary considerations in public health policy given the limited financial resources for heath[40].

Limitation of this study. It must also be noted that there were several limitations associated with the present study. First, our findings might be biased by changes in the age structure of the population and different population over a specified period because temporal trends in population and health care utilization were not quantified in terms of age standardized rate. Therefore, the trend of disease burden at each time was not evaluated based on quantification. Second, population and health care data from CSY 2020 were combined with modeled disease burden data from GBD 2019 based on an assumption that these two sources of data are consistent. Both data come from different databases, so there are some limitations on the burden of diseases in Chinese population. Third, the most recent trends could not be captured due to time lags in data release by the NBS of China and GBD studies. Fourth, all the limitations of the GBD methodology described elsewhere [3, 16, 17] could have potentially affected the present study. Fifth, owing to the nature of the data

For peer review only - http://bmjopen.bmj.com/site/about/guidelines.xhtml

and methods from CSY, we are currently unable to parse population estimates into more narrow age groups and provide deflator-adjusted health expenditure data. National-level data were collected and analyzed, and therefore, within-country variations were not considered.

Conclusion

Comprehensive age-cause-risk factor-specific strategies that target the younger population in China are needed to mitigate the causes and risk factors which have paralleled dynamic population changes that have occurred between 2000 and 2019 in China. The disease burden from metabolic risk factors, neoplasms and musculoskeletal disorders were identified as key domains that require intervention to reduce an increasing disease burden among individuals currently older than 70 years, as well as those approaching this age group. There remain huge unmet clinical needs across different age and future study should focus on improve suboptimal quality of life for patients with new drugs and strategies.

For peer review only

For peer review only - http://bmjopen.bmj.com/site/about/guidelines.xhtml

Contributors

Prof. Jieruo Gu had full access to all of the data in the study and takes responsibility for the integrity of the data and the accuracy of the data analysis. Dr. Dongze Wu, Dr.Zhenguo Liang, Dr. Cui Guo, and Prof. Jieruo Gu conceived and designed the study, performed the analysis, wrote the paper. All authors reviewed and revised the paper.

Conflict of Interest

The authors confirm that there are no conflicts of interest.

Funding Sources

Project funded by China Postdoctoral Science Foundation (2020TQ0380) and Guangdong Clinical Research Center of Immune disease (2020B1111170008). The Global Burden of Disease Study is funded by the Bill and Melinda Gates Foundation. The funder was not involved in the preparation of this manuscript.

Data sharing statement

Data used for the analyses are publicly available from the Institute of Health Metrics and Evaluation (www.ihme.org) and the National Bureau of Statistics of China (https://data.stats.gov.cn/english/index.htm; http://www.stats.gov.cn/tjsj/ndsj/2020/indexeh.htm).

Ethical approval

Data released from the Global Health Data Exchange query and the China Statistical Yearbook did not require informed patient consent. This study used an anonymized publicly available data set with no identifiable information of the survey participants. Thus, ethics approval was not required for this study.

For peer review only - http://bmjopen.bmj.com/site/about/guidelines.xhtml

For peer review only

References

1. Lopez-Otin, C., et al., *The hallmarks of aging.* Cell, 2013. **153**(6): p. 1194-217.
2. Lopez-Otin, C. and G. Kroemer, *Hallmarks of Health.* Cell, 2021. **184**(1): p. 33-63.

[dataset] 3. Collaborators, G.B.D.D., *Global age-sex-specific fertility, mortality, healthy life expectancy (HALE), and population estimates in 204 countries and territories, 1950-2019: a comprehensive demographic analysis for the Global Burden of Disease Study 2019.* Lancet, 2020. **396**(10258): p. 1160-1203.

4. Vollset, S.E., et al., *Fertility, mortality, migration, and population scenarios for 195 countries and territories from 2017 to 2100: a forecasting analysis for the Global Burden of Disease Study.* Lancet, 2020. **396**(10258): p. 1285-1306.
5. Zeng, Y. and T. Hesketh, *The effects of China's universal two-child policy.* Lancet, 2016. **388**(10054): p. 1930-1938.
6. Tatum, M., *China's three-child policy.* Lancet, 2021. **397**(10291): p. 2238.
7. Li, X., et al., *The primary health-care system in China.* Lancet, 2017. **390**(10112): p. 2584-2594.
8. Yip, W., et al., *10 years of health-care reform in China: progress and gaps in Universal Health Coverage.* Lancet, 2019. **394**(10204): p. 1192-1204.
9. Moses, H., 3rd, et al., *The anatomy of health care in the United States.* JAMA, 2013. **310**(18): p. 1947-63.
10. Zhong, N.S., et al., *Epidemiology and cause of severe acute respiratory syndrome (SARS) in Guangdong, People's Republic of China, in February, 2003.* Lancet, 2003. **362**(9393): p. 1353-8.
11. Zhu, N., et al., *A Novel Coronavirus from Patients with Pneumonia in China, 2019.* N Engl J Med, 2020. **382**(8): p. 727-733.
12. Gong, P., et al., *Urbanisation and health in China.* Lancet, 2012. **379**(9818): p. 843-52.
13. Zhou, M., et al., *Cause-specific mortality for 240 causes in China during 1990-2013: a systematic subnational analysis for the Global Burden of Disease Study 2013.* Lancet, 2016. **387**(10015): p. 251-72.
14. Zhou, M., et al., *Mortality, morbidity, and risk factors in China and its provinces, 1990-2017: a systematic analysis for the Global Burden of Disease Study 2017.* Lancet, 2019. **394**(10204): p. 1145-1158.

[dataset] 15. Ning, J.J., et al. *China Statistical Yearbook 2020.* Available from: http://www.stats.gov.cn/tjsj/ndsj/2020/indexeh.htm.

[dataset] 16. Diseases, G.B.D. and C. Injuries, *Global burden of 369 diseases and injuries in 204 countries and territories, 1990-2019: a systematic analysis for the Global Burden of Disease Study 2019.* Lancet, 2020. **396**(10258): p. 1204-1222.

[dataset] 17. Collaborators, G.B.D.R.F., *Global burden of 87 risk factors in 204 countries and territories, 1990-2019: a systematic analysis for the Global Burden of Disease Study 2019.* Lancet, 2020. **396**(10258): p. 1223-1249.

18. Wang, X.Q. and P.J. Chen, *Population ageing challenges health care in China.* Lancet, 2014. **383**(9920): p. 870.
19. Feng, R.M., et al., *Current cancer situation in China: good or bad news from the 2018 Global Cancer Statistics?* Cancer Commun (Lond), 2019. **39**(1): p. 22.

For peer review only - http://bmjopen.bmj.com/site/about/guidelines.xhtml

20. Chen, W., et al., *Disparities by province, age, and sex in site-specific cancer burden attributable to 23 potentially modifiable risk factors in China: a comparative risk assessment.* Lancet Glob Health, 2019. **7**(2): p. e257-e269.
21. Li, G., et al., *Characteristics of expedited programmes for cancer drug approval in China.* Nat Rev Drug Discov, 2021. **20**(6): p. 416.
22. Zhao, D., et al., *Epidemiology of cardiovascular disease in China: current features and implications.* Nat Rev Cardiol, 2019. **16**(4): p. 203-212.
23. Du, X., et al., *Epidemiology of Cardiovascular Disease in China and Opportunities for Improvement: JACC International.* J Am Coll Cardiol, 2019. **73**(24): p. 3135-3147.
24. Liu, S., Y. Li, and M. Zhou, *Addressing the Cardiovascular Disease Burden in China-Is It Possible Without Surgery?-Reply.* JAMA Cardiol, 2019. **4**(9): p. 953.
25. Luo, H. and Y. Zhao, *Increasing tobacco sales under the anti-smoking policy in China.* Lancet, 2020. **395**(10240): p. 1834.
26. Yang, G., et al., *The road to effective tobacco control in China.* Lancet, 2015. **385**(9972): p. 1019-28.
27. Rich, Z.C. and S. Xiao, *Tobacco as a social currency: cigarette gifting and sharing in China.* Nicotine Tob Res, 2012. **14**(3): p. 258-63.
28. Dan, X., S. Yuankai, and W. Chen, *Tobacco in China.* Lancet, 2014. **383**(9934): p. 2045-2046.
29. Wu, D., et al., *Pattern and trend of five major musculoskeletal disorders in China from 1990 to 2017: findings from the Global Burden of Disease Study 2017.* BMC Med, 2021. **19**(1): p. 34.
30. Tilman, D. and M. Clark, *Global diets link environmental sustainability and human health.* Nature, 2014. **515**(7528): p. 518-22.
31. Su, M., et al., *Availability, cost, and prescription patterns of antihypertensive medications in primary health care in China: a nationwide cross-sectional survey.* Lancet, 2017. **390**(10112): p. 2559-2568.
32. Brouwers, S., et al., *Arterial hypertension.* Lancet, 2021.
33. Partridge, L., J. Deelen, and P.E. Slagboom, *Facing up to the global challenges of ageing.* Nature, 2018. **561**(7721): p. 45-56.
34. Beard, J.R., et al., *The World report on ageing and health: a policy framework for healthy ageing.* Lancet, 2016. **387**(10033): p. 2145-2154.
35. Global Burden of Disease Health Financing Collaborator, N., *Past, present, and future of global health financing: a review of development assistance, government, out-of-pocket, and other private spending on health for 195 countries, 1995-2050.* Lancet, 2019. **393**(10187): p. 2233-2260.
36. Dieleman, J.L., et al., *Factors Associated With Increases in US Health Care Spending, 1996-2013.* JAMA, 2017. **318**(17): p. 1668-1678.
37. The, L., *China's health-care reform: an independent evaluation.* Lancet, 2019. **394**(10204): p. 1113.
38. Jan, S., et al., *Action to address the household economic burden of non-communicable diseases.* Lancet, 2018. **391**(10134): p. 2047-2058.
39. Li, X., et al., *Quality of primary health care in China: challenges and recommendations.* Lancet, 2020. **395**(10239): p. 1802-1812.

For peer review only

For peer review only - http://bmjopen.bmj.com/site/about/guidelines.xhtml

474 40. Li, H. and L. Jiang, *Catastrophic medical insurance in China.* Lancet, 2017.
475 **390**(10104): p. 1724-1725.

For peer review only

For peer review only - http://bmjopen.bmj.com/site/about/guidelines.xhtml

Figure legends

Figure 1. Temporal population trends in China between 2000 and 2019.

Temporal population composition is presented according to gender and area (A), age group (B), birth rate, death rate, and natural growth rate (C), and gross ratio, children ratio, and old dependency ratio (D).

Supplemental appendix

sFigure 1. Trend in life expectancy and healthy life expectancy at birth and at 60–64 years according to gender between 2000 and 2019.

sTable 1. AAPC in life expectancy at birth and at 60–64 years for 2000–2019

sTable 2. Percentage of healthy life expectancy within total life expectancy at birth for 2000–2019

sTable 3. Percentage of healthy life expectancy within total life expectancy at 60–64 years for 2000–2019

sTable 4. AAPC of all-cause disease burden for 2000–2019

sTable 5. AAPC of causes for 2000–2019

sTable 6. AAPC of risk factors for 2000–2019

For peer review only

For peer review only - http://bmjopen.bmj.com/site/about/guidelines.xhtml

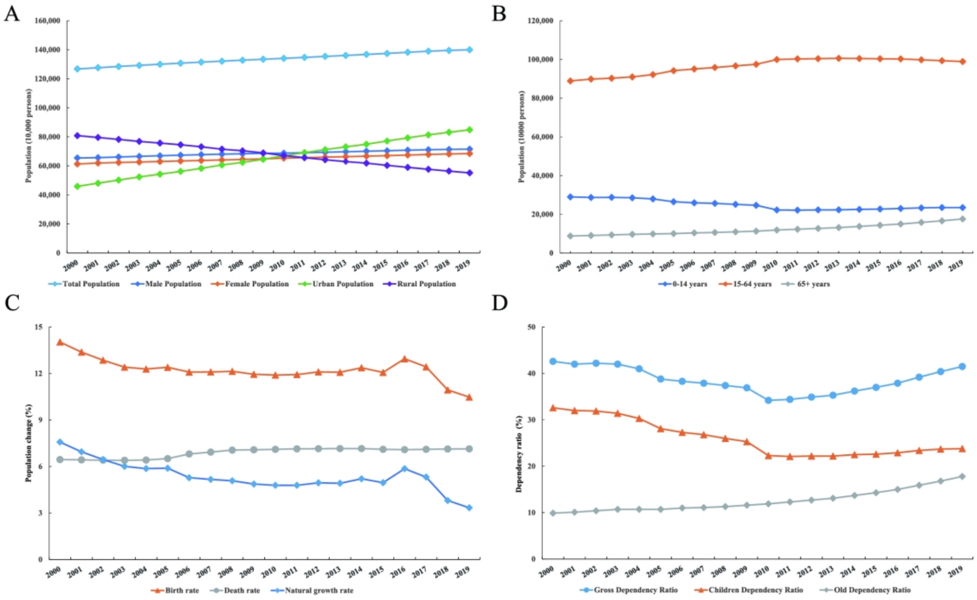

Temporal population trends in China between 2000 and 2019.

200x123mm (300 x 300 DPI)

Supplemental Appendix

Temporal trend of population structure, burden of diseases, healthcare resources and expenditure in China, 2000-2019

Table of Contents

Glossary and Definitions

Section 1: Population

Section 2: Health Care Institutions

Section 3: Medical Personnel

Section 4: Beds in Health Care Institutions

Section 5: Health Expenditures

Supplementary Figures

sFigure 1. Trend in life expectancy and healthy life expectancy at birth and at 60–64 years according to gender between 2000 and 2019.

Supplementary Tables

sTable 1. AAPC in life expectancy at birth and at 60–64 years for 2000–2019

sTable 2. Percentage of healthy life expectancy within total life expectancy at birth for 2000–2019

sTable 3. Percentage of healthy life expectancy within total life expectancy at 60–64 years for 2000–2019

sTable 4. AAPC of all-cause disease burden for 2000–2019

sTable 5. AAPC of causes for 2000–2019

sTable 6. AAPC of risk factors for 2000–2019

For peer review only

Glossary and Definitions

Glossary and definitions of population, healthcare institutions, medical personnel, beds in health care institutions, and health expenditure data were compiled from the 2020 China Statistical Yearbook (CSY) (http://www.stats.gov.cn/tjsj/ndsj/2020/indexeh.htm).

Section 1: Population

Total Population (year-end) (10000 persons): Year-end population refers to the population at midnight on 31 December. Annual statistics regarding the total population do not include data for Hong Kong, the Macau special administrative region and Taiwan province, and overseas numbers.

Urban Population refers to people residing in cities and towns in China.

Rural Population refers to people who reside outside of urban areas.

Birth Rate, Death Rate, and Natural Growth Rate of Population

Birth Rate (or Crude Birth Rate) refers to the ratio of the number of births to the average population (or mid-period population) during a certain period of time (usually a year). Birth rate in the chapter refers to annual birth rate and it is calculated as: number of births / annual average population × 1000. Number of births in the formula refers to live births (i.e., when a baby has breathed or showed any vital phenomena regardless of duration of gestation). Annual average population is the average of the population at the beginning of the year and the population at the end of the year. Sometimes it is substituted by the mid-year population.

Death Rate (or Crude Death Rate) refers to the ratio of the number of deaths to the average population (or mid-period population) during a certain period of time (usually a year). Death rate in the chapter refers to annual death rate and is calculated as: number of deaths / annual average population × 1000. Total Population (year-end) (10000 persons) refers to the population at midnight on 31 December.

Gross Dependency Ratio (or Coefficient) (%) refers to the ratio of non-working-age population to the working-age population. In general, Gross Dependency Ratio describes the number of non-working-age individuals that every 100 people of working age take care of. This reflects the basic relation between population and economic development from a demographic perspective.

Children Dependency Ratio (or Coefficient) (%) refers to the ratio of children to the working-age population. It describes the number of children that every 100 people of working age take care of.

Old Dependency Ratio (or Coefficient) (%) refers to the ratio of the elderly to the working-age population. It describes the number of elderly that every 100 people of working age take care of. Old dependency ratio is one of the indicators that reflect the social implication of population aging from an economic perspective.

Note: For the years, 2000 and 2010, census year estimates were available; the rest of the data were estimated based on annual national sample surveys.

Section 2: Health Care Institutions

Number of Health Care Institutions (unit)

Medical and health care institutions refer to units which have qualified to receive a Certification of Health Care Institution by the administration of public health, or a Certification of Corporate Unit by the civil affairs, administration for industry and commerce, commission office for public sector reform. Health care institutions engage in medical care, disease prevention and control, health supervision and inspection, medicine research, and on-job training. Health care institutions include hospitals, health care institutions at the grass-root level, specialized public health institutions, and other medical and health care institutions.

Number of Hospitals (unit)

Hospitals were considered to include general hospitals, hospitals of traditional Chinese medicine, hospitals that integrate traditional Chinese medicine and Western medicine, national hospitals, specialized hospitals, and nursing hospitals. Specialized disease prevention and cure centers, maternal and child health hospitals, and sanatoriums were excluded.

For peer review only

Health Care Institutions at Grass-root Level (unit)

Health care institutions at the grass-root level included: community health service centers, community service stations, institutes of health on the street, institutes of health in villages and towns, outpatient departments, and clinics (health centers).

Note:
1. Number of village clinics was included in health care institutions.
2. Since 2002, health care institutions no longer include headquarters of higher and secondary medical schools, drug test institutions, frontier health and quarantine institutions, and family.

Section 3: Medical Personnel

Number of Medical Personnel (10000 persons)

Medical personnel refer to all employees engaged at health care institutions, such as hospitals, health care institutions at grass-root level, specialized public health institutions, and other medical and health care institutions. These employees include medical technical personnel, village doctors and assistants, other technical personnel, and managerial and service staff. Medical personnel also refer to workers with payment of wages at the end of the year, including various types of employment personnel (including contract workers) and staff rehired for more than six months. Temporary workers, emeritus and retired, persons leaving the unit while remaining labor relations, and rehired and temporarily employed individuals for less than six months were excluded.

Number of Licensed Doctors (10000 persons)

Licensed doctors refer to medical workers who have obtained licenses of qualified doctors and are employed in medical treatment, disease prevention, or healthcare institutions. Licensed doctors engaged in management positions were excluded. Licensed doctors are divided into four categories: clinician, Chinese medicine physician, dentist, and public health physician.

Number of Registered Nurses (10000 persons)

Registered nurses refer to practical nursing personnel with a certificate of registered nurse. Nurses engaged in management positions were excluded.

Number of Other Technical Personnel (10000 persons)

Other technical personnel represent staff who graduated from high school and other institutions in the majors of chemistry and mathematics, as well as other non-health professionals, who are engaged in health promotion, research, teaching, and other technical work.

Number of Administrative Personnel (10000 persons)

Administrative personnel include persons in charge, personnel mainly engaged in health care, disease control, health surveillance, medical research, and teaching, other business management, and personnel mainly engaged in the party and government, personnel, finance, information, security, and other administrative work.

Note: Data of medical personnel and medical technical personnel include 10000 sanitation supervisors who serve as public servants.

Section 4: Beds in Health Care Institutions

Number of Beds in Health Care Institutions (10000 units)

Number of beds in health care institutions refers to a fixed sum of beds (non-prepared beds) at the end of the year. These include formal beds, simple beds, care beds, beds being disinfected and repaired, and disabled beds due to expansion or overhaul. Obstetric neonatal beds, expectant beds of confinement rooms, inventory beds, observation beds, extra beds, and escort beds for patient's family members were excluded.

Number of Beds in Medical Institutions per 10000 Population

Number of beds in medical institutions refers to a fixed sum of beds (non-prepared beds) at the end of the year. These include formal beds, simple beds, care beds, beds being disinfected and repaired, and disabled beds due to expansion or overhaul. Obstetric neonatal beds, expectant beds of confinement rooms, inventory beds, observation beds, extra beds, and escort beds for patient's family members were excluded.

For peer review only

Number of Beds in Urban Medical Institutions (10000 units)

Number of beds in urban medical institutions refers to a fixed sum of beds (non-prepared beds) at the end of the year. These include formal beds, simple beds, care beds, beds being disinfected and repaired, and disabled beds due to expansion or overhaul. Obstetric neonatal beds, expectant beds of confinement rooms, inventory beds, observation beds, extra beds, and escort beds for patient's family members were excluded.

Number of Beds in Rural Medical Institutions (10000 units)

Number of beds in rural medical institutions refers to a fixed sum of beds (non-prepared beds) at the end of the year. These include formal beds, simple beds, care beds, beds being disinfected and repaired, and disabled beds due to expansion or overhaul. Obstetric neonatal beds, expectant beds of confinement rooms, inventory beds, observation beds, extra beds, and escort beds for patient's family members were excluded.

Beds of Medical Institutions per 10000 Population

Beds of Medical Institutions per 10000 Population = number of beds of medical and health care institutions / population ×10000. The population value used is based on household registration data from the Ministry of Public Security.

Section 5: Health Expenditures

Total Health Expenditure (100 million yuan)

Total health expenditure refers to the total expenditure on medical and health care services (usually in monetary value) by a country or region within a certain period of time (usually a year). Total health expenditure is counted according to the source method. Under certain economic conditions, it reflects the emphasis and burden level of cost on government health care, the society's healthcare, and on individual citizens. Total health expenditure also represents the main features of health financing mode and fairness, as well as the rationality of health financing.

Government Health Expenditure (100 million yuan)

Government expenditure on public health refers to expenditures by the government at all levels of medical and health care services, medical subsidies, health administration and health insurance management, and undertakings of family planning.

Social Health Expenditure (100 million yuan)

Social expenditure on public health refers to all inputs from society, and not from the government in the form of expenditures on social medical security, commercial health insurance, private expenditure on operation of medical and health care, social donations and contributions, and income from administrative fees.

Out-of-pocket Health Expenditure (100 million yuan)

Individual cash expenditure on health refers to the expenditure in cash by rural and urban residents for various health services, including self-payments of residents within the system of multi-medical insurance.

Per Capita Health Expenditure (yuan)

Per capita health expenditure refers to the ratio of total expenditure on health in a year to the average population.

For peer review only

Supplementary Figures

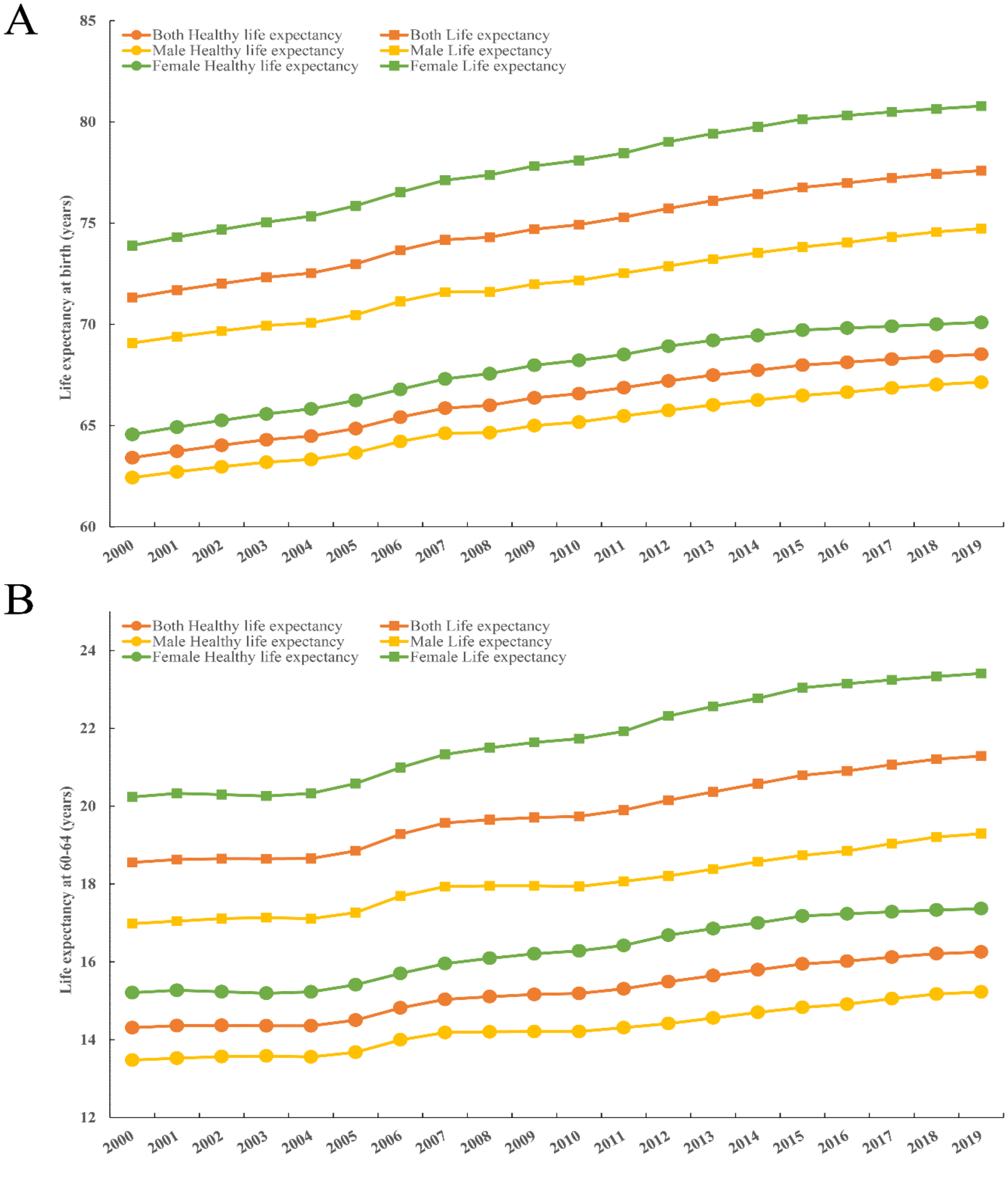

sFigure 1. Trend in life expectancy and healthy life expectancy at birth and at 60–64 years according to gender between 2000 and 2019.
The trend of life expectancy and healthy life expectancy at birth (A) and at 60–64 years (B) according to gender between 2000 and 2019. The distance between life expectancy and healthy life expectancy denotes unhealthy life expectancy (ill-span).

Supplementary Tables

sTable 1. AAPC in life expectancy at birth and at 60–64 years for 2000–2019

	Life expectancy (95% UI)			AAPC (95% CI)	
	2000	2019	2000–2019	2000–2009	2010–2019
**Life expectancy at birth**					
Both					
Life expectancy	71.33 (70.56 to 72.06)	77.59 (76.27 to 79.00)	0.44 (0.43 to 0.46)*	0.50 (0.47 to 0.53)*	0.40 (0.38 to 0.42)*
Healthy life expectancy	63.42 (60.07 to 65.54)	68.53 (60.88 to 70.97)	0.41 (0.38 to 0.43)*	0.50 (0.45 to 0.54)*	0.31 (0.29 to 0.34)*
Male					
Life expectancy	69.08 (68.02 to 70.16)	74.74 (72.78 to 76.70)	0.42 (0.38 to 0.46)*	0.46 (0.37 to 0.54)*	0.39 (0.37 to 0.41)*
Healthy life expectancy	62.43 (60.44 to 64.36)	67.14 (60.64 to 69.82)	0.38 (0.36 to 0.41)*	0.44 (0.39 to 0.49)*	0.32 (0.30 to 0.35)*
Female					
Life expectancy	73.90 (72.91 to 74.81)	80.78 (79.10 to 82.47)	0.47 (0.43 to 0.51)*	0.57 (0.50 to 0.63)*	0.37 (0.32 to 0.41)*
Healthy life expectancy	64.57 (60.86 to 66.96)	70.10 (60.92 to 73.19)	0.43 (0.40 to 0.46)*	0.56 (0.50 to 0.62)*	0.30 (0.27 to 0.33)*
**Life expectancy at 60–64 years**					
Both					
Life expectancy	18.56 (18.06 to 19.02)	21.29 (20.32 to 22.33)	0.76 (0.60 to 0.91)*	0.65 (0.37 to 0.93)*	0.91 (0.81 to 1.01)*
Healthy life expectancy	14.31 (13.16 to 15.43)	16.26 (14.82 to 17.67)	0.69 (0.51 to 0.87)*	0.64 (0.27 to 1.01)*	0.73 (0.66 to 0.80)*
Male					
Life expectancy	16.99 (16.32 to 17.66)	19.29 (17.97 to 20.68)	0.68 (0.61 to 0.74)*	0.58 (0.46 to 0.70)*	0.85 (0.80 to 0.89)*
Healthy life expectancy	13.48 (12.42 to 14.53)	15.23 (13.82 to 16.79)	0.65 (0.55 to 0.76)*	0.56 (0.37 to 0.75)*	0.81 (0.76 to 0.86)*
Female					
Life expectancy	20.24 (19.62 to 20.86)	23.41 (22.10 to 24.77)	0.76 (0.55 to 0.98)*	0.75 (0.41 to 1.09)*	0.76 (0.47 to 1.04)*
Healthy life expectancy	15.21 (13.83 to 16.46)	17.37 (15.61 to 19.16)	0.70 (0.54 to 0.85)*	0.69 (0.40 to 0.98)*	0.68 (0.54 to 0.81)*

Note: AAPC: average annual percent changes; UI, uncertainty interval; CI, confidence interval.
*AAPC significantly differs from zero at the alpha = 0.05 level.

For peer review only

sTable 2. Percentage of healthy life expectancy within total life expectancy at birth for 2000–2019

Year	Healthy life expectancy at birth (years)			Life expectancy at birth (years)			Healthy within total life expectancy (%)		
	Total	Male	Female	Total	Male	Female	Total	Male	Female
2000	63.42	62.43	64.57	71.33	69.08	73.90	88.90	90.38	87.37
2001	63.73	62.72	64.92	71.69	69.40	74.31	88.90	90.37	87.37
2002	64.03	62.96	65.26	72.02	69.67	74.69	88.90	90.37	87.38
2003	64.30	63.20	65.57	72.33	69.94	75.05	88.89	90.36	87.38
2004	64.49	63.33	65.83	72.54	70.08	75.34	88.90	90.37	87.37
2005	64.86	63.66	66.25	72.98	70.47	75.86	88.87	90.34	87.33
2006	65.42	64.22	66.79	73.67	71.14	76.54	88.80	90.27	87.27
2007	65.87	64.62	67.31	74.19	71.60	77.12	88.79	90.25	87.27
2008	66.00	64.65	67.57	74.31	71.61	77.38	88.83	90.28	87.32
2009	66.38	65.00	67.98	74.70	71.99	77.82	88.85	90.30	87.35
2010	66.58	65.18	68.22	74.92	72.17	78.10	88.87	90.31	87.36
2011	66.88	65.48	68.51	75.29	72.53	78.46	88.82	90.27	87.31
2012	67.21	65.75	68.92	75.73	72.88	79.03	88.75	90.22	87.21
2013	67.50	66.03	69.21	76.11	73.23	79.42	88.69	90.17	87.14
2014	67.74	66.26	69.45	76.44	73.53	79.76	88.62	90.11	87.08
2015	67.99	66.49	69.72	76.77	73.83	80.14	88.56	90.06	87.00
2016	68.12	66.65	69.82	76.98	74.05	80.32	88.49	90.01	86.93
2017	68.28	66.86	69.91	77.23	74.33	80.50	88.41	89.95	86.85
2018	68.42	67.03	70.01	77.44	74.57	80.65	88.36	89.89	86.81
2019	68.53	67.14	70.10	77.59	74.74	80.78	88.31	89.84	86.78

For peer review only

sTable 3. Percentage of healthy life expectancy within total life expectancy at 60–64 years for 2000–2019

Year	Healthy life expectancy at 60–64 years			Life expectancy at 60–64 year			Percentage of healthy within total life expectancy		
	Total	Male	Female	Total	Male	Female	Total	Male	Female
2000	14.31	13.48	15.21	18.56	16.99	20.24	77.12%	79.35%	75.15%
2001	14.36	13.52	15.27	18.63	17.05	20.33	77.09%	79.33%	75.11%
2002	14.37	13.57	15.23	18.65	17.11	20.30	77.05%	79.29%	75.06%
2003	14.36	13.58	15.19	18.65	17.14	20.26	76.99%	79.24%	74.98%
2004	14.36	13.56	15.23	18.66	17.11	20.33	76.97%	79.24%	74.93%
2005	14.50	13.68	15.41	18.85	17.27	20.58	76.93%	79.21%	74.87%
2006	14.82	14.00	15.70	19.29	17.69	20.99	76.84%	79.12%	74.80%
2007	15.04	14.18	15.95	19.57	17.93	21.33	76.82%	79.09%	74.79%
2008	15.11	14.20	16.09	19.65	17.95	21.50	76.87%	79.12%	74.84%
2009	15.16	14.21	16.21	19.71	17.95	21.64	76.93%	79.17%	74.90%
2010	15.19	14.21	16.28	19.74	17.94	21.73	76.96%	79.21%	74.93%
2011	15.31	14.31	16.42	19.90	18.07	21.93	76.94%	79.20%	74.89%
2012	15.49	14.42	16.69	20.15	18.21	22.32	76.87%	79.19%	74.78%
2013	15.65	14.56	16.86	20.37	18.39	22.56	76.82%	79.18%	74.71%
2014	15.80	14.71	17.00	20.58	18.58	22.77	76.77%	79.16%	74.65%
2015	15.95	14.83	17.18	20.79	18.74	23.04	76.69%	79.13%	74.55%
2016	16.02	14.91	17.23	20.91	18.85	23.15	76.62%	79.11%	74.45%
2017	16.12	15.05	17.28	21.07	19.04	23.25	76.54%	79.08%	74.35%
2018	16.21	15.17	17.33	21.21	19.21	23.33	76.45%	78.99%	74.28%
2019	16.26	15.23	17.37	21.29	19.29	23.41	76.36%	78.93%	74.18%

For peer review only

sTable 4. AAPC of all-cause disease burden for 2000–2019

Subcategory	All-cause estimates (95% UI)		AAPC (95% CI)		
	2000	2019	2000–2019	2000–2009	2010–2019
**Number**					
Incidence	5,291,848,308 (4,993,009,197 to 5,644,956,487)	5,790,243,111 (5,505,879,019 to 6,107,248,437)	0.48 (0.45 to 0.50)*	0.19 (0.16 to 0.22)*	0.82 (0.78 to 0.85)*
Prevalence	1,241,468,252 (1,234,531,622 to 1,247,534,242)	1,362,817,881 (1,356,594,402 to 1,368,714,232)	0.49 (0.49 to 0.50)*	0.47 (0.47 to 0.48)*	0.52 (0.51 to 0.52)*
Death	8,653,295 (8,170,487 to 9,200,531)	10,653,448 (9,310,978 to 12,069,760)	1.12 (0.93 to 1.31)*	0.89 (0.61 to 1.18)*	1.27 (1.03 to 1.51)*
Years lived with disability	116,137,933 (86,494,934 to 150,490,082)	153,797,874 (115,409,603 to 199,426,123)	1.49 (1.46 to 1.52)*	1.29 (1.24 to 1.34)*	1.74 (1.72 to 1.77)*
Years of life lost	260,536,746 (246,148,674 to 276,354,908)	228,407,694 (197,155,841 to 260,733,993)	-0.70 (-0.95 to -0.46)*	-1.34 (-1.53 to -1.15)*	-0.07 (-0.51 to 0.38)
DALY	376,674,679 (343,098,150 to 416,187,725)	382,205,568 (332,307,099 to 433,824,950)	0.08 (-0.06 to 0.22)	-0.44 (-0.58 to -0.31)*	0.60 (0.39 to 0.82)*
Risk factor (DALY)	176,792,905 (163,998,804 to 190,556,819)	187,705,961 (165,023,927 to 212,244,649)	0.34 (0.20 to 0.48)*	-0.27 (-0.52 to -0.03)*	0.92 (0.79 to 1.06)*
**Age standardized rate**					
Incidence	414,111 (390,563 to 440,957)	418,081 (394,196 to 444,709)	0.04 (0.02 to 0.07)*	-0.21 (-0.24 to -0.17)*	0.34 (0.30 to 0.38)*
Prevalence	94,824 (94,187 to 95,367)	93,439 (92,723 to 94,090)	-0.08 (-0.09 to -0.07)*	-0.17 (-0.19 to -0.15)*	0.00 (-0.01 to 0.00)
Death	956 (910 to 1,010)	634 (561 to 711)	-2.14 (-2.28 to -2.00)*	-2.00 (-2.20 to -1.79)*	-2.38 (-2.56 to -2.21)*
Years lived with disability	9,078 (6,779 to 11,713)	8,924 (6,654 to 11,539)	-0.08 (-0.12 to -0.05)*	-0.34 (-0.38 to -0.30)*	0.22 (0.17 to 0.28)*
Years of life lost	24,833 (23,513 to26,239)	13,347 (11,701 to14,997)	-3.22 (-3.33 to -3.11)*	-3.43 (-3.59 to -3.27)*	-3.09 (-3.23 to -2.96)*
DALY	33,911 (31,131 to 37,035)	22,271 (19,482 to 25,195)	-2.21 (-2.35 to -2.07)*	-2.54 (-2.64 to -2.43)*	-1.85 (-2.12 to -1.58)*
Risk factor (DALY)	16,689 (15,569 to 17,886)	10,310 (9,122 to 11,524)	-2.49 (-2.69 to -2.29)*	-2.93 (-3.27 to -2.58)*	-2.07 (-2.30 to -1.85)*

Note: AAPC: average annual percent changes; UI, uncertainty interval; CI, confidence interval; DALY, disability-adjusted life years. *AAPC significantly differs from zero at the alpha = 0.05 level.

For peer review only

sTable 5. AAPC of causes for 2000–2019

Causes	DALY (95% UI)				AAPC (95% CI)		
	2000	Rank	2019	Rank	2000–2019	2000–2009	2010–2019
**All-age numbers**							
Neglected tropical diseases and malaria	1,704,065 (1,121,580 to 2,525,119)	21	1,137,806 (692, 480 to 1,800,439)	22	-2.07 (-2.35 to -1.79)*	-3.57 (-3.89 to -3.25)*	-0.50 (-0.97 to -0.02)*
Nutritional deficiencies	4,183,588 (3,082,941 to 5,605,252)	19	2,606,926 (1,815,566 to 3,659,294)	18	-2.47 (-2.64 to -2.30)*	-4.30 (-4.49 to -4.11)*	-0.53 (-0.79 to -0.26)*
Neoplasms	54,415,394 (50,247,614 to 58,721,812)	2	67,519,830 (1,418,891 to 3,248,407)	2	1.13 (0.95 to 1.31)*	0.87 (0.57 to 1.17)*	1.42 (1.20 to 1.65)*
Cardiovascular diseases	67,584,492 (62,771,497 to 73,832,854)	1	91,933,122 (1,135,346 to 3,124,922)	1	1.62 (1.45 to 1.79)*	1.70 (1.40 to 2.00)*	1.42 (1.32 to 1.53)*
Chronic respiratory diseases	28,653,966 (23,770,991 to 30,931,146)	3	22,520,549 (1,719,779 to 3,416,880)	4	-1.28 (-1.50 to -1.06)*	-2.02 (-2.40 to -1.65)*	-0.50 (-0.75 to -0.26)*
Digestive diseases	11,699,071 (10,590,221 to 12,920,126)	12	10,017,880 (1,549,912 to 3,496,363)	12	-0.80 (-0.87 to -0.73)*	-1.02 (-1.13 to -0.90)*	-0.51 (-0.60 to -0.43)*
Neurological disorders	12,506,303 (6,994,212 to 20,592,252)	11	17,381,406 (1,666,405 to 3,082,962)	7	1.75 (1.67 to 1.82)*	1.79 (1.70 to 1.89)*	1.72 (1.61 to 1.84)*
Mental disorders	18,110,029 (13,449,072 to 23,810,039)	9	20,293,827 (1,145,615 to 3,345,133)	5	0.59 (0.49 to 0.69)*	0.45 (0.40 to 0.49)*	0.75 (0.54 to 0.95)*
Musculoskeletal disorders	20,089,552 (14,362,360 to 26,771,117)	7	29,933,924 (1,427,199 to 3,823,644)	3	2.10 (2.04 to 2.16)*	2.44 (2.36 to 2.52)*	1.75 (1.67 to 1.83)*
Other non-communicable diseases	24,572,991 (20,333,568 to 29,891,261)	4	19,126,072 (1,446,224 to 3,590,726)	6	-1.31 (-1.48 to -1.13)*	-2.43 (-2.53 to -2.33)*	-0.04 (-0.40 to -032)
Skin and subcutaneous diseases	7,809,025 (5,108,660 to 11,567,830)	16	8,264,702 (1,394,816 to 3,356,265)	13	0.29 (0.27 to 0.32)*	0.33 (0.29 to 0.38)*	0.28 (0.26 to 0.29)*
Sense organ diseases	10,740,782 (7,311,356 to 15,203,366)	14	15,981,667 (1,788,420 to 3,997,480)	9	2.14 (1.98 to 2.30)*	1.98 (1.94 to 2.03)*	2.31 (1.97 to 2.64)*
Transport injuries	16,631,845 (15,221,113 to 18,940,342)	10	13,745,455 (1,719,684 to 3,768,996)	11	-1.02 (-1.35 to -0.69)*	0.57 (0.21 to 0.95)*	-2.66 (-3.22 to -2.10)*
Unintentional injuries	21,114,052 (19,362,287 to 23,062,953)	6	16,003,462 (1,056,699 to 3,613,615)	8	-1.53 (-2.01 to -1.04)*	-1.53 (-2.01 to -1.04)*	-1.53 (-2.01 to -1.04)*
Self-harm and interpersonal violence	11,171,276 (9,775,023 to 12,180,298)	13	5,870,049 (1,030,733 to 3,924,034)	16	-3.35 (-3.70 to -3.01)*	-4.09 (-4.73 to -3.45)*	-2.47 (-2.75 to -2.18)*
HIV/AIDS and sexually transmitted infections	921,444,444 (727,466,466 to 1,222,963)	22	1,752,419 (1,430, 192 to 2,114,213)	19	3.47 (2.72 to 4.22)*	3.07 (2.38 to 3.77)*	4.07 (2.71 to 5.45)*
Respiratory infections and tuberculosis	21,938,222 (20,220,847 to 23,581,022)	5	6,647,872 (1,853,547 to 3,613,257)	15	-6.10 (-6.41 to -5.79)*	-8.88 (-9.36 to -8.40)*	-3.34 (-3.65 to -3.03)*
Enteric infections	3,407,267 (2,990,655 to 3,948,769)	20	1,408,098 (1,047,418 to 3,827,650)	20	-4.41 (-5.28 to -3.54)*	-9.05 (-9.96 to -8.13)*	0.68 (-0.56 to 1.92)
Other infectious diseases	4,581,989 (3,724,438 to 6,219,671)	18	1,244,099 (1,063,407 to 3,462,432)	21	-6.57 (-6.95 to -6.19)*	-9.03 (-9.45 to -8.61)*	-4.04 (-4.71 to -3.36)*
Maternal and neonatal disorders	18,110,589 (16,543,873 to 19,663,056)	8	7,151,571 (1,273,977 to 3,174,922)	14	-4.82 (-5.40 to -4.23)*	-6.06 (-6.60 to -5.51)*	-3.56 (-4.60 to -2.51)*
Substance use disorders	6,291,914 (5,043,188 to 7,751,372)	17	5,755,658 (1,373,805 to 3,343,432)	17	-0.39 (-0.82 to 0.03)	-1.74 (-2.49 to -0.99)*	0.94 (0.53 to 1.36)*
Diabetes and kidney diseases	10,436,824 (9,083,252 to 11,910,785)	15	15,909,173 (1,346,885 to 3,824,247)	10	2.30 (2.09 to 2.51)*	2.87 (2.44 to 3.31)*	1.79 (1.70 to 1.89)*
**Age-standardized rate**							
Neglected tropical diseases and malaria	132 (89 to 194)	21	68 (42 to 107)	22	-3.40 (-3.66 to -3.13)*	-4.75 (-5.06 to -4.45)*	-1.97 (-2.42 to -1.51)*
Nutritional deficiencies	369 (284 to 480)	20	175 (122 to 241)	18	-3.88 (-4.07 to -3.69)*	-5.98 (-6.27 to -5.70)*	-1.84 (-2.11 to -1.58)*
Neoplasms	4,505 (4,163 to 4,847)	2	3,421 (2,933 to 3,946)	2	-1.46 (-1.71 to -1.21)*	-1.62 (-1.88 to -1.36)*	-1.25 (-1.68 to -1.81)*
Cardiovascular diseases	6,441 (5,997 to 7,005)	1	4,938 (4,318 to 5,577)	1	-1.40 (-1.56 to -1.24)*	-1.10 (-1.39 to -0.82)*	-1.84 (-1.94 to -1.75)*
Chronic respiratory diseases	2,947 (2,458 to 3,175)	3	1,270 (1,114 to 1,474)	5	-4.34 (-4.61 to -4.07)*	-4.90 (-5.37 to -4.44)*	-3.74 (-4.04 to -3.45)*
Digestive diseases	974 (889 to 1,070)	12	539 (463 to 616)	14	-3.07 (-3.14 to -3.00)*	-3.29 (-3.41 to -3.16)*	-2.77 (-2.84 to -2.71)*
Neurological disorders	1,103 (650 to 1,773)	11	1,077 (598 to 1,780)	8	-0.15 (-0.24 to -0.06)*	-0.09 (-0.16 to -0.02)*	-0.22 (-0.40 to -0.03)*
Mental disorders	1,317 (978 to 1,727)	9	1,248 (922 to 1,627)	6	-0.27 (-0.34 to -0.20)*	-0.56 (-0.59 to -0.53)*	0.01 (-0.13 to 0.14)

For peer review only

Musculoskeletal disorders	1,536 (1,102 to 2,050)	8	1,585 (1,140 to 2,112)	3	0.16 (0.10 to 0.23)*	0.31 (0.26 to 0.36)*	0.02 (-0.11 to 0.15)
Other non-communicable diseases	2,315 (1,963 to 2,719)	5	1,349 (1,066 to 1,726)	4	-2.81 (-2.96 to -2.67)*	-3.49 (-3.65 to -3.34)*	-2.00 (-2.25 to -1.75)*
Skin and subcutaneous diseases	606 (397 to 898)	16	606 (391 to 909)	13	0.00 (-0.01 to 0.01)	-0.05 (-0.07 to -0.04)*	0.06 (0.03 to 0.08)*
Sense organ diseases	926 (633 to 1,299)	13	863 (591 to 1,226)	9	-0.36 (-0.45 to -0.27)*	-0.46 (-0.49 to -0.43)*	-0.25 (-0.43 to -0.07)*
Transport injuries	1,248 (1,146 to 1,433)	10	860 (740 to 975)	10	-1.98 (-2.42 to -1.54)*	-0.63 (-1.14 to -0.13)*	-3.39 (-4.12 to -2.65)*
Unintentional injuries	1,886 (1,744 to 2,045)	7	1,102 (919 to 1,254)	7	-2.81 (-3.24 to -2.39)*	-2.81 (-3.24 to -2.39)*	-2.81 (-3.24 to -2.39)*
Self-harm and interpersonal violence	807 (708 to 877)	15	361 (310 to 421)	16	-4.19 (-4.62 to -3.76)*	-5.13 (-5.93 to -4.32)*	-3.08 (-3.43 to -2.73)*
HIV/AIDS and sexually transmitted infections	78 (56 to 114)	22	116 (92 to 147)	19	2.02 (1.50 to 2.53)*	2.61 (2.31 to 2.91)*	1.88 (1.05 to 2.72)*
Respiratory infections and tuberculosis	2,396 (2,209 to 2,582)	4	518 (458 to 593)	15	-7.77 (-7.93 to -7.61)*	-9.56 (-9.70 to -9.41)*	-5.88 (-6.18 to -5.58)*
Enteric infections	369 (326 to 422)	19	111 (84 to 144)	20	-6.04 (-6.52 to -5.56)*	-10.47 (-11.00 to -9.94)*	-1.52 (-2.31 to -0.73)*
Other infectious diseases	473 (376 to 659)	17	110 (92 to 131)	21	-7.37 (-7.84 to -6.91)*	-8.51 (-9.21 to -7.80)*	-6.04 (-6.58 to -5.50)*
Maternal and neonatal disorders	2,177 (1,983 to 2,375)	6	792 (699 to 897)	12	-5.26 (-5.36 to -5.16)*	-5.26 (-5.36 to -5.16)*	-5.26 (-5.36 to -5.16)*
Substance use disorders	428 (345 to 526)	18	347 (262 to 443)	17	-1.03 (-1.38 to -0.69)*	-2.60 (-3.02 to -2.18)*	0.85 (0.35 to 1.36)*
Diabetes and kidney diseases	876 (764 to 997)	14	817 (688 to 961)	11	-0.32 (-0.47 to -0.16)*	0.46 (0.17 to 0.75)*	-1.01 (-1.12 to -0.90)*

Note: AAPC: average annual percent changes; UI, uncertainty interval; CI, confidence interval.
*AAPC significantly differs from zero at the alpha = 0.05 level.

For peer review only

sTable 6. AAPC of risk factors for 2000–2019

Risk Factors	DALY (95% UI)				AAPC (95% CI)		
	2000	Rank	2019	Rank	2000–2019	2000–2009	2010–2019
**All-age numbers**							
Unsafe water, sanitation, and handwashing	4,234,714 (3,363,558 to 5,211,019)	15	1,152,504 (805,846 to 1,554,455)	18	-6.60 (-7.28 to -5.91)*	-10.80 (-11.04 to -10.55)*	-2.34 (-3.77 to -0.88)*
Air pollution	49,335,959 (43,328,149 to 55,138,636)	2	42,509,703 (36,337,918 to 49,482,918)	4	-0.78 (-0.98 to -0.58)*	-1.17 (-1.53 to -0.82)*	-0.46 (-0.68 to -0.24)*
Other environmental risks	5,386,702 (3,749,375 to 7,246,194)	14	6,306,070 (4,075,171 to 8,773,453)	13	0.85 (0.72 to 0.98)*	1.14 (0.91 to 1.38)*	0.46 (0.38 to 0.53)*
Child and maternal malnutrition	27,730,286 (24,920,872 to 30,776,034)	5	7,253,783 (6,277,469 to 8,340,678)	12	-6.81 (-7.62 to -5.99)*	-8.92 (-9.38 to -8.47)*	-4.75 (-6.37 to -3.10)*
Tobacco	57,264,585 (52,395,093 to 62,486,440)	1	64,116,000 (54,575,903 to 75,181,983)	1	0.60 (0.42 to 0.78)*	0.30 (0.02 to 0.58)*	0.90 (0.66 to 1.15)*
Alcohol use	14,673,585 (12,536,787 to 17,097,523)	8	17,265,060 (13,765,337 to 21,321,000)	8	0.77 (0.68 to 0.85)*	0.77 (0.68 to 0.85)*	0.77 (0.68 to 0.85)*
Drug use	6,301,278 (5,433,686 to 7,296,963)	13	5,121,794 (4,187,484 to 6,121,198)	14	-1.03 (-1.22 to -0.84)*	-3.28 (-3.50 to -3.07)*	1.54 (1.21 to 1.87)*
High fasting plasma glucose	17,924,145 (14,527,382 to 21,999,850)	7	28,228,439 (22,052,698 to 35,375,022)	5	2.36 (1.82 to 2.90)*	3.64 (2.83 to 4.47)*	1.11 (0.41 to 1.81)*
High systolic blood pressure	35,341,494 (30,227,215 to 40,383,079)	3	54,441,615 (45,474,002 to 63,689,070)	2	2.32 (2.18 to 2.46)*	2.66 (2.40 to 2.91)*	1.84 (1.77 to 1.92)*
High body-mass index	11,704,488 (3,880,854 to 22,527,461)	9	24,830,041 (11,788,976 to 40,545,899)	6	4.02 (3.74 to 4.29)*	4.33 (3.83 to 4.83)*	3.59 (3.48 to 3.70)*
Low bone mineral density	1,769,378 (1,477,619 to 2,061,186)	16	3,320,275 (2,594,480 to 4,004,265)	15	3.39 (3.12 to 3.67)*	4.14 (3.73 to 4.56)*	2.78 (2.42 to 3.15)*
Dietary risks	34,253,322 (27,427,086 to 43,429,953)	4	46,813,126 (35,644,728 to 60,011,858)	3	1.66 (1.53 to 1.78)*	1.97 (1.74 to 2.21)*	1.28 (1.21 to 1.35)*
Low physical activity	1,275,222 (632,556 to 2,484,126)	18	2,507,120 (1,195,139 to 4,842,346)	17	3.54 (2.70 to 4.38)*	3.88 (2.39 to 5.39)*	3.03 (2.65 to 3.41)*
Occupational risks	18,571,331 (16,040,002 to 21,379,719)	6	15,486,849 (12,897,016 to 18,259,216)	9	-0.97 (-1.12 to -0.82)*	-1.51 (-1.78 to -1.24)*	-0.40 (-0.52 to -0.29)*
Intimate partner violence	789,841 (433,187 to 1,234,095)	19	789,926,926 (354,381 to 1,304,433)	19	0.07 (-0.17 to 0.30)	-0.50 (-0.78 to -0.22)*	0.76 (0.39 to 1.14)*
Unsafe sex	1,482,696 (1,293,280 to 1,925,395)	17	2,724,797 (1,976,323 to 3,277,196)	16	3.20 (2.90 to 3.49)*	3.72 (3.50 to 3.94)*	2.95 (2.39 to 3.51)*
Non-optimal temperature	8,427,319 (7,191,510 to 9,779,073)	12	9,325,364 (7,641,727 to 11,224,758)	11	0.44 (-0.88 to 1.77)	-0.61 (-1.39 to 0.18)	1.14 (-1.01 to 3.34)
Kidney dysfunction	8,884,203 (7,881,193 to 10,012,008)	11	13,353,909 (11,150,932 to 15,771,793)	10	2.25 (1.83 to 2.67)*	3.04 (2.37 to 3.71)*	1.47 (0.91 to 2.03)*
High LDL cholesterol	11,217,788 (9,135,992 to 13,780,576)	10	19,813,962 (15,205,317 to 25,139,359)	7	3.11 (2.85 to 3.36)*	3.86 (3.39 to 4.33)*	2.23 (2.08 to 2.37)*
Childhood sexual abuse and bullying	762,744 (311,431 to 1,443,447)	20	646,857,857 (278,772 to 1,183,126)	20	-0.86 (-0.97 to -0.75)*	-1.17 (-1.29 to -1.04)*	-0.40 (-0.58 to -0.22)*
**Age-standardized rate**							
Unsafe water, sanitation, and handwashing	475 (376 to 587)	14	91 (64 to 122)	18	-8.23 (-8.79 to -7.67)*	-11.69 (-12.52 to -10.85)*	-4.58 (-5.22 to -3.93)*
Air pollution	4,860 (4,243 to 5,438)	2	2,265 (1,950 to 2,615)	4	-3.93 (-4.15 to -3.70)*	-4.08 (-4.49 to -3.68)*	-3.86 (-3.97 to -3.74)*
Other environmental risks	488 (338 to 653)	13	331 (213 to 460)	13	-2.00 (-2.13 to -1.87)*	-1.49 (-1.73 to -1.25)*	-2.64 (-2.71 to -2.56)*
Child and maternal malnutrition	3,285 (2,955 to 3,649)	4	786 (690 to 899)	10	-7.24 (-7.44 to -7.04)*	-8.34 (-8.58 to -8.10)*	-6.13 (-6.41 to -5.85)*
Tobacco	5,287 (4,858 to 5,765)	1	3,228 (2,759 to 3,767)	1	-2.57 (-2.83 to -2.32)*	-2.80 (-3.20 to -2.40)*	-2.33 (-2.68 to -1.98)*
Alcohol use	1,137 (972 to 1,328)	8	908 (729 to 1,116)	8	-1.18 (-1.50 to -0.86)*	-1.41 (-1.54 to -1.28)*	-0.92 (-1.56 to -0.27)*
Drug use	449 (390 to 518)	15	295 (238 to 361)	14	-2.11 (-2.74 to -1.48)*	-4.70 (-4.97 to -4.43)*	0.85 (-0.48 to 2.19)
High fasting plasma glucose	1,647 (1,338 to 2,051)	6	1,452 (1,139 to 1,832)	5	-0.72 (-1.23 to -0.20)*	0.72 (-0.06 to 1.51)	-2.11 (-2.76 to -1.44)*
High systolic blood pressure	3,304 (2,820 to 3,777)	3	2,844 (2,392 to 3,321)	2	-0.77 (-0.89 to -0.65)*	-0.29 (-0.51 to -0.07)*	-1.40 (-1.47 to -1.34)*
High body-mass index	962 (315 to 1,891)	10	1,231 (578 to 2,023)	6	1.28 (0.95 to 1.60)*	1.50 (0.91 to 2.10)*	0.94 (0.82 to 1.07)*

For peer review only - http://bmjopen.bmj.com/site/about/guidelines.xhtml

Low bone mineral density	164 (137 to 192)	16	177 (138 to 214)	15	0.40 (0.27 to 0.54)*	0.70 (0.55 to 0.86)*	0.20 (-0.02 to 0.42)
Dietary risks	3,022 (2,391 to 3,875)	5	2,394 (1,823 to 3,071)	3	-1.23 (-1.36 to -1.09)*	-0.78 (-1.03 to -0.52)*	-1.75 (-1.83 to -1.68)*
Low physical activity	149 (74 to 287)	17	146 (70 to 280)	16	-0.19 (-0.74 to 0.36)	0.48 (0.17 to 0.79)*	-0.93 (-2.03 to 0.18)
Occupational risks	1,482 (1,277 to 1,700)	7	844 (701 to 997)	9	-2.95 (-3.08 to -2.81)*	-3.67 (-3.88 to -3.45)*	-2.22 (-2.37 to -2.07)*
Intimate partner violence	55 (30 to 86)	19	46 (22 to 76)	20	-0.86 (-1.11 to -0.62)*	-1.68 (-1.94 to -1.41)*	0.09 (-0.32 to 0.50)
Unsafe sex	108 (94 to 141)	18	142 (106 to 170)	17	1.47 (0.88 to 2.06)*	1.83 (1.43 to 2.23)*	1.34 (0.19 to 2.51)*
Non-optimal temperature	936 (813 to 1,075)	11	483 (399 to 582)	12	-3.61 (-4.04 to -3.18)*	-4.39 (-5.06 to -3.71)*	-2.91 (-3.41 to -2.42)*
Kidney dysfunction	781 (690 to 882)	12	709 (593 to 832)	11	-0.45 (-0.97 to 0.08)	054 (-0.31 to 1.39)	-1.41 (-2.10 to -0.72)*
High LDL cholesterol	1,005 (790 to 1,284)	9	1,052 (800 to 1,345)	7	0.28 (0.10 to 0.45)*	1.19 (0.87 to 1.51)*	-0.75 (-0.85 to -0.66)*
Childhood sexual abuse and bullying	54 (22 to 102)	20	50 (20 to 91)	19	-0.41 (-0.51 to -0.31)*	-1.05 (-1.21 to -0.89)*	0.40 (0.31 to 0.48)*

Note: AAPC: average annual percent changes; UI, uncertainty interval; CI, confidence interval; LDL, low-density lipoprotein.
*AAPC significantly differs from zero at the alpha = 0.05 level.

For peer review only

STROBE Statement—Checklist of items that should be included in reports of ***cohort studies***

	Item No	Recommendation
**Title and abstract**	1	(*a*) Indicate the study's design with a commonly used term in the title or the abstract. (Line 24-25, page 2)
		(*b*) Provide in the abstract an informative and balanced summary of what was done and what was found. (Line 30-35, page 2)
**Introduction**		
Background/rationale	2	Explain the scientific background and rationale for the investigation being reported. (Line67-69, page 4)
Objectives	3	State specific objectives, including any prespecified hypotheses. (Line88-91, page 4-5)
**Methods**		
Study design	4	Present key elements of study design early in the paper
Setting	5	Describe the setting, locations, and relevant dates, including periods of recruitment, exposure, follow-up, and data collection
Participants	6	(*a*) Give the eligibility criteria, and the sources and methods of selection of participants. Describe methods of follow-up
		(*b*) For matched studies, give matching criteria and number of exposed and unexposed
Variables	7	Clearly define all outcomes, exposures, predictors, potential confounders, and effect modifiers. Give diagnostic criteria, if applicable
Data sources/ measurement	8*	For each variable of interest, give sources of data and details of methods of assessment (measurement). Describe comparability of assessment methods if there is more than one group. (Line 94-112, page 5-6)
Bias	9	Describe any efforts to address potential sources of bias
Study size	10	Explain how the study size was arrived at
Quantitative variables	11	Explain how quantitative variables were handled in the analyses. If applicable, describe which groupings were chosen and why
Statistical methods	12	(*a*) Describe all statistical methods, including those used to control for confounding. (Line121-128, page 6)
		(*b*) Describe any methods used to examine subgroups and interactions. (Line128-130, page 6)
		(*c*) Explain how missing data were addressed
		(*d*) If applicable, explain how loss to follow-up was addressed
		(*e*) Describe any sensitivity analyses
**Results**		
Participants	13*	(a) Report numbers of individuals at each stage of study—eg numbers potentially eligible, examined for eligibility, confirmed eligible, included in the study, completing follow-up, and analysed.
		(b) Give reasons for non-participation at each stage. (Line138-139， page 7 and line 162-163, page 10 and line 184-186 page 13)
		(c) Consider use of a flow diagram
Descriptive data	14*	(a) Give characteristics of study participants (eg demographic, clinical, social) and information on exposures and potential confounders
		(b) Indicate number of participants with missing data for each variable of interest
		(c) Summarise follow-up time (eg, average and total amount)
Outcome data	15*	Report numbers of outcome events or summary measures over time. (Line 155-

For peer review only - http://bmjopen.bmj.com/site/about/guidelines.xhtml

		158，page 7-8 and line 177-180, page 10 and line 223-228, page 14-15 and line 243-247, page 15)
Main results	16	(*a*) Give unadjusted estimates and, if applicable, confounder-adjusted estimates and their precision (eg, 95% confidence interval). Make clear which confounders were adjusted for and why they were included
		(*b*) Report category boundaries when continuous variables were categorized
		(*c*) If relevant, consider translating estimates of relative risk into absolute risk for a meaningful time period
Other analyses	17	Report other analyses done—eg analyses of subgroups and interactions, and sensitivity analyses
**Discussion**		
Key results	18	Summarise key results with reference to study objectives. (line308-315, page22)
Limitations	19	Discuss limitations of the study, taking into account sources of potential bias or imprecision. Discuss both direction and magnitude of any potential bias. (line332-346, page22-23)
Interpretation	20	Give a cautious overall interpretation of results considering objectives, limitations, multiplicity of analyses, results from similar studies, and other relevant evidence
Generalisability	21	Discuss the generalisability (external validity) of the study results
**Other information**		
Funding	22	Give the source of funding and the role of the funders for the present study and, if applicable, for the original study on which the present article is based. (line369-372, page25)

*Give information separately for exposed and unexposed groups.

Note: An Explanation and Elaboration article discusses each checklist item and gives methodological background and published examples of transparent reporting. The STROBE checklist is best used in conjunction with this article (freely available on the Web sites of PLoS Medicine at http://www.plosmedicine.org/, Annals of Internal Medicine at http://www.annals.org/, and Epidemiology at http://www.epidem.com/). Information on the STROBE Initiative is available at http://www.strobe-statement.org.

For peer review only

For peer review only - http://bmjopen.bmj.com/site/about/guidelines.xhtml

BMJ Open

Temporal trend of population structure, burden of diseases, healthcare resources and expenditure in China, 2000-2019

Journal:	*BMJ Open*
Manuscript ID	bmjopen-2022-062091.R2
Article Type:	Original research
Date Submitted by the Author:	07-Sep-2022
Complete List of Authors:	Liang, Zhenguo; Third Affiliated Hospital of Sun Yat-Sen University, Department of Rheumatology<br>WU, Dongze; Third Affiliated Hospital of Sun Yat-Sen University, Department of Rheumatology<br>Guo, Cui; The Chinese University of Hong Kong<br>Gu, Jieruo; Third Affiliated Hospital of Sun Yat-Sen University, Department of Rheumatology
**Primary Subject Heading**:	Health policy
Secondary Subject Heading:	Global health, Health policy
Keywords:	Health economics < HEALTH SERVICES ADMINISTRATION & MANAGEMENT, Health policy < HEALTH SERVICES ADMINISTRATION & MANAGEMENT, Health & safety < HEALTH SERVICES ADMINISTRATION & MANAGEMENT

SCHOLARONE™
Manuscripts

I, the Submitting Author has the right to grant and does grant on behalf of all authors of the Work (as defined in the below author licence), an exclusive licence and/or a non-exclusive licence for contributions from authors who are: i) UK Crown employees; ii) where BMJ has agreed a CC-BY licence shall apply, and/or iii) in accordance with the terms applicable for US Federal Government officers or employees acting as part of their official duties; on a worldwide, perpetual, irrevocable, royalty-free basis to BMJ Publishing Group Ltd ("BMJ") its licensees and where the relevant Journal is co-owned by BMJ to the co-owners of the Journal, to publish the Work in this journal and any other BMJ products and to exploit all rights, as set out in our licence.

The Submitting Author accepts and understands that any supply made under these terms is made by BMJ to the Submitting Author unless you are acting as an employee on behalf of your employer or a postgraduate student of an affiliated institution which is paying any applicable article publishing charge ("APC") for Open Access articles. Where the Submitting Author wishes to make the Work available on an Open Access basis (and intends to pay the relevant APC), the terms of reuse of such Open Access shall be governed by a Creative Commons licence – details of these licences and which Creative Commons licence will apply to this Work are set out in our licence referred to above.

Other than as permitted in any relevant BMJ Author's Self Archiving Policies, I confirm this Work has not been accepted for publication elsewhere, is not being considered for publication elsewhere and does not duplicate material already published. I confirm all authors consent to publication of this Work and authorise the granting of this licence.

For peer review only

Temporal trend of population structure, burden of diseases, healthcare resources and expenditure in China, 2000-2019

Authors

Zhenguo Liang[1#], Dongze Wu, PhD[1#], Cui Guo[2#], PhD, Jieruo Gu, PhD[1*]

Institution

1.Department of Rheumatology, The Third Affiliated Hospital of Sun Yat-sen University, Guangzhou, China

2.Jockey Club School of Public Health and Primary Care, The Chinese University of Hong Kong, Hong Kong SAR, China

[#]Contributed equally to the manuscript.

Corresponding authors

[*]Corresponding to Prof. Jieruo Gu.

Prof. Jieguo Gu, PhD

Address: No. 600 Tianhe Road, Tianhe District, Guangzhou, Guangdong, China, 510000

E-mail: gujieruo@mail.sysu.edu.cn

Telephone: (+86) 2085253333 / Fax number: (+86) 2085253336

Running Head: Population ageing in China

Keywords: Trend, Population, Heath Care, Disease Burden, China

Word Count: 3970 Reference:44 Table Count: 4 Figure Count:1

For peer review only - http://bmjopen.bmj.com/site/about/guidelines.xhtml

Abstract

Objectives

To explore the evolutionary trend of population structure, disease burden, healthcare resources and expenditure in China, and to identify key domains that are most in need of intervention.

Design

A cross-sectional and longitudinal analysis

Data source

Population and healthcare data from China Statistical Yearbook (CSY), and disease burden attributable to causes and risk factors from the Global Burden of Diseases (GBD) between 2000 and 2019.

Measures and methods

We used the Joinpoint Regression Program to measure trends in population composition, population change, dependency ratio, healthcare institution, personnel, expenditure, disease burden from 2000 to 2019.

Results

Regarding the population in China between 2000 and 2019, a decreasing trend was observed among youth aged 0–14 years (AAPC: -1.17), a slow rising trend was observed among individuals aged 15–64 years (AAPC: 1.10), and a rapidly increasing trend was observed among individuals older than 65 years (AAPC: 3.67). Astonishing increasing trends in healthcare institutions (AAPC: 3.97), medical personnel (AAPC: 3.26), and healthcare expenditures (AAPC: 15.28) were also observed. Among individuals younger than 70 years, neoplasms (AAPC: 0.54) and cardiovascular diseases (AAPC: 0.67) remained among the top three causes, while tobacco (AAPC: 0.22)

For peer review only - http://bmjopen.bmj.com/site/about/guidelines.xhtml

remained a top three risk factor. However, while musculoskeletal disorders (AAPC: 1.88) were not a top three cause in 2000, they are a top three cause in 2019.

Conclusion

Comprehensive age-cause-risk factor-specific strategies are key to reconcile the tension among triangle of population ageing, disease burden and healthcare expenditure. The disease burden from cardiometabolic diseases, neoplasms and musculoskeletal disorders were identified as key domains that require intervention to reduce an increasing disease burden among individuals currently older than 70 years, as well as those approaching this age group.

Keywords: Trend, Population, Heath Care, Disease Burden, China

Strengths and limitations of this study

- This study characterized the temporal trend of population structure and composition, health resources and expenditures from China Statistical Yearbook, expectancy, and disease burden attributable to 22 causes and 20 risk factors from Global Burden of Disease study in China from 2000 to 2019.
- Population composition of population (0-14), working population (15-64), post-working population (65+) were used to reflect trend of working population and population ageing.
- The analysis of disease burden in population younger and older than 70 years aimed to identify the cause or risk factor (driver) which has the greatest impact on the trend of disease burden attributable to 22 causes and 20 risk factors from 2000 to 2019.
- The principal limitation of this study is unavailability of provincial level data in disease burden, population, and healthcare and inability to provide granular policy recommendation.

For peer review only - http://bmjopen.bmj.com/site/about/guidelines.xhtml

For peer review only

Introduction

Together with the world's population, people in China struggle against cancer and aging despite efforts to extend lifespan and maximize healthspan[1, 2]. Since 1950, the lifespan at birth has been extended by 26 years in China, while the total fertility rate has decreased from 5.91 to 1.43[3]. Between 2000 and 2019, the life expectancy for males has increased from 69.1 to 74.4 years, while the life expectancy for females has increased from 73.9 to 80.8 years[3]. It has been forecasted that China will become the largest economy by 2035, and that the population in China will decline by 48.0% from 2017 to 2100[4]. Moreover, in the next 20 years, the universal two-to-three-child policy may not reverse a shrinking workforce and rapid population ageing, especially in rural areas[5, 6]. In 2009, China expanded social health insurance coverage to its entire population and reformed its health care delivery system[7, 8].

Health care systems coordinate complex interrelationships among individuals who receive and provide medical financial support[9]. It would have been difficult for China to rigorously control the two pandemics that occurred in 2003 and 2019 without a healthcare system. In particular, an increasing number of modernized medical institutions and well-educated personnel have been established over the past two decades in China[10, 11]. Accelerating rural-to-urban migration and corresponding increases in urban populations have also both led to improved access to health care, although substantial health risks can accompany changes in human activities, diets, and social structures[12]. Although two GBD studies have assessed the disease burden and risk factor at provincial level in China[13, 14], the study further to evaluate healthcare professional and expenditure and to identify key domain to mitigate the aging driven disease burden.

Ageing society in China continue to exacerbate the burden borne by current family and public healthcare systems, which make it is necessary to make long-term strategic plans to respond to the

For peer review only - http://bmjopen.bmj.com/site/about/guidelines.xhtml

pressures of an ageing society at the governmental, individual, and technologic level[15]. There are a very low spatial and temporal matching degrees between the population-ageing rate and the number of medical resources per thousand residents in China, and the geographical pattern of the temporal matching between them exhibited a feature of north-south differentiation [16]. China does not have a typical disease characteristic of an aging society comparing the characteristics of the aging population in China with those in the world[17]. Namely, China faces the dual threat of non-communicable diseases and communicable diseases, and the former account for most of the age-related disease burden. Although there are great number of studies centering on trend of population, burden of disease, healthcare in China, but they failed to dissect the implication of a central tension among 3 mutually conflicting forces: intractable disease burden, population ageing, and upsurging healthcare expenditure. The study tries to find a solution to reconcile the three contradictory forces, achieving longer healthy lifespan in the ageing society at high-cost performance of health expenditure. Possible driving forces of disease burden among ageing populations were also examined to provide a basis for targeted interventions, such as early disease prevention and health management among older populations.

Methods

Data sources

The National Bureau of Statistics (NBS) of China annually publishes the China Statistical Yearbook (CSY)[18]. The Department of Population and Employment Statistics of the NBS conducts statistical population surveys as follows. A national population census is conducted in years ending with 0; a national 1 percent population sample survey is conducted in years ending with 5; and sample surveys on population changes are conducted in the remaining years. The latter

For peer review only - http://bmjopen.bmj.com/site/about/guidelines.xhtml

covers about 1 per thousand of the total population of China. The sample surveys on population change take the entire nation of China as the population, while each province, autonomous region, or municipality represents a sub-population. A stratified multi-stage systematic probability-proportional-to-size (PPS) cluster sampling scheme is used. Data regarding population, healthcare institutions, personnel, beds, and health expenditures are described in detail in the Supplemental Appendix (including a glossary and definitions).

The Global Burden of Disease（GBD）2019 study assessed life expectancy, disease burden, risk factor, etiology, and impairment[3, 19, 20]. All-cause incidence, prevalence, death, years lived with disability (YLD), years of life lost (YLL), disability-adjusted life years (DALYs), and attributable risk factors were assessed between 1990 and 2019. The study identified twenty-two level 2 leading causes and twenty level 2 leading risk factors. Both causes and risk factors were assessed according to number, rate, and age-standardized rate of DALYs. The estimation methods and data for burden of disease published in GBD 2019 were used to assess disease burden in China between 2000 and 2019 for the present study.

Aggregated data of population and healthcare from CSY and aggregated data of life expectancy, disease burden attributable to 22 causes or 20 risk factors from GBD between 2000 and 2019 were used to quantify temporal trend of population structure, burden of diseases, healthcare resources and expenditure in China.

Outcomes of measurements

The primary outcomes are temporal trend in population (population composition, population change, dependency ratio), healthcare (number of healthcare institution, number of health personnel, healthcare expenditure), life expectancy (healthy and total), disease burden attributable to 22 causes or 20 risk factors (disability adjusted life years, DALYs) from 2000 to 2019. The

For peer review only - http://bmjopen.bmj.com/site/about/guidelines.xhtml

secondary outcomes are i) the main drivers of disease burden from 22 causes or 20 risk factors in individuals younger and older than 70, ii) temporal trend in population, healthcare, and disease burden attributable to 22 causes or 20 risk factors from 2000 to 2009, and from 2010 to 2019. Driver refers the cause or risk factor that have the greatest impact on the total disease burden attributable to 22 causes or 20 risk factors from 2000 to 2019.

Statistical analysis

The population composition, change, dependency ratio, healthcare institution, personnel, and expenditures in terms of number, disease burden and risk factors in terms of number and rate, were assessed by using a Joinpoint regression model. The disease burden and risk factor in individuals younger and older than 70 years in term of number and rate were also assessed to identify the driving causes and risk factors. Average annual percent change (AAPC) and associated 95% confidence intervals (CIs) were calculated for the study period. Temporal increasing and decreasing trends were defined according to the statistical significance of the AAPC compared to zero. AAPC values with 95% CIs overlapping with zero were considered stable. Two segmented line regression with joinpoint of 2000, 2009, and 2019 were calculated to show the temporal trend from 2000 to 2009, from 2010 to 2019. All statistical analyses were performed by using the Joinpoint Regression Program (version 4.8.0.1, Statistical Methodology and Applications Branch, Surveillance Research Program, National Cancer Institute, Bethesda, MD, USA). P-values less than 0.05 were considered statistically significant at a two-tailed level.

Patient and public involvement statement

Patients and the public were not involved in any way in this research.

Results

Population trends between 2000 and 2019

For peer review only - http://bmjopen.bmj.com/site/about/guidelines.xhtml

Initially, temporal trends of the population structure in China were examined based on CSY data (gender and age composition, birth rate, and dependency ratio). From 2000 through 2019, an increasing trend in total population (AAPC: 0.52, 95% CI: 0.51 to 0.54), male population (AAPC: 0.47, 95% CI: 0.44 to 0.51), and female population (AAPC: 0.59, 95% CI: 0.56 to 0.62) were observed. As expected, a striking contrary trend in urban population (AAPC: 3.28, 95%CI: 3.18 to 3.38) and rural population (AAPC: -1.98, 95%CI: -2.02 to -1.95) were observed. We further identified that an increasing trend in urban population was attenuated, while a decreasing trend in rural population was aggravated, during 2010–2019 compared to 2000–2009 (Figure 1A, Table 1).

A clear decreasing trend in the younger population (ages 0–14 years) was observed in 2000–2019 (AAPC: -1.17, 95%CI: -1.78 to -0.55), especially in 2000–2009. An increasing trend in the working population (ages 15–64 years) was observed in 2000–2009 (AAPC: 1.10, 95%CI: 1.03 to 1.18), and then it gradually disappeared over 2010–2019. In contrast, an increasing trend in the post-working population (65+ years) that was observed in 2000–2019 (AAPC: 3.67, 95%CI: 3.51 to 3.82) gradually intensified from 2000–2009 to 2010–2019 (Figure 1B, Table 1).

From 2000 to 2019, the birth rate (AAPC: -1.53, 95%CI: -2.31 to -0.75) and natural growth rate (AAPC: -4.20, 95%CI: -5.52 to -2.87) continued to decrease. Conversely, from 2000 to 2009, the death rate continued to increase (AAPC: 1.10, 95%CI: 0.75 to 1.46), yet from 2010–2019 it remained stable (AAPC: 0.03, 95%CI: -0.06 to 0.13) (Figure 1C, Table 1). The past two decades also exhibited a reverse trend in gross dependency ratio from 2010–2019, a muted decreasing trend in children dependency ratio from 2000–2009 to 2010–2019, and a strengthened increasing trend in old dependency ratio from 2000–2009 to 2010–2019 (Figure 1D, Table 1).

For peer review only

For peer review only - http://bmjopen.bmj.com/site/about/guidelines.xhtml

Table 1. AAPC in population composition, change, and dependency ratio between 2000 and 2019

Subcategory	Number			AAPC (95% CI)		
	2000	2009	2019	2000–2019	2000–2009	2010–2019
**Population composition by gender and area (10000 persons)**						
Total Population	126,743	133,450	140,005	0.52 (0.51 to 0.54)*	0.57 (0.56 to 0.58)*	0.48 (0.45 to 0.51)*
Male Population	65,437	68,647	71,527	0.47 (0.44 to 0.51)*	0.53 (0.50 to 0.57)*	0.43 (0.37 to 0.49)*
Female Population	61,306	64,803	68,478	0.59 (0.56 to 0.62)*	0.62 (0.56 to 0.67)*	0.56 (0.55 to 0.57)*
Urban Population	45,906	64,512	84,843	3.28 (3.18 to 3.38)*	3.86 (3.75 to 3.98)*	2.68 (2.51 to 2.84)*
Rural Population	80,837	68,938	55,162	-1.98 (-2.02 to -1.95)*	-1.79 (-1.86 to -1.73)*	-2.15 (-2.18 to -2.12)*
**Population composition by age group (10000 persons)**						
Population Aged 0–14 y	29,012	24,659	23,492	-1.17 (-1.78 to -0.55)*	-2.24 (-2.76 to -1.71)*	0.31 (-0.23 to 0.85)
Population Aged 15–64 y	88,910	97,484	98,910	0.59 (0.52 to 0.66)*	1.10 (1.03 to 1.18)*	0.02 (-0.10 to 0.14)
Population Aged 65+ y	8,821	11,307	17,603	3.67 (3.51 to 3.82)*	2.81 (2.66 to 2.96)*	4.51 (4.25 to 4.76)*
**Population change (%)**						
Birth Rate	14.03	11.95	10.48	-1.53 (-2.31 to -0.75)*	-1.63 (-2.32 to -0.93)*	-1.57 (-3.03 to -0.09)*
Death Rate	6.45	7.08	7.14	0.54 (0.37 to 0.71)*	1.10 (0.75 to 1.46)*	0.03 (-0.06 to 0.13)
Natural Growth Rate	7.58	4.87	3.34	-4.20 (-5.52 to -2.87)*	-4.64 (-5.69 to -3.57)*	-4.40 (-6.93 to -1.80)*
**Dependency ratio (%)**						
Gross Dependency Ratio	42.6	36.9	41.5	-0.25 (-0.54 to 0.03)	-1.97 (-2.26 to -1.68)*	1.70 (1.21 to 2.19)*
Children Dependency Ratio	32.6	25.3	23.8	-1.76 (-2.54 to -0.96)*	-3.33 (-4.00 to -2.66)*	0.33 (-0.37 to 1.03)
Old Dependency Ratio	9.9	11.6	17.8	3.12 (2.93 to 3.31)*	1.76 (1.45 to 2.08)*	4.49 (4.27 to 4.70)*

AAPC: average annual percent change; CI, confidence interval. *AAPC significantly differs from zero at a two-tailed level of 0.05.

For peer review only

For peer review only - http://bmjopen.bmj.com/site/about/guidelines.xhtml

Health resources and expenditure between 2000 and 2019

Temporal trends in health sources, including institutions, personnel, and expenditures, were examined based on CSY data. A consistent increasing trend in number of hospitals (AAPC: 3.97, 95%CI: 3.69 to 4.24), including general hospitals, hospitals that specialize in traditional Chinese medicine, and specialized hospitals, was observed. The number of medical personnel (AAPC: 3.26, 95%CI: 2.76 to 3.77), including medical technical personnel, licensed assistant doctors, licensed doctors, and registered nurses, also continued to increase in 2000–2019. Moreover, the rate of increase of number of medical personnel was higher in 2010–2019 than in 2000–2009. The number of beds in health care institutions (AAPC: 5.54, 95%CI: 5.20 to 5.88), including hospitals, township health service centers, specialized public health institutions, and women and children care agencies, also increased in 2000–2019. Like medical personnel, the rate increase for beds in health care institutions was greater in 2010–2019 than in 2000–2009 (Table 2).

Total healthcare expenditure increased from ¥458.66 billion (US$ 55.40 billion) in 2000 to ¥6584.14 billion (US$ 954.68 billion) in 2019, with an AAPC of 15.28 (95%CI: 14.64 to 15.91). The per capita health expenditure increased from ¥361.88 in 2000 to ¥4702.79 in 2019, with an AAPC of 14.67 (95%CI: 14.04 to 15.30). Striking increasing trends in government health expenditure (AAPC: 18.95, 95%CI: 16.72 to 21.22), social health expenditure (AAPC: 19.03, 95%CI: 17.56 to 20.52), and out-of-pocket health expenditure (AAPC: 10.62, 95%CI: 9.24 to 12.02) were also observed in 2000–2019 (Table 2).

For peer review only - http://bmjopen.bmj.com/site/about/guidelines.xhtml

Table 2. AAPC in healthcare institution, personnel, and expenditures between 2000 and 2019

Subcategory	Number			AAPC (95% CI)		
	2000	2009	2019	2000—2019	2000—2009	2010—2019
**Health care institution (unit)**						
Number of Hospitals	16,318	20,291	34,354	3.97 (3.69 to 4.24)*	2.41 (2.03 to 2.80)*	5.72 (5.25 to 6.19)*
Number of General Hospitals	11,872	13,364	19,963	2.84 (2.56 to 3.13)*	1.37 (0.97 to 1.77)*	4.51 (4.03 to 4.99)*
Number of Hospitals Specialized in Traditional Chinese Medicine	2,453	2,728	4,221	2.92 (2.50 to 3.34)*	1.35 (1.20 to 1.50)*	4.69 (3.82 to 5.58)*
Number of Specialized Hospitals	1,543	3,716	8,531	9.65 (9.02 to 10.28)*	10.98 (9.69 to 12.28)*	8.46 (8.25 to 8.68)*
Number of Health Care Institutions at Grass-root Level	1,000,169	882,153	954,390	-0.45 (-0.82 to -0.08)*	-1.88 (-2.60 to -1.15)*	0.85 (0.67 to 1.04)*
Number of Township Health Service Centers	49,229	38,475	36,112	-1.63 (-1.83 to -1.44)*	-2.79 (-3.10 to -2.47)*	-0.47 (-0.66 to -0.28)*
Number of Village Clinics	709,458	632,770	616,094	-1.03 (-1.40 to -0.65)*	-1.88 (-2.53 to -1.23)*	-0.51 (-0.86 to -0.17)*
Number of Outpatient Department	240,934	182,448	266,659	0.42 (-0.18 to 1.03)	-2.73 (-3.45 to -2.01)*	4.05 (3.03 to 5.07)*
Number of Specialized Public Health Institutions	11,386	11,665	15,958	1.63 (-0.23 to 3.54)	0.12 (-0.68 to 0.93)	3.35 (-0.52 to 7.36)
Number of Center for Disease Control and Prevention	3,741	3,536	3,403	-0.40 (-0.50 to -0.29)*	-0.40 (-0.50 to -0.29)*	-0.40 (-0.50 to -0.29)*
Number of Specialized Disease Prevention & Treatment Institution	1,839	1,291	1,128	-2.47 (-2.97 to -1.98)*	-3.61 (-4.46 to -2.76)*	-1.49 (-2.05 to -0.93)*
Number of Women and Children Care Agencies	3,163	3,020	3,071	-0.11 (-0.29 to 0.08)	-0.45 (-0.80 to -0.10)*	0.20 (0.09 to 0.32)*
**Medical Personnel (10000 persons)**						
Number of Medical Personnel	691.04	778.14	1292.83	3.26 (2.76 to 3.77)*	1.15 (0.16 to 2.14)*	5.16 (4.87 to 5.45)*
Number of Medical Technical Personnel	449.08	553.51	1015.40	4.34 (3.99 to 4.68)*	2.30 (1.61 to 3.01)*	6.20 (6.07 to 6.34)*
Number of Licensed (Assistant) Doctors	207.58	232.92	386.69	3.12 (2.64 to 3.59)*	0.92 (0.05 to 1.81)*	5.36 (4.98 to 5.74)*
Number of Licensed Doctors	160.33	190.54	321.05	3.53 (3.12 to 3.95)*	1.50 (0.73 to 2.27)*	5.62 (5.29 to 5.95)*
Number of Registered Nurse	126.68	185.48	444.50	6.79 (6.20 to 7.38)*	4.37 (3.26 to 5.49)*	8.92 (8.49 to 9.34)*
Number of Pharmacist	41.44	34.19	48.34	0.76 (0.23 to 1.29)*	-2.28 (3.31 to -1.23)*	3.57 (3.27 to 3.88)*
Number of Village Doctors and Assistants	131.94	105.10	84.23	-2.49 (-4.28 to -0.66)*	-3.44 (-6.25 to -0.55)*	-2.16 (-4.10 to -0.17)*
Number of Other Technical Personnel	15.75	27.50	50.39	6.30 (6.07 to 6.53)*	6.30 (6.07 to 6.53)*	6.30 (6.07 to 6.53)*
Number of Administrative Personnel	42.68	36.27	54.38	1.25 (0.42 to 2.09)*	-1.74 (-3.27 to -0.18)*	4.01 (3.49 to 4.54)*
Number of Logistics Technical Workers	51.59	55.77	88.43	2.95 (2.13 to 3.78)*	0.84 (-0.26 to 1.94)	4.71 (3.57 to 5.86)*
**Beds in Health Care Institutions**						
Number of Beds in Health Care Institutions (10000 units)	317.70	441.66	880.70	5.54 (5.20 to 5.88)*	3.60 (3.01 to 4.20)*	7.11 (6.82 to 7.41)*
Number of Beds in Hospitals (10000 beds)	216.67	312.08	686.65	6.35 (5.96 to 6.74)*	4.17 (3.55 to 4.81)*	8.16 (7.74 to 8.59)*
Number of Beds in Community Health Service Centers (10000 units)	76.65	109.98	163.11	3.96 (2.90 to 5.04)*	3.73 (1.68 to 5.83)*	3.28 (2.75 to 3.82)*
Number of Beds in Township Health Service Centers (10000 beds)	73.48	93.34	136.99	3.29 (2.18 to 4.41)*	2.38 (0.26 to 4.54)*	3.48 (2.93 to 4.04)*

For peer review only

For peer review only - http://bmjopen.bmj.com/site/about/guidelines.xhtml

Number of Beds in Specialized Public Health Institutions (10000 units)	11.86	15.40	28.50	4.80 (4.31 to 5.28)*	2.97 (2.52 to 3.42)*	6.24 (5.55 to 6.93)*
Number of Beds in Women and Children Care Agencies (10000 units)	7.12	12.61	24.32	6.79 (6.41 to 7.18)*	6.65 (6.15 to 7.15)*	6.74 (6.25 to 7.24)*
Number of Beds in Specialized Disease Prevention & Treatment Institution (10000 beds)	2.84	2.71	4.11	2.29 (0.21 to 4.41)*	0.19 (-3.08 to 3.57)	3.75 (1.61 to 5.95)*
**Health Expenditure (100 million yuan)**						
Total Health Expenditure	4586.63	17541.92	65841.39	15.28 (14.64 to 15.91)*	15.99 (15.09 to 16.90)*	14.04 (13.44 to 14.63)*
Government Health Expenditure	709.52	4816.26	18016.95	18.95 (16.72 to 21.22)*	24.39 (20.91 to 27.97)*	13.52 (11.30 to 15.79)*
Social Health Expenditure	1171.94	6154.49	29150.57	19.03 (17.56 to 20.52)*	21.13 (18.16 to 24.18)*	17.17 (16.18 to 18.16)*
Out-of-pocket Health Expenditure	2705.17	6571.16	18673.87	10.62 (9.24 to 12.02)*	9.82 (9.21 to 10.44)*	10.96 (8.48 to 13.48)*
Per Capita Health Expenditure (yuan)	361.88	1314.26	4702.79	14.67 (14.04 to 15.30)*	15.34 (14.45 to 16.24)*	13.47 (12.89 to 14.06)*

AAPC: average annual percent change; CI, confidence interval. *AAPC significantly differs from zero at a two-tailed level of 0.05.

For peer review only

For peer review only - http://bmjopen.bmj.com/site/about/guidelines.xhtml

Population health, disease burden and risk factors between 2000 and 2019

According to data from GBD 2019, life expectancy at birth and at 60–64 years were estimated to increase (AAPC: 0.44, 95%CI: 0.43 to 0.46; AAPC: 0.76, 95%CI: 0.60 to 0.91, respectively). In addition, the percentage of healthy life expectancy within total life expectancy at birth and at 60–64 years decreased from 88.90 to 88.32, and from 77.12 to 76.36, respectively (sFigure 1, sTable 1–3). Overall, the number of all-cause DALYs was stable during 2000–2019 (AAPC: 0.08, 95%CI -0.06 to 0.22). However, it decreased between 2000 and 2009 (AAPC: -0.44, 95%CI: -0.58 to -0.31), and it increased between 2010 and 2019 (AAPC: 0.60, 95% CI: 0.39 to 0.82) (sTable 4).

In 2000, the top three causes of DALYs were cardiovascular diseases, neoplasms, and chronic respiratory diseases. In 2019, they were cardiovascular diseases, neoplasms, and musculoskeletal disorders. From 2000–2019, the top three drivers of increasing number of DALYs were HIV/AIDS and sexually transmitted infections (AAPC: 3.47, 95%CI: 2.72 to 4.22), diabetes and kidney diseases (AAPC: 2.30, 95%CI: 2.09 to 2.51), and sense organ diseases (AAPC: 2.14, 95%CI: 1.98 to 2.30). The top two drivers of increasing age-standardized rate of DALYs were HIV/AIDS and sexually transmitted infections (AAPC: 2.02, 95%CI: 1.50 to 2.53) and musculoskeletal disorders (AAPC: 0.16, 95%CI: 0.10 to 0.23) (sTable 5).

In 2000, the top three risk factors were tobacco, air pollution, and high systolic blood pressure. In 2019, they were tobacco, high systolic blood pressure, and dietary risks. Between 2000 and 2019, the top three leading risk factors with the largest increasing number of DALYs were high body-mass index (AAPC: 4.02, 95%CI: 3.74 to 4.29), low physical activity (AAPC: 3.54, 95%CI: 2.70 to 4.38), and low bone mineral density (AAPC:

For peer review only

For peer review only - http://bmjopen.bmj.com/site/about/guidelines.xhtml

3.39, 95%CI: 3.12 to 3.67). The top three leading factors with the largest increase in age-standardized rate of DALYs were unsafe sex (AAPC: 1.47, 95% CI: 0.88 to 2.06), high body-mass index (AAPC: 1.28, 95%CI: 0.95 to 1.60), and low bone mineral density (AAPC: 0.40, 95%CI: 0.27 to 0.54) (sTable 6).

Trends in disease burden between younger individuals and those older than 70 years

Finally, trends in causes and risk factors for the population with a cut-off point of 70 years were examined for 2000–2019. Among individuals younger than 70 years, musculoskeletal disorders replaced other non-communicable diseases as one of the top three causes from 2000 to 2019. In contrast, the top three causes remained the same among individuals older than 70 years, and included cardiovascular diseases, neoplasms, and chronic respiratory disease (Table 3). Among the population younger than 70 years, the causes with the largest increment in number of DALYs were HIV/AIDS and sexually transmitted infections (AAPC: 3.33, 95%CI: 2.58 to 4.08), musculoskeletal disorders (AAPC: 1.88, 95%CI: 1.84 to 1.92), and sense organ diseases (AAPC: 1.71, 95%CI: 1.43 to 2.00). In the same population, the largest increments in rate of DALYs were HIV/AIDS and sexually transmitted infections (AAPC: 3.42, 95%CI: 2.76 to 4.09), musculoskeletal disorders (AAPC: 2.04, 95%CI: 2.00 to 2.08), and diabetes and kidney diseases (AAPC: 1.88, 95%CI: 1.58 to 2.18). In comparison, the causes among individuals older than 70 years that had the largest increment in both number and rate of DALYs were maternal and neonatal disorders (AAPC: 10.64, 95%CI: 10.47 to 10.82; AAPC: 6.84, 95%CI: 6.69 to 6.98, respectively), HIV/AIDS and sexually transmitted infections (AAPC: 8.17, 95%CI: 6.93 to 9.43;AAPC:4.39, 95%CI: 3.26 to 3.53, respectively), and unintentional injuries (AAPC:4.66, 95%CI: 3.90 to 5.43; AAPC: 1.01, 95%CI: 0.46 to 1.56, respectively) (Table

For peer review only

For peer review only - http://bmjopen.bmj.com/site/about/guidelines.xhtml

3).

Among the population younger than 70 years, dietary risks (AAPC: 1.02, 95%CI: 0.85 to 1.19) and high systolic blood pressure (AAPC: 1.55, 95%CI: 1.29 to 1.81) replaced air pollution and child and maternal malnutrition to enter the top three risk factors for 2000–2019. In contrast, the top three risk factors remained the same between 2000 and 2019 for the population older than 70 years, and they include: high systolic blood pressure, tobacco, and air pollution (Table 3). Regarding risk factors among the population younger than 70 years, the largest increments in number and rate of DALYs were high body-mass index (AAPC: 3.70, 95%CI: 3.59 to 3.82; AAPC: 3.88, 95%CI: 3.69 to 4.07, respectively), unsafe sex (AAPC: 3.11, 95%CI: 2.80 to 3.42; AAPC: 3.28, 95%CI: 2.96 to 3.60, respectively), and low physical activity (AAPC: 2.75, 95%CI: 2.34 to 3.15; AAPC: 2.91, 95%CI: 2.46 to 3.36, respectively). In contrast, the risk factors with the largest increment in number of DALYs among those older than 70 years were high body-mass index (AAPC: 5.22, 95%CI: 4.79 to 5.65), low bone mineral density (AAPC: 5.22, 95%CI: 4.98 to 5.46), and high LDL cholesterol (AAPC: 5.02, 95%CI: 4.62 to 5.41). Furthermore, the same risk factors with different rankings were observed in terms of rate of DALYs for the same population: low bone mineral density (AAPC: 1.62, 95%CI: 1.38 to 1.86), high body-mass index (AAPC: 1.50, 95%CI: 1.01 to 2.00), and high LDL cholesterol (AAPC: 1.44, 95%CI: 1.04 to 1.84), respectively (Table 4).

For peer review only - http://bmjopen.bmj.com/site/about/guidelines.xhtml

Table 3. The AAPC of 22 causes in people younger and older than 70 years of age presented in DALYs (95% UI)

Causes	Age < 70 years			Age ≥ 70 years		
	2000	2019	AAPC (95% CI)	2000	2019	AAPC (95% CI)
Number						
Neglected tropical diseases and malaria	1,566,614 (1,022,581 to 2,343,199)	977,312 (586,629 to 1,582,219)	-2.42 (-2.73 to -2.10)*	137,451 (93,607 to 195,272)	160,494 (103,985 to 236,535)	0.83 (0.72 to 0.94)*
Nutritional deficiencies	3,912,001 (2,872,239 to 5,269,234)	2,247,485 (1,515,113 to 3,202,132)	-2.91 (-3.07 to -2.76)*	271,587 (210,115 to 352,577)	359,440 (285,475 to 450,281)	1.51 (1.21 to 1.81)*
Neoplasms	43,633,030 (40,123,940 to 47,258,486)	48,503,754 (40,915,702 to 56,689,779)	0.54 (0.36 to 0.73)*	10,782,364 (10,021,201 to 11,549,415)	19,016,076 (16,369,289 to 21,712,270)	3.00 (2.66 to 3.34)*
Cardiovascular diseases	40,746,813 (37,734,622 to 44,374,198)	45,823,157 (39,092,553 to 53,248,142)	0.67 (0.52 to 0.82)*	26,837,679 (24,915,174 to 29,373,891)	46,109,965 (40,669,589 to 51,698,469)	2.77 (2.26 to 3.29)*
Chronic respiratory diseases	13,237,789 (11,103,026 to 14,475,351)	8,969,697 (7,748,010 to 10,534,163)	-2.04 (-2.22 to -1.86)*	15,416,177 (12,665,862 to 16,567,447)	13,550,852 (11,816,464 to 16,036,186)	-0.70 (-1.15 to -0.24)*
Digestive diseases	9,775,769 (8,808,397 to 10,835,090)	7,710,658 (6,445,828 to 8,964,870)	-1.21 (-1.30 to -1.13)*	1,923,302 (1,762,799 to 2,106,969)	2,307,223 (2,029,697 to 2,600,797)	0.92 (0.72 to 1.13)*
Neurological disorders	9,677,839 (4,755,522 to 16,786,166)	11,207,269 (5,201,198 to 19,881,074)	0.78 (0.70 to 0.86)*	2,828,463 (1,694,636 to 5,326,904)	6,174,136 (3,629,888 to 11,661,806)	4.20 (4.04 to 4.35)*
Mental disorders	17,241,666 (12,746,572 to 22,736,338)	18,539,346 (13,738,299 to 24,152,654)	0.37 (0.29 to 0.46)*	868,363 (645,857 to 1,119,766)	1,754,481 (1,291,689 to 2,254,704)	3.77 (3.70 to 3.83)*
Musculoskeletal disorders	17,725,280 (12,654,675 to 23,783,378)	25,286,535 (18,045,649 to 33,942,087)	1.88 (1.84 to 1.92)*	2,364,272 (1,679,407 to 3,221,901)	4,647,389 (3,292,799 to 6,332,328)	3.60 (3.53 to 3.66)*
Other non-communicable diseases	23,430,651 (19,446,807 to 28,355,357)	16,912,189 (12,820,141 to 22,584,969)	-1.67 (-1.77 to -1.58)*	1,142,340 (831,796 to 1,540,224)	2,213,884 (1,599,002 to 2,992,458)	3.43 (2.88 to 3.98)*
Skin and subcutaneous diseases	7,427,502 (4,833,763 to 10,987,503)	7,564,844 (4,920,805 to 11,324,882)	0.09 (0.07 to 0.12)*	381,523 (263,385 to 542,001)	699,858 (475,558 to 1,030,747)	3.21 (3.14 to 3.28)*
Sense organ diseases	7,881,599 (5,268,499 to 11,424,474)	10,796,982 (7,099,138 to 15,924,830)	1.71 (1.43 to 2.00)*	2,859,183 (2,030,582 to 3,892,635)	5,184,684 (3,650,080 to 7,090,135)	3.18 (3.02 to 3.34)*
Transport injuries	16,117,112 (14,766,378 to 18,356,266)	12,597,412 (10,747,152 to 14,456,584)	-1.31 (-1.64 to -0.98)*	514,733 (454,228 to 601,574)	1,148,043 (946,296 to 1,360,515)	4.24 (3.92 to 4.57)*
Unintentional injuries	19,929,305 (18,307,020 to 21,760,124)	13,173,922 (10,853,663 to 15,202,200)	-2.15 (-2.65 to -1.65)*	1,184,747 (1,024,676 to 1,371,585)	2,829,540 (2,151,923 to 3,469,812)	4.66 (3.90 to 5.43)*
Self-harm and interpersonal violence	10,574,276 (9,275,669 to 11,544,266)	5,232,715 (4,479,867 to 6,200,044)	-3.64 (-3.94 to -3.34)*	597,000 (504,957 to 649,633)	637,335 (544,995 to 738,168)	0.37 (-0.73 to 1.50)
HIV/AIDS and sexually transmitted infections	904,578 (711,182 to 1,201,186)	1,677,944 (1,372,709 to 2,020,540)	3.33 (2.58 to 4.08)*	16,867 (13,900 to 22,250)	74,476 (55,158 to 94,754)	8.17 (6.93 to 9.43)*
Respiratory infections and tuberculosis	19,691,768 (18,048,674 to 21,296,455)	4,601,349 (3,952,381 to 5,376,466)	-7.32 (-7.79 to -6.85)*	2,246,454 (2,045,162 to 2,412,569)	2,046,523 (1,783,057 to 2,379,652)	-0.54 (-0.96 to -0.13)*
Enteric infections	3,259,938 (2,859,853 to 3,751,832)	1,179,711 (874,430 to 1,534,794)	-5.11 (-5.55 to -4.66)*	147,328 (108,525 to 198,192)	228,387 (164,251 to 306,599)	2.47 (2.25 to 2.69)*
Other infectious diseases	4,467,414 (3,610,475 to 6,110,768)	1,143,025 (971,599 to 1,357,011)	-6.85 (-7.29 to -6.42)*	114,576 (103,794 to 126,392)	101,074 (86,237 to 116,157)	-0.59 (-0.98 to -0.21)*
Maternal and neonatal disorders	18,100,148 (16,534,223 to 19,650,436)	7,080,589 (6,220,729 to 8,094,637)	-4.86 (-5.45 to -4.27)*	10,442 (7,450 to 14,363)	70,983 (51,665 to 96,401)	10.64 (10.47 to 10.82)*
Substance use disorders	6,144,467 (4,909,131 to 7,567,428)	5,459,715 (4,162,774 to 6,975,972)	-0.55 (-0.97 to -0.13)*	147,447 (122,722 to 177,177)	295,943 (226,537 to 375,653)	3.80 (3.23 to 4.37)*
Diabetes and kidney diseases	8,018,292 (6,937,163 to 9,207,068)	10,979,432 (9,048,531 to 13,121,983)	1.70 (1.42 to 1.98)*	2,418,531 (2,116,442 to 2,752,813)	4,929,741 (4,274,671 to 5,652,658)	3.78 (3.60 to 3.96)*
Rate						
Neglected tropical diseases and malaria	33 (21 to 49)	21 (13 to 34)	-2.26 (-2.56 to -1.96)*	250 (170 to 355)	149 (96 to 219)	-2.70 (-2.80 to -2.59)*
Nutritional deficiencies	82 (60 to 110)	48 (33 to 69)	-2.77 (-2.96 to -2.58)*	494 (383 to 642)	333 (264 to 417)	-2.03 (-2.30 to -1.76)*
Neoplasms	911 (838 to 987)	1,045 (881 to 1,221)	0.68 (0.59 to 0.77)*	19,629 (18,243 to 21,025)	17,613 (15,162 to 20,110)	-0.56 (-0.93 to -0.19)*
Cardiovascular diseases	851 (788 to 927)	987 (842 to 1,147)	0.84 (0.68 to 1.00)*	48,857 (45,357 to 53,474)	42,708 (37,669 to 47,885)	-0.69 (-0.82 to -0.57)*
Chronic respiratory diseases	276 (232 to 302)	193 (167 to 227)	-1.86 (-2.02 to -1.69)*	28,064 (23,058 to 30,160)	12,551 (10,945 to 14,853)	-4.13 (-4.60 to -3.66)*

For peer review only - http://bmjopen.bmj.com/site/about/guidelines.xhtml

Digestive diseases	204 (184 to 226)	166 (139 to 193)	-1.06 (-1.15 to -0.96)*	3,501 (3,209 to 3,836)	2,137 (1,880 to 2,409)	-2.55 (-2.66 to -2.45)*
Neurological disorders	202 (99 to 351)	241 (112 to 428)	0.94 (0.82 to 1.06)*	5,149 (3,085 to 9,697)	5,719 (3,362 to 10,801)	0.59 (0.29 to 0.90)*
Mental disorders	360 (266 to 475)	399 (296 to 520)	0.55 (0.51 to 0.59)*	1,581 (1,176 to 2,038)	1,625 (1,196 to 2,088)	0.15 (0.12 to 0.18)*
Musculoskeletal disorders	370 (264 to 497)	545 (389 to 731)	2.04 (2.00 to 2.08)*	4,304 (3,057 to 5,865)	4,305 (3,050 to 5,865)	-0.04 (-0.15 to 0.08)
Other non-communicable diseases	489 (406 to 592)	364 (276 to 486)	-1.54 (-1.66 to -1.42)*	2,080 (1,514 to 2,804)	2,051 (1,481 to 2,772)	-0.16 (-0.60 to 0.29)
Skin and subcutaneous diseases	155 (101 to 229)	163 (106 to 244)	0.26 (0.23 to 0.29)*	695 (479 to 987)	648 (440 to 955)	-0.38 (-0.39 to -0.36)*
Sense organ diseases	165 (110 to 239)	233 (153 to 343)	1.86 (1.70 to 2.02)*	5,205 (3,697 to 7,086)	4,802 (3,381 to 6,567)	-0.43 (-0.63 to -0.23)*
Transport injuries	337 (308 to 383)	271 (231 to 311)	-1.15 (-1.49 to -0.81)*	937 (827 to 1,095)	1,063 (876 to 1,260)	0.64 (0.33 to 0.96)*
Unintentional injuries	416 (382 to 454)	284 (234 to 327)	-1.93 (-2.44 to -1.42)*	2,157 (1,865 to 2,497)	2,621 (1,993 to 3,214)	1.01 (0.46 to 1.56)*
Self-harm and interpersonal violence	221 (194 to 241)	113 (96 to 134)	-3.49 (-3.80 to -3.18)*	1,087 (919 to 1,183)	590 (505 to 684)	-3.10 (-4.05 to -2.14)*
HIV/AIDS and sexually transmitted infections	19 (15 to 25)	36 (30 to 44)	3.42 (2.76 to 4.09)*	31 (25 to 41)	69 (51 to 88)	4.39 (3.26 to 3.53)*
Respiratory infections and tuberculosis	411 (377 to 445)	99 (85 to 116)	-7.17 (-7.61 to -6.71)*	4,090 (3,723 to 4,392)	1,896 (1,652 to 2,204)	-4.08 (-4.44 to -3.71)*
Enteric infections	68 (60 to 78)	25 (19 to 33)	-4.91 (-5.76 to -4.05)*	268 (198 to 361)	212 (152 to 284)	-1.20 (-1.45 to -0.96)*
Other infectious diseases	93 (75 to 128)	25 (21 to 29)	-6.70 (-7.14 to -6.27)*	209 (189 to 230)	94 (80 to 108)	-4.06 (-4.48 to -3.64)*
Maternal and neonatal disorders	378 (345 to 410)	153 (134 to 174)	-4.71 (-5.29 to -4.14)*	19 (14 to 26)	66 (48 to 89)	6.84 (6.69 to 6.98)*
Substance use disorders	128 (103 to 158)	118 (90 to 150)	-0.38 (-0.85 to 0.09)*	268 (223 to 323)	274 (210 to 348)	0.12 (-0.31 to 0.55)
Diabetes and kidney diseases	167 (145 to 192)	236 (195 to 283)	1.88 (1.58 to 2.18)*	4,403 (3,853 to 5,011)	4,566 (3,959 to 5,236)	0.21 (-0.05 to 0.48)

AAPC: average annual percent change; DALYs, disability adjusted life years; UI, uncertainty interval, CI, confidence interval.
*AAPC significantly differs from zero at a two-tailed level of 0.05.

For peer review only

For peer review only - http://bmjopen.bmj.com/site/about/guidelines.xhtml

Table 4. The AAPC of twenty risk factors in people younger and older than 70 years of age

Risk factors	Age < 70 years			Age ≥ 70 years		
	2000	2019	AAPC (95% CI)	2000	2019	AAPC (95% CI)
**Number**						
Unsafe water, sanitation, and handwashing	3,996,889 (3,183,257 to 4,887,734)	923,837 (645,583 to 1,254,723)	-7.42 (-8.15 to -6.69)*	237,825 (168,202 to 320,654)	228,667 (159,219 to 307,023)	-0.14 (-0.42 to 0.13)
Air pollution	32,546,257 (28,559,720 to 36,241,262)	24,301,291 (20,582,581 to 28,569,480)	-1.52 (-1.70 to -1.34)*	16,789,702 (14,415,333 to 19,203,189)	18,208,412 (15,565,447 to 21,260,363)	0.27 (0.00 to 0.54)*
Other environmental risks	3,596,355 (2,485,008 to 4,876,076)	3,222,783 (1,977,748 to 4,651,395)	-0.53 (-0.77 to -0.29)*	1,790,347 (1,199,126 to 2,480,222)	3,083,288 (2,034,326 to 4,259,059)	2.86 (2.48 to 3.23)*
Child and maternal malnutrition	27,473,911 (24,705,249 to 30,472,454)	6,937,292 (6,012,640 to 7,958,846)	-6.98 (-7.79 to -6.16)*	256,375 (197,956 to 332,924)	316,491 (248,797 to 398,362)	1.15 (0.66 to 1.63)*
Tobacco	37,227,472 (33,809,017 to 40,999,710)	38,743,936 (32,232,707 to 46,296,710)	0.22 (0.08 to 0.37)*	20,037,114 (18,407,452 to 21,694,478)	25,372,064 (22,095,893 to 28,843,357)	1.24 (0.79 to 1.69)*
Alcohol use	12,593,606 (10,822,456 to 14,698,783)	13,876,946 (11,135,736 to 16,941,170)	0.47 (0.39 to 0.56)*	2,079,979 (1,635,218 to 2,555,460)	3,388,114 (2,514,663 to 4,384,540)	2.51 (2.10 to 2.92)*
Drug use	5,880,735 (5,044,491 to 6,863,455)	4,596,666 (3,725,262 to 5,556,055)	-1.24 (-1.46 to -1.02)*	420,543 (338,823 to 505,234)	525,128 (420,389 to 638,546)	1.10 (0.84 to 1.35)*
High fasting plasma glucose	10,850,938 (8,940,115 to 13,134,425)	16,082,215 (12,638,442 to 19,989,086)	2.09 (1.69 to 2.50)*	7,073,207 (5,239,551 to 9,435,756)	12,146,224 (9,068,765 to 16,392,373)	2.86 (2.43 to 3.29)*
High systolic blood pressure	21,853,891 (18,103,552 to 25,435,298)	28,919,385 (23,316,926 to 34,533,463)	1.55 (1.29 to 1.81)*	13,487,603 (10,597,533 to 16,167,287)	25,522,231 (20,992,213 to 30,524,269)	3.37 (2.90 to 3.84)*
High body-mass index	9,311,731 (3,191,606 to 17,435,563)	18,520,264 (9,033,132 to 29,514,190)	3.70 (3.59 to 3.82)*	2,392,758 (706,129 to 5,015,786)	6,309,777 (2,532,862 to 11,224,097)	5.22 (4.79 to 5.65)*
Low bone mineral density	1,234,815 (1,026,536 to 1,443,017)	1,913,892 (1,512,289 to 2,304,790)	2.37 (2.10 to 2.64)*	534,562 (448,070 to 635,290)	1,406,383 (1,058,504 to 1,740,813)	5.22 (4.98 to 5.46)*
Dietary risks	24,107,231 (19,699,737 to 29,721,184)	29,061,110 (22,436,447 to 36,581,781)	1.02 (0.85 to 1.19)*	10,146,090 (7,449,653 to 13,767,645)	17,752,016 (12,683,501 to 23,791,732)	2.90 (2.59 to 3.21)*
Low physical activity	479,307 (211,814 to 1,031,049)	793,859 (332,155 to 1,622,549)	2.75 (2.34 to 3.15)*	795,916 (391,872 to 1,491,328)	1,713,262 (787,433 to 3,275,090)	3.97 (3.34 to 4.60)*
Occupational risks	14,894,928 (12,651,846 to 17,338,599)	11,828,821 (9,596,301 to 14,324,948)	-1.20 (-1.31 to -1.10)*	3,676,403 (2,901,359 to 4,413,363)	3,658,028 (2,941,788 to 4,424,853)	-0.10 (-0.45 to 0.25)
Intimate partner violence	766,126 (417,803 to 1,191,498)	739,392 (335,422 to 1,209,468)	-0.12 (-0.36 to 0.12)*	23,715 (10,039 to 44,392)	50,534 (16,543 to 99,799)	4.12 (3.96 to 4.29)*
Unsafe sex	1,352,963 (1,174,728 to 1,769,992)	2,445,381 (1,761,748 to 2,945,569)	3.11 (2.80 to 3.42)*	129,732 (113,048 to 182,721)	279,416 (197,910 to 338,222)	4.29 (3.79 to 4.79)*
Non-optimal temperature	3,641,568 (2,714,325 to 4,594,010)	3,468,758 (2,670,613 to 4,405,174)	-0.24 (-1.11 to 0.64)	4,785,750 (4,234,955 to 5,404,845)	5,856,605 (4,952,561 to 6,867,429)	0.76 (0.49 to 1.02)*
Kidney dysfunction	6,244,259 (5,571,754 to 7,041,339)	7,536,042 (6,221,117 to 9,011,471)	1.08 (0.82 to 1.35)*	2,639,944 (2,227,097 to 3,100,279)	5,817,866 (4,717,831 to 7,022,861)	4.30 (3.63 to 4.97)*
High LDL cholesterol	8,217,261 (6,919,451 to 9,686,198)	12,184,167 (9,541,238 to 14,971,019)	2.16 (1.91 to 2.42)*	3,000,527 (1,868,209 to 4,589,459)	7,629,795 (4,751,976 to 11,248,818)	5.02 (4.62 to 5.41)*
Childhood sexual abuse and bullying	755,840 (307,189 to 1,434,919)	629,932 (269,309 to 1,156,818)	-0.95 (-1.06 to -0.84)*	6,905 (2,954 to 12,651)	16,924 (7,261 to 32,044)	4.86 (4.72 to 5.00)*
**Rate**						
Unsafe water, sanitation, and handwashing	83 (66 to 102)	20 (14 to 27)	-7.24 (-7.97 to -6.51)*	433 (306 to 584)	212 (147 to 284)	-3.66 (-3.92 to -3.40)*
Air pollution	680 (596 to 757)	523 (443 to 615)	-1.36 (-1.53 to -1.19)*	30,565 (26,242 to 34,958)	16,865 (14,417 to 19,692)	-3.04 (-3.35 to -2.74)*
Other environmental risks	75 (52 to 102)	69 (43 to 100)	-0.37 (-0.59 to -0.14)*	3,259 (2,183 to 4,515)	2,856 (1,884 to 3,945)	-0.64 (-0.95 to -0.32)*
Child and maternal malnutrition	574 (516 to 636)	149 (130 to 171)	-6.83 (-7.48 to -6.18)*	467 (360 to 606)	293 (230 to 369)	-2.43 (-2.80 to -2.06)*
Tobacco	777 (706 to 856)	835 (694 to 997)	0.38 (0.23 to 0.53)*	36,476 (33,510 to 39,494)	23,500 (20,466 to 26,716)	-2.29 (-2.71 to -1.87)*
Alcohol use	263 (226 to 307)	299 (240 to 365)	0.61 (0.33 to 0.90)*	3,786 (2,977 to 4,652)	3,138 (2,329 to 4,061)	-1.09 (-1.57 to -0.61)*
Drug use	123 (105 to 143)	99 (80 to 120)	-1.02 (-1.22 to -0.82)*	766 (617 to 920)	486 (389 to 591)	-2.34 (-2.54 to -2.15)*

For peer review only - http://bmjopen.bmj.com/site/about/guidelines.xhtml

High fasting plasma glucose	227 (187 to 274)	346 (272 to 431)	2.25 (1.82 to 2.69)*	12,876 (9,538 to 17,177)	11,250 (8,400 to 15,183)	-0.79 (-1.48 to -0.10)*
High systolic blood pressure	456 (378 to 531)	623 (502 to 744)	1.72 (1.47 to 1.98)*	24,553 (19,292 to 29,432)	23,639 (19,444 to 28,272)	-0.15 (-0.42 to 0.13)
High body-mass index	194 (67 to 364)	399 (195 to 636)	3.88 (3.69 to 4.07)*	4,356 (1,285 to 9,131)	5,844 (2,346 to 10,396)	1.50 (1.01 to 2.00)*
Low bone mineral density	26 (21 to 30)	41 (33 to 50)	2.54 (2.20 to 2.89)*	973 (816 to 1,157)	1,303 (980 to 1,612)	1.62 (1.38 to 1.86)*
Dietary risks	503 (411 to 621)	626 (483 to 788)	1.19 (1.02 to 1.36)*	18,470 (13,562 to 25,063)	16,442 (11,748 to 22,037)	-0.58 (-0.74 to -0.43)*
Low physical activity	10 (4 to 22)	17 (7 to 35)	2.91 (2.46 to 3.36)*	1,449 (713 to 2,715)	1,587 (729 to 3,033)	0.40 (-0.21 to 1.02)*
Occupational risks	311 (264 to 362)	255 (207 to 309)	-1.05 (-1.10 to -0.99)*	6,693 (5,282 to 8,034)	3,388 (2,725 to 4,098)	-3.51 (-3.97 to -3.05)*
Intimate partner violence	16 (9 to 25)	16 (7 to 26)	0.02 (-0.23 to 0.27)*	43 (18 to 81)	47 (15 to 92)	0.49 (0.31 to 0.66)*
Unsafe sex	28 (25 to 37)	53 (38 to 63)	3.28 (2.96 to 3.60)*	236 (206 to 333)	259 (183 to 313)	0.69 (0.16 to 1.22)*
Non-optimal temperature	76 (57 to 96)	75 (58 to 95)	-0.05 (-0.89 to 0.81)*	8,712 (7,710 to 9,839)	5,425 (4,587 to 6,361)	-2.59 (-3.60 to -1.56)*
Kidney dysfunction	130 (116 to 147)	162 (134 to 194)	1.25 (1.00 to 1.49)*	4,806 (4,054 to 5,644)	5,389 (4,370 to 6,505)	0.64 (-0.02 to 1.31)
High LDL cholesterol	172 (144 to 202)	262 (206 to 322)	2.33 (2.07 to 2.59)*	5,462 (3,401 to 8,355)	7,067 (4,401 to 10,419)	1.44 (1.04 to 1.84)*
Childhood sexual abuse and bullying	16 (6 to 30)	14 (6 to 25)	-0.80 (-0.89 to -0.72)*	13 (5 to 23)	16 (7 to 30)	1.21 (1.06 to 1.37)*

AAPC: average annual percent change; DALYs, disability adjusted life years; UI, uncertainty interval, CI, confidence interval.
*AAPC significantly differs from zero at a two-tailed level of 0.05.

For peer review only

For peer review only - http://bmjopen.bmj.com/site/about/guidelines.xhtml

Discussion

In the past two decades, although China invested hugely on heath institution, personnel, and expenditures to extend life expectancy and improve healthy life expectancy, population structure deteriorated, the total disease burden remained largely unchanged, ageing driven disease burden continuously intensified. The trends we observed in the population structure of China highlights the potential for an irreversible population decline to occur before the household wealth of G7 nations is achieved in China. Despite easing of birth limits in China, couples have been put off by high costs of living, expensive childcare, career choices, and maternity leave. In addition, the post-working population has been increasing at an accelerating rate, suggesting a clear deterioration in population structure and ageing[19]. Thus, the ability of China to ease strains on its ageing population in the next 30 years is a pressing concern[21].

On the one hand, continuous effort is needed to address the top three causes and risk factors which have been identified among the population in China between 2000 and 2019. Our findings show that neoplasms and cardiovascular diseases remain among the top three causes, and tobacco remains among the top three risk factors for individuals younger than 70 years. Comprehensive regional-specific strategies, including effective tobacco-control policy, recommendations for healthier lifestyles, and control of chronic infections, are needed as the cancer spectrum in China is changing from that of a developing country to that of a developed country[22, 23]. Regulatory reforms also need to further reduce the lag between approval of new cancer drugs by the US FDA and their subsequent approval in China, and they need to facilitate the development and registration of drugs that may have clinical superiority over existing drugs[24]. Turning the inverted pyramid of the health care

For peer review only - http://bmjopen.bmj.com/site/about/guidelines.xhtml

system is essential for the battle against cardiovascular disease due to the increasing number of older patients, the high proportion of out-of-hospital deaths, and the gaps in lifestyle indicators between what is preferred and what is currently observed[25, 26]. All provinces in Chinese mainland have made great progress in increasing life expectancy at birth. Life course management of disease across the entire spectrum of health care services has led to a shift from a patient-centered and treatment-dominated model to a people-centered and health-centered model. It is hypothesized that the latter may help to integrate preventive and treatment measures for cardiovascular disease[27]. Tobacco sales have also increased despite a tight anti-smoking policy in China. Potential interference from the tobacco industry and the social currency of tobacco are suspected to be factors[28-30]. Therefore, great efforts should be taken not only to minimize these practices, but also to slow tobacco production[31].

On the other hand, it is crucial to focus on incremental driving causes and risk factors in the early stages to mitigate future ageing-driven disease burden. Our data show that musculoskeletal disorders, dietary risks, and high systolic blood pressure are among the top three causes and risk factors in people younger than 70 years. Split analyses were performed to examine variations according to age, and a cut-off point of 70 years was used because ischemic heart disease and stroke were the top-ranked causes of DALYs in the population aged 70 years and older[19]. A previous study quantified the temporal trends in disease burden of musculoskeletal disorders which could be attributed to changes in risk factors [27]. The results obtained support population-wide initiatives which target high body-mass index to help mitigate the burden of musculoskeletal disorders[32]. Changes in diets to correct for urbanization-driven dietary shifts to higher consumption of refined

For peer review only

For peer review only - http://bmjopen.bmj.com/site/about/guidelines.xhtml

sugars, refined fats, oils, and meats may also help prevent diet-related and chronic non-communicable diseases[33]. With resources available to control high systolic blood pressure and reduce the burden of hypertension, efforts are needed to improve access to high value antihypertensive medications and to implement artificial intelligence-based digital health[34, 35].

We identified key domains that require intervention in order to reduce an increasing disease burden among individuals older than 70 years. Most importantly, the top three causes and risk factors for this age group remained unchanged. Thus, our findings indicate that greater effort is needed to tackle the rapidly increasing age-standardized rate of DALYs due to traditional major risk factors, including high body-mass index, low bone mineral density, and high LDL cholesterol. Moreover, the increasing burden of maternal and neonatal disorders, HIV/AIDS and sexually transmitted infections, and unintentional injuries needs to be highlighted as well.

Health care for older individuals is undergoing unprecedented challenges due to underprepared geriatric hospitals and nursing homes, an unbalanced health care insurance system between urban and rural areas, and an absence of education on national aging and post-working health[21]. Consequently, there is an urgent need to identify traits in older people who experience short ill-spans. By identifying healthy behaviors and casual genetic and metabolic factors of the latter, it may be possible to decrease the burden of late-life diseases and extend healthspan[36]. The development of long-term health systems to meet the needs of older individuals in an age-friendly environment is fundamental to fostering healthy ageing and maximizing the functional abilities of older individuals[37].

Health care has experienced an upsurge in regard to medical institutions, personnel,

For peer review only

For peer review only - http://bmjopen.bmj.com/site/about/guidelines.xhtml

347 beds, and expenditures. Our findings clearly demonstrate that China has increased its health
348 spending much faster than its economic growth. In 2018, China provided $644.7 million
349 for the development of health assistance[38]. This trend in increasing health care
350 expenditures will be further aggravated considering the high costs related to chronic
351 illnesses, population growth, and ageing in the United States[9, 39]. While China has
352 reformed its primary health care system to insure its population[7, 40], improving the
353 quality of primary health care and increasing reimbursement for chronic non-
354 communicable diseases are key steps to alleviating national disease burden[41, 42]. In
355 addition, the cost-effectiveness of heath expenditures on disease burden and targeting the
356 poor should be primary considerations in public health policy given the limited financial
357 resources for heath[43].

358 Two set of age group was used to specifically assess the trend of population
359 composition and disease burden. Population composition (0-14, 15-64, 65+) and
360 dependency ratio (gross, children, old) were used to reflect the trend of working population
361 and population ageing as age 0-14, 15-64 and 65+ group were younger population, working
362 population, post-working population, respectively. The analysis of disease burden in
363 population younger and older than 70 years aimed to identify the cause or risk factor (driver)
364 which has the greatest impact on the trend of disease burden attributable to 22 causes and
365 20 risk factors from 2000 to 2019. The outpoint of age 70 was chosen because women in
366 164 and men in 165 of 186 countries and territories had a higher probability of dying before
367 70 years of age from a non-communicable disease (NCD) than from communicable,
368 maternal, perinatal, and nutritional conditions combined according to NCD Countdown
369 2030[19, 44].

For peer review only - http://bmjopen.bmj.com/site/about/guidelines.xhtml

Limitation of this study. It must also be noted that there were several limitations associated with the present study. First, our findings might be biased by changes in the age structure of the population and different population over a specified period because temporal trends in population and health care utilization were not quantified in terms of age standardized rate. Therefore, the trend of disease burden at each time was not evaluated based on quantification. Second, population and health care data from CSY 2020 were combined with modeled disease burden data from GBD 2019 based on an assumption that these two sources of data are consistent. Both data come from different databases, so there are some limitations on the burden of diseases in Chinese population. Third, the most recent trends could not be captured due to time lags in data release by the NBS of China and GBD studies. Fourth, all the limitations of the GBD methodology described elsewhere [3, 19, 20] could have potentially affected the present study. Fifth, owing to the nature of the data and methods from CSY, we are currently unable to parse population estimates into more narrow age groups and provide deflator-adjusted health expenditure data. National-level data were collected and analyzed, and therefore, within-country variations were not considered.

Conclusion

Comprehensive age-cause-risk factor-specific strategies are key to reconcile the tension among triangle of population ageing, disease burden and healthcare expenditure. The disease burden from cardiometabolic diseases, neoplasms and musculoskeletal disorders were identified as key domains that require intervention to reduce an increasing disease burden among individuals currently older than 70 years, as well as those approaching this age group.

For peer review only - http://bmjopen.bmj.com/site/about/guidelines.xhtml

For peer review only

For peer review only - http://bmjopen.bmj.com/site/about/guidelines.xhtml

Contributors

Prof. Jieruo Gu had full access to all of the data in the study and takes responsibility for the integrity of the data and the accuracy of the data analysis. Dr. Dongze Wu, Dr.Zhenguo Liang, Dr. Cui Guo, and Prof. Jieruo Gu conceived and designed the study, performed the analysis, wrote the paper. All authors reviewed and revised the paper.

Conflict of Interest

The authors confirm that there are no conflicts of interest.

Funding Sources

Project funded by China Postdoctoral Science Foundation (2020TQ0380) and Guangdong Clinical Research Center of Immune disease (2020B1111170008). The Global Burden of Disease Study is funded by the Bill and Melinda Gates Foundation. The funder was not involved in the preparation of this manuscript.

Data sharing statement

Data used for the analyses are publicly available from the Institute of Health Metrics and Evaluation (www.ihme.org) and the National Bureau of Statistics of China (https://data.stats.gov.cn/english/index.htm; http://www.stats.gov.cn/tjsj/ndsj/2020/indexeh.htm).

Ethical approval

Data released from the Global Health Data Exchange query and the China Statistical Yearbook did not require informed patient consent. This study used an anonymized publicly available data set with no identifiable information of the survey participants. Thus, ethics approval was not required for this study.

For peer review only

For peer review only - http://bmjopen.bmj.com/site/about/guidelines.xhtml

References

1. Lopez-Otin, C., et al., *The hallmarks of aging.* Cell, 2013. **153**(6): p. 1194-217.
2. Lopez-Otin, C. and G. Kroemer, *Hallmarks of Health.* Cell, 2021. **184**(1): p. 33-63.
[dataset]3. Collaborators, G.B.D.D., *Global age-sex-specific fertility, mortality, healthy life expectancy (HALE), and population estimates in 204 countries and territories, 1950-2019: a comprehensive demographic analysis for the Global Burden of Disease Study 2019.* Lancet, 2020. **396**(10258): p. 1160-1203.
4. Vollset, S.E., et al., *Fertility, mortality, migration, and population scenarios for 195 countries and territories from 2017 to 2100: a forecasting analysis for the Global Burden of Disease Study.* Lancet, 2020. **396**(10258): p. 1285-1306.
5. Zeng, Y. and T. Hesketh, *The effects of China's universal two-child policy.* Lancet, 2016. **388**(10054): p. 1930-1938.
6. Tatum, M., *China's three-child policy.* Lancet, 2021. **397**(10291): p. 2238.
7. Li, X., et al., *The primary health-care system in China.* Lancet, 2017. **390**(10112): p. 2584-2594.
8. Yip, W., et al., *10 years of health-care reform in China: progress and gaps in Universal Health Coverage.* Lancet, 2019. **394**(10204): p. 1192-1204.
9. Moses, H., 3rd, et al., *The anatomy of health care in the United States.* JAMA, 2013. **310**(18): p. 1947-63.
10. Zhong, N.S., et al., *Epidemiology and cause of severe acute respiratory syndrome (SARS) in Guangdong, People's Republic of China, in February, 2003.* Lancet, 2003. **362**(9393): p. 1353-8.
11. Zhu, N., et al., *A Novel Coronavirus from Patients with Pneumonia in China, 2019.* N Engl J Med, 2020. **382**(8): p. 727-733.
12. Gong, P., et al., *Urbanisation and health in China.* Lancet, 2012. **379**(9818): p. 843-52.
13. Zhou, M., et al., *Mortality, morbidity, and risk factors in China and its provinces, 1990-2017: a systematic analysis for the Global Burden of Disease Study 2017.* Lancet, 2019. **394**(10204): p. 1145-1158.
14. Zhou, M., et al., *Cause-specific mortality for 240 causes in China during 1990-2013: a systematic subnational analysis for the Global Burden of Disease Study 2013.* Lancet, 2016. **387**(10015): p. 251-72.
15. Fang, E.F., et al., *A research agenda for ageing in China in the 21st century (2nd edition): Focusing on basic and translational research, long-term care, policy and social networks.* Ageing Res Rev, 2020. **64**: p. 101174.
16. Li, J., et al., *Spatiotemporal matching between medical resources and population ageing in China from 2008 to 2017.* BMC Public Health, 2020. **20**(1): p. 845.
17. Hu, D., et al., *Age-Related Disease Burden in China, 1997-2017: Findings From the Global Burden of Disease Study.* Front Public Health, 2021. **9**: p. 638704.
[dataset]18. Ning, J.J., et al. *China Statistical Yearbook 2020*. Available from: http://www.stats.gov.cn/tjsj/ndsj/2020/indexeh.htm.
[dataset]19. *Diseases, G.B.D. and C. Injuries, Global burden of 369 diseases and injuries in 204 countries and territories, 1990-2019: a systematic analysis for the Global Burden of Disease Study 2019.* . Lancet, 2020. **396**(10258): p. 1204-1222.

For peer review only - http://bmjopen.bmj.com/site/about/guidelines.xhtml

[dataset]20. Collaborators, G.B.D.R.F., *Global burden of 87 risk factors in 204 countries and territories, 1990-2019: a systematic analysis for the Global Burden of Disease Study 2019.* Lancet, 2020. **396**(10258): p. 1223-1249.

21. Wang, X.Q. and P.J. Chen, *Population ageing challenges health care in China.* Lancet, 2014. **383**(9920): p. 870.

22. Feng, R.M., et al., *Current cancer situation in China: good or bad news from the 2018 Global Cancer Statistics?* Cancer Commun (Lond), 2019. **39**(1): p. 22.

23. Chen, W., et al., *Disparities by province, age, and sex in site-specific cancer burden attributable to 23 potentially modifiable risk factors in China: a comparative risk assessment.* Lancet Glob Health, 2019. **7**(2): p. e257-e269.

24. Li, G., et al., *Characteristics of expedited programmes for cancer drug approval in China.* Nat Rev Drug Discov, 2021. **20**(6): p. 416.

25. Zhao, D., et al., *Epidemiology of cardiovascular disease in China: current features and implications.* Nat Rev Cardiol, 2019. **16**(4): p. 203-212.

26. Du, X., et al., *Epidemiology of Cardiovascular Disease in China and Opportunities for Improvement: JACC International.* J Am Coll Cardiol, 2019. **73**(24): p. 3135-3147.

27. Liu, S., Y. Li, and M. Zhou, *Addressing the Cardiovascular Disease Burden in China-Is It Possible Without Surgery?-Reply.* JAMA Cardiol, 2019. **4**(9): p. 953.

28. Luo, H. and Y. Zhao, *Increasing tobacco sales under the anti-smoking policy in China.* Lancet, 2020. **395**(10240): p. 1834.

29. Yang, G., et al., *The road to effective tobacco control in China.* Lancet, 2015. **385**(9972): p. 1019-28.

30. Rich, Z.C. and S. Xiao, *Tobacco as a social currency: cigarette gifting and sharing in China.* Nicotine Tob Res, 2012. **14**(3): p. 258-63.

31. Dan, X., S. Yuankai, and W. Chen, *Tobacco in China.* Lancet, 2014. **383**(9934): p. 2045-2046.

32. Wu, D., et al., *Pattern and trend of five major musculoskeletal disorders in China from 1990 to 2017: findings from the Global Burden of Disease Study 2017.* BMC Med, 2021. **19**(1): p. 34.

33. Tilman, D. and M. Clark, *Global diets link environmental sustainability and human health.* Nature, 2014. **515**(7528): p. 518-22.

34. Su, M., et al., *Availability, cost, and prescription patterns of antihypertensive medications in primary health care in China: a nationwide cross-sectional survey.* Lancet, 2017. **390**(10112): p. 2559-2568.

35. Brouwers, S., et al., *Arterial hypertension.* Lancet, 2021.

36. Partridge, L., J. Deelen, and P.E. Slagboom, *Facing up to the global challenges of ageing.* Nature, 2018. **561**(7721): p. 45-56.

37. Beard, J.R., et al., *The World report on ageing and health: a policy framework for healthy ageing.* Lancet, 2016. **387**(10033): p. 2145-2154.

38. Global Burden of Disease Health Financing Collaborator, N., *Past, present, and future of global health financing: a review of development assistance, government, out-of-pocket, and other private spending on health for 195 countries, 1995-2050.* Lancet, 2019. **393**(10187): p. 2233-2260.

39. Dieleman, J.L., et al., *Factors Associated With Increases in US Health Care Spending, 1996-2013.* JAMA, 2017. **318**(17): p. 1668-1678.

For peer review only - http://bmjopen.bmj.com/site/about/guidelines.xhtml

40. The, L., *China's health-care reform: an independent evaluation.* Lancet, 2019. **394**(10204): p. 1113.
41. Jan, S., et al., *Action to address the household economic burden of non-communicable diseases.* Lancet, 2018. **391**(10134): p. 2047-2058.
42. Li, X., et al., *Quality of primary health care in China: challenges and recommendations.* Lancet, 2020. **395**(10239): p. 1802-1812.
43. Li, H. and L. Jiang, *Catastrophic medical insurance in China.* Lancet, 2017. **390**(10104): p. 1724-1725.
44. *NCD Countdown 2030: worldwide trends in non-communicable disease mortality and progress towards Sustainable Development Goal target 3.4.* Lancet, 2018. **392**(10152): p. 1072-1088.

For peer review only

For peer review only - http://bmjopen.bmj.com/site/about/guidelines.xhtml

Figure legends

Figure 1. Temporal population trends in China between 2000 and 2019.

Temporal population composition is presented according to gender and area (A), age group (B), birth rate, death rate, and natural growth rate (C), and gross ratio, children ratio, and old dependency ratio (D).

Supplemental appendix

sFigure 1. Trend in life expectancy and healthy life expectancy at birth and at 60–64 years according to gender between 2000 and 2019.

sTable 1. AAPC in life expectancy at birth and at 60–64 years for 2000–2019

sTable 2. Percentage of healthy life expectancy within total life expectancy at birth for 2000–2019

sTable 3. Percentage of healthy life expectancy within total life expectancy at 60–64 years for 2000–2019

sTable 4. AAPC of all-cause disease burden for 2000–2019

sTable 5. AAPC of causes for 2000–2019

sTable 6. AAPC of risk factors for 2000–2019

For peer review only

For peer review only - http://bmjopen.bmj.com/site/about/guidelines.xhtml

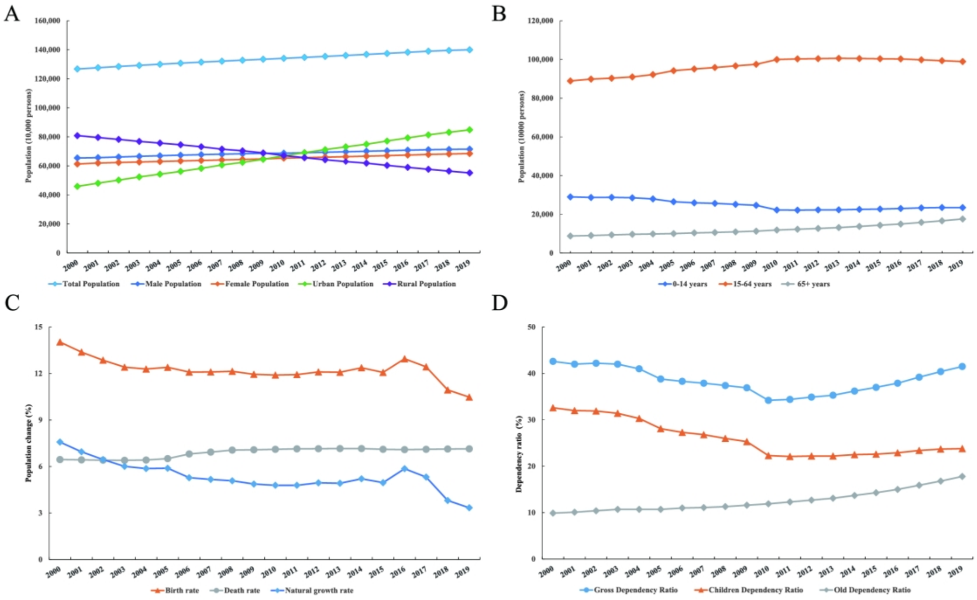

Temporal population trends in China between 2000 and 2019.

200x123mm (300 x 300 DPI)

Supplemental Appendix

Temporal trend of population structure, burden of diseases, healthcare resources and expenditure in China, 2000-2019

Table of Contents

Glossary and Definitions

Section 1: Population

Section 2: Health Care Institutions

Section 3: Medical Personnel

Section 4: Beds in Health Care Institutions

Section 5: Health Expenditures

Supplementary Figures

sFigure 1. Trend in life expectancy and healthy life expectancy at birth and at 60─64 years according to gender between 2000 and 2019.

Supplementary Tables

sTable 1. AAPC in life expectancy at birth and at 60─64 years for 2000─2019

sTable 2. Percentage of healthy life expectancy within total life expectancy at birth for 2000─2019

sTable 3. Percentage of healthy life expectancy within total life expectancy at 60─64 years for 2000─2019

sTable 4. AAPC of all-cause disease burden for 2000─2019

sTable 5. AAPC of causes for 2000─2019

sTable 6. AAPC of risk factors for 2000–2019

For peer review only

Glossary and Definitions

Glossary and definitions of population, healthcare institutions, medical personnel, beds in health care institutions, and health expenditure data were compiled from the 2020 China Statistical Yearbook (CSY) (http://www.stats.gov.cn/tjsj/ndsj/2020/indexeh.htm).

Section 1: Population

Total Population (year-end) (10000 persons): Year-end population refers to the population at midnight on 31 December. Annual statistics regarding the total population do not include data for Hong Kong, the Macau special administrative region and Taiwan province, and overseas numbers.

Urban Population refers to people residing in cities and towns in China.

Rural Population refers to people who reside outside of urban areas.

Birth Rate, Death Rate, and Natural Growth Rate of Population

Birth Rate (or Crude Birth Rate) refers to the ratio of the number of births to the average population (or mid-period population) during a certain period of time (usually a year). Birth rate in the chapter refers to annual birth rate and it is calculated as: number of births / annual average population × 1000. Number of births in the formula refers to live births (i.e., when a baby has breathed or showed any vital phenomena regardless of duration of gestation). Annual average population is the average of the population at the beginning of the year and the population at the end of the year. Sometimes it is substituted by the mid-year population.

Death Rate (or Crude Death Rate) refers to the ratio of the number of deaths to the average population (or mid-period population) during a certain period of time (usually a year). Death rate in the chapter refers to annual death rate and is calculated as: number of deaths / annual average population × 1000. Total Population (year-end) (10000 persons) refers to the population at midnight on 31 December.

Gross Dependency Ratio (or Coefficient) (%) refers to the ratio of non-working-age population to the working-age population. In general, Gross Dependency Ratio describes the number of non-working-age individuals that every 100 people of working age take care of. This reflects the basic relation between population and economic development from a demographic perspective.

Children Dependency Ratio (or Coefficient) (%) refers to the ratio of children to the working-age population. It describes the number of children that every 100 people of working age take care of.

Old Dependency Ratio (or Coefficient) (%) refers to the ratio of the elderly to the working-age population. It describes the number of elderly that every 100 people of working age take care of. Old dependency ratio is one of the indicators that reflect the social implication of population aging from an economic perspective.

Note: For the years, 2000 and 2010, census year estimates were available; the rest of the data were estimated based on annual national sample surveys.

Section 2: Health Care Institutions

Number of Health Care Institutions (unit)

Medical and health care institutions refer to units which have qualified to receive a Certification of Health Care Institution by the administration of public health, or a Certification of Corporate Unit by the civil affairs, administration for industry and commerce, commission office for public sector reform. Health care institutions engage in medical care, disease prevention and control, health supervision and inspection, medicine research, and on-job training. Health care institutions include hospitals, health care institutions at the grass-root level, specialized public health institutions, and other medical and health care institutions.

Number of Hospitals (unit)

Hospitals were considered to include general hospitals, hospitals of traditional Chinese medicine, hospitals that integrate traditional Chinese medicine and Western medicine, national hospitals, specialized hospitals, and nursing hospitals. Specialized disease prevention and cure centers, maternal and child health hospitals, and sanatoriums were excluded.

For peer review only

Health Care Institutions at Grass-root Level (unit)

Health care institutions at the grass-root level included: community health service centers, community service stations, institutes of health on the street, institutes of health in villages and towns, outpatient departments, and clinics (health centers).

Note:
1. Number of village clinics was included in health care institutions.
2. Since 2002, health care institutions no longer include headquarters of higher and secondary medical schools, drug test institutions, frontier health and quarantine institutions, and family.

Section 3: Medical Personnel

Number of Medical Personnel (10000 persons)

Medical personnel refer to all employees engaged at health care institutions, such as hospitals, health care institutions at grass-root level, specialized public health institutions, and other medical and health care institutions. These employees include medical technical personnel, village doctors and assistants, other technical personnel, and managerial and service staff. Medical personnel also refer to workers with payment of wages at the end of the year, including various types of employment personnel (including contract workers) and staff rehired for more than six months. Temporary workers, emeritus and retired, persons leaving the unit while remaining labor relations, and rehired and temporarily employed individuals for less than six months were excluded.

Number of Licensed Doctors (10000 persons)

Licensed doctors refer to medical workers who have obtained licenses of qualified doctors and are employed in medical treatment, disease prevention, or healthcare institutions. Licensed doctors engaged in management positions were excluded. Licensed doctors are divided into four categories: clinician, Chinese medicine physician, dentist, and public health physician.

Number of Registered Nurses (10000 persons)

Registered nurses refer to practical nursing personnel with a certificate of registered nurse. Nurses engaged in management positions were excluded.

Number of Other Technical Personnel (10000 persons)

Other technical personnel represent staff who graduated from high school and other institutions in the majors of chemistry and mathematics, as well as other non-health professionals, who are engaged in health promotion, research, teaching, and other technical work.

Number of Administrative Personnel (10000 persons)

Administrative personnel include persons in charge, personnel mainly engaged in health care, disease control, health surveillance, medical research, and teaching, other business management, and personnel mainly engaged in the party and government, personnel, finance, information, security, and other administrative work.

Note: Data of medical personnel and medical technical personnel include 10000 sanitation supervisors who serve as public servants.

Section 4: Beds in Health Care Institutions

Number of Beds in Health Care Institutions (10000 units)

Number of beds in health care institutions refers to a fixed sum of beds (non-prepared beds) at the end of the year. These include formal beds, simple beds, care beds, beds being disinfected and repaired, and disabled beds due to expansion or overhaul. Obstetric neonatal beds, expectant beds of confinement rooms, inventory beds, observation beds, extra beds, and escort beds for patient's family members were excluded.

Number of Beds in Medical Institutions per 10000 Population

Number of beds in medical institutions refers to a fixed sum of beds (non-prepared beds) at the end of the year. These include formal beds, simple beds, care beds, beds being disinfected and repaired, and disabled beds due to expansion or overhaul. Obstetric neonatal beds, expectant beds of confinement rooms, inventory beds, observation beds, extra beds, and escort beds for patient's family members were excluded.

For peer review only

Number of Beds in Urban Medical Institutions (10000 units)

Number of beds in urban medical institutions refers to a fixed sum of beds (non-prepared beds) at the end of the year. These include formal beds, simple beds, care beds, beds being disinfected and repaired, and disabled beds due to expansion or overhaul. Obstetric neonatal beds, expectant beds of confinement rooms, inventory beds, observation beds, extra beds, and escort beds for patient's family members were excluded.

Number of Beds in Rural Medical Institutions (10000 units)

Number of beds in rural medical institutions refers to a fixed sum of beds (non-prepared beds) at the end of the year. These include formal beds, simple beds, care beds, beds being disinfected and repaired, and disabled beds due to expansion or overhaul. Obstetric neonatal beds, expectant beds of confinement rooms, inventory beds, observation beds, extra beds, and escort beds for patient's family members were excluded.

Beds of Medical Institutions per 10000 Population

Beds of Medical Institutions per 10000 Population = number of beds of medical and health care institutions / population ×10000. The population value used is based on household registration data from the Ministry of Public Security.

Section 5: Health Expenditures

Total Health Expenditure (100 million yuan)

Total health expenditure refers to the total expenditure on medical and health care services (usually in monetary value) by a country or region within a certain period of time (usually a year). Total health expenditure is counted according to the source method. Under certain economic conditions, it reflects the emphasis and burden level of cost on government health care, the society’s healthcare, and on individual citizens. Total health expenditure also represents the main features of health financing mode and fairness, as well as the rationality of health financing.

Government Health Expenditure (100 million yuan)

Government expenditure on public health refers to expenditures by the government at all levels of medical and health care services, medical subsidies, health administration and health insurance management, and undertakings of family planning.

Social Health Expenditure (100 million yuan)

Social expenditure on public health refers to all inputs from society, and not from the government in the form of expenditures on social medical security, commercial health insurance, private expenditure on operation of medical and health care, social donations and contributions, and income from administrative fees.

Out-of-pocket Health Expenditure (100 million yuan)

Individual cash expenditure on health refers to the expenditure in cash by rural and urban residents for various health services, including self-payments of residents within the system of multi-medical insurance.

Per Capita Health Expenditure (yuan)

Per capita health expenditure refers to the ratio of total expenditure on health in a year to the average population.

For peer review only

Supplementary Figures

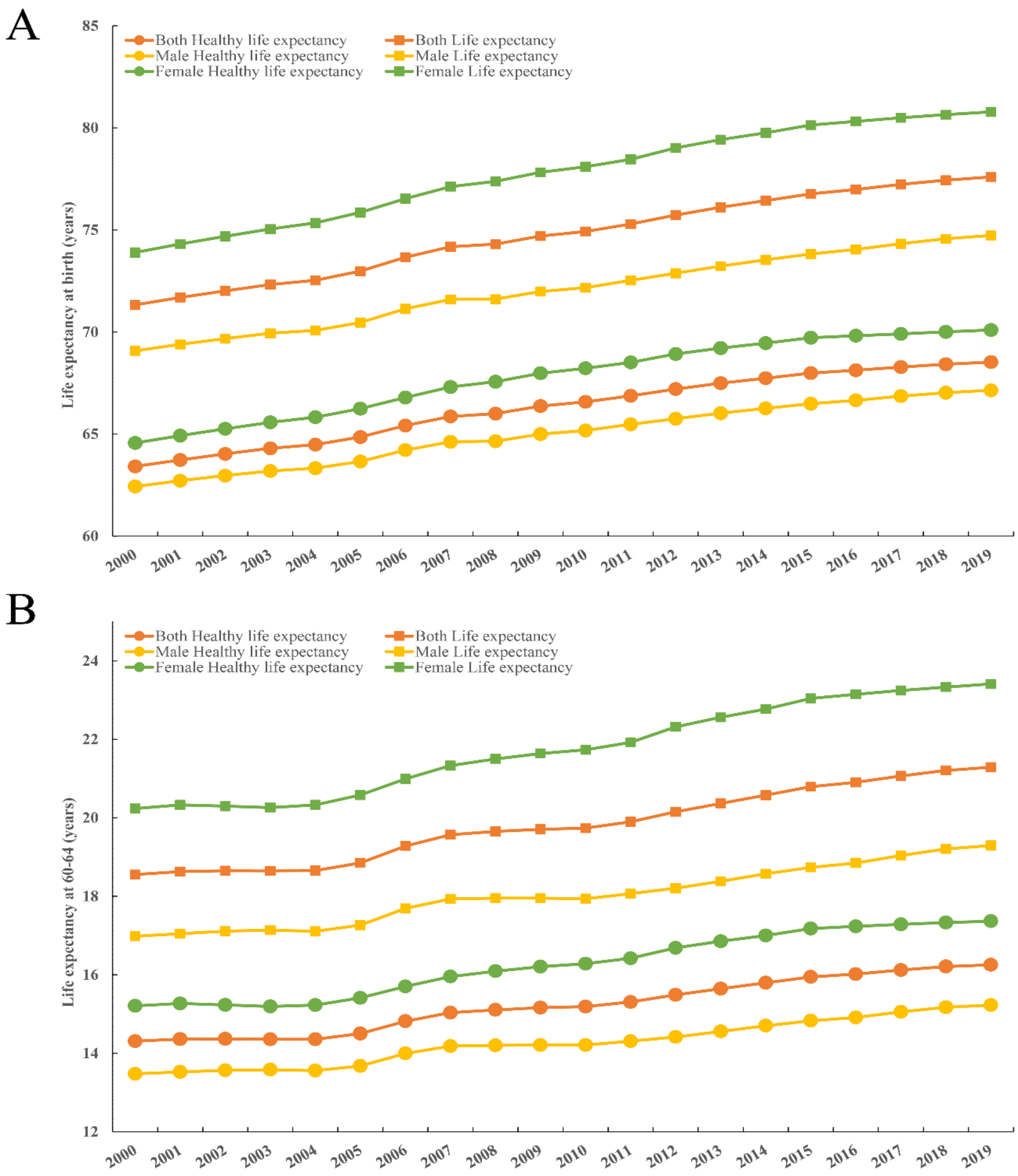

sFigure 1. Trend in life expectancy and healthy life expectancy at birth and at 60–64 years according to gender between 2000 and 2019.

The trend of life expectancy and healthy life expectancy at birth (A) and at 60–64 years (B) according to gender between 2000 and 2019. The distance between life expectancy and healthy life expectancy denotes unhealthy life expectancy (ill-span).

Supplementary Tables

sTable 1. AAPC in life expectancy at birth and at 60–64 years for 2000–2019

	Life expectancy (95% UI)		AAPC (95% CI)		
	2000	2019	2000–2019	2000–2009	2010–2019
**Life expectancy at birth**					
Both					
Life expectancy	71.33 (70.56 to 72.06)	77.59 (76.27 to 79.00)	0.44 (0.43 to 0.46)*	0.50 (0.47 to 0.53)*	0.40 (0.38 to 0.42)*
Healthy life expectancy	63.42 (60.07 to 65.54)	68.53 (60.88 to 70.97)	0.41 (0.38 to 0.43)*	0.50 (0.45 to 0.54)*	0.31 (0.29 to 0.34)*
Male					
Life expectancy	69.08 (68.02 to 70.16)	74.74 (72.78 to 76.70)	0.42 (0.38 to 0.46)*	0.46 (0.37 to 0.54)*	0.39 (0.37 to 0.41)*
Healthy life expectancy	62.43 (60.44 to 64.36)	67.14 (60.64 to 69.82)	0.38 (0.36 to 0.41)*	0.44 (0.39 to 0.49)*	0.32 (0.30 to 0.35)*
Female					
Life expectancy	73.90 (72.91 to 74.81)	80.78 (79.10 to 82.47)	0.47 (0.43 to 0.51)*	0.57 (0.50 to 0.63)*	0.37 (0.32 to 0.41)*
Healthy life expectancy	64.57 (60.86 to 66.96)	70.10 (60.92 to 73.19)	0.43 (0.40 to 0.46)*	0.56 (0.50 to 0.62)*	0.30 (0.27 to 0.33)*
**Life expectancy at 60–64 years**					
Both					
Life expectancy	18.56 (18.06 to 19.02)	21.29 (20.32 to 22.33)	0.76 (0.60 to 0.91)*	0.65 (0.37 to 0.93)*	0.91 (0.81 to 1.01)*
Healthy life expectancy	14.31 (13.16 to 15.43)	16.26 (14.82 to 17.67)	0.69 (0.51 to 0.87)*	0.64 (0.27 to 1.01)*	0.73 (0.66 to 0.80)*
Male					
Life expectancy	16.99 (16.32 to 17.66)	19.29 (17.97 to 20.68)	0.68 (0.61 to 0.74)*	0.58 (0.46 to 0.70)*	0.85 (0.80 to 0.89)*
Healthy life expectancy	13.48 (12.42 to 14.53)	15.23 (13.82 to 16.79)	0.65 (0.55 to 0.76)*	0.56 (0.37 to 0.75)*	0.81 (0.76 to 0.86)*
Female					
Life expectancy	20.24 (19.62 to 20.86)	23.41 (22.10 to 24.77)	0.76 (0.55 to 0.98)*	0.75 (0.41 to 1.09)*	0.76 (0.47 to 1.04)*
Healthy life expectancy	15.21 (13.83 to 16.46)	17.37 (15.61 to 19.16)	0.70 (0.54 to 0.85)*	0.69 (0.40 to 0.98)*	0.68 (0.54 to 0.81)*

Note: AAPC: average annual percent changes; UI, uncertainty interval; CI, confidence interval.
*AAPC significantly differs from zero at the alpha = 0.05 level.

For peer review only

sTable 2. Percentage of healthy life expectancy within total life expectancy at birth for 2000–2019

Year	Healthy life expectancy at birth (years)			Life expectancy at birth (years)			Healthy within total life expectancy (%)		
	Total	Male	Female	Total	Male	Female	Total	Male	Female
2000	63.42	62.43	64.57	71.33	69.08	73.90	88.90	90.38	87.37
2001	63.73	62.72	64.92	71.69	69.40	74.31	88.90	90.37	87.37
2002	64.03	62.96	65.26	72.02	69.67	74.69	88.90	90.37	87.38
2003	64.30	63.20	65.57	72.33	69.94	75.05	88.89	90.36	87.38
2004	64.49	63.33	65.83	72.54	70.08	75.34	88.90	90.37	87.37
2005	64.86	63.66	66.25	72.98	70.47	75.86	88.87	90.34	87.33
2006	65.42	64.22	66.79	73.67	71.14	76.54	88.80	90.27	87.27
2007	65.87	64.62	67.31	74.19	71.60	77.12	88.79	90.25	87.27
2008	66.00	64.65	67.57	74.31	71.61	77.38	88.83	90.28	87.32
2009	66.38	65.00	67.98	74.70	71.99	77.82	88.85	90.30	87.35
2010	66.58	65.18	68.22	74.92	72.17	78.10	88.87	90.31	87.36
2011	66.88	65.48	68.51	75.29	72.53	78.46	88.82	90.27	87.31
2012	67.21	65.75	68.92	75.73	72.88	79.03	88.75	90.22	87.21
2013	67.50	66.03	69.21	76.11	73.23	79.42	88.69	90.17	87.14
2014	67.74	66.26	69.45	76.44	73.53	79.76	88.62	90.11	87.08
2015	67.99	66.49	69.72	76.77	73.83	80.14	88.56	90.06	87.00
2016	68.12	66.65	69.82	76.98	74.05	80.32	88.49	90.01	86.93
2017	68.28	66.86	69.91	77.23	74.33	80.50	88.41	89.95	86.85
2018	68.42	67.03	70.01	77.44	74.57	80.65	88.36	89.89	86.81
2019	68.53	67.14	70.10	77.59	74.74	80.78	88.31	89.84	86.78

For peer review only

sTable 3. Percentage of healthy life expectancy within total life expectancy at 60–64 years for 2000–2019

Year	Healthy life expectancy at 60–64 years			Life expectancy at 60–64 year			Percentage of healthy within total life expectancy		
	Total	Male	Female	Total	Male	Female	Total	Male	Female
2000	14.31	13.48	15.21	18.56	16.99	20.24	77.12%	79.35%	75.15%
2001	14.36	13.52	15.27	18.63	17.05	20.33	77.09%	79.33%	75.11%
2002	14.37	13.57	15.23	18.65	17.11	20.30	77.05%	79.29%	75.06%
2003	14.36	13.58	15.19	18.65	17.14	20.26	76.99%	79.24%	74.98%
2004	14.36	13.56	15.23	18.66	17.11	20.33	76.97%	79.24%	74.93%
2005	14.50	13.68	15.41	18.85	17.27	20.58	76.93%	79.21%	74.87%
2006	14.82	14.00	15.70	19.29	17.69	20.99	76.84%	79.12%	74.80%
2007	15.04	14.18	15.95	19.57	17.93	21.33	76.82%	79.09%	74.79%
2008	15.11	14.20	16.09	19.65	17.95	21.50	76.87%	79.12%	74.84%
2009	15.16	14.21	16.21	19.71	17.95	21.64	76.93%	79.17%	74.90%
2010	15.19	14.21	16.28	19.74	17.94	21.73	76.96%	79.21%	74.93%
2011	15.31	14.31	16.42	19.90	18.07	21.93	76.94%	79.20%	74.89%
2012	15.49	14.42	16.69	20.15	18.21	22.32	76.87%	79.19%	74.78%
2013	15.65	14.56	16.86	20.37	18.39	22.56	76.82%	79.18%	74.71%
2014	15.80	14.71	17.00	20.58	18.58	22.77	76.77%	79.16%	74.65%
2015	15.95	14.83	17.18	20.79	18.74	23.04	76.69%	79.13%	74.55%
2016	16.02	14.91	17.23	20.91	18.85	23.15	76.62%	79.11%	74.45%
2017	16.12	15.05	17.28	21.07	19.04	23.25	76.54%	79.08%	74.35%
2018	16.21	15.17	17.33	21.21	19.21	23.33	76.45%	78.99%	74.28%
2019	16.26	15.23	17.37	21.29	19.29	23.41	76.36%	78.93%	74.18%

For peer review only

sTable 4. AAPC of all-cause disease burden for 2000–2019

Subcategory	All-cause estimates (95% UI)		AAPC (95% CI)		
	2000	2019	2000–2019	2000–2009	2010–2019
**Number**					
Incidence	5,291,848,308 (4,993,009,197 to 5,644,956,487)	5,790,243,111 (5,505,879,019 to 6,107,248,437)	0.48 (0.45 to 0.50)*	0.19 (0.16 to 0.22)*	0.82 (0.78 to 0.85)*
Prevalence	1,241,468,252 (1,234,531,622 to 1,247,534,242)	1,362,817,881 (1,356,594,402 to 1,368,714,232)	0.49 (0.49 to 0.50)*	0.47 (0.47 to 0.48)*	0.52 (0.51 to 0.52)*
Death	8,653,295 (8,170,487 to 9,200,531)	10,653,448 (9,310,978 to 12,069,760)	1.12 (0.93 to 1.31)*	0.89 (0.61 to 1.18)*	1.27 (1.03 to 1.51)*
Years lived with disability	116,137,933 (86,494,934 to 150,490,082)	153,797,874 (115,409,603 to 199,426,123)	1.49 (1.46 to 1.52)*	1.29 (1.24 to 1.34)*	1.74 (1.72 to 1.77)*
Years of life lost	260,536,746 (246,148,674 to 276,354,908)	228,407,694 (197,155,841 to 260,733,993)	-0.70 (-0.95 to -0.46)*	-1.34 (-1.53 to -1.15)*	-0.07 (-0.51 to 0.38)
DALY	376,674,679 (343,098,150 to 416,187,725)	382,205,568 (332,307,099 to 433,824,950)	0.08 (-0.06 to 0.22)	-0.44 (-0.58 to -0.31)*	0.60 (0.39 to 0.82)*
Risk factor (DALY)	176,792,905 (163,998,804 to 190,556,819)	187,705,961 (165,023,927 to 212,244,649)	0.34 (0.20 to 0.48)*	-0.27 (-0.52 to -0.03)*	0.92 (0.79 to 1.06)*
**Age standardized rate**					
Incidence	414,111 (390,563 to 440,957)	418,081 (394,196 to 444,709)	0.04 (0.02 to 0.07)*	-0.21 (-0.24 to -0.17)*	0.34 (0.30 to 0.38)*
Prevalence	94,824 (94,187 to 95,367)	93,439 (92,723 to 94,090)	-0.08 (-0.09 to -0.07)*	-0.17 (-0.19 to -0.15)*	0.00 (-0.01 to 0.00)
Death	956 (910 to 1,010)	634 (561 to 711)	-2.14 (-2.28 to -2.00)*	-2.00 (-2.20 to -1.79)*	-2.38 (-2.56 to -2.21)*
Years lived with disability	9,078 (6,779 to 11,713)	8,924 (6,654 to 11,539)	-0.08 (-0.12 to -0.05)*	-0.34 (-0.38 to -0.30)*	0.22 (0.17 to 0.28)*
Years of life lost	24,833 (23,513 to26,239)	13,347 (11,701 to14,997)	-3.22 (-3.33 to -3.11)*	-3.43 (-3.59 to -3.27)*	-3.09 (-3.23 to -2.96)*
DALY	33,911 (31,131 to 37,035)	22,271 (19,482 to 25,195)	-2.21 (-2.35 to -2.07)*	-2.54 (-2.64 to -2.43)*	-1.85 (-2.12 to -1.58)*
Risk factor (DALY)	16,689 (15,569 to 17,886)	10,310 (9,122 to 11,524)	-2.49 (-2.69 to -2.29)*	-2.93 (-3.27 to -2.58)*	-2.07 (-2.30 to -1.85)*

Note: AAPC: average annual percent changes; UI, uncertainty interval; CI, confidence interval; DALY, disability-adjusted life years. *AAPC significantly differs from zero at the alpha = 0.05 level.

For peer review only

sTable 5. AAPC of causes for 2000–2019

Causes	DALY (95% UI)				AAPC (95% CI)		
	2000	Rank	2019	Rank	2000–2019	2000–2009	2010–2019
**All-age numbers**							
Neglected tropical diseases and malaria	1,704,065 (1,121,580 to 2,525,119)	21	1,137,806 (692, 480 to 1,800,439)	22	-2.07 (-2.35 to -1.79)*	-3.57 (-3.89 to -3.25)*	-0.50 (-0.97 to -0.02)*
Nutritional deficiencies	4,183,588 (3,082,941 to 5,605,252)	19	2,606,926 (1,815,566 to 3,659,294)	18	-2.47 (-2.64 to -2.30)*	-4.30 (-4.49 to -4.11)*	-0.53 (-0.79 to -0.26)*
Neoplasms	54,415,394 (50,247,614 to 58,721,812)	2	67,519,830 (1,418,891 to 3,248,407)	2	1.13 (0.95 to 1.31)*	0.87 (0.57 to 1.17)*	1.42 (1.20 to 1.65)*
Cardiovascular diseases	67,584,492 (62,771,497 to 73,832,854)	1	91,933,122 (1,135,346 to 3,124,922)	1	1.62 (1.45 to 1.79)*	1.70 (1.40 to 2.00)*	1.42 (1.32 to 1.53)*
Chronic respiratory diseases	28,653,966 (23,770,991 to 30,931,146)	3	22,520,549 (1,719,779 to 3,416,880)	4	-1.28 (-1.50 to -1.06)*	-2.02 (-2.40 to -1.65)*	-0.50 (-0.75 to -0.26)*
Digestive diseases	11,699,071 (10,590,221 to 12,920,126)	12	10,017,880 (1,549,912 to 3,496,363)	12	-0.80 (-0.87 to -0.73)*	-1.02 (-1.13 to -0.90)*	-0.51 (-0.60 to -0.43)*
Neurological disorders	12,506,303 (6,994,212 to 20,592,252)	11	17,381,406 (1,666,405 to 3,082,962)	7	1.75 (1.67 to 1.82)*	1.79 (1.70 to 1.89)*	1.72 (1.61 to 1.84)*
Mental disorders	18,110,029 (13,449,072 to 23,810,039)	9	20,293,827 (1,145,615 to 3,345,133)	5	0.59 (0.49 to 0.69)*	0.45 (0.40 to 0.49)*	0.75 (0.54 to 0.95)*
Musculoskeletal disorders	20,089,552 (14,362,360 to 26,771,117)	7	29,933,924 (1,427,199 to 3,823,644)	3	2.10 (2.04 to 2.16)*	2.44 (2.36 to 2.52)*	1.75 (1.67 to 1.83)*
Other non-communicable diseases	24,572,991 (20,333,568 to 29,891,261)	4	19,126,072 (1,446,224 to 3,590,726)	6	-1.31 (-1.48 to -1.13)*	-2.43 (-2.53 to -2.33)*	-0.04 (-0.40 to -032)
Skin and subcutaneous diseases	7,809,025 (5,108,660 to 11,567,830)	16	8,264,702 (1,394,816 to 3,356,265)	13	0.29 (0.27 to 0.32)*	0.33 (0.29 to 0.38)*	0.28 (0.26 to 0.29)*
Sense organ diseases	10,740,782 (7,311,356 to 15,203,366)	14	15,981,667 (1,788,420 to 3,997,480)	9	2.14 (1.98 to 2.30)*	1.98 (1.94 to 2.03)*	2.31 (1.97 to 2.64)*
Transport injuries	16,631,845 (15,221,113 to 18,940,342)	10	13,745,455 (1,719,684 to 3,768,996)	11	-1.02 (-1.35 to -0.69)*	0.57 (0.21 to 0.95)*	-2.66 (-3.22 to -2.10)*
Unintentional injuries	21,114,052 (19,362,287 to 23,062,953)	6	16,003,462 (1,056,699 to 3,613,615)	8	-1.53 (-2.01 to -1.04)*	-1.53 (-2.01 to -1.04)*	-1.53 (-2.01 to -1.04)*
Self-harm and interpersonal violence	11,171,276 (9,775,023 to 12,180,298)	13	5,870,049 (1,030,733 to 3,924,034)	16	-3.35 (-3.70 to -3.01)*	-4.09 (-4.73 to -3.45)*	-2.47 (-2.75 to -2.18)*
HIV/AIDS and sexually transmitted infections	921,444,444 (727,466,466 to 1,222,963)	22	1,752,419 (1,430, 192 to 2,114,213)	19	3.47 (2.72 to 4.22)*	3.07 (2.38 to 3.77)*	4.07 (2.71 to 5.45)*
Respiratory infections and tuberculosis	21,938,222 (20,220,847 to 23,581,022)	5	6,647,872 (1,853,547 to 3,613,257)	15	-6.10 (-6.41 to -5.79)*	-8.88 (-9.36 to -8.40)*	-3.34 (-3.65 to -3.03)*
Enteric infections	3,407,267 (2,990,655 to 3,948,769)	20	1,408,098 (1,047,418 to 3,827,650)	20	-4.41 (-5.28 to -3.54)*	-9.05 (-9.96 to -8.13)*	0.68 (-0.56 to 1.92)
Other infectious diseases	4,581,989 (3,724,438 to 6,219,671)	18	1,244,099 (1,063,407 to 3,462,432)	21	-6.57 (-6.95 to -6.19)*	-9.03 (-9.45 to -8.61)*	-4.04 (-4.71 to -3.36)*
Maternal and neonatal disorders	18,110,589 (16,543,873 to 19,663,056)	8	7,151,571 (1,273,977 to 3,174,922)	14	-4.82 (-5.40 to -4.23)*	-6.06 (-6.60 to -5.51)*	-3.56 (-4.60 to -2.51)*
Substance use disorders	6,291,914 (5,043,188 to 7,751,372)	17	5,755,658 (1,373,805 to 3,343,432)	17	-0.39 (-0.82 to 0.03)	-1.74 (-2.49 to -0.99)*	0.94 (0.53 to 1.36)*
Diabetes and kidney diseases	10,436,824 (9,083,252 to 11,910,785)	15	15,909,173 (1,346,885 to 3,824,247)	10	2.30 (2.09 to 2.51)*	2.87 (2.44 to 3.31)*	1.79 (1.70 to 1.89)*
**Age-standardized rate**							
Neglected tropical diseases and malaria	132 (89 to 194)	21	68 (42 to 107)	22	-3.40 (-3.66 to -3.13)*	-4.75 (-5.06 to -4.45)*	-1.97 (-2.42 to -1.51)*
Nutritional deficiencies	369 (284 to 480)	20	175 (122 to 241)	18	-3.88 (-4.07 to -3.69)*	-5.98 (-6.27 to -5.70)*	-1.84 (-2.11 to -1.58)*
Neoplasms	4,505 (4,163 to 4,847)	2	3,421 (2,933 to 3,946)	2	-1.46 (-1.71 to -1.21)*	-1.62 (-1.88 to -1.36)*	-1.25 (-1.68 to -1.81)*
Cardiovascular diseases	6,441 (5,997 to 7,005)	1	4,938 (4,318 to 5,577)	1	-1.40 (-1.56 to -1.24)*	-1.10 (-1.39 to -0.82)*	-1.84 (-1.94 to -1.75)*
Chronic respiratory diseases	2,947 (2,458 to 3,175)	3	1,270 (1,114 to 1,474)	5	-4.34 (-4.61 to -4.07)*	-4.90 (-5.37 to -4.44)*	-3.74 (-4.04 to -3.45)*
Digestive diseases	974 (889 to 1,070)	12	539 (463 to 616)	14	-3.07 (-3.14 to -3.00)*	-3.29 (-3.41 to -3.16)*	-2.77 (-2.84 to -2.71)*
Neurological disorders	1,103 (650 to 1,773)	11	1,077 (598 to 1,780)	8	-0.15 (-0.24 to -0.06)*	-0.09 (-0.16 to -0.02)*	-0.22 (-0.40 to -0.03)*
Mental disorders	1,317 (978 to 1,727)	9	1,248 (922 to 1,627)	6	-0.27 (-0.34 to -0.20)*	-0.56 (-0.59 to -0.53)*	0.01 (-0.13 to 0.14)

For peer review only

Musculoskeletal disorders	1,536 (1,102 to 2,050)	8	1,585 (1,140 to 2,112)	3	0.16 (0.10 to 0.23)*	0.31 (0.26 to 0.36)*	0.02 (-0.11 to 0.15)
Other non-communicable diseases	2,315 (1,963 to 2,719)	5	1,349 (1,066 to 1,726)	4	-2.81 (-2.96 to -2.67)*	-3.49 (-3.65 to -3.34)*	-2.00 (-2.25 to -1.75)*
Skin and subcutaneous diseases	606 (397 to 898)	16	606 (391 to 909)	13	0.00 (-0.01 to 0.01)	-0.05 (-0.07 to -0.04)*	0.06 (0.03 to 0.08)*
Sense organ diseases	926 (633 to 1,299)	13	863 (591 to 1,226)	9	-0.36 (-0.45 to -0.27)*	-0.46 (-0.49 to -0.43)*	-0.25 (-0.43 to -0.07)*
Transport injuries	1,248 (1,146 to 1,433)	10	860 (740 to 975)	10	-1.98 (-2.42 to -1.54)*	-0.63 (-1.14 to -0.13)*	-3.39 (-4.12 to -2.65)*
Unintentional injuries	1,886 (1,744 to 2,045)	7	1,102 (919 to 1,254)	7	-2.81 (-3.24 to -2.39)*	-2.81 (-3.24 to -2.39)*	-2.81 (-3.24 to -2.39)*
Self-harm and interpersonal violence	807 (708 to 877)	15	361 (310 to 421)	16	-4.19 (-4.62 to -3.76)*	-5.13 (-5.93 to -4.32)*	-3.08 (-3.43 to -2.73)*
HIV/AIDS and sexually transmitted infections	78 (56 to 114)	22	116 (92 to 147)	19	2.02 (1.50 to 2.53)*	2.61 (2.31 to 2.91)*	1.88 (1.05 to 2.72)*
Respiratory infections and tuberculosis	2,396 (2,209 to 2,582)	4	518 (458 to 593)	15	-7.77 (-7.93 to -7.61)*	-9.56 (-9.70 to -9.41)*	-5.88 (-6.18 to -5.58)*
Enteric infections	369 (326 to 422)	19	111 (84 to 144)	20	-6.04 (-6.52 to -5.56)*	-10.47 (-11.00 to -9.94)*	-1.52 (-2.31 to -0.73)*
Other infectious diseases	473 (376 to 659)	17	110 (92 to 131)	21	-7.37 (-7.84 to -6.91)*	-8.51 (-9.21 to -7.80)*	-6.04 (-6.58 to -5.50)*
Maternal and neonatal disorders	2,177 (1,983 to 2,375)	6	792 (699 to 897)	12	-5.26 (-5.36 to -5.16)*	-5.26 (-5.36 to -5.16)*	-5.26 (-5.36 to -5.16)*
Substance use disorders	428 (345 to 526)	18	347 (262 to 443)	17	-1.03 (-1.38 to -0.69)*	-2.60 (-3.02 to -2.18)*	0.85 (0.35 to 1.36)*
Diabetes and kidney diseases	876 (764 to 997)	14	817 (688 to 961)	11	-0.32 (-0.47 to -0.16)*	0.46 (0.17 to 0.75)*	-1.01 (-1.12 to -0.90)*

Note: AAPC: average annual percent changes; UI, uncertainty interval; CI, confidence interval.
*AAPC significantly differs from zero at the alpha = 0.05 level.

For peer review only

sTable 6. AAPC of risk factors for 2000–2019

Risk Factors	DALY (95% UI)				AAPC (95% CI)		
	2000	Rank	2019	Rank	2000–2019	2000–2009	2010–2019
**All-age numbers**							
Unsafe water, sanitation, and handwashing	4,234,714 (3,363,558 to 5,211,019)	15	1,152,504 (805,846 to 1,554,455)	18	-6.60 (-7.28 to -5.91)*	-10.80 (-11.04 to -10.55)*	-2.34 (-3.77 to -0.88)*
Air pollution	49,335,959 (43,328,149 to 55,138,636)	2	42,509,703 (36,337,918 to 49,482,918)	4	-0.78 (-0.98 to -0.58)*	-1.17 (-1.53 to -0.82)*	-0.46 (-0.68 to -0.24)*
Other environmental risks	5,386,702 (3,749,375 to 7,246,194)	14	6,306,070 (4,075,171 to 8,773,453)	13	0.85 (0.72 to 0.98)*	1.14 (0.91 to 1.38)*	0.46 (0.38 to 0.53)*
Child and maternal malnutrition	27,730,286 (24,920,872 to 30,776,034)	5	7,253,783 (6,277,469 to 8,340,678)	12	-6.81 (-7.62 to -5.99)*	-8.92 (-9.38 to -8.47)*	-4.75 (-6.37 to -3.10)*
Tobacco	57,264,585 (52,395,093 to 62,486,440)	1	64,116,000 (54,575,903 to 75,181,983)	1	0.60 (0.42 to 0.78)*	0.30 (0.02 to 0.58)*	0.90 (0.66 to 1.15)*
Alcohol use	14,673,585 (12,536,787 to 17,097,523)	8	17,265,060 (13,765,337 to 21,321,000)	8	0.77 (0.68 to 0.85)*	0.77 (0.68 to 0.85)*	0.77 (0.68 to 0.85)*
Drug use	6,301,278 (5,433,686 to 7,296,963)	13	5,121,794 (4,187,484 to 6,121,198)	14	-1.03 (-1.22 to -0.84)*	-3.28 (-3.50 to -3.07)*	1.54 (1.21 to 1.87)*
High fasting plasma glucose	17,924,145 (14,527,382 to 21,999,850)	7	28,228,439 (22,052,698 to 35,375,022)	5	2.36 (1.82 to 2.90)*	3.64 (2.83 to 4.47)*	1.11 (0.41 to 1.81)*
High systolic blood pressure	35,341,494 (30,227,215 to 40,383,079)	3	54,441,615 (45,474,002 to 63,689,070)	2	2.32 (2.18 to 2.46)*	2.66 (2.40 to 2.91)*	1.84 (1.77 to 1.92)*
High body-mass index	11,704,488 (3,880,854 to 22,527,461)	9	24,830,041 (11,788,976 to 40,545,899)	6	4.02 (3.74 to 4.29)*	4.33 (3.83 to 4.83)*	3.59 (3.48 to 3.70)*
Low bone mineral density	1,769,378 (1,477,619 to 2,061,186)	16	3,320,275 (2,594,480 to 4,004,265)	15	3.39 (3.12 to 3.67)*	4.14 (3.73 to 4.56)*	2.78 (2.42 to 3.15)*
Dietary risks	34,253,322 (27,427,086 to 43,429,953)	4	46,813,126 (35,644,728 to 60,011,858)	3	1.66 (1.53 to 1.78)*	1.97 (1.74 to 2.21)*	1.28 (1.21 to 1.35)*
Low physical activity	1,275,222 (632,556 to 2,484,126)	18	2,507,120 (1,195,139 to 4,842,346)	17	3.54 (2.70 to 4.38)*	3.88 (2.39 to 5.39)*	3.03 (2.65 to 3.41)*
Occupational risks	18,571,331 (16,040,002 to 21,379,719)	6	15,486,849 (12,897,016 to 18,259,216)	9	-0.97 (-1.12 to -0.82)*	-1.51 (-1.78 to -1.24)*	-0.40 (-0.52 to -0.29)*
Intimate partner violence	789,841 (433,187 to 1,234,095)	19	789,926,926 (354,381 to 1,304,433)	19	0.07 (-0.17 to 0.30)	-0.50 (-0.78 to -0.22)*	0.76 (0.39 to 1.14)*
Unsafe sex	1,482,696 (1,293,280 to 1,925,395)	17	2,724,797 (1,976,323 to 3,277,196)	16	3.20 (2.90 to 3.49)*	3.72 (3.50 to 3.94)*	2.95 (2.39 to 3.51)*
Non-optimal temperature	8,427,319 (7,191,510 to 9,779,073)	12	9,325,364 (7,641,727 to 11,224,758)	11	0.44 (-0.88 to 1.77)	-0.61 (-1.39 to 0.18)	1.14 (-1.01 to 3.34)
Kidney dysfunction	8,884,203 (7,881,193 to 10,012,008)	11	13,353,909 (11,150,932 to 15,771,793)	10	2.25 (1.83 to 2.67)*	3.04 (2.37 to 3.71)*	1.47 (0.91 to 2.03)*
High LDL cholesterol	11,217,788 (9,135,992 to 13,780,576)	10	19,813,962 (15,205,317 to 25,139,359)	7	3.11 (2.85 to 3.36)*	3.86 (3.39 to 4.33)*	2.23 (2.08 to 2.37)*
Childhood sexual abuse and bullying	762,744 (311,431 to 1,443,447)	20	646,857,857 (278,772 to 1,183,126)	20	-0.86 (-0.97 to -0.75)*	-1.17 (-1.29 to -1.04)*	-0.40 (-0.58 to -0.22)*
**Age-standardized rate**							
Unsafe water, sanitation, and handwashing	475 (376 to 587)	14	91 (64 to 122)	18	-8.23 (-8.79 to -7.67)*	-11.69 (-12.52 to -10.85)*	-4.58 (-5.22 to -3.93)*
Air pollution	4,860 (4,243 to 5,438)	2	2,265 (1,950 to 2,615)	4	-3.93 (-4.15 to -3.70)*	-4.08 (-4.49 to -3.68)*	-3.86 (-3.97 to -3.74)*
Other environmental risks	488 (338 to 653)	13	331 (213 to 460)	13	-2.00 (-2.13 to -1.87)*	-1.49 (-1.73 to -1.25)*	-2.64 (-2.71 to -2.56)*
Child and maternal malnutrition	3,285 (2,955 to 3,649)	4	786 (690 to 899)	10	-7.24 (-7.44 to -7.04)*	-8.34 (-8.58 to -8.10)*	-6.13 (-6.41 to -5.85)*
Tobacco	5,287 (4,858 to 5,765)	1	3,228 (2,759 to 3,767)	1	-2.57 (-2.83 to -2.32)*	-2.80 (-3.20 to -2.40)*	-2.33 (-2.68 to -1.98)*
Alcohol use	1,137 (972 to 1,328)	8	908 (729 to 1,116)	8	-1.18 (-1.50 to -0.86)*	-1.41 (-1.54 to -1.28)*	-0.92 (-1.56 to -0.27)*
Drug use	449 (390 to 518)	15	295 (238 to 361)	14	-2.11 (-2.74 to -1.48)*	-4.70 (-4.97 to -4.43)*	0.85 (-0.48 to 2.19)
High fasting plasma glucose	1,647 (1,338 to 2,051)	6	1,452 (1,139 to 1,832)	5	-0.72 (-1.23 to -0.20)*	0.72 (-0.06 to 1.51)	-2.11 (-2.76 to -1.44)*
High systolic blood pressure	3,304 (2,820 to 3,777)	3	2,844 (2,392 to 3,321)	2	-0.77 (-0.89 to -0.65)*	-0.29 (-0.51 to -0.07)*	-1.40 (-1.47 to -1.34)*
High body-mass index	962 (315 to 1,891)	10	1,231 (578 to 2,023)	6	1.28 (0.95 to 1.60)*	1.50 (0.91 to 2.10)*	0.94 (0.82 to 1.07)*

For peer review only

For peer review only - http://bmjopen.bmj.com/site/about/guidelines.xhtml

Low bone mineral density	164 (137 to 192)	16	177 (138 to 214)	15	0.40 (0.27 to 0.54)*	0.70 (0.55 to 0.86)*	0.20 (-0.02 to 0.42)
Dietary risks	3,022 (2,391 to 3,875)	5	2,394 (1,823 to 3,071)	3	-1.23 (-1.36 to -1.09)*	-0.78 (-1.03 to -0.52)*	-1.75 (-1.83 to -1.68)*
Low physical activity	149 (74 to 287)	17	146 (70 to 280)	16	-0.19 (-0.74 to 0.36)	0.48 (0.17 to 0.79)*	-0.93 (-2.03 to 0.18)
Occupational risks	1,482 (1,277 to 1,700)	7	844 (701 to 997)	9	-2.95 (-3.08 to -2.81)*	-3.67 (-3.88 to -3.45)*	-2.22 (-2.37 to -2.07)*
Intimate partner violence	55 (30 to 86)	19	46 (22 to 76)	20	-0.86 (-1.11 to -0.62)*	-1.68 (-1.94 to -1.41)*	0.09 (-0.32 to 0.50)
Unsafe sex	108 (94 to 141)	18	142 (106 to 170)	17	1.47 (0.88 to 2.06)*	1.83 (1.43 to 2.23)*	1.34 (0.19 to 2.51)*
Non-optimal temperature	936 (813 to 1,075)	11	483 (399 to 582)	12	-3.61 (-4.04 to -3.18)*	-4.39 (-5.06 to -3.71)*	-2.91 (-3.41 to -2.42)*
Kidney dysfunction	781 (690 to 882)	12	709 (593 to 832)	11	-0.45 (-0.97 to 0.08)	054 (-0.31 to 1.39)	-1.41 (-2.10 to -0.72)*
High LDL cholesterol	1,005 (790 to 1,284)	9	1,052 (800 to 1,345)	7	0.28 (0.10 to 0.45)*	1.19 (0.87 to 1.51)*	-0.75 (-0.85 to -0.66)*
Childhood sexual abuse and bullying	54 (22 to 102)	20	50 (20 to 91)	19	-0.41 (-0.51 to -0.31)*	-1.05 (-1.21 to -0.89)*	0.40 (0.31 to 0.48)*

Note: AAPC: average annual percent changes; UI, uncertainty interval; CI, confidence interval; LDL, low-density lipoprotein.
*AAPC significantly differs from zero at the alpha = 0.05 level.

For peer review only

STROBE Statement—Checklist of items that should be included in reports of ***cohort studies***

	Item No	Recommendation
**Title and abstract**	1	(*a*) Indicate the study's design with a commonly used term in the title or the abstract. (Line 25-26, page 2)
		(*b*) Provide in the abstract an informative and balanced summary of what was done and what was found. (Line 30-53, page 2-3)
**Introduction**		
Background/rationale	2	Explain the scientific background and rationale for the investigation being reported. (Line70-72, page 5)
Objectives	3	State specific objectives, including any prespecified hypotheses. (Line102-106, page 6)
**Methods**		
Study design	4	Present key elements of study design early in the paper
Setting	5	Describe the setting, locations, and relevant dates, including periods of recruitment, exposure, follow-up, and data collection
Participants	6	(*a*) Give the eligibility criteria, and the sources and methods of selection of participants. Describe methods of follow-up
		(*b*) For matched studies, give matching criteria and number of exposed and unexposed
Variables	7	Clearly define all outcomes, exposures, predictors, potential confounders, and effect modifiers. Give diagnostic criteria, if applicable
Data sources/ measurement	8*	For each variable of interest, give sources of data and details of methods of assessment (measurement). Describe comparability of assessment methods if there is more than one group. (Line 110-128, page 6-7)
Bias	9	Describe any efforts to address potential sources of bias
Study size	10	Explain how the study size was arrived at
Quantitative variables	11	Explain how quantitative variables were handled in the analyses. If applicable, describe which groupings were chosen and why
Statistical methods	12	(*a*) Describe all statistical methods, including those used to control for confounding. (Line144-153, page 8)
		(*b*) Describe any methods used to examine subgroups and interactions. (Line153-156, page 6)
		(*c*) Explain how missing data were addressed
		(*d*) If applicable, explain how loss to follow-up was addressed
		(*e*) Describe any sensitivity analyses
**Results**		
Participants	13*	(a) Report numbers of individuals at each stage of study—eg numbers potentially eligible, examined for eligibility, confirmed eligible, included in the study, completing follow-up, and analysed.
		(b) Give reasons for non-participation at each stage. (Line161-162， page 9 and line 185-186, page 11 and line 207-209 page 14)
		(c) Consider use of a flow diagram
Descriptive data	14*	(a) Give characteristics of study participants (eg demographic, clinical, social) and information on exposures and potential confounders. (Line162-168， page 9)
		(b) Indicate number of participants with missing data for each variable of interest
		(c) Summarise follow-up time (eg, average and total amount)
Outcome data	15*	Report numbers of outcome events or summary measures over time. (Line 160，

For peer review only - http://bmjopen.bmj.com/site/about/guidelines.xhtml

		page 8 and line 184, page 10 and line 206, page 13)
Main results	16	(*a*) Give unadjusted estimates and, if applicable, confounder-adjusted estimates and their precision (eg, 95% confidence interval). Make clear which confounders were adjusted for and why they were included
		(*b*) Report category boundaries when continuous variables were categorized
		(*c*) If relevant, consider translating estimates of relative risk into absolute risk for a meaningful time period
Other analyses	17	Report other analyses done—eg analyses of subgroups and interactions, and sensitivity analyses
**Discussion**		
Key results	18	Summarise key results with reference to study objectives. (line329-336, page23)
Limitations	19	Discuss limitations of the study, taking into account sources of potential bias or imprecision. Discuss both direction and magnitude of any potential bias. (line370-384, page25)
Interpretation	20	Give a cautious overall interpretation of results considering objectives, limitations, multiplicity of analyses, results from similar studies, and other relevant evidence
Generalisability	21	Discuss the generalisability (external validity) of the study results
**Other information**		
Funding	22	Give the source of funding and the role of the funders for the present study and, if applicable, for the original study on which the present article is based. (line400-404, page26)

*Give information separately for exposed and unexposed groups.

Note: An Explanation and Elaboration article discusses each checklist item and gives methodological background and published examples of transparent reporting. The STROBE checklist is best used in conjunction with this article (freely available on the Web sites of PLoS Medicine at http://www.plosmedicine.org/, Annals of Internal Medicine at http://www.annals.org/, and Epidemiology at http://www.epidem.com/). Information on the STROBE Initiative is available at http://www.strobe-statement.org.

For peer review only - http://bmjopen.bmj.com/site/about/guidelines.xhtml

For peer review only

BMJ Open

Temporal trend of population structure, burden of diseases, healthcare resources and expenditure in China, 2000-2019

Journal:	*BMJ Open*
Manuscript ID	bmjopen-2022-062091.R3
Article Type:	Original research
Date Submitted by the Author:	25-Oct-2022
Complete List of Authors:	Liang, Zhenguo; Third Affiliated Hospital of Sun Yat-Sen University, Department of Rheumatology<br>WU, Dongze; Third Affiliated Hospital of Sun Yat-Sen University, Department of Rheumatology; Sichuan Provincial People's Hospital, Department of Rheumatology and Immunology<br>Guo, Cui; The Chinese University of Hong Kong; The University of Hong Kong, Department of Urban Planning and Design, Faculty of Architecture<br>Gu, Jieruo; Third Affiliated Hospital of Sun Yat-Sen University, Department of Rheumatology
**Primary Subject Heading**:	Health policy
Secondary Subject Heading:	Global health, Health policy
Keywords:	Health economics < HEALTH SERVICES ADMINISTRATION & MANAGEMENT, Health policy < HEALTH SERVICES ADMINISTRATION & MANAGEMENT, Health & safety < HEALTH SERVICES ADMINISTRATION & MANAGEMENT

SCHOLARONE™ Manuscripts

I, the Submitting Author has the right to grant and does grant on behalf of all authors of the Work (as defined in the below author licence), an exclusive licence and/or a non-exclusive licence for contributions from authors who are: i) UK Crown employees; ii) where BMJ has agreed a CC-BY licence shall apply, and/or iii) in accordance with the terms applicable for US Federal Government officers or employees acting as part of their official duties; on a worldwide, perpetual, irrevocable, royalty-free basis to BMJ Publishing Group Ltd ("BMJ") its licensees and where the relevant Journal is co-owned by BMJ to the co-owners of the Journal, to publish the Work in this journal and any other BMJ products and to exploit all rights, as set out in our licence.

The Submitting Author accepts and understands that any supply made under these terms is made by BMJ to the Submitting Author unless you are acting as an employee on behalf of your employer or a postgraduate student of an affiliated institution which is paying any applicable article publishing charge ("APC") for Open Access articles. Where the Submitting Author wishes to make the Work available on an Open Access basis (and intends to pay the relevant APC), the terms of reuse of such Open Access shall be governed by a Creative Commons licence – details of these licences and which Creative Commons licence will apply to this Work are set out in our licence referred to above.

Other than as permitted in any relevant BMJ Author's Self Archiving Policies, I confirm this Work has not been accepted for publication elsewhere, is not being considered for publication elsewhere and does not duplicate material already published. I confirm all authors consent to publication of this Work and authorise the granting of this licence.

For peer review only

Temporal trend of population structure, burden of diseases, healthcare resources and expenditure in China, 2000-2019

Authors

Zhenguo Liang, PhD[1#], Dongze Wu, PhD [1,2,3#], Cui Guo[4,5#], PhD, Jieruo Gu, PhD[1*]

Institution

1.Department of Rheumatology, The Third Affiliated Hospital of Sun Yat-sen University, Guangzhou, China

2.Department of Rheumatology and Immunology, Sichuan Provincial People's Hospital, University of Electronic Science and Technology of China, Chengdu, China.

3.Chinese Academy of Sciences Sichuan Translational Medicine Research Hospital, Chengdu 610072, China.

4.Jockey Club School of Public Health and Primary Care, The Chinese University of Hong Kong, Hong Kong SAR, China

5. Department of Urban Planning and Design, Faculty of Architecture, The University of Hong Kong, Hong Kong SAR, China

[#]Contributed equally to the manuscript.

Corresponding authors

[*]Corresponding to Prof. Jieruo Gu.

Prof. Jieguo Gu, PhD

Address: No. 600 Tianhe Road, Tianhe District, Guangzhou, Guangdong, China, 510000

E-mail: gujieruo@mail.sysu.edu.cn

Telephone: (+86) 2085253333 / Fax number: (+86) 2085253336

Running Head: Population ageing in China

For peer review only

For peer review only - http://bmjopen.bmj.com/site/about/guidelines.xhtml

24 **Keywords:** Trend, Population, Heath Care, Disease Burden, China

25 **Word Count: 3934 Reference:44 Table Count: 4 Figure Count:1**

For peer review only

For peer review only - http://bmjopen.bmj.com/site/about/guidelines.xhtml

Abstract

Objectives

To explore the evolutionary trend of population structure, disease burden, healthcare resources and expenditure in China, and to identify key domains that are most in need of intervention.

Design

A cross-sectional and longitudinal analysis

Data source

Population and healthcare data from China Statistical Yearbook (CSY), and disease burden attributable to causes and risk factors from the Global Burden of Diseases (GBD) between 2000 and 2019.

Measures and methods

We used the Joinpoint Regression Program to measure trends in population composition, population change, dependency ratio, healthcare institution, personnel, expenditure, disease burden from 2000 to 2019.

Results

Regarding the population in China between 2000 and 2019, a decreasing trend was observed among youth aged 0–14 years (AAPC: -1.17), a slow rising trend was observed among individuals aged 15–64 years (AAPC: 1.10), and a rapidly increasing trend was observed among individuals older than 65 years (AAPC: 3.67). Astonishing increasing trends in healthcare institutions (AAPC: 3.97), medical personnel (AAPC: 3.26), and healthcare expenditures (AAPC: 15.28) were also observed. Among individuals younger than 70 years, neoplasms (AAPC: 0.54) and cardiovascular diseases (AAPC: 0.67) remained among the top three causes, while tobacco (AAPC: 0.22)

For peer review only - http://bmjopen.bmj.com/site/about/guidelines.xhtml

remained a top three risk factor. However, while musculoskeletal disorders (AAPC: 1.88) were not a top three cause in 2000, they are a top three cause in 2019.

Conclusion

Comprehensive age-cause-risk factor-specific strategies are key to reconcile the tension among triangle of population ageing, disease burden and healthcare expenditure. The disease burden from cardiometabolic diseases, neoplasms and musculoskeletal disorders were identified as key domains that require intervention to reduce an increasing disease burden among individuals currently older than 70 years, as well as those approaching this age group.

Keywords: Trend, Population, Heath Care, Disease Burden, China

Strengths and limitations of this study

- This study is the first to comprehensively characterize the patterns and temporal trends in population structure and composition, health resources and expenditures, life expectancy and disease burden in China from 2000 to 2019.
- This study not only provided an overview of population, healthcare, and disease burden in China, but also inform the policymakers to pay more attention to the increasing ageing driven disease burden in older population.
- Although the data from Global Burden of Disease and China Statistical Yearbook are well refined, the quality of the data source needs to be further improved.
- The principal limitation of this study is unavailability of provincial level data in disease burden, population, and healthcare and inability to provide granular policy recommendation.

For peer review only

Introduction

Together with the world's population, people in China struggle against cancer and aging despite efforts to extend lifespan and maximize healthspan[1, 2]. Since 1950, the lifespan at birth has been extended by 26 years in China, while the total fertility rate has decreased from 5.91 to 1.43[3]. Between 2000 and 2019, the life expectancy for males has increased from 69.1 to 74.4 years, while the life expectancy for females has increased from 73.9 to 80.8 years[3]. It has been forecasted that China will become the largest economy by 2035, and that the population in China will decline by 48.0% from 2017 to 2100[4]. Moreover, in the next 20 years, the universal two-to-three-child policy may not reverse a shrinking workforce and rapid population ageing, especially in rural areas[5, 6]. In 2009, China expanded social health insurance coverage to its entire population and reformed its health care delivery system[7, 8].

Health care systems coordinate complex interrelationships among individuals who receive and provide medical financial support[9]. It would have been difficult for China to rigorously control the two pandemics that occurred in 2003 and 2019 without a healthcare system. In particular, an increasing number of modernized medical institutions and well-educated personnel have been established over the past two decades in China[10, 11]. Accelerating rural-to-urban migration and corresponding increases in urban populations have also both led to improved access to health care, although substantial health risks can accompany changes in human activities, diets, and social structures[12]. Although two GBD studies have assessed the disease burden and risk factor at provincial level in China[13, 14], the study further to evaluate healthcare professional and expenditure and to identify key domain to mitigate the aging driven disease burden.

Ageing society in China continue to exacerbate the burden borne by current family and public healthcare systems, which make it is necessary to make long-term strategic plans to respond to the

For peer review only - http://bmjopen.bmj.com/site/about/guidelines.xhtml

pressures of an ageing society at the governmental, individual, and technologic level[15]. There are a very low spatial and temporal matching degrees between the population-ageing rate and the number of medical resources per thousand residents in China, and the geographical pattern of the temporal matching between them exhibited a feature of north-south differentiation [16]. China does not have a typical disease characteristic of an aging society comparing the characteristics of the aging population in China with those in the world[17]. Namely, China faces the dual threat of non-communicable diseases and communicable diseases, and the former account for most of the age-related disease burden. Although there are great number of studies centering on trend of population, burden of disease, healthcare in China, but they failed to dissect the implication of a central tension among 3 mutually conflicting forces: intractable disease burden, population ageing, and upsurging healthcare expenditure. The study tries to find a solution to reconcile the three contradictory forces, achieving longer healthy lifespan in the ageing society at high-cost performance of health expenditure. Possible driving forces of disease burden among ageing populations were also examined to provide a basis for targeted interventions, such as early disease prevention and health management among older populations.

Methods

Data sources

The National Bureau of Statistics (NBS) of China annually publishes the China Statistical Yearbook (CSY)[18]. The Department of Population and Employment Statistics of the NBS conducts statistical population surveys as follows. A national population census is conducted in years ending with 0; a national 1 percent population sample survey is conducted in years ending with 5; and sample surveys on population changes are conducted in the remaining years. The latter

For peer review only - http://bmjopen.bmj.com/site/about/guidelines.xhtml

covers about 1 per thousand of the total population of China. The sample surveys on population change take the entire nation of China as the population, while each province, autonomous region, or municipality represents a sub-population. A stratified multi-stage systematic probability-proportional-to-size (PPS) cluster sampling scheme is used. Data regarding population, healthcare institutions, personnel, beds, and health expenditures are described in detail in the Supplemental Appendix (including a glossary and definitions).

The Global Burden of Disease（GBD）2019 study assessed life expectancy, disease burden, risk factor, etiology, and impairment[3, 19, 20]. All-cause incidence, prevalence, death, years lived with disability (YLD), years of life lost (YLL), disability-adjusted life years (DALYs), and attributable risk factors were assessed between 1990 and 2019. The study identified twenty-two level 2 leading causes and twenty level 2 leading risk factors. Both causes and risk factors were assessed according to number, rate, and age-standardized rate of DALYs. The estimation methods and data for burden of disease published in GBD 2019 were used to assess disease burden in China between 2000 and 2019 for the present study.

Aggregated data of population and healthcare from CSY and aggregated data of life expectancy, disease burden attributable to 22 causes or 20 risk factors from GBD between 2000 and 2019 were used to quantify temporal trend of population structure, burden of diseases, healthcare resources and expenditure in China.

Outcomes of measurements

The primary outcomes are temporal trend in population (population composition, population change, dependency ratio), healthcare (number of healthcare institution, number of health personnel, healthcare expenditure), life expectancy (healthy and total), disease burden attributable to 22 causes or 20 risk factors (disability adjusted life years, DALYs) from 2000 to 2019. The

For peer review only - http://bmjopen.bmj.com/site/about/guidelines.xhtml

secondary outcomes are i) the main drivers of disease burden from 22 causes or 20 risk factors in individuals younger and older than 70, ii) temporal trend in population, healthcare, and disease burden attributable to 22 causes or 20 risk factors from 2000 to 2009, and from 2010 to 2019. Driver refers the cause or risk factor that have the greatest impact on the total disease burden attributable to 22 causes or 20 risk factors from 2000 to 2019.

Age groups of population and disease burden

Two set of age groups were used to specifically assess the trend of population composition and disease burden. Population composition (0-14, 15-64, 65+) and dependency ratio (gross, children, old) were used to reflect the trend of working population and population ageing as age 0-14, 15-64 and 65+ group were younger population, working population, post-working population, respectively. The analysis of disease burden in population younger and older than 70 years aimed to identify the cause or risk factor (driver) which has the greatest impact on the trend of disease burden attributable to 22 causes and 20 risk factors from 2000 to 2019. The cut-point of age 70 was chosen because women in 164 and men in 165 of 186 countries and territories had a higher probability of dying before 70 years of age from a non-communicable disease (NCD) than from communicable, maternal, perinatal, and nutritional conditions combined according to NCD Countdown 2030[19, 21].

Statistical analysis

The population composition, change, dependency ratio, healthcare institution, personnel, and expenditures in terms of number, disease burden and risk factors in terms of number and rate, were assessed by using a Joinpoint regression model. The disease burden and risk factor in individuals younger and older than 70 years in term of number and rate were also assessed to identify the driving causes and risk factors. Average annual percent change (AAPC) and associated 95%

For peer review only - http://bmjopen.bmj.com/site/about/guidelines.xhtml

confidence intervals (CIs) were calculated for the study period. Temporal increasing and decreasing trends were defined according to the statistical significance of the AAPC compared to zero. AAPC values with 95% CIs overlapping with zero were considered stable. Two segmented line regression with joinpoint of 2000, 2009, and 2019 were calculated to show the temporal trend from 2000 to 2009, from 2010 to 2019. All statistical analyses were performed by using the Joinpoint Regression Program (version 4.8.0.1, Statistical Methodology and Applications Branch, Surveillance Research Program, National Cancer Institute, Bethesda, MD, USA). P-values less than 0.05 were considered statistically significant at a two-tailed level.

Patient and public involvement statement

Patients and the public were not involved in any way in this research.

Results

Population trends between 2000 and 2019

Initially, temporal trends of the population structure in China were examined based on CSY data in terms of gender and age composition, birth rate, and dependency ratio. From 2000 through 2019, an increasing trend in total population (AAPC: 0.52, 95% CI: 0.51 to 0.54), male population (AAPC: 0.47, 95% CI: 0.44 to 0.51), and female population (AAPC: 0.59, 95% CI: 0.56 to 0.62) were observed. As expected, a striking contrary trend in urban population (AAPC: 3.28, 95%CI: 3.18 to 3.38) and rural population (AAPC: -1.98, 95%CI: -2.02 to -1.95) were observed. We further identified that an increasing trend in urban population was attenuated, while a decreasing trend in rural population was aggravated, during 2010–2019 compared to 2000–2009 (Figure 1A, Table 1).

A clear decreasing trend in the younger population (ages 0–14 years) was observed in 2000–2019 (AAPC: -1.17, 95%CI: -1.78 to -0.55), especially in 2000–2009. An increasing trend in the working population (ages 15–64 years) was observed in 2000–2009 (AAPC: 1.10, 95%CI: 1.03 to

For peer review only - http://bmjopen.bmj.com/site/about/guidelines.xhtml

1.18), and then it gradually disappeared over 2010–2019. In contrast, an increasing trend in the post-working population (65+ years) that was observed in 2000–2019 (AAPC: 3.67, 95%CI: 3.51 to 3.82) gradually intensified from 2000–2009 to 2010–2019 (Figure 1B, Table 1).

From 2000 to 2019, the birth rate (AAPC: -1.53, 95%CI: -2.31 to -0.75) and natural growth rate (AAPC: -4.20, 95%CI: -5.52 to -2.87) continued to decrease. Conversely, from 2000 to 2009, the death rate continued to increase (AAPC: 1.10, 95%CI: 0.75 to 1.46), yet from 2010–2019 it remained stable (AAPC: 0.03, 95%CI: -0.06 to 0.13) (Figure 1C, Table 1). The past two decades also exhibited a reverse trend in gross dependency ratio from 2010–2019, a muted decreasing trend in children dependency ratio from 2000–2009 to 2010–2019, and a strengthened increasing trend in old dependency ratio from 2000–2009 to 2010–2019 (Figure 1D, Table 1).

For peer review only

For peer review only - http://bmjopen.bmj.com/site/about/guidelines.xhtml

197 **Table 1.** The average annual percent change in population composition, change, and dependency ratio between 2000 and 2019

Subcategory	Number			AAPC (95% CI)		
	2000	2009	2019	2000–2019	2000–2009	2010–2019
**Population composition by gender and area (10000 persons)**						
Total Population	126,743	133,450	140,005	0.52 (0.51 to 0.54)*	0.57 (0.56 to 0.58)*	0.48 (0.45 to 0.51)*
Male Population	65,437	68,647	71,527	0.47 (0.44 to 0.51)*	0.53 (0.50 to 0.57)*	0.43 (0.37 to 0.49)*
Female Population	61,306	64,803	68,478	0.59 (0.56 to 0.62)*	0.62 (0.56 to 0.67)*	0.56 (0.55 to 0.57)*
Urban Population	45,906	64,512	84,843	3.28 (3.18 to 3.38)*	3.86 (3.75 to 3.98)*	2.68 (2.51 to 2.84)*
Rural Population	80,837	68,938	55,162	-1.98 (-2.02 to -1.95)*	-1.79 (-1.86 to -1.73)*	-2.15 (-2.18 to -2.12)*
**Population composition by age group (10000 persons)**						
Population Aged 0–14 y	29,012	24,659	23,492	-1.17 (-1.78 to -0.55)*	-2.24 (-2.76 to -1.71)*	0.31 (-0.23 to 0.85)
Population Aged 15–64 y	88,910	97,484	98,910	0.59 (0.52 to 0.66)*	1.10 (1.03 to 1.18)*	0.02 (-0.10 to 0.14)
Population Aged 65+ y	8,821	11,307	17,603	3.67 (3.51 to 3.82)*	2.81 (2.66 to 2.96)*	4.51 (4.25 to 4.76)*
**Population change (%)**						
Birth Rate	14.03	11.95	10.48	-1.53 (-2.31 to -0.75)*	-1.63 (-2.32 to -0.93)*	-1.57 (-3.03 to -0.09)*
Death Rate	6.45	7.08	7.14	0.54 (0.37 to 0.71)*	1.10 (0.75 to 1.46)*	0.03 (-0.06 to 0.13)
Natural Growth Rate	7.58	4.87	3.34	-4.20 (-5.52 to -2.87)*	-4.64 (-5.69 to -3.57)*	-4.40 (-6.93 to -1.80)*
**Dependency ratio (%)**						
Gross Dependency Ratio	42.6	36.9	41.5	-0.25 (-0.54 to 0.03)	-1.97 (-2.26 to -1.68)*	1.70 (1.21 to 2.19)*
Children Dependency Ratio	32.6	25.3	23.8	-1.76 (-2.54 to -0.96)*	-3.33 (-4.00 to -2.66)*	0.33 (-0.37 to 1.03)
Old Dependency Ratio	9.9	11.6	17.8	3.12 (2.93 to 3.31)*	1.76 (1.45 to 2.08)*	4.49 (4.27 to 4.70)*

198 AAPC: average annual percent change; CI, confidence interval. *AAPC significantly differs from zero at a two-tailed level of 0.05.

For peer review only - http://bmjopen.bmj.com/site/about/guidelines.xhtml

Health resources and expenditure between 2000 and 2019

Temporal trends in health sources, including institutions, personnel, and expenditures, were examined based on CSY data. A consistent increasing trend in number of hospitals (AAPC: 3.97, 95%CI: 3.69 to 4.24), including general hospitals, hospitals that specialize in traditional Chinese medicine, and specialized hospitals, was observed. The number of medical personnel (AAPC: 3.26, 95%CI: 2.76 to 3.77), including medical technical personnel, licensed assistant doctors, licensed doctors, and registered nurses, also continued to increase in 2000–2019. Moreover, the rate of increase of number of medical personnel was higher in 2010–2019 than in 2000–2009. The number of beds in health care institutions (AAPC: 5.54, 95%CI: 5.20 to 5.88), including hospitals, township health service centers, specialized public health institutions, and women and children care agencies, also increased in 2000–2019. Like medical personnel, the rate increase for beds in health care institutions was greater in 2010–2019 than in 2000–2009 (Table 2).

Total healthcare expenditure increased from ¥458.66 billion (US$ 55.40 billion) in 2000 to ¥6584.14 billion (US$ 954.68 billion) in 2019, with an AAPC of 15.28 (95%CI: 14.64 to 15.91). The per capita health expenditure increased from ¥361.88 in 2000 to ¥4702.79 in 2019, with an AAPC of 14.67 (95%CI: 14.04 to 15.30). Striking increasing trends in government health expenditure (AAPC: 18.95, 95%CI: 16.72 to 21.22), social health expenditure (AAPC: 19.03, 95%CI: 17.56 to 20.52), and out-of-pocket health expenditure (AAPC: 10.62, 95%CI: 9.24 to 12.02) were also observed in 2000–2019 (Table 2).

For peer review only - http://bmjopen.bmj.com/site/about/guidelines.xhtml

Table 2. The average annual percent change in healthcare institution, personnel, and expenditures between 2000 and 2019

Subcategory	Number			AAPC (95% CI)		
	2000	2009	2019	2000—2019	2000—2009	2010—2019
**Health care institution (unit)**						
Number of Hospitals	16,318	20,291	34,354	3.97 (3.69 to 4.24)*	2.41 (2.03 to 2.80)*	5.72 (5.25 to 6.19)*
Number of General Hospitals	11,872	13,364	19,963	2.84 (2.56 to 3.13)*	1.37 (0.97 to 1.77)*	4.51 (4.03 to 4.99)*
Number of Hospitals Specialized in Traditional Chinese Medicine	2,453	2,728	4,221	2.92 (2.50 to 3.34)*	1.35 (1.20 to 1.50)*	4.69 (3.82 to 5.58)*
Number of Specialized Hospitals	1,543	3,716	8,531	9.65 (9.02 to 10.28)*	10.98 (9.69 to 12.28)*	8.46 (8.25 to 8.68)*
Number of Health Care Institutions at Grass-root Level	1,000,169	882,153	954,390	-0.45 (-0.82 to -0.08)*	-1.88 (-2.60 to -1.15)*	0.85 (0.67 to 1.04)*
Number of Township Health Service Centers	49,229	38,475	36,112	-1.63 (-1.83 to -1.44)*	-2.79 (-3.10 to -2.47)*	-0.47 (-0.66 to -0.28)*
Number of Village Clinics	709,458	632,770	616,094	-1.03 (-1.40 to -0.65)*	-1.88 (-2.53 to -1.23)*	-0.51 (-0.86 to -0.17)*
Number of Outpatient Department	240,934	182,448	266,659	0.42 (-0.18 to 1.03)	-2.73 (-3.45 to -2.01)*	4.05 (3.03 to 5.07)*
Number of Specialized Public Health Institutions	11,386	11,665	15,958	1.63 (-0.23 to 3.54)	0.12 (-0.68 to 0.93)	3.35 (-0.52 to 7.36)
Number of Center for Disease Control and Prevention	3,741	3,536	3,403	-0.40 (-0.50 to -0.29)*	-0.40 (-0.50 to -0.29)*	-0.40 (-0.50 to -0.29)*
Number of Specialized Disease Prevention & Treatment Institution	1,839	1,291	1,128	-2.47 (-2.97 to -1.98)*	-3.61 (-4.46 to -2.76)*	-1.49 (-2.05 to -0.93)*
Number of Women and Children Care Agencies	3,163	3,020	3,071	-0.11 (-0.29 to 0.08)	-0.45 (-0.80 to -0.10)*	0.20 (0.09 to 0.32)*
**Medical Personnel (10000 persons)**						
Number of Medical Personnel	691.04	778.14	1292.83	3.26 (2.76 to 3.77)*	1.15 (0.16 to 2.14)*	5.16 (4.87 to 5.45)*
Number of Medical Technical Personnel	449.08	553.51	1015.40	4.34 (3.99 to 4.68)*	2.30 (1.61 to 3.01)*	6.20 (6.07 to 6.34)*
Number of Licensed (Assistant) Doctors	207.58	232.92	386.69	3.12 (2.64 to 3.59)*	0.92 (0.05 to 1.81)*	5.36 (4.98 to 5.74)*
Number of Licensed Doctors	160.33	190.54	321.05	3.53 (3.12 to 3.95)*	1.50 (0.73 to 2.27)*	5.62 (5.29 to 5.95)*
Number of Registered Nurse	126.68	185.48	444.50	6.79 (6.20 to 7.38)*	4.37 (3.26 to 5.49)*	8.92 (8.49 to 9.34)*
Number of Pharmacist	41.44	34.19	48.34	0.76 (0.23 to 1.29)*	-2.28 (3.31 to -1.23)*	3.57 (3.27 to 3.88)*
Number of Village Doctors and Assistants	131.94	105.10	84.23	-2.49 (-4.28 to -0.66)*	-3.44 (-6.25 to -0.55)*	-2.16 (-4.10 to -0.17)*
Number of Other Technical Personnel	15.75	27.50	50.39	6.30 (6.07 to 6.53)*	6.30 (6.07 to 6.53)*	6.30 (6.07 to 6.53)*
Number of Administrative Personnel	42.68	36.27	54.38	1.25 (0.42 to 2.09)*	-1.74 (-3.27 to -0.18)*	4.01 (3.49 to 4.54)*
Number of Logistics Technical Workers	51.59	55.77	88.43	2.95 (2.13 to 3.78)*	0.84 (-0.26 to 1.94)	4.71 (3.57 to 5.86)*
**Beds in Health Care Institutions**						
Number of Beds in Health Care Institutions (10000 units)	317.70	441.66	880.70	5.54 (5.20 to 5.88)*	3.60 (3.01 to 4.20)*	7.11 (6.82 to 7.41)*
Number of Beds in Hospitals (10000 beds)	216.67	312.08	686.65	6.35 (5.96 to 6.74)*	4.17 (3.55 to 4.81)*	8.16 (7.74 to 8.59)*
Number of Beds in Community Health Service Centers (10000 units)	76.65	109.98	163.11	3.96 (2.90 to 5.04)*	3.73 (1.68 to 5.83)*	3.28 (2.75 to 3.82)*
Number of Beds in Township Health Service Centers (10000 beds)	73.48	93.34	136.99	3.29 (2.18 to 4.41)*	2.38 (0.26 to 4.54)*	3.48 (2.93 to 4.04)*

For peer review only

For peer review only - http://bmjopen.bmj.com/site/about/guidelines.xhtml

Number of Beds in Specialized Public Health Institutions (10000 units)	11.86	15.40	28.50	4.80 (4.31 to 5.28)*	2.97 (2.52 to 3.42)*	6.24 (5.55 to 6.93)*
Number of Beds in Women and Children Care Agencies (10000 units)	7.12	12.61	24.32	6.79 (6.41 to 7.18)*	6.65 (6.15 to 7.15)*	6.74 (6.25 to 7.24)*
Number of Beds in Specialized Disease Prevention & Treatment Institution (10000 beds)	2.84	2.71	4.11	2.29 (0.21 to 4.41)*	0.19 (-3.08 to 3.57)	3.75 (1.61 to 5.95)*
**Health Expenditure (100 million yuan)**						
Total Health Expenditure	4586.63	17541.92	65841.39	15.28 (14.64 to 15.91)*	15.99 (15.09 to 16.90)*	14.04 (13.44 to 14.63)*
Government Health Expenditure	709.52	4816.26	18016.95	18.95 (16.72 to 21.22)*	24.39 (20.91 to 27.97)*	13.52 (11.30 to 15.79)*
Social Health Expenditure	1171.94	6154.49	29150.57	19.03 (17.56 to 20.52)*	21.13 (18.16 to 24.18)*	17.17 (16.18 to 18.16)*
Out-of-pocket Health Expenditure	2705.17	6571.16	18673.87	10.62 (9.24 to 12.02)*	9.82 (9.21 to 10.44)*	10.96 (8.48 to 13.48)*
Per Capita Health Expenditure (yuan)	361.88	1314.26	4702.79	14.67 (14.04 to 15.30)*	15.34 (14.45 to 16.24)*	13.47 (12.89 to 14.06)*

AAPC: average annual percent change; CI, confidence interval. *AAPC significantly differs from zero at a two-tailed level of 0.05.

For peer review only

For peer review only - http://bmjopen.bmj.com/site/about/guidelines.xhtml

Population health, disease burden and risk factors between 2000 and 2019

According to data from GBD 2019, life expectancy at birth and at 60–64 years were estimated to increase (AAPC: 0.44, 95%CI: 0.43 to 0.46; AAPC: 0.76, 95%CI: 0.60 to 0.91, respectively). In addition, the percentage of healthy life expectancy within total life expectancy at birth and at 60–64 years decreased from 88.90 to 88.32, and from 77.12 to 76.36, respectively (sFigure 1, sTable 1–3). Overall, the number of all-cause DALYs was stable during 2000–2019 (AAPC: 0.08, 95%CI -0.06 to 0.22). However, it decreased between 2000 and 2009 (AAPC: -0.44, 95%CI: -0.58 to -0.31), and it increased between 2010 and 2019 (AAPC: 0.60, 95% CI: 0.39 to 0.82) (sTable 4).

In 2000, the top three causes of DALYs were cardiovascular diseases, neoplasms, and chronic respiratory diseases. In 2019, they were cardiovascular diseases, neoplasms, and musculoskeletal disorders. From 2000–2019, the top three drivers of increasing number of DALYs were HIV/AIDS and sexually transmitted infections (AAPC: 3.47, 95%CI: 2.72 to 4.22), diabetes and kidney diseases (AAPC: 2.30, 95%CI: 2.09 to 2.51), and sense organ diseases (AAPC: 2.14, 95%CI: 1.98 to 2.30). The top two drivers of increasing age-standardized rate of DALYs were HIV/AIDS and sexually transmitted infections (AAPC: 2.02, 95%CI: 1.50 to 2.53) and musculoskeletal disorders (AAPC: 0.16, 95%CI: 0.10 to 0.23) (sTable 5).

In 2000, the top three risk factors were tobacco, air pollution, and high systolic blood pressure. In 2019, they were tobacco, high systolic blood pressure, and dietary risks. Between 2000 and 2019, the top three leading risk factors with the largest increasing number of DALYs were high body-mass index (AAPC: 4.02, 95%CI: 3.74 to 4.29), low physical activity (AAPC: 3.54, 95%CI: 2.70 to 4.38), and low bone mineral density (AAPC:

For peer review only

3.39, 95%CI: 3.12 to 3.67). The top three leading factors with the largest increase in age-standardized rate of DALYs were unsafe sex (AAPC: 1.47, 95% CI: 0.88 to 2.06), high body-mass index (AAPC: 1.28, 95%CI: 0.95 to 1.60), and low bone mineral density (AAPC: 0.40, 95%CI: 0.27 to 0.54) (sTable 6).

Trends in disease burden between younger individuals and those older than 70 years

Finally, trends in causes and risk factors for the population with a cut-off point of 70 years were examined for 2000–2019. Among individuals younger than 70 years, musculoskeletal disorders replaced other non-communicable diseases as one of the top three causes from 2000 to 2019. In contrast, the top three causes remained the same among individuals older than 70 years, and included cardiovascular diseases, neoplasms, and chronic respiratory disease (Table 3). Among the population younger than 70 years, the causes with the largest increment in number of DALYs were HIV/AIDS and sexually transmitted infections (AAPC: 3.33, 95%CI: 2.58 to 4.08), musculoskeletal disorders (AAPC: 1.88, 95%CI: 1.84 to 1.92), and sense organ diseases (AAPC: 1.71, 95%CI: 1.43 to 2.00). In the same population, the largest increments in rate of DALYs were HIV/AIDS and sexually transmitted infections (AAPC: 3.42, 95%CI: 2.76 to 4.09), musculoskeletal disorders (AAPC: 2.04, 95%CI: 2.00 to 2.08), and diabetes and kidney diseases (AAPC: 1.88, 95%CI: 1.58 to 2.18). In comparison, the causes among individuals older than 70 years that had the largest increment in both number and rate of DALYs were maternal and neonatal disorders (AAPC: 10.64, 95%CI: 10.47 to 10.82; AAPC: 6.84, 95%CI: 6.69 to 6.98, respectively), HIV/AIDS and sexually transmitted infections (AAPC: 8.17, 95%CI: 6.93 to 9.43;AAPC:4.39, 95%CI: 3.26 to 3.53, respectively), and unintentional injuries (AAPC:4.66, 95%CI: 3.90 to 5.43; AAPC: 1.01, 95%CI: 0.46 to 1.56, respectively) (Table

For peer review only - http://bmjopen.bmj.com/site/about/guidelines.xhtml

3).

Among the population younger than 70 years, dietary risks (AAPC: 1.02, 95%CI: 0.85 to 1.19) and high systolic blood pressure (AAPC: 1.55, 95%CI: 1.29 to 1.81) replaced air pollution and child and maternal malnutrition to enter the top three risk factors from 2000 to 2019. In contrast, the top three risk factors remained the same between 2000 and 2019 for the population older than 70 years, and they include: high systolic blood pressure, tobacco, and air pollution (Table 3). Regarding risk factors among the population younger than 70 years, the largest increments in number and rate of DALYs were high body-mass index (AAPC: 3.70, 95%CI: 3.59 to 3.82; AAPC: 3.88, 95%CI: 3.69 to 4.07, respectively), unsafe sex (AAPC: 3.11, 95%CI: 2.80 to 3.42; AAPC: 3.28, 95%CI: 2.96 to 3.60, respectively), and low physical activity (AAPC: 2.75, 95%CI: 2.34 to 3.15; AAPC: 2.91, 95%CI: 2.46 to 3.36, respectively). In contrast, the risk factors with the largest increment in number of DALYs among those older than 70 years were high body-mass index (AAPC: 5.22, 95%CI: 4.79 to 5.65), low bone mineral density (AAPC: 5.22, 95%CI: 4.98 to 5.46), and high LDL cholesterol (AAPC: 5.02, 95%CI: 4.62 to 5.41). Furthermore, the same risk factors with different rankings were observed in terms of rate of DALYs for the same population: low bone mineral density (AAPC: 1.62, 95%CI: 1.38 to 1.86), high body-mass index (AAPC: 1.50, 95%CI: 1.01 to 2.00), and high LDL cholesterol (AAPC: 1.44, 95%CI: 1.04 to 1.84), respectively (Table 4).

For peer review only - http://bmjopen.bmj.com/site/about/guidelines.xhtml

Table 3. The average annual percent change of 22 causes in people younger and older than 70 years of age presented in disability adjusted life years

Causes	Age < 70 years			Age ≥ 70 years		
	2000	2019	AAPC (95% CI)	2000	2019	AAPC (95% CI)
Number						
Neglected tropical diseases and malaria	1,566,614 (1,022,581 to 2,343,199)	977,312 (586,629 to 1,582,219)	-2.42 (-2.73 to -2.10)*	137,451 (93,607 to 195,272)	160,494 (103,985 to 236,535)	0.83 (0.72 to 0.94)*
Nutritional deficiencies	3,912,001 (2,872,239 to 5,269,234)	2,247,485 (1,515,113 to 3,202,132)	-2.91 (-3.07 to -2.76)*	271,587 (210,115 to 352,577)	359,440 (285,475 to 450,281)	1.51 (1.21 to 1.81)*
Neoplasms	43,633,030 (40,123,940 to 47,258,486)	48,503,754 (40,915,702 to 56,689,779)	0.54 (0.36 to 0.73)*	10,782,364 (10,021,201 to 11,549,415)	19,016,076 (16,369,289 to 21,712,270)	3.00 (2.66 to 3.34)*
Cardiovascular diseases	40,746,813 (37,734,622 to 44,374,198)	45,823,157 (39,092,553 to 53,248,142)	0.67 (0.52 to 0.82)*	26,837,679 (24,915,174 to 29,373,891)	46,109,965 (40,669,589 to 51,698,469)	2.77 (2.26 to 3.29)*
Chronic respiratory diseases	13,237,789 (11,103,026 to 14,475,351)	8,969,697 (7,748,010 to 10,534,163)	-2.04 (-2.22 to -1.86)*	15,416,177 (12,665,862 to 16,567,447)	13,550,852 (11,816,464 to 16,036,186)	-0.70 (-1.15 to -0.24)*
Digestive diseases	9,775,769 (8,808,397 to 10,835,090)	7,710,658 (6,445,828 to 8,964,870)	-1.21 (-1.30 to -1.13)*	1,923,302 (1,762,799 to 2,106,969)	2,307,223 (2,029,697 to 2,600,797)	0.92 (0.72 to 1.13)*
Neurological disorders	9,677,839 (4,755,522 to 16,786,166)	11,207,269 (5,201,198 to 19,881,074)	0.78 (0.70 to 0.86)*	2,828,463 (1,694,636 to 5,326,904)	6,174,136 (3,629,888 to 11,661,806)	4.20 (4.04 to 4.35)*
Mental disorders	17,241,666 (12,746,572 to 22,736,338)	18,539,346 (13,738,299 to 24,152,654)	0.37 (0.29 to 0.46)*	868,363 (645,857 to 1,119,766)	1,754,481 (1,291,689 to 2,254,704)	3.77 (3.70 to 3.83)*
Musculoskeletal disorders	17,725,280 (12,654,675 to 23,783,378)	25,286,535 (18,045,649 to 33,942,087)	1.88 (1.84 to 1.92)*	2,364,272 (1,679,407 to 3,221,901)	4,647,389 (3,292,799 to 6,332,328)	3.60 (3.53 to 3.66)*
Other non-communicable diseases	23,430,651 (19,446,807 to 28,355,357)	16,912,189 (12,820,141 to 22,584,969)	-1.67 (-1.77 to -1.58)*	1,142,340 (831,796 to 1,540,224)	2,213,884 (1,599,002 to 2,992,458)	3.43 (2.88 to 3.98)*
Skin and subcutaneous diseases	7,427,502 (4,833,763 to 10,987,503)	7,564,844 (4,920,805 to 11,324,882)	0.09 (0.07 to 0.12)*	381,523 (263,385 to 542,001)	699,858 (475,558 to 1,030,747)	3.21 (3.14 to 3.28)*
Sense organ diseases	7,881,599 (5,268,499 to 11,424,474)	10,796,982 (7,099,138 to 15,924,830)	1.71 (1.43 to 2.00)*	2,859,183 (2,030,582 to 3,892,635)	5,184,684 (3,650,080 to 7,090,135)	3.18 (3.02 to 3.34)*
Transport injuries	16,117,112 (14,766,378 to 18,356,266)	12,597,412 (10,747,152 to 14,456,584)	-1.31 (-1.64 to -0.98)*	514,733 (454,228 to 601,574)	1,148,043 (946,296 to 1,360,515)	4.24 (3.92 to 4.57)*
Unintentional injuries	19,929,305 (18,307,020 to 21,760,124)	13,173,922 (10,853,663 to 15,202,200)	-2.15 (-2.65 to -1.65)*	1,184,747 (1,024,676 to 1,371,585)	2,829,540 (2,151,923 to 3,469,812)	4.66 (3.90 to 5.43)*
Self-harm and interpersonal violence	10,574,276 (9,275,669 to 11,544,266)	5,232,715 (4,479,867 to 6,200,044)	-3.64 (-3.94 to -3.34)*	597,000 (504,957 to 649,633)	637,335 (544,995 to 738,168)	0.37 (-0.73 to 1.50)
HIV/AIDS and sexually transmitted infections	904,578 (711,182 to 1,201,186)	1,677,944 (1,372,709 to 2,020,540)	3.33 (2.58 to 4.08)*	16,867 (13,900 to 22,250)	74,476 (55,158 to 94,754)	8.17 (6.93 to 9.43)*
Respiratory infections and tuberculosis	19,691,768 (18,048,674 to 21,296,455)	4,601,349 (3,952,381 to 5,376,466)	-7.32 (-7.79 to -6.85)*	2,246,454 (2,045,162 to 2,412,569)	2,046,523 (1,783,057 to 2,379,652)	-0.54 (-0.96 to -0.13)*
Enteric infections	3,259,938 (2,859,853 to 3,751,832)	1,179,711 (874,430 to 1,534,794)	-5.11 (-5.55 to -4.66)*	147,328 (108,525 to 198,192)	228,387 (164,251 to 306,599)	2.47 (2.25 to 2.69)*
Other infectious diseases	4,467,414 (3,610,475 to 6,110,768)	1,143,025 (971,599 to 1,357,011)	-6.85 (-7.29 to -6.42)*	114,576 (103,794 to 126,392)	101,074 (86,237 to 116,157)	-0.59 (-0.98 to -0.21)*
Maternal and neonatal disorders	18,100,148 (16,534,223 to 19,650,436)	7,080,589 (6,220,729 to 8,094,637)	-4.86 (-5.45 to -4.27)*	10,442 (7,450 to 14,363)	70,983 (51,665 to 96,401)	10.64 (10.47 to 10.82)*
Substance use disorders	6,144,467 (4,909,131 to 7,567,428)	5,459,715 (4,162,774 to 6,975,972)	-0.55 (-0.97 to -0.13)*	147,447 (122,722 to 177,177)	295,943 (226,537 to 375,653)	3.80 (3.23 to 4.37)*
Diabetes and kidney diseases	8,018,292 (6,937,163 to 9,207,068)	10,979,432 (9,048,531 to 13,121,983)	1.70 (1.42 to 1.98)*	2,418,531 (2,116,442 to 2,752,813)	4,929,741 (4,274,671 to 5,652,658)	3.78 (3.60 to 3.96)*
Rate						
Neglected tropical diseases and malaria	33 (21 to 49)	21 (13 to 34)	-2.26 (-2.56 to -1.96)*	250 (170 to 355)	149 (96 to 219)	-2.70 (-2.80 to -2.59)*
Nutritional deficiencies	82 (60 to 110)	48 (33 to 69)	-2.77 (-2.96 to -2.58)*	494 (383 to 642)	333 (264 to 417)	-2.03 (-2.30 to -1.76)*
Neoplasms	911 (838 to 987)	1,045 (881 to 1,221)	0.68 (0.59 to 0.77)*	19,629 (18,243 to 21,025)	17,613 (15,162 to 20,110)	-0.56 (-0.93 to -0.19)*
Cardiovascular diseases	851 (788 to 927)	987 (842 to 1,147)	0.84 (0.68 to 1.00)*	48,857 (45,357 to 53,474)	42,708 (37,669 to 47,885)	-0.69 (-0.82 to -0.57)*
Chronic respiratory diseases	276 (232 to 302)	193 (167 to 227)	-1.86 (-2.02 to -1.69)*	28,064 (23,058 to 30,160)	12,551 (10,945 to 14,853)	-4.13 (-4.60 to -3.66)*

For peer review only - http://bmjopen.bmj.com/site/about/guidelines.xhtml

Digestive diseases	204 (184 to 226)	166 (139 to 193)	-1.06 (-1.15 to -0.96)*	3,501 (3,209 to 3,836)	2,137 (1,880 to 2,409)	-2.55 (-2.66 to -2.45)*
Neurological disorders	202 (99 to 351)	241 (112 to 428)	0.94 (0.82 to 1.06)*	5,149 (3,085 to 9,697)	5,719 (3,362 to 10,801)	0.59 (0.29 to 0.90)*
Mental disorders	360 (266 to 475)	399 (296 to 520)	0.55 (0.51 to 0.59)*	1,581 (1,176 to 2,038)	1,625 (1,196 to 2,088)	0.15 (0.12 to 0.18)*
Musculoskeletal disorders	370 (264 to 497)	545 (389 to 731)	2.04 (2.00 to 2.08)*	4,304 (3,057 to 5,865)	4,305 (3,050 to 5,865)	-0.04 (-0.15 to 0.08)
Other non-communicable diseases	489 (406 to 592)	364 (276 to 486)	-1.54 (-1.66 to -1.42)*	2,080 (1,514 to 2,804)	2,051 (1,481 to 2,772)	-0.16 (-0.60 to 0.29)
Skin and subcutaneous diseases	155 (101 to 229)	163 (106 to 244)	0.26 (0.23 to 0.29)*	695 (479 to 987)	648 (440 to 955)	-0.38 (-0.39 to -0.36)*
Sense organ diseases	165 (110 to 239)	233 (153 to 343)	1.86 (1.70 to 2.02)*	5,205 (3,697 to 7,086)	4,802 (3,381 to 6,567)	-0.43 (-0.63 to -0.23)*
Transport injuries	337 (308 to 383)	271 (231 to 311)	-1.15 (-1.49 to -0.81)*	937 (827 to 1,095)	1,063 (876 to 1,260)	0.64 (0.33 to 0.96)*
Unintentional injuries	416 (382 to 454)	284 (234 to 327)	-1.93 (-2.44 to -1.42)*	2,157 (1,865 to 2,497)	2,621 (1,993 to 3,214)	1.01 (0.46 to 1.56)*
Self-harm and interpersonal violence	221 (194 to 241)	113 (96 to 134)	-3.49 (-3.80 to -3.18)*	1,087 (919 to 1,183)	590 (505 to 684)	-3.10 (-4.05 to -2.14)*
HIV/AIDS and sexually transmitted infections	19 (15 to 25)	36 (30 to 44)	3.42 (2.76 to 4.09)*	31 (25 to 41)	69 (51 to 88)	4.39 (3.26 to 3.53)*
Respiratory infections and tuberculosis	411 (377 to 445)	99 (85 to 116)	-7.17 (-7.61 to -6.71)*	4,090 (3,723 to 4,392)	1,896 (1,652 to 2,204)	-4.08 (-4.44 to -3.71)*
Enteric infections	68 (60 to 78)	25 (19 to 33)	-4.91 (-5.76 to -4.05)*	268 (198 to 361)	212 (152 to 284)	-1.20 (-1.45 to -0.96)*
Other infectious diseases	93 (75 to 128)	25 (21 to 29)	-6.70 (-7.14 to -6.27)*	209 (189 to 230)	94 (80 to 108)	-4.06 (-4.48 to -3.64)*
Maternal and neonatal disorders	378 (345 to 410)	153 (134 to 174)	-4.71 (-5.29 to -4.14)*	19 (14 to 26)	66 (48 to 89)	6.84 (6.69 to 6.98)*
Substance use disorders	128 (103 to 158)	118 (90 to 150)	-0.38 (-0.85 to 0.09)*	268 (223 to 323)	274 (210 to 348)	0.12 (-0.31 to 0.55)
Diabetes and kidney diseases	167 (145 to 192)	236 (195 to 283)	1.88 (1.58 to 2.18)*	4,403 (3,853 to 5,011)	4,566 (3,959 to 5,236)	0.21 (-0.05 to 0.48)

AAPC: average annual percent change; DALYs, disability adjusted life years; UI, uncertainty interval, CI, confidence interval.
*AAPC significantly differs from zero at a two-tailed level of 0.05.

For peer review only - http://bmjopen.bmj.com/site/about/guidelines.xhtml

For peer review only

Table 4. The average annual percent change of twenty risk factors in people younger and older than 70 years of age

Risk factors	Age < 70 years			Age ≥ 70 years		
	2000	2019	AAPC (95% CI)	2000	2019	AAPC (95% CI)
**Number**						
Unsafe water, sanitation, and handwashing	3,996,889 (3,183,257 to 4,887,734)	923,837 (645,583 to 1,254,723)	-7.42 (-8.15 to -6.69)*	237,825 (168,202 to 320,654)	228,667 (159,219 to 307,023)	-0.14 (-0.42 to 0.13)
Air pollution	32,546,257 (28,559,720 to 36,241,262)	24,301,291 (20,582,581 to 28,569,480)	-1.52 (-1.70 to -1.34)*	16,789,702 (14,415,333 to 19,203,189)	18,208,412 (15,565,447 to 21,260,363)	0.27 (0.00 to 0.54)*
Other environmental risks	3,596,355 (2,485,008 to 4,876,076)	3,222,783 (1,977,748 to 4,651,395)	-0.53 (-0.77 to -0.29)*	1,790,347 (1,199,126 to 2,480,222)	3,083,288 (2,034,326 to 4,259,059)	2.86 (2.48 to 3.23)*
Child and maternal malnutrition	27,473,911 (24,705,249 to 30,472,454)	6,937,292 (6,012,640 to 7,958,846)	-6.98 (-7.79 to -6.16)*	256,375 (197,956 to 332,924)	316,491 (248,797 to 398,362)	1.15 (0.66 to 1.63)*
Tobacco	37,227,472 (33,809,017 to 40,999,710)	38,743,936 (32,232,707 to 46,296,710)	0.22 (0.08 to 0.37)*	20,037,114 (18,407,452 to 21,694,478)	25,372,064 (22,095,893 to 28,843,357)	1.24 (0.79 to 1.69)*
Alcohol use	12,593,606 (10,822,456 to 14,698,783)	13,876,946 (11,135,736 to 16,941,170)	0.47 (0.39 to 0.56)*	2,079,979 (1,635,218 to 2,555,460)	3,388,114 (2,514,663 to 4,384,540)	2.51 (2.10 to 2.92)*
Drug use	5,880,735 (5,044,491 to 6,863,455)	4,596,666 (3,725,262 to 5,556,055)	-1.24 (-1.46 to -1.02)*	420,543 (338,823 to 505,234)	525,128 (420,389 to 638,546)	1.10 (0.84 to 1.35)*
High fasting plasma glucose	10,850,938 (8,940,115 to 13,134,425)	16,082,215 (12,638,442 to 19,989,086)	2.09 (1.69 to 2.50)*	7,073,207 (5,239,551 to 9,435,756)	12,146,224 (9,068,765 to 16,392,373)	2.86 (2.43 to 3.29)*
High systolic blood pressure	21,853,891 (18,103,552 to 25,435,298)	28,919,385 (23,316,926 to 34,533,463)	1.55 (1.29 to 1.81)*	13,487,603 (10,597,533 to 16,167,287)	25,522,231 (20,992,213 to 30,524,269)	3.37 (2.90 to 3.84)*
High body-mass index	9,311,731 (3,191,606 to 17,435,563)	18,520,264 (9,033,132 to 29,514,190)	3.70 (3.59 to 3.82)*	2,392,758 (706,129 to 5,015,786)	6,309,777 (2,532,862 to 11,224,097)	5.22 (4.79 to 5.65)*
Low bone mineral density	1,234,815 (1,026,536 to 1,443,017)	1,913,892 (1,512,289 to 2,304,790)	2.37 (2.10 to 2.64)*	534,562 (448,070 to 635,290)	1,406,383 (1,058,504 to 1,740,813)	5.22 (4.98 to 5.46)*
Dietary risks	24,107,231 (19,699,737 to 29,721,184)	29,061,110 (22,436,447 to 36,581,781)	1.02 (0.85 to 1.19)*	10,146,090 (7,449,653 to 13,767,645)	17,752,016 (12,683,501 to 23,791,732)	2.90 (2.59 to 3.21)*
Low physical activity	479,307 (211,814 to 1,031,049)	793,859 (332,155 to 1,622,549)	2.75 (2.34 to 3.15)*	795,916 (391,872 to 1,491,328)	1,713,262 (787,433 to 3,275,090)	3.97 (3.34 to 4.60)*
Occupational risks	14,894,928 (12,651,846 to 17,338,599)	11,828,821 (9,596,301 to 14,324,948)	-1.20 (-1.31 to -1.10)*	3,676,403 (2,901,359 to 4,413,363)	3,658,028 (2,941,788 to 4,424,853)	-0.10 (-0.45 to 0.25)
Intimate partner violence	766,126 (417,803 to 1,191,498)	739,392 (335,422 to 1,209,468)	-0.12 (-0.36 to 0.12)*	23,715 (10,039 to 44,392)	50,534 (16,543 to 99,799)	4.12 (3.96 to 4.29)*
Unsafe sex	1,352,963 (1,174,728 to 1,769,992)	2,445,381 (1,761,748 to 2,945,569)	3.11 (2.80 to 3.42)*	129,732 (113,048 to 182,721)	279,416 (197,910 to 338,222)	4.29 (3.79 to 4.79)*
Non-optimal temperature	3,641,568 (2,714,325 to 4,594,010)	3,468,758 (2,670,613 to 4,405,174)	-0.24 (-1.11 to 0.64)	4,785,750 (4,234,955 to 5,404,845)	5,856,605 (4,952,561 to 6,867,429)	0.76 (0.49 to 1.02)*
Kidney dysfunction	6,244,259 (5,571,754 to 7,041,339)	7,536,042 (6,221,117 to 9,011,471)	1.08 (0.82 to 1.35)*	2,639,944 (2,227,097 to 3,100,279)	5,817,866 (4,717,831 to 7,022,861)	4.30 (3.63 to 4.97)*
High LDL cholesterol	8,217,261 (6,919,451 to 9,686,198)	12,184,167 (9,541,238 to 14,971,019)	2.16 (1.91 to 2.42)*	3,000,527 (1,868,209 to 4,589,459)	7,629,795 (4,751,976 to 11,248,818)	5.02 (4.62 to 5.41)*
Childhood sexual abuse and bullying	755,840 (307,189 to 1,434,919)	629,932 (269,309 to 1,156,818)	-0.95 (-1.06 to -0.84)*	6,905 (2,954 to 12,651)	16,924 (7,261 to 32,044)	4.86 (4.72 to 5.00)*
**Rate**						
Unsafe water, sanitation, and handwashing	83 (66 to 102)	20 (14 to 27)	-7.24 (-7.97 to -6.51)*	433 (306 to 584)	212 (147 to 284)	-3.66 (-3.92 to -3.40)*
Air pollution	680 (596 to 757)	523 (443 to 615)	-1.36 (-1.53 to -1.19)*	30,565 (26,242 to 34,958)	16,865 (14,417 to 19,692)	-3.04 (-3.35 to -2.74)*
Other environmental risks	75 (52 to 102)	69 (43 to 100)	-0.37 (-0.59 to -0.14)*	3,259 (2,183 to 4,515)	2,856 (1,884 to 3,945)	-0.64 (-0.95 to -0.32)*
Child and maternal malnutrition	574 (516 to 636)	149 (130 to 171)	-6.83 (-7.48 to -6.18)*	467 (360 to 606)	293 (230 to 369)	-2.43 (-2.80 to -2.06)*
Tobacco	777 (706 to 856)	835 (694 to 997)	0.38 (0.23 to 0.53)*	36,476 (33,510 to 39,494)	23,500 (20,466 to 26,716)	-2.29 (-2.71 to -1.87)*
Alcohol use	263 (226 to 307)	299 (240 to 365)	0.61 (0.33 to 0.90)*	3,786 (2,977 to 4,652)	3,138 (2,329 to 4,061)	-1.09 (-1.57 to -0.61)*
Drug use	123 (105 to 143)	99 (80 to 120)	-1.02 (-1.22 to -0.82)*	766 (617 to 920)	486 (389 to 591)	-2.34 (-2.54 to -2.15)*

For peer review only

For peer review only - http://bmjopen.bmj.com/site/about/guidelines.xhtml

High fasting plasma glucose	227 (187 to 274)	346 (272 to 431)	2.25 (1.82 to 2.69)*	12,876 (9,538 to 17,177)	11,250 (8,400 to 15,183)	-0.79 (-1.48 to -0.10)*
High systolic blood pressure	456 (378 to 531)	623 (502 to 744)	1.72 (1.47 to 1.98)*	24,553 (19,292 to 29,432)	23,639 (19,444 to 28,272)	-0.15 (-0.42 to 0.13)
High body-mass index	194 (67 to 364)	399 (195 to 636)	3.88 (3.69 to 4.07)*	4,356 (1,285 to 9,131)	5,844 (2,346 to 10,396)	1.50 (1.01 to 2.00)*
Low bone mineral density	26 (21 to 30)	41 (33 to 50)	2.54 (2.20 to 2.89)*	973 (816 to 1,157)	1,303 (980 to 1,612)	1.62 (1.38 to 1.86)*
Dietary risks	503 (411 to 621)	626 (483 to 788)	1.19 (1.02 to 1.36)*	18,470 (13,562 to 25,063)	16,442 (11,748 to 22,037)	-0.58 (-0.74 to -0.43)*
Low physical activity	10 (4 to 22)	17 (7 to 35)	2.91 (2.46 to 3.36)*	1,449 (713 to 2,715)	1,587 (729 to 3,033)	0.40 (-0.21 to 1.02)*
Occupational risks	311 (264 to 362)	255 (207 to 309)	-1.05 (-1.10 to -0.99)*	6,693 (5,282 to 8,034)	3,388 (2,725 to 4,098)	-3.51 (-3.97 to -3.05)*
Intimate partner violence	16 (9 to 25)	16 (7 to 26)	0.02 (-0.23 to 0.27)*	43 (18 to 81)	47 (15 to 92)	0.49 (0.31 to 0.66)*
Unsafe sex	28 (25 to 37)	53 (38 to 63)	3.28 (2.96 to 3.60)*	236 (206 to 333)	259 (183 to 313)	0.69 (0.16 to 1.22)*
Non-optimal temperature	76 (57 to 96)	75 (58 to 95)	-0.05 (-0.89 to 0.81)*	8,712 (7,710 to 9,839)	5,425 (4,587 to 6,361)	-2.59 (-3.60 to -1.56)*
Kidney dysfunction	130 (116 to 147)	162 (134 to 194)	1.25 (1.00 to 1.49)*	4,806 (4,054 to 5,644)	5,389 (4,370 to 6,505)	0.64 (-0.02 to 1.31)
High LDL cholesterol	172 (144 to 202)	262 (206 to 322)	2.33 (2.07 to 2.59)*	5,462 (3,401 to 8,355)	7,067 (4,401 to 10,419)	1.44 (1.04 to 1.84)*
Childhood sexual abuse and bullying	16 (6 to 30)	14 (6 to 25)	-0.80 (-0.89 to -0.72)*	13 (5 to 23)	16 (7 to 30)	1.21 (1.06 to 1.37)*

AAPC: average annual percent change; DALYs, disability adjusted life years; UI, uncertainty interval, CI, confidence interval.
*AAPC significantly differs from zero at a two-tailed level of 0.05.

For peer review only - http://bmjopen.bmj.com/site/about/guidelines.xhtml

293 **Discussion**

294 In the past two decades, although China invested hugely on heath institution, 295 personnel, and expenditures to extend life expectancy and improve healthy life expectancy, 296 population structure deteriorated, the total disease burden remained largely unchanged, 297 ageing driven disease burden continuously intensified. The trends we observed in the 298 population structure of China highlights the potential for an irreversible population decline 299 to occur before the household wealth of G7 nations is achieved in China. Despite easing of 300 birth limits in China, couples have been put off by high costs of living, expensive childcare, 301 career choices, and maternity leave. In addition, the post-working population has been 302 increasing at an accelerating rate, suggesting a clear deterioration in population structure 303 and ageing[19]. Thus, the ability of China to ease strains on its ageing population in the 304 next 30 years is a pressing concern[22].

305 On the one hand, continuous effort is needed to address the top three causes and risk 306 factors which have been identified among the population in China between 2000 and 2019. 307 Our findings show that neoplasms and cardiovascular diseases remain among the top three 308 causes, and tobacco remains among the top three risk factors for individuals younger than 309 70 years. Comprehensive regional-specific strategies, including effective tobacco-control 310 policy, recommendations for healthier lifestyles, and control of chronic infections, are 311 needed as the cancer spectrum in China is changing from that of a developing country to 312 that of a developed country[23, 24]. Regulatory reforms also need to further reduce the lag 313 between approval of new cancer drugs by the US FDA and their subsequent approval in 314 China, and they need to facilitate the development and registration of drugs that may have 315 clinical superiority over existing drugs[25]. Turning the inverted pyramid of the health care

For peer review only - http://bmjopen.bmj.com/site/about/guidelines.xhtml

system is essential for the battle against cardiovascular disease due to the increasing number of older patients, the high proportion of out-of-hospital deaths, and the gaps in lifestyle indicators between what is preferred and what is currently observed[26, 27]. All provinces in Chinese mainland have made great progress in increasing life expectancy at birth. Life course management of disease across the entire spectrum of health care services has led to a shift from a patient-centered and treatment-dominated model to a people-centered and health-centered model. It is hypothesized that the latter may help to integrate preventive and treatment measures for cardiovascular disease[28]. Tobacco sales have also increased despite a tight anti-smoking policy in China. Potential interference from the tobacco industry and the social currency of tobacco are suspected to be factors[29-31]. Therefore, great efforts should be taken not only to minimize these practices, but also to slow tobacco production[32].

On the other hand, it is crucial to focus on incremental driving causes and risk factors in the early stages to mitigate future ageing-driven disease burden. Our data show that musculoskeletal disorders, dietary risks, and high systolic blood pressure are among the top three causes and risk factors in people younger than 70 years. Split analyses were performed to examine variations according to age, and a cut-off point of 70 years was used because ischemic heart disease and stroke were the top-ranked causes of DALYs in the population aged 70 years and older[19]. A previous study quantified the temporal trends in disease burden of musculoskeletal disorders which could be attributed to changes in risk factors [27]. The results obtained support population-wide initiatives which target high body-mass index to help mitigate the burden of musculoskeletal disorders[33]. Changes in diets to correct for urbanization-driven dietary shifts to higher consumption of refined

For peer review only - http://bmjopen.bmj.com/site/about/guidelines.xhtml

sugars, refined fats, oils, and meats may also help prevent diet-related and chronic non-communicable diseases[34]. With resources available to control high systolic blood pressure and reduce the burden of hypertension, efforts are needed to improve access to better antihypertensive medications and to implement artificial intelligence-based digital health[35, 36].

We identified key domains that require intervention in order to reduce an increasing disease burden among individuals older than 70 years. Most importantly, the top three causes and risk factors for this age group remained unchanged. Thus, our findings indicate that greater effort is needed to tackle the rapidly increasing age-standardized rate of DALYs due to traditional major risk factors, including high body-mass index, low bone mineral density, and high LDL cholesterol. Moreover, the increasing burden of maternal and neonatal disorders, HIV/AIDS and sexually transmitted infections, and unintentional injuries needs to be highlighted as well.

Health care for older individuals is undergoing unprecedented challenges due to underprepared geriatric hospitals and nursing homes, an unbalanced health care insurance system between urban and rural areas, and an absence of education on national aging and post-working health[22]. Consequently, there is an urgent need to identify traits in older people who experience short ill-spans. By identifying healthy behaviors and casual genetic and metabolic factors of the latter, it may be possible to decrease the burden of late-life diseases and extend healthspan[37]. The development of long-term health systems to meet the needs of older individuals in an age-friendly environment is fundamental to fostering healthy ageing and maximizing the functional abilities of older individuals[38].

Health care has experienced an upsurge regarding medical institutions, personnel,

For peer review only - http://bmjopen.bmj.com/site/about/guidelines.xhtml

beds, and expenditures. Our findings clearly demonstrate that China has increased its health spending much faster than its economic growth. In 2018, China provided $644.7 million for the development of health assistance[39]. This trend in increasing health care expenditures will be further aggravated considering the high costs related to chronic illnesses, population growth, and ageing in the United States[9, 40]. While China has reformed its primary health care system to insure its population[7, 41], improving the quality of primary health care and increasing reimbursement for chronic non-communicable diseases are key steps to alleviating national disease burden[42, 43]. In addition, the cost-effectiveness of heath expenditures on disease burden and targeting the poor should be primary considerations in public health policy given the limited financial resources for heath[44].

Limitation of this study. It must also be noted that there were several limitations associated with the present study. First, our findings might be biased by changes in the age structure of the population and different population over a specified period because temporal trends in population and health care utilization were not quantified in terms of age standardized rate. Second, population and health care data from CSY 2020 were combined with modeled disease burden data from GBD 2019 based on an assumption that these two sources of data are consistent. Third, the most recent trends could not be captured due to time lags in data release by the NBS of China and GBD studies. Fourth, all the limitations of the GBD methodology described elsewhere [3, 19, 20] could have potentially affected the present study. Fifth, owing to the nature of the data and methods from CSY, we are currently unable to parse population estimates into more narrow age groups and

For peer review only - http://bmjopen.bmj.com/site/about/guidelines.xhtml

provide deflator-adjusted health expenditure data. National-level data were collected and analyzed, and therefore, within-country variations were not considered.

Conclusion

Comprehensive age-cause-risk factor-specific strategies are key to reconcile the tension among triangle of population ageing, disease burden and healthcare expenditure. The disease burden from cardiometabolic diseases, neoplasms and musculoskeletal disorders were identified as key domains that require intervention to reduce an increasing disease burden among individuals currently older than 70 years, as well as those approaching this age group.

For peer review only

For peer review only - http://bmjopen.bmj.com/site/about/guidelines.xhtml

Contributors

Prof. Jieruo Gu had full access to all of the data in the study and takes responsibility for the integrity of the data and the accuracy of the data analysis. Dr.Zhenguo Liang, Dr. Dongze Wu, Dr. Cui Guo, and Prof. Jieruo Gu conceived and designed the study, performed the analysis, wrote the paper. All authors reviewed and revised the paper.

Conflict of Interest

The authors confirm that there are no conflicts of interest.

Funding Sources

Project funded by China Postdoctoral Science Foundation (2020TQ0380) and Guangdong Clinical Research Center of Immune disease (2020B1111170008). The Global Burden of Disease Study is funded by the Bill and Melinda Gates Foundation. The funder was not involved in the preparation of this manuscript.

Data sharing statement

Data used for the analyses are publicly available from the Institute of Health Metrics and Evaluation (https://ghdx.healthdata.org/gbd-2019; https://ghdx.healthdata.org/gbd-2019/data-input-sources) and the National Bureau of Statistics of China (https://data.stats.gov.cn/english/index.htm; http://www.stats.gov.cn/tjsj/ndsj/2020/indexeh.htm).

Ethical approval

Data released from the Global Health Data Exchange query and the China Statistical Yearbook did not require informed patient consent. This study used an anonymized publicly available data set with no identifiable information of the survey participants. Thus, ethics approval was not required for this study.

For peer review only

For peer review only - http://bmjopen.bmj.com/site/about/guidelines.xhtml

References

1. Lopez-Otin, C., et al., *The hallmarks of aging.* Cell, 2013. **153**(6): p. 1194-217.
2. Lopez-Otin, C. and G. Kroemer, *Hallmarks of Health.* Cell, 2021. **184**(1): p. 33-63.

[dataset] 3. Collaborators, G.B.D.D., *Global age-sex-specific fertility, mortality, healthy life expectancy (HALE), and population estimates in 204 countries and territories, 1950-2019: a comprehensive demographic analysis for the Global Burden of Disease Study 2019.* Lancet, 2020. **396**(10258): p. 1160-1203.

4. Vollset, S.E., et al., *Fertility, mortality, migration, and population scenarios for 195 countries and territories from 2017 to 2100: a forecasting analysis for the Global Burden of Disease Study.* Lancet, 2020. **396**(10258): p. 1285-1306.
5. Zeng, Y. and T. Hesketh, *The effects of China's universal two-child policy.* Lancet, 2016. **388**(10054): p. 1930-1938.
6. Tatum, M., *China's three-child policy.* Lancet, 2021. **397**(10291): p. 2238.
7. Li, X., et al., *The primary health-care system in China.* Lancet, 2017. **390**(10112): p. 2584-2594.
8. Yip, W., et al., *10 years of health-care reform in China: progress and gaps in Universal Health Coverage.* Lancet, 2019. **394**(10204): p. 1192-1204.
9. Moses, H., 3rd, et al., *The anatomy of health care in the United States.* JAMA, 2013. **310**(18): p. 1947-63.
10. Zhong, N.S., et al., *Epidemiology and cause of severe acute respiratory syndrome (SARS) in Guangdong, People's Republic of China, in February, 2003.* Lancet, 2003. **362**(9393): p. 1353-8.
11. Zhu, N., et al., *A Novel Coronavirus from Patients with Pneumonia in China, 2019.* N Engl J Med, 2020. **382**(8): p. 727-733.
12. Gong, P., et al., *Urbanisation and health in China.* Lancet, 2012. **379**(9818): p. 843-52.
13. Zhou, M., et al., *Mortality, morbidity, and risk factors in China and its provinces, 1990-2017: a systematic analysis for the Global Burden of Disease Study 2017.* Lancet, 2019. **394**(10204): p. 1145-1158.
14. Zhou, M., et al., *Cause-specific mortality for 240 causes in China during 1990-2013: a systematic subnational analysis for the Global Burden of Disease Study 2013.* Lancet, 2016. **387**(10015): p. 251-72.

[dataset] 15. Fang, E.F., et al., *A research agenda for ageing in China in the 21st century (2nd edition): Focusing on basic and translational research, long-term care, policy and social networks.* Ageing Res Rev, 2020. **64**: p. 101174.

[dataset] 16. Li, J., et al., *Spatiotemporal matching between medical resources and population ageing in China from 2008 to 2017.* BMC Public Health, 2020. **20**(1): p. 845.

[dataset] 17. Hu, D., et al., *Age-Related Disease Burden in China, 1997-2017: Findings From the Global Burden of Disease Study.* Front Public Health, 2021. **9**: p. 638704.

18. Ning, J.J., et al. *China Statistical Yearbook 2020*. Available from: http://www.stats.gov.cn/tjsj/ndsj/2020/indexeh.htm.

For peer review only - http://bmjopen.bmj.com/site/about/guidelines.xhtml

19. *Diseases, G.B.D. and C. Injuries, Global burden of 369 diseases and injuries in 204 countries and territories, 1990-2019: a systematic analysis for the Global Burden of Disease Study 2019. .* Lancet, 2020. **396**(10258): p. 1204-1222.
20. Collaborators, G.B.D.R.F., *Global burden of 87 risk factors in 204 countries and territories, 1990-2019: a systematic analysis for the Global Burden of Disease Study 2019.* Lancet, 2020. **396**(10258): p. 1223-1249.
21. *NCD Countdown 2030: worldwide trends in non-communicable disease mortality and progress towards Sustainable Development Goal target 3.4.* Lancet, 2018. **392**(10152): p. 1072-1088.
22. Wang, X.Q. and P.J. Chen, *Population ageing challenges health care in China.* Lancet, 2014. **383**(9920): p. 870.
23. Feng, R.M., et al., *Current cancer situation in China: good or bad news from the 2018 Global Cancer Statistics?* Cancer Commun (Lond), 2019. **39**(1): p. 22.
24. Chen, W., et al., *Disparities by province, age, and sex in site-specific cancer burden attributable to 23 potentially modifiable risk factors in China: a comparative risk assessment.* Lancet Glob Health, 2019. **7**(2): p. e257-e269.
25. Li, G., et al., *Characteristics of expedited programmes for cancer drug approval in China.* Nat Rev Drug Discov, 2021. **20**(6): p. 416.
26. Zhao, D., et al., *Epidemiology of cardiovascular disease in China: current features and implications.* Nat Rev Cardiol, 2019. **16**(4): p. 203-212.
27. Du, X., et al., *Epidemiology of Cardiovascular Disease in China and Opportunities for Improvement: JACC International.* J Am Coll Cardiol, 2019. **73**(24): p. 3135-3147.
28. Liu, S., Y. Li, and M. Zhou, *Addressing the Cardiovascular Disease Burden in China-Is It Possible Without Surgery?-Reply.* JAMA Cardiol, 2019. **4**(9): p. 953.
29. Luo, H. and Y. Zhao, *Increasing tobacco sales under the anti-smoking policy in China.* Lancet, 2020. **395**(10240): p. 1834.
30. Yang, G., et al., *The road to effective tobacco control in China.* Lancet, 2015. **385**(9972): p. 1019-28.
31. Rich, Z.C. and S. Xiao, *Tobacco as a social currency: cigarette gifting and sharing in China.* Nicotine Tob Res, 2012. **14**(3): p. 258-63.
32. Dan, X., S. Yuankai, and W. Chen, *Tobacco in China.* Lancet, 2014. **383**(9934): p. 2045-2046.
33. Wu, D., et al., *Pattern and trend of five major musculoskeletal disorders in China from 1990 to 2017: findings from the Global Burden of Disease Study 2017.* BMC Med, 2021. **19**(1): p. 34.
34. Tilman, D. and M. Clark, *Global diets link environmental sustainability and human health.* Nature, 2014. **515**(7528): p. 518-22.
35. Su, M., et al., *Availability, cost, and prescription patterns of antihypertensive medications in primary health care in China: a nationwide cross-sectional survey.* Lancet, 2017. **390**(10112): p. 2559-2568.
36. Brouwers, S., et al., *Arterial hypertension.* Lancet, 2021.
37. Partridge, L., J. Deelen, and P.E. Slagboom, *Facing up to the global challenges of ageing.* Nature, 2018. **561**(7721): p. 45-56.
38. Beard, J.R., et al., *The World report on ageing and health: a policy framework for healthy ageing.* Lancet, 2016. **387**(10033): p. 2145-2154.

For peer review only - http://bmjopen.bmj.com/site/about/guidelines.xhtml

39. Global Burden of Disease Health Financing Collaborator, N., *Past, present, and future of global health financing: a review of development assistance, government, out-of-pocket, and other private spending on health for 195 countries, 1995-2050.* Lancet, 2019. **393**(10187): p. 2233-2260.
40. Dieleman, J.L., et al., *Factors Associated With Increases in US Health Care Spending, 1996-2013.* JAMA, 2017. **318**(17): p. 1668-1678.
41. The, L., *China's health-care reform: an independent evaluation.* Lancet, 2019. **394**(10204): p. 1113.
42. Jan, S., et al., *Action to address the household economic burden of non-communicable diseases.* Lancet, 2018. **391**(10134): p. 2047-2058.
43. Li, X., et al., *Quality of primary health care in China: challenges and recommendations.* Lancet, 2020. **395**(10239): p. 1802-1812.
44. Li, H. and L. Jiang, *Catastrophic medical insurance in China.* Lancet, 2017. **390**(10104): p. 1724-1725.

For peer review only

For peer review only - http://bmjopen.bmj.com/site/about/guidelines.xhtml

Figure legends

Figure 1. Temporal trends of population in China between 2000 and 2019.

Temporal trends of population composition are presented according to gender and area (A), age group (B), birth rate, death rate, and natural growth rate (C), and gross ratio, children ratio, and old dependency ratio (D).

Supplemental appendix

sFigure 1. Trend in life expectancy and healthy life expectancy at birth and at 60-64 years according to gender between 2000 and 2019.

sTable 1. The average annual percent change in life expectancy at birth and at 60-64 years for 2000-2019

sTable 2. Percentage of healthy life expectancy within total life expectancy at birth for 2000-2019

sTable 3. Percentage of healthy life expectancy within total life expectancy at 60-64 years for 2000-2019

sTable 4. The average annual percent change of all-cause disease burden for 2000-2019

sTable 5. The average annual percent change of causes for 2000-2019

sTable 6. The average annual percent change of risk factors for 2000-2019

For peer review only

For peer review only - http://bmjopen.bmj.com/site/about/guidelines.xhtml

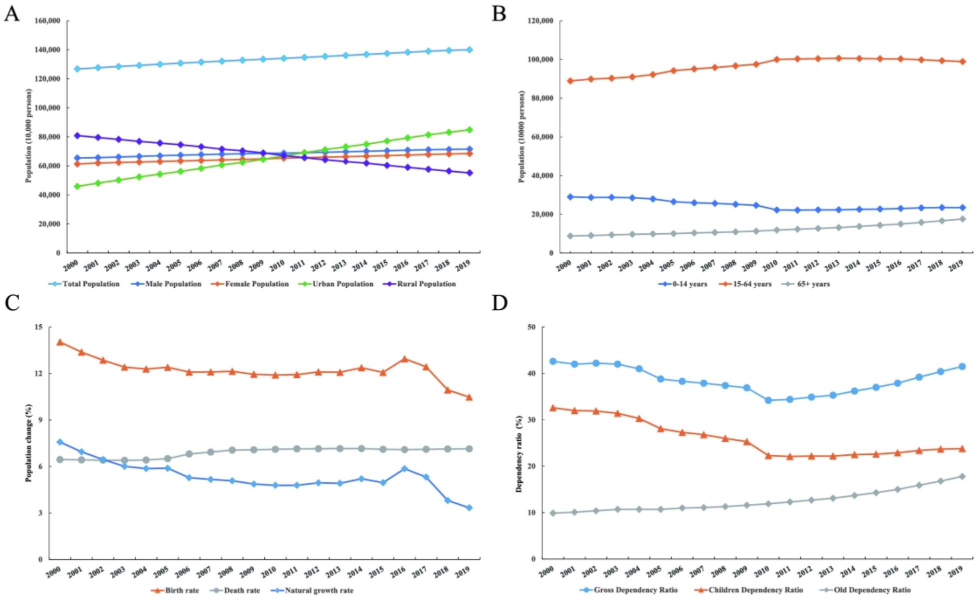

Temporal trends of population in China between 2000 and 2019.

200x123mm (300 x 300 DPI)

Supplemental Appendix

Temporal trend of population structure, burden of diseases, healthcare resources and expenditure in China, 2000-2019

Table of Contents

Glossary and Definitions

Section 1: Population

Section 2: Health Care Institutions

Section 3: Medical Personnel

Section 4: Beds in Health Care Institutions

Section 5: Health Expenditures

Supplementary Figures

sFigure 1. Trend in life expectancy and healthy life expectancy at birth and at 60–64 years according to gender between 2000 and 2019.

Supplementary Tables

sTable 1. The average annual percent change in life expectancy at birth and at 60–64 years for 2000–2019

sTable 2. Percentage of healthy life expectancy within total life expectancy at birth for 2000–2019

sTable 3. Percentage of healthy life expectancy within total life expectancy at 60–64 years for 2000–2019

sTable 4. The average annual percent change of all-cause disease burden for 2000–2019

sTable 5. The average annual percent change of causes for 2000–2019

sTable 6. The average annual percent change of risk factors for 2000–2019

For peer review only

Glossary and Definitions

Glossary and definitions of population, healthcare institutions, medical personnel, beds in health care institutions, and health expenditure data were compiled from the 2020 China Statistical Yearbook (CSY) (http://www.stats.gov.cn/tjsj/ndsj/2020/indexeh.htm).

Section 1: Population

Total Population (year-end) (10000 persons): Year-end population refers to the population at midnight on 31 December. Annual statistics regarding the total population do not include data for Hong Kong, the Macau special administrative region and Taiwan province, and overseas numbers.

Urban Population refers to people residing in cities and towns in China.

Rural Population refers to people who reside outside of urban areas.

Birth Rate, Death Rate, and Natural Growth Rate of Population

Birth Rate (or Crude Birth Rate) refers to the ratio of the number of births to the average population (or mid-period population) during a certain period of time (usually a year). Birth rate in the chapter refers to annual birth rate and it is calculated as: number of births / annual average population × 1000. Number of births in the formula refers to live births (i.e., when a baby has breathed or showed any vital phenomena regardless of duration of gestation). Annual average population is the average of the population at the beginning of the year and the population at the end of the year. Sometimes it is substituted by the mid-year population.

Death Rate (or Crude Death Rate) refers to the ratio of the number of deaths to the average population (or mid-period population) during a certain period of time (usually a year). Death rate in the chapter refers to annual death rate and is calculated as: number of deaths / annual average population × 1000. Total Population (year-end) (10000 persons) refers to the population at midnight on 31 December.

Gross Dependency Ratio (or Coefficient) (%) refers to the ratio of non-working-age population to the working-age population. In general, Gross Dependency Ratio describes the number of non-working-age individuals that every 100 people of working age take care of. This reflects the basic relation between population and economic development from a demographic perspective.

Children Dependency Ratio (or Coefficient) (%) refers to the ratio of children to the working-age population. It describes the number of children that every 100 people of working age take care of.

Old Dependency Ratio (or Coefficient) (%) refers to the ratio of the elderly to the working-age population. It describes the number of elderly that every 100 people of working age take care of. Old dependency ratio is one of the indicators that reflect the social implication of population aging from an economic perspective.

Note: For the years, 2000 and 2010, census year estimates were available; the rest of the data were estimated based on annual national sample surveys.

Section 2: Health Care Institutions

Number of Health Care Institutions (unit)

Medical and health care institutions refer to units which have qualified to receive a Certification of Health Care Institution by the administration of public health, or a Certification of Corporate Unit by the civil affairs, administration for industry and commerce, commission office for public sector reform. Health care institutions engage in medical care, disease prevention and control, health supervision and inspection, medicine research, and on-job training. Health care institutions include hospitals, health care institutions at the grass-root level, specialized public health institutions, and other medical and health care institutions.

Number of Hospitals (unit)

Hospitals were considered to include general hospitals, hospitals of traditional Chinese medicine, hospitals that integrate traditional Chinese medicine and Western medicine, national hospitals, specialized hospitals, and nursing hospitals. Specialized disease prevention and cure centers, maternal and child health hospitals, and sanatoriums were excluded.

For peer review only

Health Care Institutions at Grass-root Level (unit)

Health care institutions at the grass-root level included: community health service centers, community service stations, institutes of health on the street, institutes of health in villages and towns, outpatient departments, and clinics (health centers).

Note:
1. Number of village clinics was included in health care institutions.
2. Since 2002, health care institutions no longer include headquarters of higher and secondary medical schools, drug test institutions, frontier health and quarantine institutions, and family.

Section 3: Medical Personnel

Number of Medical Personnel (10000 persons)

Medical personnel refer to all employees engaged at health care institutions, such as hospitals, health care institutions at grass-root level, specialized public health institutions, and other medical and health care institutions. These employees include medical technical personnel, village doctors and assistants, other technical personnel, and managerial and service staff. Medical personnel also refer to workers with payment of wages at the end of the year, including various types of employment personnel (including contract workers) and staff rehired for more than six months. Temporary workers, emeritus and retired, persons leaving the unit while remaining labor relations, and rehired and temporarily employed individuals for less than six months were excluded.

Number of Licensed Doctors (10000 persons)

Licensed doctors refer to medical workers who have obtained licenses of qualified doctors and are employed in medical treatment, disease prevention, or healthcare institutions. Licensed doctors engaged in management positions were excluded. Licensed doctors are divided into four categories: clinician, Chinese medicine physician, dentist, and public health physician.

Number of Registered Nurses (10000 persons)

Registered nurses refer to practical nursing personnel with a certificate of registered nurse. Nurses engaged in management positions were excluded.

Number of Other Technical Personnel (10000 persons)

Other technical personnel represent staff who graduated from high school and other institutions in the majors of chemistry and mathematics, as well as other non-health professionals, who are engaged in health promotion, research, teaching, and other technical work.

Number of Administrative Personnel (10000 persons)

Administrative personnel include persons in charge, personnel mainly engaged in health care, disease control, health surveillance, medical research, and teaching, other business management, and personnel mainly engaged in the party and government, personnel, finance, information, security, and other administrative work.

Note: Data of medical personnel and medical technical personnel include 10000 sanitation supervisors who serve as public servants.

Section 4: Beds in Health Care Institutions

Number of Beds in Health Care Institutions (10000 units)

Number of beds in health care institutions refers to a fixed sum of beds (non-prepared beds) at the end of the year. These include formal beds, simple beds, care beds, beds being disinfected and repaired, and disabled beds due to expansion or overhaul. Obstetric neonatal beds, expectant beds of confinement rooms, inventory beds, observation beds, extra beds, and escort beds for patient's family members were excluded.

Number of Beds in Medical Institutions per 10000 Population

Number of beds in medical institutions refers to a fixed sum of beds (non-prepared beds) at the end of the year. These include formal beds, simple beds, care beds, beds being disinfected and repaired, and disabled beds due to expansion or overhaul. Obstetric neonatal beds, expectant beds of confinement rooms, inventory beds, observation beds, extra beds, and escort beds for patient's family members were excluded.

For peer review only

Number of Beds in Urban Medical Institutions (10000 units)

Number of beds in urban medical institutions refers to a fixed sum of beds (non-prepared beds) at the end of the year. These include formal beds, simple beds, care beds, beds being disinfected and repaired, and disabled beds due to expansion or overhaul. Obstetric neonatal beds, expectant beds of confinement rooms, inventory beds, observation beds, extra beds, and escort beds for patient's family members were excluded.

Number of Beds in Rural Medical Institutions (10000 units)

Number of beds in rural medical institutions refers to a fixed sum of beds (non-prepared beds) at the end of the year. These include formal beds, simple beds, care beds, beds being disinfected and repaired, and disabled beds due to expansion or overhaul. Obstetric neonatal beds, expectant beds of confinement rooms, inventory beds, observation beds, extra beds, and escort beds for patient's family members were excluded.

Beds of Medical Institutions per 10000 Population

Beds of Medical Institutions per 10000 Population = number of beds of medical and health care institutions / population ×10000. The population value used is based on household registration data from the Ministry of Public Security.

Section 5: Health Expenditures

Total Health Expenditure (100 million yuan)

Total health expenditure refers to the total expenditure on medical and health care services (usually in monetary value) by a country or region within a certain period of time (usually a year). Total health expenditure is counted according to the source method. Under certain economic conditions, it reflects the emphasis and burden level of cost on government health care, the society’s healthcare, and on individual citizens. Total health expenditure also represents the main features of health financing mode and fairness, as well as the rationality of health financing.

Government Health Expenditure (100 million yuan)

Government expenditure on public health refers to expenditures by the government at all levels of medical and health care services, medical subsidies, health administration and health insurance management, and undertakings of family planning.

Social Health Expenditure (100 million yuan)

Social expenditure on public health refers to all inputs from society, and not from the government in the form of expenditures on social medical security, commercial health insurance, private expenditure on operation of medical and health care, social donations and contributions, and income from administrative fees.

Out-of-pocket Health Expenditure (100 million yuan)

Individual cash expenditure on health refers to the expenditure in cash by rural and urban residents for various health services, including self-payments of residents within the system of multi-medical insurance.

Per Capita Health Expenditure (yuan)

Per capita health expenditure refers to the ratio of total expenditure on health in a year to the average population.

For peer review only

Supplementary Figures

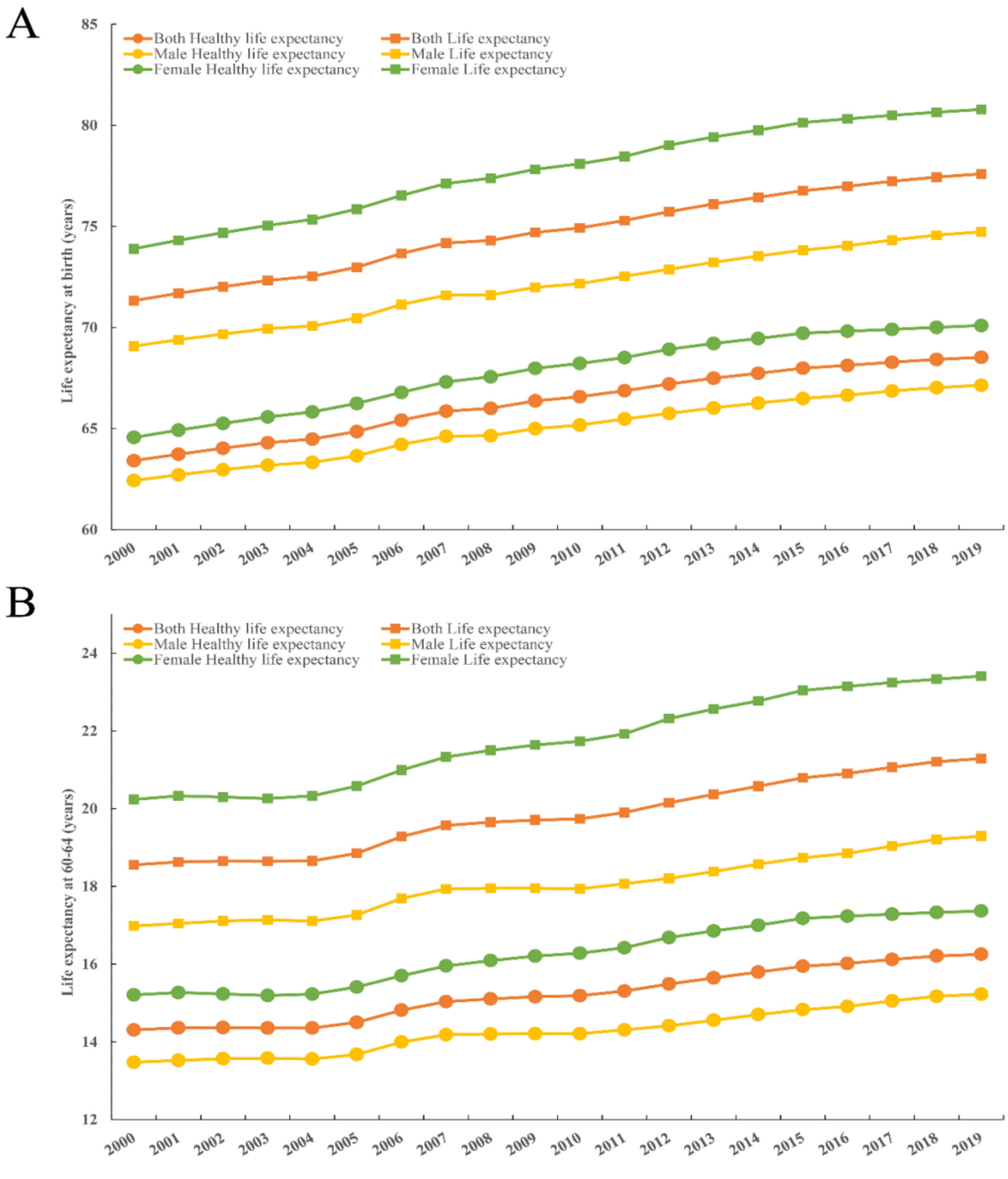

sFigure 1. Trend in life expectancy and healthy life expectancy at birth and at 60–64 years according to gender between 2000 and 2019.
The trend of life expectancy and healthy life expectancy at birth (A) and at 60–64 years (B) according to gender between 2000 and 2019. The distance between life expectancy and healthy life expectancy denotes unhealthy life expectancy (ill-span).

Supplementary Tables

sTable 1. The average annual percent change in life expectancy at birth and at 60–64 years for 2000–2019

	Life expectancy (95% UI)		AAPC (95% CI)		
	2000	2019	2000–2019	2000–2009	2010–2019
**Life expectancy at birth**					
Both					
Life expectancy	71.33 (70.56 to 72.06)	77.59 (76.27 to 79.00)	0.44 (0.43 to 0.46)*	0.50 (0.47 to 0.53)*	0.40 (0.38 to 0.42)*
Healthy life expectancy	63.42 (60.07 to 65.54)	68.53 (60.88 to 70.97)	0.41 (0.38 to 0.43)*	0.50 (0.45 to 0.54)*	0.31 (0.29 to 0.34)*
Male					
Life expectancy	69.08 (68.02 to 70.16)	74.74 (72.78 to 76.70)	0.42 (0.38 to 0.46)*	0.46 (0.37 to 0.54)*	0.39 (0.37 to 0.41)*
Healthy life expectancy	62.43 (60.44 to 64.36)	67.14 (60.64 to 69.82)	0.38 (0.36 to 0.41)*	0.44 (0.39 to 0.49)*	0.32 (0.30 to 0.35)*
Female					
Life expectancy	73.90 (72.91 to 74.81)	80.78 (79.10 to 82.47)	0.47 (0.43 to 0.51)*	0.57 (0.50 to 0.63)*	0.37 (0.32 to 0.41)*
Healthy life expectancy	64.57 (60.86 to 66.96)	70.10 (60.92 to 73.19)	0.43 (0.40 to 0.46)*	0.56 (0.50 to 0.62)*	0.30 (0.27 to 0.33)*
**Life expectancy at 60–64 years**					
Both					
Life expectancy	18.56 (18.06 to 19.02)	21.29 (20.32 to 22.33)	0.76 (0.60 to 0.91)*	0.65 (0.37 to 0.93)*	0.91 (0.81 to 1.01)*
Healthy life expectancy	14.31 (13.16 to 15.43)	16.26 (14.82 to 17.67)	0.69 (0.51 to 0.87)*	0.64 (0.27 to 1.01)*	0.73 (0.66 to 0.80)*
Male					
Life expectancy	16.99 (16.32 to 17.66)	19.29 (17.97 to 20.68)	0.68 (0.61 to 0.74)*	0.58 (0.46 to 0.70)*	0.85 (0.80 to 0.89)*
Healthy life expectancy	13.48 (12.42 to 14.53)	15.23 (13.82 to 16.79)	0.65 (0.55 to 0.76)*	0.56 (0.37 to 0.75)*	0.81 (0.76 to 0.86)*
Female					
Life expectancy	20.24 (19.62 to 20.86)	23.41 (22.10 to 24.77)	0.76 (0.55 to 0.98)*	0.75 (0.41 to 1.09)*	0.76 (0.47 to 1.04)*
Healthy life expectancy	15.21 (13.83 to 16.46)	17.37 (15.61 to 19.16)	0.70 (0.54 to 0.85)*	0.69 (0.40 to 0.98)*	0.68 (0.54 to 0.81)*

Note: AAPC: average annual percent changes; UI, uncertainty interval; CI, confidence interval.
*AAPC significantly differs from zero at the alpha = 0.05 level.

For peer review only

sTable 2. Percentage of healthy life expectancy within total life expectancy at birth for 2000–2019

Year	Healthy life expectancy at birth (years)			Life expectancy at birth (years)			Healthy within total life expectancy (%)		
	Total	Male	Female	Total	Male	Female	Total	Male	Female
2000	63.42	62.43	64.57	71.33	69.08	73.90	88.90	90.38	87.37
2001	63.73	62.72	64.92	71.69	69.40	74.31	88.90	90.37	87.37
2002	64.03	62.96	65.26	72.02	69.67	74.69	88.90	90.37	87.38
2003	64.30	63.20	65.57	72.33	69.94	75.05	88.89	90.36	87.38
2004	64.49	63.33	65.83	72.54	70.08	75.34	88.90	90.37	87.37
2005	64.86	63.66	66.25	72.98	70.47	75.86	88.87	90.34	87.33
2006	65.42	64.22	66.79	73.67	71.14	76.54	88.80	90.27	87.27
2007	65.87	64.62	67.31	74.19	71.60	77.12	88.79	90.25	87.27
2008	66.00	64.65	67.57	74.31	71.61	77.38	88.83	90.28	87.32
2009	66.38	65.00	67.98	74.70	71.99	77.82	88.85	90.30	87.35
2010	66.58	65.18	68.22	74.92	72.17	78.10	88.87	90.31	87.36
2011	66.88	65.48	68.51	75.29	72.53	78.46	88.82	90.27	87.31
2012	67.21	65.75	68.92	75.73	72.88	79.03	88.75	90.22	87.21
2013	67.50	66.03	69.21	76.11	73.23	79.42	88.69	90.17	87.14
2014	67.74	66.26	69.45	76.44	73.53	79.76	88.62	90.11	87.08
2015	67.99	66.49	69.72	76.77	73.83	80.14	88.56	90.06	87.00
2016	68.12	66.65	69.82	76.98	74.05	80.32	88.49	90.01	86.93
2017	68.28	66.86	69.91	77.23	74.33	80.50	88.41	89.95	86.85
2018	68.42	67.03	70.01	77.44	74.57	80.65	88.36	89.89	86.81
2019	68.53	67.14	70.10	77.59	74.74	80.78	88.31	89.84	86.78

For peer review only

sTable 3. Percentage of healthy life expectancy within total life expectancy at 60–64 years for 2000–2019

Year	Healthy life expectancy at 60–64 years			Life expectancy at 60–64 year			Percentage of healthy within total life expectancy		
	Total	Male	Female	Total	Male	Female	Total	Male	Female
2000	14.31	13.48	15.21	18.56	16.99	20.24	77.12%	79.35%	75.15%
2001	14.36	13.52	15.27	18.63	17.05	20.33	77.09%	79.33%	75.11%
2002	14.37	13.57	15.23	18.65	17.11	20.30	77.05%	79.29%	75.06%
2003	14.36	13.58	15.19	18.65	17.14	20.26	76.99%	79.24%	74.98%
2004	14.36	13.56	15.23	18.66	17.11	20.33	76.97%	79.24%	74.93%
2005	14.50	13.68	15.41	18.85	17.27	20.58	76.93%	79.21%	74.87%
2006	14.82	14.00	15.70	19.29	17.69	20.99	76.84%	79.12%	74.80%
2007	15.04	14.18	15.95	19.57	17.93	21.33	76.82%	79.09%	74.79%
2008	15.11	14.20	16.09	19.65	17.95	21.50	76.87%	79.12%	74.84%
2009	15.16	14.21	16.21	19.71	17.95	21.64	76.93%	79.17%	74.90%
2010	15.19	14.21	16.28	19.74	17.94	21.73	76.96%	79.21%	74.93%
2011	15.31	14.31	16.42	19.90	18.07	21.93	76.94%	79.20%	74.89%
2012	15.49	14.42	16.69	20.15	18.21	22.32	76.87%	79.19%	74.78%
2013	15.65	14.56	16.86	20.37	18.39	22.56	76.82%	79.18%	74.71%
2014	15.80	14.71	17.00	20.58	18.58	22.77	76.77%	79.16%	74.65%
2015	15.95	14.83	17.18	20.79	18.74	23.04	76.69%	79.13%	74.55%
2016	16.02	14.91	17.23	20.91	18.85	23.15	76.62%	79.11%	74.45%
2017	16.12	15.05	17.28	21.07	19.04	23.25	76.54%	79.08%	74.35%
2018	16.21	15.17	17.33	21.21	19.21	23.33	76.45%	78.99%	74.28%
2019	16.26	15.23	17.37	21.29	19.29	23.41	76.36%	78.93%	74.18%

For peer review only

sTable 4. The average annual percent change of all-cause disease burden for 2000–2019

Subcategory	All-cause estimates (95% UI)		AAPC (95% CI)		
	2000	2019	2000–2019	2000–2009	2010–2019
**Number**					
Incidence	5,291,848,308 (4,993,009,197 to 5,644,956,487)	5,790,243,111 (5,505,879,019 to 6,107,248,437)	0.48 (0.45 to 0.50)*	0.19 (0.16 to 0.22)*	0.82 (0.78 to 0.85)*
Prevalence	1,241,468,252 (1,234,531,622 to 1,247,534,242)	1,362,817,881 (1,356,594,402 to 1,368,714,232)	0.49 (0.49 to 0.50)*	0.47 (0.47 to 0.48)*	0.52 (0.51 to 0.52)*
Death	8,653,295 (8,170,487 to 9,200,531)	10,653,448 (9,310,978 to 12,069,760)	1.12 (0.93 to 1.31)*	0.89 (0.61 to 1.18)*	1.27 (1.03 to 1.51)*
Years lived with disability	116,137,933 (86,494,934 to 150,490,082)	153,797,874 (115,409,603 to 199,426,123)	1.49 (1.46 to 1.52)*	1.29 (1.24 to 1.34)*	1.74 (1.72 to 1.77)*
Years of life lost	260,536,746 (246,148,674 to 276,354,908)	228,407,694 (197,155,841 to 260,733,993)	-0.70 (-0.95 to -0.46)*	-1.34 (-1.53 to -1.15)*	-0.07 (-0.51 to 0.38)
DALY	376,674,679 (343,098,150 to 416,187,725)	382,205,568 (332,307,099 to 433,824,950)	0.08 (-0.06 to 0.22)	-0.44 (-0.58 to -0.31)*	0.60 (0.39 to 0.82)*
Risk factor (DALY)	176,792,905 (163,998,804 to 190,556,819)	187,705,961 (165,023,927 to 212,244,649)	0.34 (0.20 to 0.48)*	-0.27 (-0.52 to -0.03)*	0.92 (0.79 to 1.06)*
**Age standardized rate**					
Incidence	414,111 (390,563 to 440,957)	418,081 (394,196 to 444,709)	0.04 (0.02 to 0.07)*	-0.21 (-0.24 to -0.17)*	0.34 (0.30 to 0.38)*
Prevalence	94,824 (94,187 to 95,367)	93,439 (92,723 to 94,090)	-0.08 (-0.09 to -0.07)*	-0.17 (-0.19 to -0.15)*	0.00 (-0.01 to 0.00)
Death	956 (910 to 1,010)	634 (561 to 711)	-2.14 (-2.28 to -2.00)*	-2.00 (-2.20 to -1.79)*	-2.38 (-2.56 to -2.21)*
Years lived with disability	9,078 (6,779 to 11,713)	8,924 (6,654 to 11,539)	-0.08 (-0.12 to -0.05)*	-0.34 (-0.38 to -0.30)*	0.22 (0.17 to 0.28)*
Years of life lost	24,833 (23,513 to26,239)	13,347 (11,701 to14,997)	-3.22 (-3.33 to -3.11)*	-3.43 (-3.59 to -3.27)*	-3.09 (-3.23 to -2.96)*
DALY	33,911 (31,131 to 37,035)	22,271 (19,482 to 25,195)	-2.21 (-2.35 to -2.07)*	-2.54 (-2.64 to -2.43)*	-1.85 (-2.12 to -1.58)*
Risk factor (DALY)	16,689 (15,569 to 17,886)	10,310 (9,122 to 11,524)	-2.49 (-2.69 to -2.29)*	-2.93 (-3.27 to -2.58)*	-2.07 (-2.30 to -1.85)*

Note: AAPC: average annual percent changes; UI, uncertainty interval; CI, confidence interval; DALY, disability-adjusted life years. *AAPC significantly differs from zero at the alpha = 0.05 level.

For peer review only

sTable 5. The average annual percent change of causes for 2000–2019

Causes	DALY (95% UI)				AAPC (95% CI)		
	2000	Rank	2019	Rank	2000–2019	2000–2009	2010–2019
**All-age numbers**							
Neglected tropical diseases and malaria	1,704,065 (1,121,580 to 2,525,119)	21	1,137,806 (692, 480 to 1,800,439)	22	-2.07 (-2.35 to -1.79)*	-3.57 (-3.89 to -3.25)*	-0.50 (-0.97 to -0.02)*
Nutritional deficiencies	4,183,588 (3,082,941 to 5,605,252)	19	2,606,926 (1,815,566 to 3,659,294)	18	-2.47 (-2.64 to -2.30)*	-4.30 (-4.49 to -4.11)*	-0.53 (-0.79 to -0.26)*
Neoplasms	54,415,394 (50,247,614 to 58,721,812)	2	67,519,830 (1,418,891 to 3,248,407)	2	1.13 (0.95 to 1.31)*	0.87 (0.57 to 1.17)*	1.42 (1.20 to 1.65)*
Cardiovascular diseases	67,584,492 (62,771,497 to 73,832,854)	1	91,933,122 (1,135,346 to 3,124,922)	1	1.62 (1.45 to 1.79)*	1.70 (1.40 to 2.00)*	1.42 (1.32 to 1.53)*
Chronic respiratory diseases	28,653,966 (23,770,991 to 30,931,146)	3	22,520,549 (1,719,779 to 3,416,880)	4	-1.28 (-1.50 to -1.06)*	-2.02 (-2.40 to -1.65)*	-0.50 (-0.75 to -0.26)*
Digestive diseases	11,699,071 (10,590,221 to 12,920,126)	12	10,017,880 (1,549,912 to 3,496,363)	12	-0.80 (-0.87 to -0.73)*	-1.02 (-1.13 to -0.90)*	-0.51 (-0.60 to -0.43)*
Neurological disorders	12,506,303 (6,994,212 to 20,592,252)	11	17,381,406 (1,666,405 to 3,082,962)	7	1.75 (1.67 to 1.82)*	1.79 (1.70 to 1.89)*	1.72 (1.61 to 1.84)*
Mental disorders	18,110,029 (13,449,072 to 23,810,039)	9	20,293,827 (1,145,615 to 3,345,133)	5	0.59 (0.49 to 0.69)*	0.45 (0.40 to 0.49)*	0.75 (0.54 to 0.95)*
Musculoskeletal disorders	20,089,552 (14,362,360 to 26,771,117)	7	29,933,924 (1,427,199 to 3,823,644)	3	2.10 (2.04 to 2.16)*	2.44 (2.36 to 2.52)*	1.75 (1.67 to 1.83)*
Other non-communicable diseases	24,572,991 (20,333,568 to 29,891,261)	4	19,126,072 (1,446,224 to 3,590,726)	6	-1.31 (-1.48 to -1.13)*	-2.43 (-2.53 to -2.33)*	-0.04 (-0.40 to -032)
Skin and subcutaneous diseases	7,809,025 (5,108,660 to 11,567,830)	16	8,264,702 (1,394,816 to 3,356,265)	13	0.29 (0.27 to 0.32)*	0.33 (0.29 to 0.38)*	0.28 (0.26 to 0.29)*
Sense organ diseases	10,740,782 (7,311,356 to 15,203,366)	14	15,981,667 (1,788,420 to 3,997,480)	9	2.14 (1.98 to 2.30)*	1.98 (1.94 to 2.03)*	2.31 (1.97 to 2.64)*
Transport injuries	16,631,845 (15,221,113 to 18,940,342)	10	13,745,455 (1,719,684 to 3,768,996)	11	-1.02 (-1.35 to -0.69)*	0.57 (0.21 to 0.95)*	-2.66 (-3.22 to -2.10)*
Unintentional injuries	21,114,052 (19,362,287 to 23,062,953)	6	16,003,462 (1,056,699 to 3,613,615)	8	-1.53 (-2.01 to -1.04)*	-1.53 (-2.01 to -1.04)*	-1.53 (-2.01 to -1.04)*
Self-harm and interpersonal violence	11,171,276 (9,775,023 to 12,180,298)	13	5,870,049 (1,030,733 to 3,924,034)	16	-3.35 (-3.70 to -3.01)*	-4.09 (-4.73 to -3.45)*	-2.47 (-2.75 to -2.18)*
HIV/AIDS and sexually transmitted infections	921,444,444 (727,466,466 to 1,222,963)	22	1,752,419 (1,430, 192 to 2,114,213)	19	3.47 (2.72 to 4.22)*	3.07 (2.38 to 3.77)*	4.07 (2.71 to 5.45)*
Respiratory infections and tuberculosis	21,938,222 (20,220,847 to 23,581,022)	5	6,647,872 (1,853,547 to 3,613,257)	15	-6.10 (-6.41 to -5.79)*	-8.88 (-9.36 to -8.40)*	-3.34 (-3.65 to -3.03)*
Enteric infections	3,407,267 (2,990,655 to 3,948,769)	20	1,408,098 (1,047,418 to 3,827,650)	20	-4.41 (-5.28 to -3.54)*	-9.05 (-9.96 to -8.13)*	0.68 (-0.56 to 1.92)
Other infectious diseases	4,581,989 (3,724,438 to 6,219,671)	18	1,244,099 (1,063,407 to 3,462,432)	21	-6.57 (-6.95 to -6.19)*	-9.03 (-9.45 to -8.61)*	-4.04 (-4.71 to -3.36)*
Maternal and neonatal disorders	18,110,589 (16,543,873 to 19,663,056)	8	7,151,571 (1,273,977 to 3,174,922)	14	-4.82 (-5.40 to -4.23)*	-6.06 (-6.60 to -5.51)*	-3.56 (-4.60 to -2.51)*
Substance use disorders	6,291,914 (5,043,188 to 7,751,372)	17	5,755,658 (1,373,805 to 3,343,432)	17	-0.39 (-0.82 to 0.03)	-1.74 (-2.49 to -0.99)*	0.94 (0.53 to 1.36)*
Diabetes and kidney diseases	10,436,824 (9,083,252 to 11,910,785)	15	15,909,173 (1,346,885 to 3,824,247)	10	2.30 (2.09 to 2.51)*	2.87 (2.44 to 3.31)*	1.79 (1.70 to 1.89)*
**Age-standardized rate**							
Neglected tropical diseases and malaria	132 (89 to 194)	21	68 (42 to 107)	22	-3.40 (-3.66 to -3.13)*	-4.75 (-5.06 to -4.45)*	-1.97 (-2.42 to -1.51)*
Nutritional deficiencies	369 (284 to 480)	20	175 (122 to 241)	18	-3.88 (-4.07 to -3.69)*	-5.98 (-6.27 to -5.70)*	-1.84 (-2.11 to -1.58)*
Neoplasms	4,505 (4,163 to 4,847)	2	3,421 (2,933 to 3,946)	2	-1.46 (-1.71 to -1.21)*	-1.62 (-1.88 to -1.36)*	-1.25 (-1.68 to -1.81)*
Cardiovascular diseases	6,441 (5,997 to 7,005)	1	4,938 (4,318 to 5,577)	1	-1.40 (-1.56 to -1.24)*	-1.10 (-1.39 to -0.82)*	-1.84 (-1.94 to -1.75)*
Chronic respiratory diseases	2,947 (2,458 to 3,175)	3	1,270 (1,114 to 1,474)	5	-4.34 (-4.61 to -4.07)*	-4.90 (-5.37 to -4.44)*	-3.74 (-4.04 to -3.45)*
Digestive diseases	974 (889 to 1,070)	12	539 (463 to 616)	14	-3.07 (-3.14 to -3.00)*	-3.29 (-3.41 to -3.16)*	-2.77 (-2.84 to -2.71)*
Neurological disorders	1,103 (650 to 1,773)	11	1,077 (598 to 1,780)	8	-0.15 (-0.24 to -0.06)*	-0.09 (-0.16 to -0.02)*	-0.22 (-0.40 to -0.03)*
Mental disorders	1,317 (978 to 1,727)	9	1,248 (922 to 1,627)	6	-0.27 (-0.34 to -0.20)*	-0.56 (-0.59 to -0.53)*	0.01 (-0.13 to 0.14)

For peer review only

For peer review only - http://bmjopen.bmj.com/site/about/guidelines.xhtml

Musculoskeletal disorders	1,536 (1,102 to 2,050)	8	1,585 (1,140 to 2,112)	3	0.16 (0.10 to 0.23)*	0.31 (0.26 to 0.36)*	0.02 (-0.11 to 0.15)
Other non-communicable diseases	2,315 (1,963 to 2,719)	5	1,349 (1,066 to 1,726)	4	-2.81 (-2.96 to -2.67)*	-3.49 (-3.65 to -3.34)*	-2.00 (-2.25 to -1.75)*
Skin and subcutaneous diseases	606 (397 to 898)	16	606 (391 to 909)	13	0.00 (-0.01 to 0.01)	-0.05 (-0.07 to -0.04)*	0.06 (0.03 to 0.08)*
Sense organ diseases	926 (633 to 1,299)	13	863 (591 to 1,226)	9	-0.36 (-0.45 to -0.27)*	-0.46 (-0.49 to -0.43)*	-0.25 (-0.43 to -0.07)*
Transport injuries	1,248 (1,146 to 1,433)	10	860 (740 to 975)	10	-1.98 (-2.42 to -1.54)*	-0.63 (-1.14 to -0.13)*	-3.39 (-4.12 to -2.65)*
Unintentional injuries	1,886 (1,744 to 2,045)	7	1,102 (919 to 1,254)	7	-2.81 (-3.24 to -2.39)*	-2.81 (-3.24 to -2.39)*	-2.81 (-3.24 to -2.39)*
Self-harm and interpersonal violence	807 (708 to 877)	15	361 (310 to 421)	16	-4.19 (-4.62 to -3.76)*	-5.13 (-5.93 to -4.32)*	-3.08 (-3.43 to -2.73)*
HIV/AIDS and sexually transmitted infections	78 (56 to 114)	22	116 (92 to 147)	19	2.02 (1.50 to 2.53)*	2.61 (2.31 to 2.91)*	1.88 (1.05 to 2.72)*
Respiratory infections and tuberculosis	2,396 (2,209 to 2,582)	4	518 (458 to 593)	15	-7.77 (-7.93 to -7.61)*	-9.56 (-9.70 to -9.41)*	-5.88 (-6.18 to -5.58)*
Enteric infections	369 (326 to 422)	19	111 (84 to 144)	20	-6.04 (-6.52 to -5.56)*	-10.47 (-11.00 to -9.94)*	-1.52 (-2.31 to -0.73)*
Other infectious diseases	473 (376 to 659)	17	110 (92 to 131)	21	-7.37 (-7.84 to -6.91)*	-8.51 (-9.21 to -7.80)*	-6.04 (-6.58 to -5.50)*
Maternal and neonatal disorders	2,177 (1,983 to 2,375)	6	792 (699 to 897)	12	-5.26 (-5.36 to -5.16)*	-5.26 (-5.36 to -5.16)*	-5.26 (-5.36 to -5.16)*
Substance use disorders	428 (345 to 526)	18	347 (262 to 443)	17	-1.03 (-1.38 to -0.69)*	-2.60 (-3.02 to -2.18)*	0.85 (0.35 to 1.36)*
Diabetes and kidney diseases	876 (764 to 997)	14	817 (688 to 961)	11	-0.32 (-0.47 to -0.16)*	0.46 (0.17 to 0.75)*	-1.01 (-1.12 to -0.90)*

Note: AAPC: average annual percent changes; UI, uncertainty interval; CI, confidence interval.
*AAPC significantly differs from zero at the alpha = 0.05 level.

For peer review only

sTable 6. The average annual percent change of risk factors for 2000–2019

Risk Factors	DALY (95% UI)				AAPC (95% CI)		
	2000	Rank	2019	Rank	2000–2019	2000–2009	2010–2019
**All-age numbers**							
Unsafe water, sanitation, and handwashing	4,234,714 (3,363,558 to 5,211,019)	15	1,152,504 (805,846 to 1,554,455)	18	-6.60 (-7.28 to -5.91)*	-10.80 (-11.04 to -10.55)*	-2.34 (-3.77 to -0.88)*
Air pollution	49,335,959 (43,328,149 to 55,138,636)	2	42,509,703 (36,337,918 to 49,482,918)	4	-0.78 (-0.98 to -0.58)*	-1.17 (-1.53 to -0.82)*	-0.46 (-0.68 to -0.24)*
Other environmental risks	5,386,702 (3,749,375 to 7,246,194)	14	6,306,070 (4,075,171 to 8,773,453)	13	0.85 (0.72 to 0.98)*	1.14 (0.91 to 1.38)*	0.46 (0.38 to 0.53)*
Child and maternal malnutrition	27,730,286 (24,920,872 to 30,776,034)	5	7,253,783 (6,277,469 to 8,340,678)	12	-6.81 (-7.62 to -5.99)*	-8.92 (-9.38 to -8.47)*	-4.75 (-6.37 to -3.10)*
Tobacco	57,264,585 (52,395,093 to 62,486,440)	1	64,116,000 (54,575,903 to 75,181,983)	1	0.60 (0.42 to 0.78)*	0.30 (0.02 to 0.58)*	0.90 (0.66 to 1.15)*
Alcohol use	14,673,585 (12,536,787 to 17,097,523)	8	17,265,060 (13,765,337 to 21,321,000)	8	0.77 (0.68 to 0.85)*	0.77 (0.68 to 0.85)*	0.77 (0.68 to 0.85)*
Drug use	6,301,278 (5,433,686 to 7,296,963)	13	5,121,794 (4,187,484 to 6,121,198)	14	-1.03 (-1.22 to -0.84)*	-3.28 (-3.50 to -3.07)*	1.54 (1.21 to 1.87)*
High fasting plasma glucose	17,924,145 (14,527,382 to 21,999,850)	7	28,228,439 (22,052,698 to 35,375,022)	5	2.36 (1.82 to 2.90)*	3.64 (2.83 to 4.47)*	1.11 (0.41 to 1.81)*
High systolic blood pressure	35,341,494 (30,227,215 to 40,383,079)	3	54,441,615 (45,474,002 to 63,689,070)	2	2.32 (2.18 to 2.46)*	2.66 (2.40 to 2.91)*	1.84 (1.77 to 1.92)*
High body-mass index	11,704,488 (3,880,854 to 22,527,461)	9	24,830,041 (11,788,976 to 40,545,899)	6	4.02 (3.74 to 4.29)*	4.33 (3.83 to 4.83)*	3.59 (3.48 to 3.70)*
Low bone mineral density	1,769,378 (1,477,619 to 2,061,186)	16	3,320,275 (2,594,480 to 4,004,265)	15	3.39 (3.12 to 3.67)*	4.14 (3.73 to 4.56)*	2.78 (2.42 to 3.15)*
Dietary risks	34,253,322 (27,427,086 to 43,429,953)	4	46,813,126 (35,644,728 to 60,011,858)	3	1.66 (1.53 to 1.78)*	1.97 (1.74 to 2.21)*	1.28 (1.21 to 1.35)*
Low physical activity	1,275,222 (632,556 to 2,484,126)	18	2,507,120 (1,195,139 to 4,842,346)	17	3.54 (2.70 to 4.38)*	3.88 (2.39 to 5.39)*	3.03 (2.65 to 3.41)*
Occupational risks	18,571,331 (16,040,002 to 21,379,719)	6	15,486,849 (12,897,016 to 18,259,216)	9	-0.97 (-1.12 to -0.82)*	-1.51 (-1.78 to -1.24)*	-0.40 (-0.52 to -0.29)*
Intimate partner violence	789,841 (433,187 to 1,234,095)	19	789,926,926 (354,381 to 1,304,433)	19	0.07 (-0.17 to 0.30)	-0.50 (-0.78 to -0.22)*	0.76 (0.39 to 1.14)*
Unsafe sex	1,482,696 (1,293,280 to 1,925,395)	17	2,724,797 (1,976,323 to 3,277,196)	16	3.20 (2.90 to 3.49)*	3.72 (3.50 to 3.94)*	2.95 (2.39 to 3.51)*
Non-optimal temperature	8,427,319 (7,191,510 to 9,779,073)	12	9,325,364 (7,641,727 to 11,224,758)	11	0.44 (-0.88 to 1.77)	-0.61 (-1.39 to 0.18)	1.14 (-1.01 to 3.34)
Kidney dysfunction	8,884,203 (7,881,193 to 10,012,008)	11	13,353,909 (11,150,932 to 15,771,793)	10	2.25 (1.83 to 2.67)*	3.04 (2.37 to 3.71)*	1.47 (0.91 to 2.03)*
High LDL cholesterol	11,217,788 (9,135,992 to 13,780,576)	10	19,813,962 (15,205,317 to 25,139,359)	7	3.11 (2.85 to 3.36)*	3.86 (3.39 to 4.33)*	2.23 (2.08 to 2.37)*
Childhood sexual abuse and bullying	762,744 (311,431 to 1,443,447)	20	646,857,857 (278,772 to 1,183,126)	20	-0.86 (-0.97 to -0.75)*	-1.17 (-1.29 to -1.04)*	-0.40 (-0.58 to -0.22)*
**Age-standardized rate**							
Unsafe water, sanitation, and handwashing	475 (376 to 587)	14	91 (64 to 122)	18	-8.23 (-8.79 to -7.67)*	-11.69 (-12.52 to -10.85)*	-4.58 (-5.22 to -3.93)*
Air pollution	4,860 (4,243 to 5,438)	2	2,265 (1,950 to 2,615)	4	-3.93 (-4.15 to -3.70)*	-4.08 (-4.49 to -3.68)*	-3.86 (-3.97 to -3.74)*
Other environmental risks	488 (338 to 653)	13	331 (213 to 460)	13	-2.00 (-2.13 to -1.87)*	-1.49 (-1.73 to -1.25)*	-2.64 (-2.71 to -2.56)*
Child and maternal malnutrition	3,285 (2,955 to 3,649)	4	786 (690 to 899)	10	-7.24 (-7.44 to -7.04)*	-8.34 (-8.58 to -8.10)*	-6.13 (-6.41 to -5.85)*
Tobacco	5,287 (4,858 to 5,765)	1	3,228 (2,759 to 3,767)	1	-2.57 (-2.83 to -2.32)*	-2.80 (-3.20 to -2.40)*	-2.33 (-2.68 to -1.98)*
Alcohol use	1,137 (972 to 1,328)	8	908 (729 to 1,116)	8	-1.18 (-1.50 to -0.86)*	-1.41 (-1.54 to -1.28)*	-0.92 (-1.56 to -0.27)*
Drug use	449 (390 to 518)	15	295 (238 to 361)	14	-2.11 (-2.74 to -1.48)*	-4.70 (-4.97 to -4.43)*	0.85 (-0.48 to 2.19)
High fasting plasma glucose	1,647 (1,338 to 2,051)	6	1,452 (1,139 to 1,832)	5	-0.72 (-1.23 to -0.20)*	0.72 (-0.06 to 1.51)	-2.11 (-2.76 to -1.44)*
High systolic blood pressure	3,304 (2,820 to 3,777)	3	2,844 (2,392 to 3,321)	2	-0.77 (-0.89 to -0.65)*	-0.29 (-0.51 to -0.07)*	-1.40 (-1.47 to -1.34)*
High body-mass index	962 (315 to 1,891)	10	1,231 (578 to 2,023)	6	1.28 (0.95 to 1.60)*	1.50 (0.91 to 2.10)*	0.94 (0.82 to 1.07)*

For peer review only - http://bmjopen.bmj.com/site/about/guidelines.xhtml

For peer review only

Low bone mineral density	164 (137 to 192)	16	177 (138 to 214)	15	0.40 (0.27 to 0.54)*	0.70 (0.55 to 0.86)*	0.20 (-0.02 to 0.42)
Dietary risks	3,022 (2,391 to 3,875)	5	2,394 (1,823 to 3,071)	3	-1.23 (-1.36 to -1.09)*	-0.78 (-1.03 to -0.52)*	-1.75 (-1.83 to -1.68)*
Low physical activity	149 (74 to 287)	17	146 (70 to 280)	16	-0.19 (-0.74 to 0.36)	0.48 (0.17 to 0.79)*	-0.93 (-2.03 to 0.18)
Occupational risks	1,482 (1,277 to 1,700)	7	844 (701 to 997)	9	-2.95 (-3.08 to -2.81)*	-3.67 (-3.88 to -3.45)*	-2.22 (-2.37 to -2.07)*
Intimate partner violence	55 (30 to 86)	19	46 (22 to 76)	20	-0.86 (-1.11 to -0.62)*	-1.68 (-1.94 to -1.41)*	0.09 (-0.32 to 0.50)
Unsafe sex	108 (94 to 141)	18	142 (106 to 170)	17	1.47 (0.88 to 2.06)*	1.83 (1.43 to 2.23)*	1.34 (0.19 to 2.51)*
Non-optimal temperature	936 (813 to 1,075)	11	483 (399 to 582)	12	-3.61 (-4.04 to -3.18)*	-4.39 (-5.06 to -3.71)*	-2.91 (-3.41 to -2.42)*
Kidney dysfunction	781 (690 to 882)	12	709 (593 to 832)	11	-0.45 (-0.97 to 0.08)	054 (-0.31 to 1.39)	-1.41 (-2.10 to -0.72)*
High LDL cholesterol	1,005 (790 to 1,284)	9	1,052 (800 to 1,345)	7	0.28 (0.10 to 0.45)*	1.19 (0.87 to 1.51)*	-0.75 (-0.85 to -0.66)*
Childhood sexual abuse and bullying	54 (22 to 102)	20	50 (20 to 91)	19	-0.41 (-0.51 to -0.31)*	-1.05 (-1.21 to -0.89)*	0.40 (0.31 to 0.48)*

Note: AAPC: average annual percent changes; UI, uncertainty interval; CI, confidence interval; LDL, low-density lipoprotein.
*AAPC significantly differs from zero at the alpha = 0.05 level.

For peer review only

STROBE Statement—Checklist of items that should be included in reports of ***cohort studies***

	Item No	Recommendation
**Title and abstract**	1	(*a*) Indicate the study's design with a commonly used term in the title or the abstract. (Line 28-30, page 3)
		(*b*) Provide in the abstract an informative and balanced summary of what was done and what was found. (Line 41-56, page 3-4)
**Introduction**		
Background/rationale	2	Explain the scientific background and rationale for the investigation being reported. (Line73-75, page 5)
Objectives	3	State specific objectives, including any prespecified hypotheses. (Line105-109, page 6)
**Methods**		
Study design	4	Present key elements of study design early in the paper
Setting	5	Describe the setting, locations, and relevant dates, including periods of recruitment, exposure, follow-up, and data collection
Participants	6	(*a*) Give the eligibility criteria, and the sources and methods of selection of participants. Describe methods of follow-up
		(*b*) For matched studies, give matching criteria and number of exposed and unexposed
Variables	7	Clearly define all outcomes, exposures, predictors, potential confounders, and effect modifiers. Give diagnostic criteria, if applicable
Data sources/ measurement	8*	For each variable of interest, give sources of data and details of methods of assessment (measurement). Describe comparability of assessment methods if there is more than one group. (Line 112-123, page 6-7)
Bias	9	Describe any efforts to address potential sources of bias
Study size	10	Explain how the study size was arrived at
Quantitative variables	11	Explain how quantitative variables were handled in the analyses. If applicable, describe which groupings were chosen and why
Statistical methods	12	(*a*) Describe all statistical methods, including those used to control for confounding. (Line159-164, page 8-9)
		(*b*) Describe any methods used to examine subgroups and interactions. (Line164-171, page 9)
		(*c*) Explain how missing data were addressed
		(*d*) If applicable, explain how loss to follow-up was addressed
		(*e*) Describe any sensitivity analyses
**Results**		
Participants	13*	(a) Report numbers of individuals at each stage of study—eg numbers potentially eligible, examined for eligibility, confirmed eligible, included in the study, completing follow-up, and analysed.
		(b) Give reasons for non-participation at each stage. (Line176-177， page 9 and line 200-201, page 12 and line 222-224 page 14)
		(c) Consider use of a flow diagram
Descriptive data	14*	(a) Give characteristics of study participants (eg demographic, clinical, social) and information on exposures and potential confounders. (Line176-177， page 9)
		(b) Indicate number of participants with missing data for each variable of interest
		(c) Summarise follow-up time (eg, average and total amount)
Outcome data	15*	Report numbers of outcome events or summary measures over time. (Line 175，

For peer review only - http://bmjopen.bmj.com/site/about/guidelines.xhtml

		page 9 and line 199, page 11 and line 221, page 14)
Main results	16	(*a*) Give unadjusted estimates and, if applicable, confounder-adjusted estimates and their precision (eg, 95% confidence interval). Make clear which confounders were adjusted for and why they were included
		(*b*) Report category boundaries when continuous variables were categorized
		(*c*) If relevant, consider translating estimates of relative risk into absolute risk for a meaningful time period
Other analyses	17	Report other analyses done—eg analyses of subgroups and interactions, and sensitivity analyses
**Discussion**		
Key results	18	Summarise key results with reference to study objectives. (line294-297, page22)
Limitations	19	Discuss limitations of the study, taking into account sources of potential bias or imprecision. Discuss both direction and magnitude of any potential bias. (line373-385, page25-26)
Interpretation	20	Give a cautious overall interpretation of results considering objectives, limitations, multiplicity of analyses, results from similar studies, and other relevant evidence
Generalisability	21	Discuss the generalisability (external validity) of the study results
**Other information**		
Funding	22	Give the source of funding and the role of the funders for the present study and, if applicable, for the original study on which the present article is based. (line401-405, page27)

*Give information separately for exposed and unexposed groups.

Note: An Explanation and Elaboration article discusses each checklist item and gives methodological background and published examples of transparent reporting. The STROBE checklist is best used in conjunction with this article (freely available on the Web sites of PLoS Medicine at http://www.plosmedicine.org/, Annals of Internal Medicine at http://www.annals.org/, and Epidemiology at http://www.epidem.com/). Information on the STROBE Initiative is available at http://www.strobe-statement.org.

For peer review only
